# Supplementary material for: Selective nitrogen insertion into aryl alkanes
Source: Nat Commun. 2024 Jul 17;15:6016. doi: 10.1038/s41467-024-50383-0 (PMC11255249; doi:10.1038/s41467-024-50383-0)
Supplement: Supplementary file 1 — Supplementary Information [file 41467_2024_50383_MOESM1_ESM.pdf]

Supplementary Information

for

Selective Nitrogen Insertion into Aryl Alkanes

Zheng Zhang,[1] Qi Li,[1] Zengrui Cheng,[2] Ning Jiao,*[,2] and Chun Zhang*[,1]

1. Institute of Molecular Plus, Tianjin Key Laboratory of Molecular Optoelectronic Science, Department of Chemistry, School of Pharmaceutical Science and Technology.
Tianjin University.
Tianjin, 300072, China.
2. State Key Laboratory of Natural and Biomimetic Drugs, Chemical Biology Center, School of Pharmaceutical Sciences.
Peking University.
Beijing, 100191, China.
* Correspondence: jiaoning@pku.edu.cn; chunzhang@tju.edu.cn

Table of contents

Supplementary methods.....2
1. General Considerations.....2
2. General procedure for the reaction.....3
3. The effect of different reaction conditions.....4
4. Synthesis of substrates.....8
5. Analytical data for compounds.....23
6. Further transformations.....43
7. Mechanistic studies.....60
8. NMR Spectra.....77
Supplementary references.....127

Supplementary methods

1. General Considerations

All manipulations were conducted with Schlenk tube. ^{1}H-NMR spectras were recorded on BrukerAVIII-400 spectrometers or JNM-ECZ400S/L1 spectrometers. Chemical shifts (in ppm) were referenced to TMS (δ = 0 ppm) in Chloroform-d as an internal standard. Data were reported as follows: chemical shift (ppm), multiplicity (s = singlet, d = doublet, t = triplet, q = quartet, dd = doublet of doublets, m = multiplet), coupling constants (Hz), integration and assignment. ^{13}C-NMR spectras were obtained by using the same NMR spectrometers and were calibrated by Chloroform-d (δ = 77.00 ppm). High resolution mass spectrometry (HRMS) data were obtained on a QTOF mass analyzer with electrospray ionization (ESI) through a Waters Acquity UPLC Class I/Xevo G2 Q-Tof. Substrates were purchased from Aldrich, TCI, Acros, Energy, Aladdin, or synthesized according to the procedures outlined below. THF was distilled from sodium benzophenone prior to use. Unless otherwise noted, materials obtained from commercial suppliers were used without further purification. For the heating reaction, the oil bath was used as heat source.

2. General procedure for the reaction

General procedure A for the selective ring-opening amination.

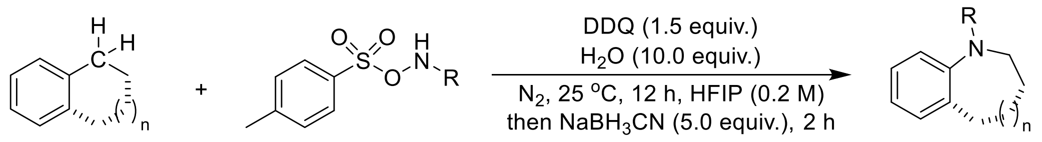

In an oven dried 25 mL Schlenk tube, which containing a stirring bar, was charged with aminating reagent (0.45 mmol, 1.5 equiv.), DDQ (0.45 mmol, 102 mg, 1.5 equiv.). Then the tube was evacuated and back-filled under N_2 flow (this sequence was repeated three times). H_2O (3.0 mmol, 0.054 mL, 10.0 equiv.), HFIP (1.5 mL) and benzyl compounds (0.3 mmol, 1.0 equiv.) were added and stirred at room temperature for 12 h. Then $NaBH_3CN$ (1.5 mmol, 96 mg, 5.0 equiv.) was added to the above reaction mixture and the reaction was stirred for 2 h at room temperature. The reaction was quenched with 2.0 mL saturated $NaHCO_3$ aq. and 3.0 mL H_2O. Then it was extracted with DCM (3.0 mL × 3). The organic layer was combined and dried over Na_2SO_4. Then filtered and concentrated by rotary evaporation. The residue was purified by silica gel chromatography (EtOAc/petroleum ether) to afford the product.

3. The effect of different reaction conditions

Supplementary Table 1: The effect of different oxidizing reagents.[a]

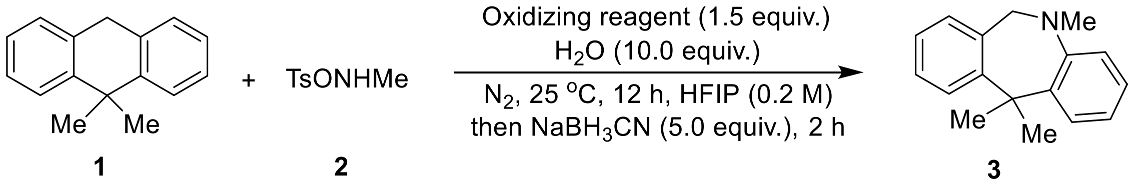

Entry	Oxidizing reagent	Yield (%)[b]
1	1,2-Dichloro-4,5-Dicyanobenzoquinone (DDQ)	98
2	1,4-Benzoquinone	26
3	Chloranil	40
4	Anthraquinone	41
5	TBHP	NR
6	H_2O_2	NR
7	None	NR

[a] Reaction conditions: **1** (0.3 mmol), **2** (0.45 mmol), oxidizing reagent (0.45 mmol), H_2O (3.0 mmol), HFIP (1.5 mL), N_2, 25 °C, 12 h, then $NaBH_3CN$ (1.5 mmol), 2 h. [b] Isolated yield.

Supplementary Table 2: The effect of different solvents.[a]

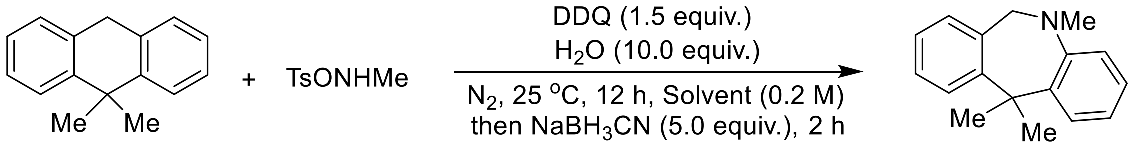

1 2 3

Entry	Solvent	Yield (%)[b]
1	HFIP	98
2	DCM	88
3	^{i}PrOH	41
4	THF	41
5	DMSO	NR
6	CF_3CH_2OH	95

[a] Reaction conditions: **1** (0.3 mmol), **2** (0.45 mmol), DDQ (0.45 mmol), H_2O (3.0 mmol), solvent (1.5 mL), N_2, 25 °C, 12 h, then $NaBH_3CN$ (1.5 mmol), 2 h. [b] Isolated yield.

Supplementary Table 3: The effect of different reducing reagents.[a]

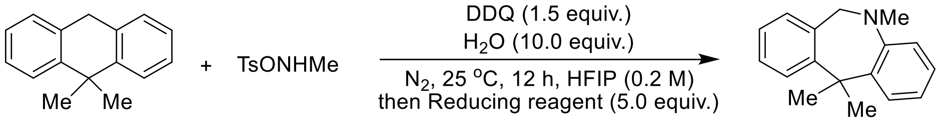

1 2 3

Entry	Reducing reagents	Yield (%)[b]
1	$NaBH_3CN$	98
2	$NaBH_4$	80
3	HBpin	91
4	$(Et)_3SiH$	trace
5	none	ND

[a] Reaction conditions: **1** (0.3 mmol), **2** (0.45 mmol), DDQ (0.45 mmol), H_2O (3.0 mmol), HFIP (1.5 mL), N_2, 25 °C, 12 h, then reducing reagent (1.5 mmol), 2 h. [b] Isolated yield.

Supplementary Table 4: The effect of different temperature.[a]

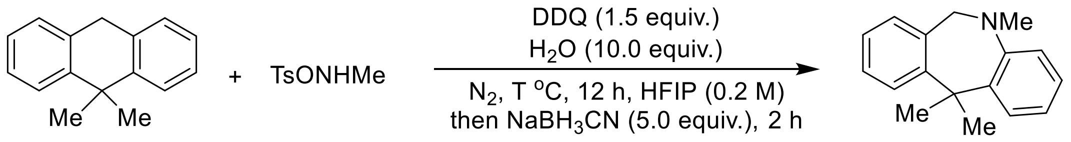

Entry	Temperature	Yield (%)[b]
1	0 °C	57
2	25 °C	98
3	40 °C	95
4	60 °C	96

[a] Reaction conditions: **1** (0.3 mmol), **2** (0.45 mmol), DDQ (0.45 mmol), H_2O (3.0 mmol), HFIP (1.5 mL), N_2, T °C, 12 h, then $NaBH_3CN$ (1.5 mmol), 2 h. [b] Isolated yield.

Supplementary Table 5: The effect of H_2O or air.[a]

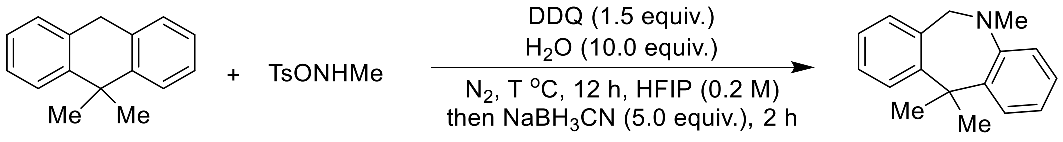

Entry	deviation from standard condition	Yield (%)[b]
1	none	98
2	w/o water	88
3	under air	87

[a] Reaction conditions: **1** (0.3 mmol), **2** (0.45 mmol), DDQ (0.45 mmol), H_2O (3.0 mmol), HFIP (1.5 mL), N_2, 25 °C, 12 h, then $NaBH_3CN$ (1.5 mmol), 2 h. [b] Isolated yield.

Supplementary Table 6: The effect of TFA as solvent or co-solvent.[a]

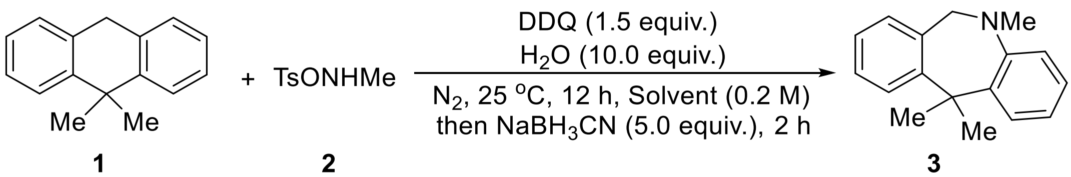

Entry	Solvent	Yield (%)[b]
1	TFA	47
2	TFA : HFIP = 2:1	31
3	TFA : HFIP = 1:1	41
4	TFA : HFIP = 1:2	61
5	HFIP	98

[a] Reaction conditions: **1** (0.3 mmol), **2** (0.45 mmol), DDQ (0.45 mmol), H_2O (3.0 mmol), Solvent (1.5 mL), N_2, 25 ^oC, 12 h, then $NaBH_3CN$ (1.5 mmol), 2 h. [b] Isolated yield.

4. Synthesis of substrates

4.1 Synthesis of benzyl compounds

All the benzyl compounds are known compounds. Several benzyl compounds (9,9-dimethyl-9,10-dihydroanthracene, 1-methyl-1,2,3,4-tetrahydronaphthalene, 5-phenyl-1,2,3,4-tetrahydronaphthalene, 6-phenyl-1,2,3,4-tetrahydronaphthalene, N-(4-isopropylphenyl)-4-methylbenzenesulfonamide and 5-isopropylbenzofuran) were prepared according to the corresponding literature reports.[1,3,4] Other benzyl compounds are commercially available. Analytical data (^{1}H NMR, ^{13}C NMR) matches with the literature.[2-5] For unknown compounds, the analytical data as below.

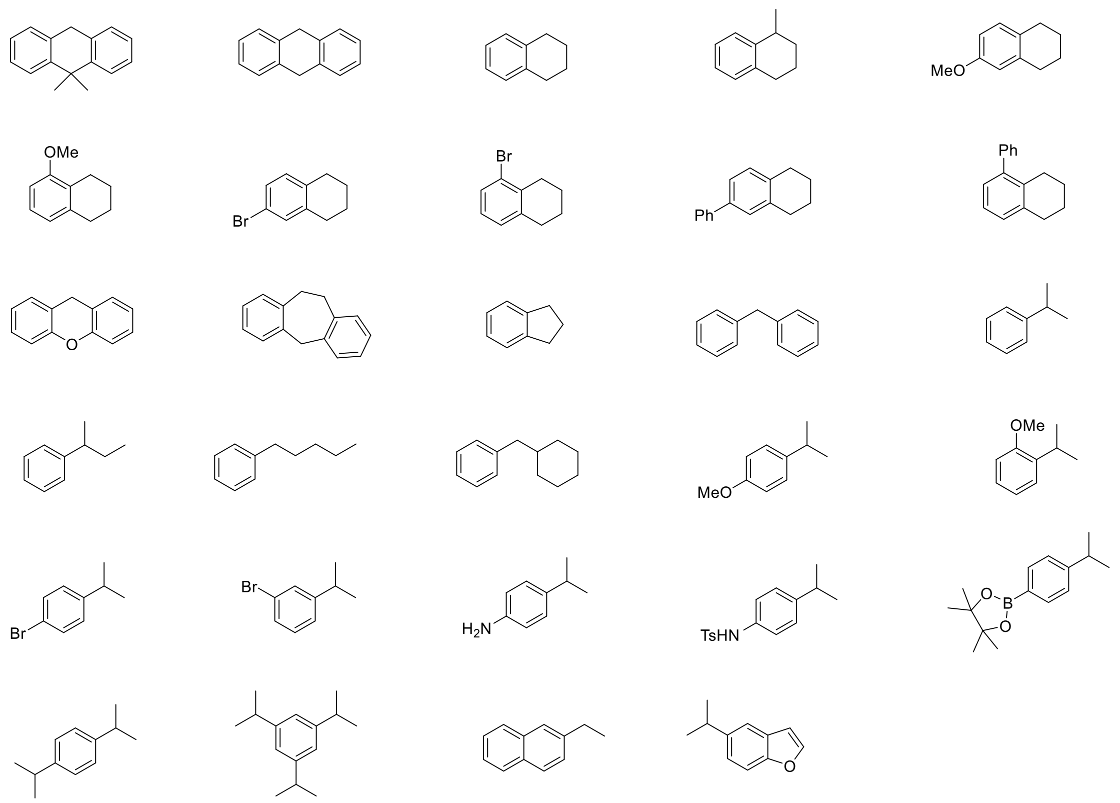

Supplementary Fig 1. Benzyl compounds used in this work

General Procedure B for synthetizing 9,9-dimethyl-9,10-dihydroanthracene and 1-methyl-1,2,3,4-tetrahydronaphthalene.

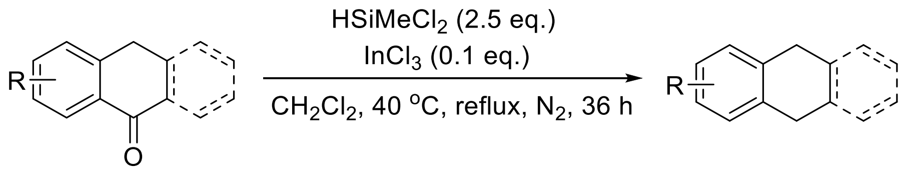

Supplementary Fig 2. Synthesis of 9,9-dimethyl-9,10-dihydroanthracene and 1-methyl-1,2,3,4-tetrahydronaphthalen

Me_2SiClH (12.5 mmol) was added to a solution of $InCl_3$ (0.5 mmol) and aryl ketone (5 mmol) in CH_2Cl_2 (5 mL) at room temperature under N_2 atmosphere. Subsequently, it was heated to 40 ^oC and subjected to reflux reaction for 36 h. After the completion of the reaction, the mixture was washed with water (2 × 50 ml) and extracted with ether (50 mL). After dried over $MgSO_4$, filtered and concentrated by rotary evaporation. The residue was purified by silica gel chromatography to afford the product. 9,9-dimethyl-9,10-dihydroanthracene[2], 1-methyl-1,2,3,4-tetrahydronaphthalene[3] are literature reported compounds.

General Procedure for synthetizing 5-phenyl-1,2,3,4-tetrahydronaphthalene and 6-phenyl-1,2,3,4-tetrahydronaphthalene.

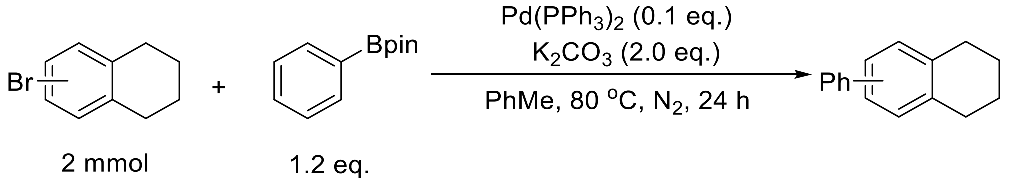

Supplementary Fig 3. Synthesis of 5-phenyl-1,2,3,4-tetrahydronaphthalene and 6-phenyl-1,2,3,4-tetrahydronaphthalen

6-bromo-1,2,3,4-tetrahydronaphthalene or 7-bromo-1,2,3,4-tetrahydronaphthalene (2.0 mmol) was added to a solution of (4,4,5,5-Tetramethyl-1,3,2-dioxaborolan-2-yl)benzene (2.4 mmol), $Pd(PPh_3)_2$ (0.2 mmol) and K_2CO_3 (4.0 mmol) in Toluene (3 mL) at room temperature under N_2 atmosphere. Subsequently, it was heated to 80 ^oC and reacted for 24 h. After the completion of the reaction, the mixture was washed with water (2 × 20 ml) and extracted with ether (20 mL). After dried over $MgSO_4$, filtered and concentrated by rotary evaporation. The residue was purified by silica gel chromatography to afford the product. 5-phenyl-1,2,3,4-tetrahydronaphthalene[5], 6-phenyl-1,2,3,4-tetrahydronaphthalene[5] are literature reported compounds.

General Procedure for synthetizing N-(4-isopropylphenyl)-4-methylbenzenesulfonamide.

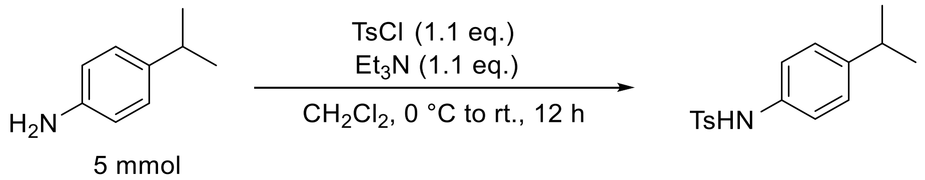

Supplementary Fig 4. Synthesis of N-(4-isopropylphenyl)-4-methylbenzenesulfonamide

4-isopropylaniline (5.0 mmol) and Et_3N (5.0 mmol) were added to a solution of TsCl (5.5 mmol) in CH_2Cl_2 (20 mL) at 0 ^oC. Subsequently, it was heated to room temeprature and reacted for 12 h. After the completion of the reaction, the mixture was washed with water (2 ×50 ml) and extracted with ether (50 mL). After dried over $MgSO_4$, filtered and concentrated by rotary evaporation. The residue was purified by silica gel chromatography (EtOAc/petroleum ether= 1:5) to afford the product

N-(4-isopropylphenyl)-4-methylbenzenesulfonamide (82%). N-(4-isopropylphenyl)-4-methylbenzenesulfonamide [4] is literature reported compound.

General Procedure for synthetizing 5-isopropylbenzofuran.

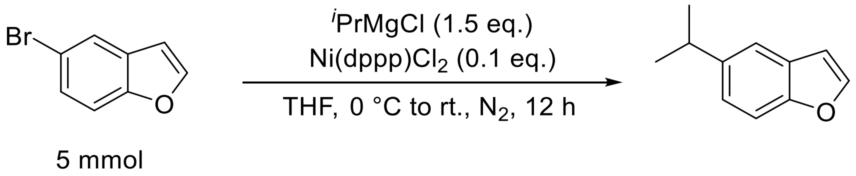

Supplementary Fig 5. Synthesis of 5-isopropylbenzofuran

^{i}PrMgCl (7.5 mmol, 2.0 M in THF) was added to a solution of Ni(dppp)Cl_2 (0.5 mmol) and 5-bromobenzofuran (5.0 mmol) in THF (5 mL) at 0 ^{o}C under N_2 atmosphere. Subsequently, it was heated to room temeprature and reacted for 12 h. After the completion of the reaction, the mixture was washed with water (2 × 50 ml) and extracted with ether (50 mL). After dried over $MgSO_4$, filtered and concentrated by rotary evaporation. The residue was purified by silica gel chromatography (petroleum ether) to afford the product.

Analytical data for synthetized known compounds:

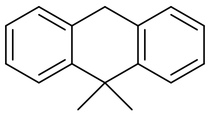

9,9-dimethyl-9,10-dihydroanthracene: The procedure was followed the procedure and afforded product as a white solid (96% yield). **R_f** = 0.7 (petroleum ether). **^{1}H NMR** (400 MHz, $CDCl_3$) *δ* 7.57 (d, *J* = 8.4 Hz, 2H), 7.32-7.27 (m, 4H), 7.22 (dt, *J* = 7.8, 1.2 Hz, 2H), 4.12 (s, 2H), 1.65 (s, 6H) ppm.

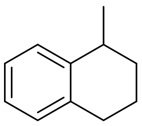

1-methyl-1,2,3,4-tetrahydronaphthalene: The procedure was followed the procedure and afforded product as a colorless oil (91% yield). **R_f** = 0.7 (petroleum ether). **^{1}H NMR** (400 MHz, $CDCl_3$) *δ* 7.24-7.21 (1H, m), 7.17-7.06 (3H, m), 2.97-2.89 (1H, m), 2.85-2.70 (2H, m), 1.99-1.86 (2H, m), 1.79-1.72 (1H, m), 1.60-1.54 (1H, m), 1.31 (3H, d, *J* = 7.3 Hz) ppm. **HRMS** (ESI-TOF) m/z calcd for $C_{11}H_{15}$ $(M + H)^+$: 147.1174, found 147.1173.

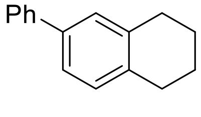

5-phenyl-1,2,3,4-tetrahydronaphthalene: The procedure was followed the

procedure and afforded product as a colorless oil (96% yield). $\mathbf{R_f}$ = 0.7 (petroleum ether). **^{1}H NMR** (400 MHz, $CDCl_3$) δ 7.59 – 7.54 (m, 2H), 7.41 (t, J = 7.6 Hz, 2H), 7.34 – 7.27 (m, 3H), 7.13 (d, J = 7.8 Hz, 1H), 2.89 – 2.73 (m, 4H), 1.83 (p, J = 3.3 Hz, 4H) ppm. **HRMS** (ESI-TOF) m/z calcd for $C_{16}H_{17}$ $(M + H)^+$: 209.1330, found 209.1330.

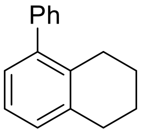

6-phenyl-1,2,3,4-tetrahydronaphthalene: The procedure was followed the procedure and afforded product as a colorless oil (98% yield). $\mathbf{R_f}$ = 0.7 (petroleum ether). **^{1}H NMR** (400 MHz, $CDCl_3$) δ 7.40 – 7.34 (m, 2H), 7.32 – 7.25 (m, 3H), 7.17 – 6.98 (m, 3H), 2.84 (t, J = 6.4 Hz, 2H), 2.57 (t, J = 6.3 Hz, 2H), 1.83 – 1.62 (m, 4H) ppm. **HRMS** (ESI-TOF) m/z calcd for $C_{16}H_{17}$ $(M + H)^+$: 209.1330, found 209.1329.

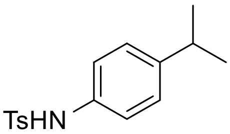

N-(4-isopropylphenyl)-4-methylbenzenesulfonamide: The procedure was followed the procedure and afforded product as a white solid (82% yield). $\mathbf{R_f}$ = 0.5 (EtOAc/petroleum ether= 1:5). **^{1}H NMR** (400 MHz, $CDCl_3$) δ 7.67 (d, J = 8.0 Hz, 2 H), 7.21 (d, J = 7.6 Hz, 2 H), 7.07 (d, J = 8.0 Hz, 2 H), 6.96 (d, J = 8.4 Hz, 2 H), 6.94 (s, 1 H), 2.87-2.77 (m, 1 H), 2.37 (s, 3 H), 1.18 (d, J = 7.2 Hz, 6 H) ppm. **HRMS** (ESI-TOF) m/z calcd for $C_{16}H_{20}O_2NS$ $(M + H)^+$: 290.1209, found 209.1205.

Analytical data for novel compound:

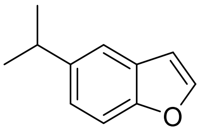

5-isopropylbenzofuran: The procedure was followed the reported literature and afforded product as a colorless oil (65% yield). $\mathbf{R_f}$ = 0.6 (petroleum ether). **^{1}H NMR** (400 MHz, $CDCl_3$) δ 7.57 – 7.54 (m, 1H), 7.43 – 7.39 (m, 2H), 7.18 – 7.13 (m, 1H), 6.70 – 6.68 (m, 1H), 3.06 – 2.93 (m, 1H), 1.29 (d, J = 7.0 Hz, 6H) ppm. **^{13}C NMR** (101 MHz, $CDCl_3$) δ 153.51, 145.00, 143.48, 127.37, 123.13, 118.22, 110.96, 106.47, 34.08, 24.53 ppm. **HRMS** (ESI-TOF) m/z calcd for $C_{11}H_{11}O$ $(M - H)^-$: 159.0810, found 159.0814.

4.2 Synthesis of amination reagents:

All the aminating reagents were prepared according to the reported literature.[6] For known compounds, their analytical data (^{1}H NMR, ^{13}C NMR) match with the data of corresponding literature.[6] For unknown compounds, the analytical data as below.

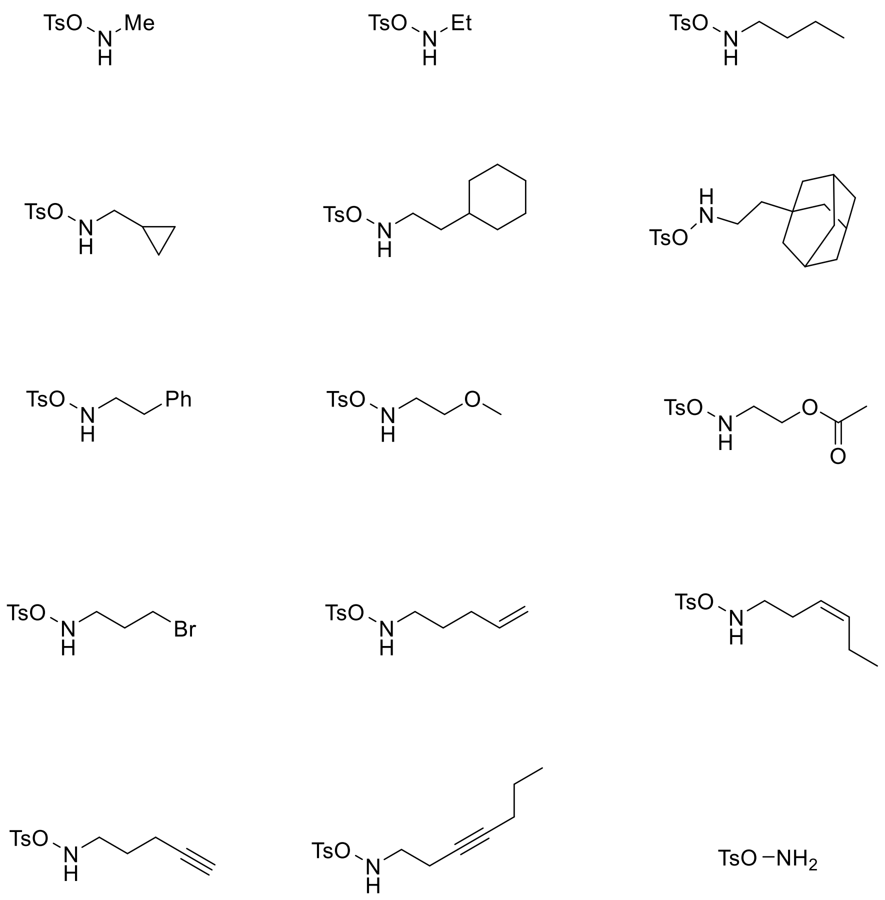

Supplementary Fig 6. Amination reagents used in this work

General Procedure C for synthetizing amination reagents

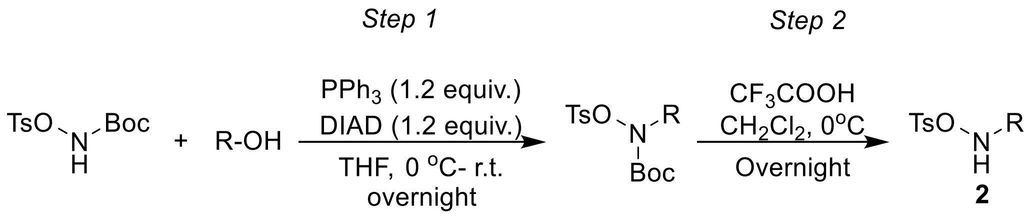

Supplementary Fig 7. Synthesis of amination reagents

In an oven dried 50 mL round bottom flask, which equipped with a stir bar was added PPh_3 (3.14g, 12.0 mmol, 1.2 equiv.). The flask was then evacuated and back-filled under N_2 flow (this sequence was repeated three times). Dry THF (12.0 mL) and DIAD (2.4 mL, 12.0 mmol, 1.2 equiv.) was added sequentially at 0 °C under nitrogen atmosphere. A white solid was formed quickly, and then it's stirred for 30 min at 0 °C. Then the solution of alcohol (10.0 mmol, 1.0 equiv.) in THF (4.0 mL) and TsONHBoc (2.87g, 10.0 mmol, 1.0 equiv.) in THF (4.0 mL) were added to the above

mixture successively. Then the reaction was stirred at 0 ^{o}C for another 2 h, the resulting mixture was then warmed to 25 ^{o}C (oil bath) and stirred overnight (monitored by TLC). The reaction mixture diluted with 10.0 mL H_2O. Then it was extracted with DCM (10.0 mL × 3). The organic layer was combined and dried over anhydrous Na_2SO_4. Then filtered and concentrated by rotary evaporation. The residue was purified by silica gel chromatography (EtOAc/petroleum ether= 1:10) to afford the TsONBocR.

To a solution of N-Boc-N-alkyl-O-tosyl hydroxylamine (1.0 equiv.) in DCM (1.0 M) was added TFA (20.0 equiv.) at 0 ^{o}C, then it was stirred at 0 ^{o}C overnight (monitored by TLC). The reaction was quenched with cold water (10.0 mL) at 0 ^{o}C and then extracted with DCM (10.0 mL × 3). The organic layer was combined and dried over anhydrous Na_2SO_4. Then filtered and concentrated by rotary evaporation. The residue was purified by silica gel chromatography (EtOAc/petroleum ether= 1:5) to afford the product[6].

Analytical data for synthetized known compounds[6]:

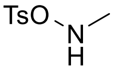

N-methyl-O-tosylhydroxylamine: The procedure was followed the Procedure **C** and afforded product as a white solid (58% yield). $\mathbf{R_f}$ = 0.4 (EtOAc : petroleum ether = 1 : 5). **^{1}H NMR** (400 MHz, $CDCl_3$) *δ* 7.82 (d, *J* = 8.2 Hz, 2H), 7.32 (d, *J* = 8.2 Hz, 2H), 5.85 (s, 1H), 2.71 (s, 3H), 2.44 (s, 3H) ppm. **HRMS** (ESI-TOF) m/z calcd for $C_8H_{12}NO_3S$ $(M + H)^+$: 202.0538, found 202.0532.

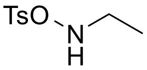

N-ethyl-O-tosylhydroxylamine: The procedure was followed the Procedure **C** and afforded product as a white solid (42% yield). $\mathbf{R_f}$ = 0.4 (EtOAc : petroleum ether = 1 : 5). **^{1}H NMR** (400 MHz, $CDCl_3$) *δ* 7.85 (d, *J* = 8.3 Hz, 2H), 7.40 – 7.29 (m, 2H), 5.96 (brs, 1H), 2.99 (q, *J* = 7.1 Hz, 2H), 2.46 (s, 3H), 0.96 (t, *J* = 7.1 Hz, 3H) ppm. **HRMS** (ESI-TOF) m/z calcd for $C_9H_{14}NO_3S$ $(M + H)^+$: 216.0694, found 216.0689.

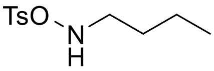

N-butyl-O-tosylhydroxylamine: The procedure was followed the reported literature and afforded product as a yellow oil (35% yield). $\mathbf{R_f}$ = 0.5 (EtOAc : petroleum ether = 1 : 5). **^{1}H NMR** (400 MHz, $CDCl_3$) *δ* 7.84 (d, *J* = 8.2 Hz, 2H), 7.33 (d, *J* = 8.2 Hz, 2H), 2.45 (s, 3H), 1.88 – 1.24 (m, 6H), 0.90 (t, *J* = 7.3 Hz, 3H) ppm. **HRMS** (ESI-TOF) m/z calcd for $C_{11}H_{18}NO_3S$ $(M + H)^+$: 244.1007, found 244.1009.

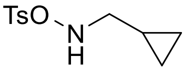

N-(cyclopropylmethyl)-O-tosylhydroxylamine: The procedure was followed the

reported literature and afforded product as a white solid (51% yield). **R_f** = 0.4 (EtOAc : petroleum ether = 1 : 5). **^{1}H NMR** (400 MHz, $CDCl_3$) δ 7.72 (d, J = 8.2 Hz, 2H), 7.15 (d, J = 8.0 Hz, 2H), 2.45 – 2.41 (m, 2H), 2.35 (s, 3H), 2.05 (s, 1H), 1.26 (t, J = 7.1 Hz, 2H), 0.56 (d, J = 7.9 Hz, 2H) ppm. **HRMS** (ESI-TOF) m/z calcd for $C_{11}H_{16}NO_3S$ $(M + H)^+$: 242.0851, found 242.0858.

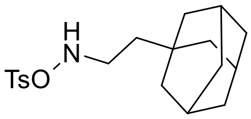

N-(2-(adamantan-1-yl)ethyl)-O-tosylhydroxylamine: The procedure was followed the reported literature and afforded product as a white solid (56% yield). **R_f** = 0.6 (EtOAc : petroleum ether = 1 : 5). **^{1}H NMR** (400 MHz, $CDCl_3$) δ 7.78 (d, J = 8.1 Hz, 2H), 7.17 (d, J = 7.9 Hz, 2H), 2.45 (s, 4H), 2.35 (s, 3H), 1.43 (d, J = 6.1 Hz, 9H), 1.34 z(d, J = 2.8 Hz, 3H), 1.28 – 1.22 (m, 2H), 1.16 (d, J = 2.7 Hz, 1H), 1.09 – 1.01 (m, 1H) ppm. **HRMS** (ESI-TOF) m/z calcd for $C_{19}H_{28}NO_3S$ $(M + H)^+$: 350.1790, found 350.1799.

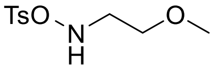

N-(2-methoxyethyl)-O-tosylhydroxylamine: The procedure was followed the Procedure **C** and afforded product as a white solid (59% yield). **R_f** = 0.3 (EtOAc : petroleum ether = 1 : 5). **^{1}H NMR** (400 MHz, $CDCl_3$) δ 7.86 (d, J = 8.4 Hz, 2H), 7.38 – 7.30 (m, 2H), 5.66 (brs, 1H), 3.44 (dd, J = 5.4, 4.4 Hz, 2H), 3.30 (s, 3H), 3.11 (dd, J = 5.3, 4.6 Hz, 2H), 2.45 (s, 3H) ppm. **HRMS** (ESI-TOF) m/z calcd for $C_{10}H_{16}NO_4S$ $(M + H)^+$: 246.0800, found 246.0807.

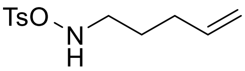

N-(pent-4-en-1-yl)-O-tosylhydroxylamine: The procedure of was followed the Procedure **C** and afforded product as a white oil (42% yield). **R_f** = 0.4 (EtOAc : petroleum ether = 1 : 5). **^{1}H NMR** (400 MHz, $CDCl_3$) δ 7.73 (d, J = 8.1 Hz, 2H), 7.18 (d, J = 7.9 Hz, 2H), 5.12 – 4.92 (m, 3H), 2.47 – 2.38 (m, 2H), 2.36 (s, 3H), 2.15 – 1.99 (m, 2H), 1.83 – 1.56 (m, 2H) ppm. **HRMS** (ESI-TOF) m/z calcd for $C_{12}H_{18}NO_3S$ $(M + H)^+$: 256.1007, found 256.1017.

Analytical data for novel compounds:

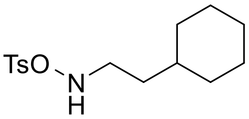

N-(2-cyclohexylethyl)-O-tosylhydroxylamine: The procedure was followed the reported literature and afforded product as a white oil (56% yield). **R_f** = 0.6 (EtOAc : petroleum ether = 1 : 5). **^{1}H NMR** (400 MHz, $CDCl_3$) δ 7.84 – 7.80 (m, 2H), 7.32 –

7.30 (d, J = 8.2 Hz, 2H), 6.32 (brs, 1H), 2.93 – 2.90 (t, J = 7.2 Hz, 2H), 2.43 (s, 3H), 1.62 – 1.56 (m, 4H), 1.52 – 1.45 (m, 2H), 1.15 – 1.00 (m, 5H), 0.79 – 0.69 (m, 2H) ppm. **^{13}C NMR** (101 MHz, $CDCl_3$) δ 144.8, 132.1, 129.4, 129.0, 50.2, 34.9, 33.7, 32.9, 26.3, 26.0, 21.6 ppm. **HRMS** (ESI-TOF) m/z calcd for $C_{15}H_{24}NO_3S$ (M + H)$^+$: 298.1477, found 298.1474.

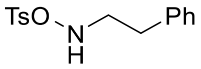

N-phenethyl-O-tosylhydroxylamine: The procedure was followed the Procedure **C** and afforded product as a white oil (58% yield). **R_f** = 0.4 (EtOAc : petroleum ether = 1 : 5). **^{1}H NMR** (400 MHz, $CDCl_3$) δ 7.84 (d, J = 8.3 Hz, 1H), 7.74 (d, J = 8.3 Hz, 3H), 7.11 (dd, J = 7.5, 5.5 Hz, 5H), 5.29 (brs, 1H), 3.17 (t, J = 6.8 Hz, 2H), 2.73 (t, J = 6.8 Hz, 2H), 2.44 (s, 3H) ppm. **^{13}C NMR** (101 MHz, $CDCl_3$) δ 144.94, 132.16, 129.42, 129.04, 128.97, 128.77, 128.69, 126.62, 126.07, 53.65, 32.70, 21.32 ppm. **HRMS** (ESI-TOF) m/z calcd for $C_{15}H_{18}NO_3S$ (M + H)$^+$: 292.1007, found 292.0999.

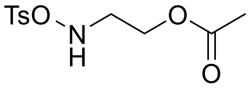

2-((tosyloxy)amino)ethyl acetate: The procedure was followed the Procedure **C** and afforded product as a white wax (59% yield). **R_f** = 0.3 (EtOAc : petroleum ether = 1 : 5). **^{1}H NMR** (400 MHz, $CDCl_3$) δ 7.85 (d, J = 8.4 Hz, 2H), 7.38 – 7.33 (m, 2H), 6.37 (brs, 1H), 4.09 (dd, J = 5.6, 4.7 Hz, 2H), 3.19 (dd, J = 5.6, 4.7 Hz, 2H), 2.46 (s, 3H), 2.02 (s, 3H) ppm. **^{13}C NMR** (101 MHz, $CDCl_3$) δ 170.62, 145.23, 131.90, 129.58, 129.11, 60.17, 51.31, 21.72, 20.76 ppm. **HRMS** (ESI-TOF) m/z calcd for $C_{11}H_{16}NO_3S$ (M + H)$^+$: 242.0851, found 242.0858.

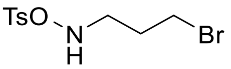

N-(3-bromopropyl)-O-tosylhydroxylamine: The procedure was followed the Procedure **C** and afforded product as a white wax (35% yield). **R_f** = 0.4 (EtOAc : petroleum ether = 1 : 5). **^{1}H NMR** (400 MHz, $CDCl_3$) δ 7.85 (d, J = 8.4 Hz, 2H), 7.36 (d, J = 8.1 Hz, 2H), 5.66 (brs, 1H), 3.26 (t, J = 6.4 Hz, 2H), 3.14 (t, J = 6.3 Hz, 2H), 2.47 (s, 3H), 1.97 - 1.88 (m, 2H) ppm. **^{13}C NMR** (101 MHz, $CDCl_3$) δ 145.30, 131.88, 129.64, 129.09, 50.58, 30.51, 29.45, 21.75 ppm. **HRMS** (ESI-TOF) m/z calcd for $C_{10}H_{15}NO_3SBr$ (M + H)$^+$: 307.9956, found 307.9965.

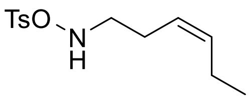

(Z)-N-(hex-3-en-1-yl)-O-tosylhydroxylamine: The procedure of was followed the Procedure **C** and afforded product as a white oil (42% yield). **R_f** = 0.4 (EtOAc : petroleum ether = 1 : 5). **^{1}H NMR** (400 MHz, $CDCl_3$) δ 7.85 (d, J = 8.3 Hz, 2H), 7.36 – 7.32 (m, 2H), 6.51 (brs, 1H), 5.25 – 5.17 (m, 2H), 2.94 (t, J = 6.8 Hz, 2H), 2.45 (s,

3H), 2.20 – 2.11 (m, 2H), 2.04 – 1.94 (m, 2H), 0.92 (t, *J* = 7.5 Hz, 3H) ppm. **^{13}C NMR** (101 MHz, $CDCl_3$) *δ* 144.83, 135.29, 132.22, 129.39, 128.99, 123.96, 27.03, 53.32, 24.32, 21.67, 14.09 ppm. **HRMS** (ESI-TOF) m/z calcd for $C_{13}H_{20}NO_3S$ $(M + H)^+$: 270.1164, found 270.1155.

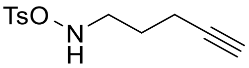

N-(pent-4-yn-1-yl)-O-tosylhydroxylamine: The procedure was followed the Procedure **C** and afforded product as a yellow oil (58% yield). **R_f** = 0.6 (EtOAc : petroleum ether = 1 : 5). **^{1}H NMR** (400 MHz, $CDCl_3$) *δ* 7.74 (d, *J* = 8.2 Hz, 2H), 7.18 (d, *J* = 8.0 Hz, 2H), 2.48 – 2.41 (m, 2H), 2.36 (s, 3H), 2.27 (dd, *J* = 6.8 Hz, 2.8, 2H), 2.05 (s, 1H), 1.26 (t, *J* = 7.1 Hz, 2H) ppm. **^{13}C NMR** (101 MHz, $CDCl_3$) *δ* 129.51, 129.04, 128.99, 126.05, 83.07, 69.10, 51.24, 24.74, 21.35, 15.57 ppm. **HRMS** (ESI-TOF) m/z calcd for $C_{12}H_{16}NO_3S$ $(M + H)^+$: 254.0851, found 254.0854.

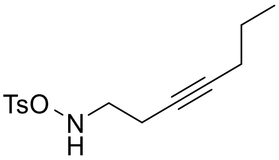

N-(hept-3-yn-1-yl)-O-tosylhydroxylamine: The procedure was followed the Procedure **C** and afforded product as a yellow oil (49% yield). **R_f** = 0.6 (EtOAc : petroleum ether = 1 : 5). **^{1}H NMR** (400 MHz, $CDCl_3$) *δ* 7.86 (d, *J* = 8.4 Hz, 2H), 7.35 (d, *J* = 8.1 Hz, 2H), 6.55 (brs, 1H), 3.00 (t, *J* = 6.2 Hz, 2H), 2.46 (s, 3H), 2.38 – 2.32 (m, 2H), 2.12 – 2.05 (m, 2H), 1.52 – 1.41 (m, 2H), 0.93 (t, *J* = 7.4 Hz, 3H) ppm. **^{13}C NMR** (101 MHz, $CDCl_3$) *δ* 144.96, 132.17, 129.48, 129.01, 83.01, 75.77, 51.44, 22.23, 21.66, 20.60, 17.09, 13.42 ppm. **HRMS** (ESI-TOF) m/z calcd for $C_{14}H_{20}NO_3S$ $(M + H)^+$: 282.1164, found 282.1161.

TsO−NH_2

O-tosylhydroxylamine: The procedure was followed the Procedure **C** and afforded product as a white wax (45% yield). **^{1}H NMR** (400 MHz, $CDCl_3$) *δ* 7.87 (d, *J* = 8.3 Hz, 2H), 7.37 (d, *J* = 8.1 Hz, 2H), 5.11 (brs, 2H), 2.46 (s, 3H) ppm. **^{13}C NMR** (101 MHz, $CDCl_3$) *δ* 145.37, 131.54, 129.66, 129.16, 21.70 ppm. **HRMS** (ESI-TOF) m/z calcd for $C_7H_{10}NO_3S$ $(M + H)^+$: 188.0381, found 188.0374.

4.3 Synthesis of deuterium substrate 50-D_2 and 49-D:

The deuterium substrate **50-D_2** was prepared according to the reported literature.[1,7,8]

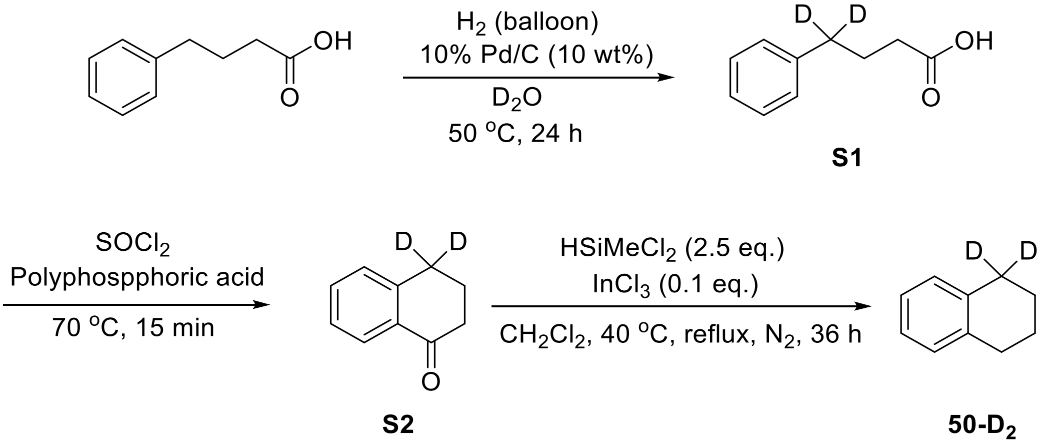

Supplementary Fig 8. Synthesis of deuterium substrate **50-D_2**

A 50 mL Schlenk flask was charged with 4-phenylbutanoic acid (0.82 g, 5 mmol, 1.0 equiv) and 10 % Pd/C (0.16 g, 10 wt%) with 10 mL D_2O. The reaction flask was fitted with a balloon full of H_2 and purged with H_2 three times. The resulted black suspension was heated at 50 ℃. After 24 h, the reaction mixture was filtered through Celite to remove Pd/C and washed with petroleum ether. The aqueous phase was extracted three times with petroleum ether. The combined organic phases were washed with brine, dried over $NaSO_4$, filtered, and concentrated. The product **S1** was used without further purification.

Then the acid chloride obtained from 4-phenylbutyric acid **S1** and thionyl chloride (10 mL) was treated with 15 g polyphosphoric acid with exclusion of moisture, and the whole was stirred at 70 ℃ for 15 min. The mixture was poured into water and extracted with ether. The combined organic phases were washed with brine, dried over $NaSO_4$, filtered, and concentrated. The product **S2** was used without further purification.

At last, the solution of Me_2SiClH (12.5 mmol) in CH_2Cl_2 (5 mL) was added to a solution of $InCl_3$ (0.5 mmol) and aryl ketone (5 mmol) in CH_2Cl_2 at room temperature under N_2 atmosphere. Subsequently, it was heated to 40 °C and subjected to reflux reaction for 36 h. After the completion of the reaction, the mixture was washed with water (2 × 50 ml) and extracted with ether (50 mL). After dried over $MgSO_4$, filtered and concentrated by rotary evaporation. The residue was purified by silica gel chromatography (petroleum ether) to afford the product **50-D_2** (391.6 mg, 58% yield, 95% D). The data match the reported literature.[9]

^{1}H NMR (400 MHz, Chloroform-d) δ 7.11 – 7.00 (m, 4H), 2.80 – 2.72 (m, 2H), 1.77 (d, J = 3.6 Hz, 4H) ppm. **HRMS** (ESI-TOF) m/z calcd for $D_2C_{10}H_{11}$ $(M + H)^+$: 135.1143, found 135.1142.

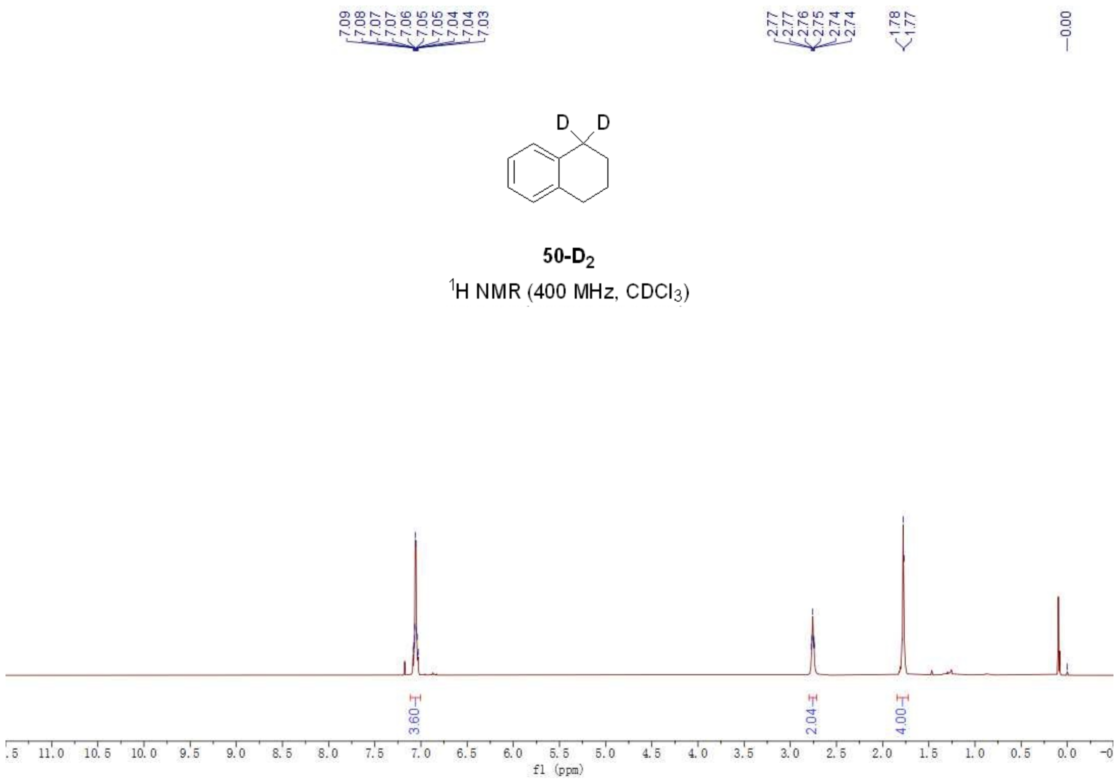

Supplementary Fig 9. The ^{1}H NMR spectrum of **50-D$_2$**

The deuterium substrate **49-D** was prepared according to the reported literature.[7]

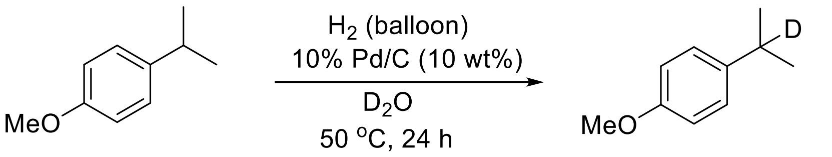

49 **49-D**

Supplementary Fig 10. Synthesis of deuterium substrate **49-D**

A 50 mL Schlenk flask was charged with 4-Isopropylanisole **49** (1.50 g, 10 mmol, 1.0 equiv) and 10 % Pd/C (0.30 g, 10 wt%) with 10 mL D_2O. The reaction flask was fitted with a balloon full of H_2 and purged with H_2 three times. The resulted black suspension was heated at 50 ℃. After 24 h, the reaction mixture was filtered through Celite to remove Pd/C and washed with petroleum ether. The aqueous phase was extracted three times with petroleum ether. The combined organic phases were washed with brine, dried over $NaSO_4$, filtered, and concentrated. The residue was purified by silica gel chromatography (petroleum ether: EtOAc = 10: 1) to afford the product **49-D** (1.40 g, 93% yield, 95% D). The data match the reported literature.[10]

^{1}H NMR (400 MHz, Chloroform-d) δ 7.14 (d, J = 8.7, 2H), 6.84 (d, J = 8.7, 2H), 3.78 (s, 3H), 2.87 (d, J = 6.9, 0H), 1.21 (t, J = 1.0, 6H). ppm. **HRMS** (ESI-TOF) m/z calcd for $DC_{10}H_{14}O$ $(M + H)^+$: 152.1186, found 152.1184.

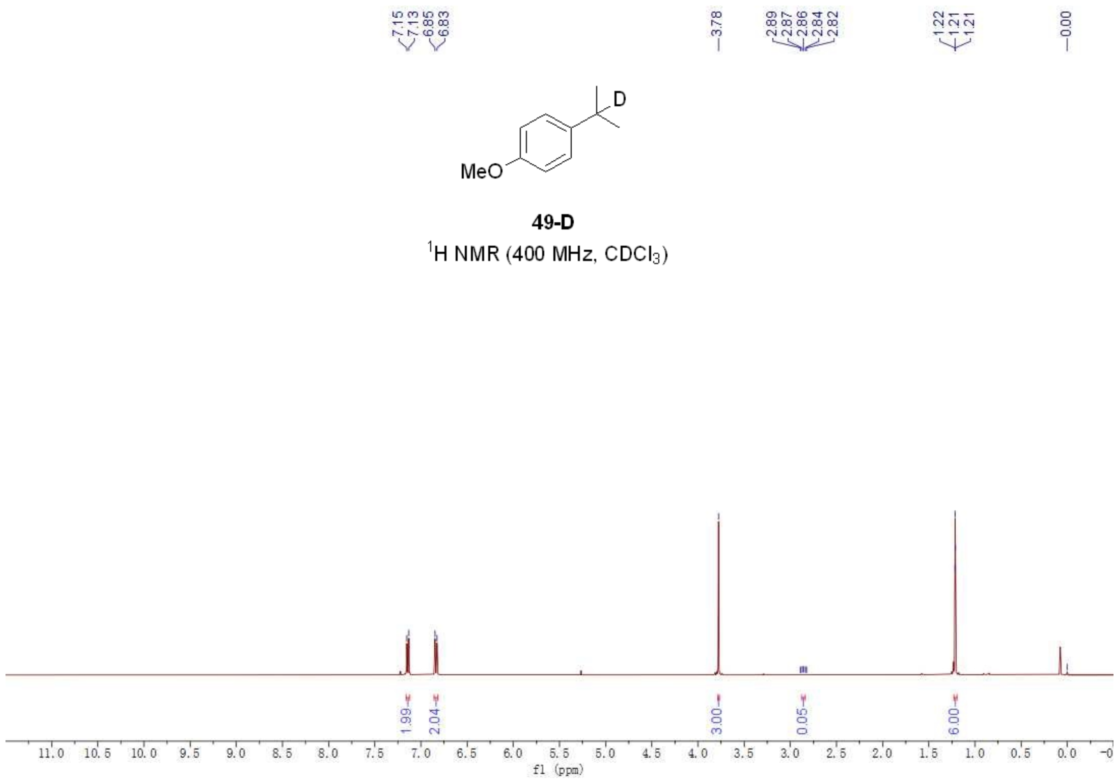

Supplementary Fig 11. The ^{1}H NMR spectrum of **49-D**

4.4 Synthesis of ibuprofen methyl ester, dehydroabietic acid methyl ester and estrone 3-methyl ether

Ibuprofen methyl ester **S3**, dehydroabietic acid methyl ester **S4** and Estrone 3-methyl ether **S5** are known compounds, and they were prepared according to the corresponding literature reports.[11,12,13] Analytical data (^{1}H NMR, ^{13}C NMR) matches with the literature. [11,12,13] The detail structure of these compounds, see below.

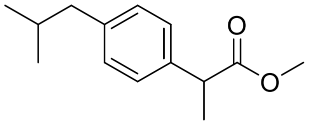

S3

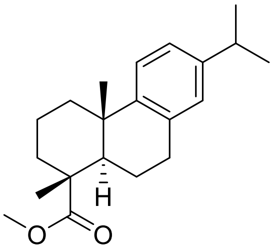

S4

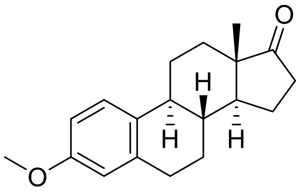

S5

Supplementary Fig 12. The structure of compound **S3**, **S4** and **S5**

General Procedure for synthetizing S3 and S4

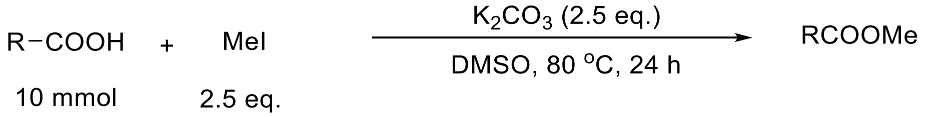

Supplementary Fig 13. Synthesis of ibuprofen methyl ester **S3** and dehydroabietic acid methyl ester **S4**

MeI (25.0 mmol) was added to a solution of Ibuprofen or Dehydroabietic acid (10.0 mmol) and K_2CO_3 (25.0 mmol) in DMSO (20 mL) at room temperature. Subsequently, it was heated to 80 ℃ and reacted for 24 h. After the completion of the reaction, the mixture was washed with water (2 × 50 ml) and extracted with ether (50 mL). After dried over $MgSO_4$, filtered and concentrated by rotary evaporation. The residue was purified by silica gel chromatography (EtOAc/petroleum ether= 1:1) to afford the product **S3** (95%) or **S4** (92%). Ibuprofen methyl ester[11] and dehydroabietic acid methyl ester[12] are literature reported compounds.

General Procedure for synthetizing S5

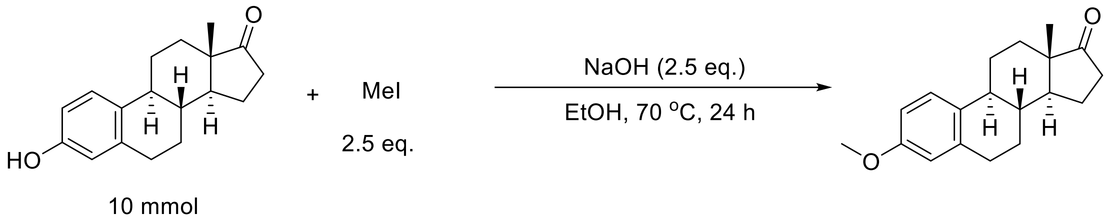

Supplementary Fig 14. Synthesis of estrone 3-methyl ether **S5**

MeI (25.0 mmol) was added to a solution of estrone (10.0 mmol) and NaOH (25.0 mmol) in EtOH (20 mL) at room temperature. Subsequently, it was heated to 70 ℃ and reacted for 24 h. After the completion of the reaction, the mixture was washed with water (2 × 50 ml) and extracted with ether (50 mL). After dried over $MgSO_4$, filtered and concentrated by rotary evaporation. The residue was purified by silica gel chromatography (EtOAc/petroleum ether= 1:1) to afford the product **S5** (65%). Estrone 3-methyl ether[13] is literature reported compound.

Analytical data for synthetized known compounds:

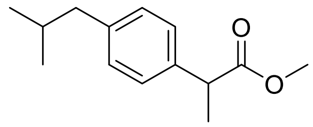

Ibuprofen methyl ester: The procedure was followed the reported literature and afforded product as a colorless oil (95% yield). $\mathbf{R_f}$ = 0.5 (EtOAc : petroleum ether = 1 : 5). **^{1}H NMR** (400 MHz, $CDCl_3$) δ 7.19 (d, J = 8.1 Hz, 2H), 7.09 (d, J = 8.2 Hz, 2H), 3.69 (q, J = 7.2 Hz, 1H), 3.64 (s, 3H), 2.44 (d, J = 7.2 Hz, 2H), 1.91 – 1.78 (m, 1H), 1.48 (d, J = 7.2 Hz, 3H), 0.89 (d, J = 6.6 Hz, 6H) ppm. **HRMS** (ESI-TOF) m/z calcd for $C_{14}H_{21}NO_2$ $(M + H)^+$: 221.1542, found 221.1540.

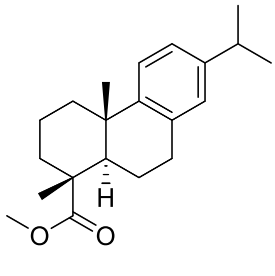

Dehydroabietic acid methyl ester: The procedure was followed the procedure and afforded product as a colorless oil (92% yield). $\mathbf{R_f}$ = 0.3 (EtOAc : petroleum ether = 1 : 1). **^{1}H NMR** (400 MHz, $CDCl_3$) δ 7.20 (d, J = 8.2 Hz, 1H), 7.04 (dd, J = 8.2, 2.1 Hz, 1H), 6.92 (d, J = 2.0 Hz, 1H), 3.70 (s, 3H), 2.95 – 2.89 (m, 2H), 2.88 – 2.83 (m, 1H), 2.36 – 2.31 (m, 1H), 2.28 (dd, J = 12.5, 2.2 Hz, 1H), 1.90 – 1.85 (m, 1H), 1.82 (td, J = 5.0, 1.8 Hz, 2H), 1.78 – 1.73 (m, 2H), 1.69 – 1.67 (m, 1H), 1.55 (dd, J = 12.6, 4.4 Hz, 1H), 1.47 – 1.43 (m, 1H), 1.32 (s, 3H), 1.27 (s, 3H), 1.25 (d, J = 2.9 Hz, 6H) ppm.

HRMS (ESI-TOF) m/z calcd for $C_{21}H_{31}O_2$ $(M + H)^+$: 315.2324, found 315.2320.

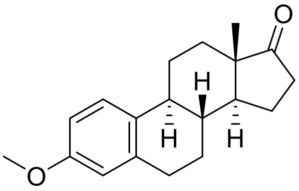

Estrone 3-methyl ether: The procedure of was followed the procedure and afforded product as a white solid (65% yield). $\mathbf{R_f}$ = 0.5 (EtOAc : petroleum ether = 2 : 1). **^{1}H NMR** (400 MHz, $CDCl_3$) δ 7.23 – 7.18 (m, 1H), 6.74 – 6.70 (m, 1H), 6.65 (d, J = 2.8 Hz, 1H), 3.78 (s, 3H), 2.94 – 2.85 (m, 2H), 2.54 – 2.46 (m, 1H), 2.44 – 2.35 (m, 1H), 2.30 – 2.21 (m, 1H), 2.21 – 1.92 (m, 4H), 1.66 – 1.42 (m, 6H), 0.91 (s, 3H) ppm. **HRMS** (ESI-TOF) m/z calcd for $C_{19}H_{25}O_2$ $(M + H)^+$: 285.1855, found 285.1849.

5. Analytical data for compounds

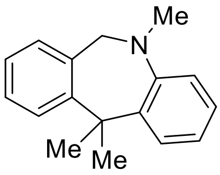

3

5,11,11-trimethyl-6,11-dihydro-5H-dibenzo[b,e]azepine 3: The procedure **A** was followed using 9,9-dimethyl-9,10-dihydroanthracene (62.5 mg, 0.30 mmol, 1.0 equiv.) and N-methyl-O-tosylhydroxylamine (90.0 mg, 0.45 mmol, 1.5 equiv.). Purified by silica gel chromatography (EtOAc : petroleum ether = 1 : 50) and afforded product **3** as a white wax (69.8 mg, 98% yield). $\mathbf{R_f}$ = 0.6 (EtOAc : petroleum ether = 1 : 20). **^{1}H NMR (400 MHz, Chloroform-d)** δ 7.41 (dd, J = 7.6, 1.7 Hz, 1H), 7.31 (dd, J = 7.9, 1.6 Hz, 1H), 7.19 – 7.14 (m, 1H), 7.12 – 7.02 (m, 2H), 6.98 – 6.91 (m, 2H), 6.89 – 6.80 (m, 1H), 4.34 (s, 2H), 2.91 (s, 3H), 1.92 (s, 6H) ppm. **^{13}C NMR (101 MHz, Chloroform-d)** δ 149.27, 145.43, 141.66, 136.18, 129.02, 127.34, 126.45, 126.16, 125.21, 124.42, 121.07, 118.40, 60.78, 41.81, 41.08, 28.81 ppm. **HRMS** (ESI-TOF) m/z calcd for $C_{17}H_{20}N$ (M + H)$^+$: 238.1596, found 238.1603.

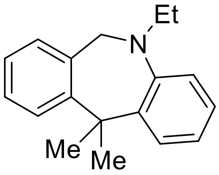

4

5-ethyl-11,11-dimethyl-6,11-dihydro-5H-dibenzo[b,e]azepine 4: The procedure **A** was followed using 9,9-dimethyl-9,10-dihydroanthracene (62.5 mg, 0.30 mmol, 1.0 equiv.) and N-ethyl-O-tosylhydroxylamine (96.9 mg, 0.45 mmol, 1.5 equiv.). Purified by silica gel chromatography (EtOAc : petroleum ether = 1 : 50) and afforded product **4** as a light yellow oil (63.9 mg, 85% yield). $\mathbf{R_f}$ = 0.6 (EtOAc : petroleum ether = 1 : 20). **^{1}H NMR (400 MHz, Chloroform-d)** δ 7.47 – 7.40 (m, 1H), 7.38 – 7.31 (m, 1H), 7.20 – 7.14 (m, 1H), 7.14 – 7.05 (m, 2H), 7.03 – 6.94 (m, 2H), 6.93 – 6.84 (m, 1H), 4.34 (s, 2H), 3.27 (q, J = 7.0 Hz, 2H), 1.95 (s, 6H), 1.26 (t, J = 7.1 Hz, 3H) ppm. **^{13}C NMR (101 MHz, Chloroform-d)** δ 149.02, 145.54, 142.59, 136.88, 128.94, 127.24, 126.31, 126.06, 125.50, 124.66, 121.53, 119.47, 58.52, 47.80, 41.33, 29.65, 12.96 ppm. **HRMS** (ESI-TOF) m/z calcd for $C_{18}H_{22}N$ (M + H)$^+$: 252.1752, found 252.1762.

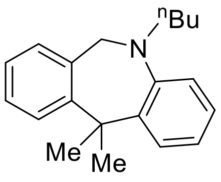

5

5-butyl-11,11-dimethyl-6,11-dihydro-5H-dibenzo[b,e]azepine 5: The procedure **A** was followed using 9,9-dimethyl-9,10-dihydroanthracene (62.5 mg, 0.30 mmol, 1.0 equiv.) and N-butyl-O-tosylhydroxylamine (109.5 mg, 0.45 mmol, 1.5 equiv.). Purified by silica gel chromatography (EtOAc : petroleum ether = 1 : 50) and afforded product **5** as a light yellow oil (69.6 mg, 83% yield). $\mathbf{R_f}$ = 0.6 (EtOAc : petroleum ether = 1 : 20). **^{1}H NMR (400 MHz, Chloroform-d)** *δ* 7.46 – 7.41 (m, 1H), 7.36 – 7.32 (m, 1H), 7.18 – 7.13 (m, 1H), 7.13 – 7.04 (m, 2H), 7.03 – 6.99 (m, 1H), 6.98 – 6.93 (m, 1H), 6.91 – 6.84 (m, 1H), 4.33 (s, 2H), 3.22 – 3.14 (m, 2H), 1.95 (s, 6H), 1.72 – 1.63 (m, 2H), 1.45 – 1.35 (m, 2H), 0.94 (t, *J* = 7.3 Hz, 3H) ppm.. **^{13}C NMR (101 MHz, Chloroform-d)** *δ* 149.31, 145.56, 142.43, 136.81, 128.96, 127.22, 126.32, 126.06, 125.52, 124.60, 121.46, 119.43, 59.05, 53.82, 41.35, 29.68, 29.63, 20.82, 14.03 ppm. **HRMS** (ESI-TOF) m/z calcd for $C_{20}H_{26}N$ (M + H)$^+$: 280.2065, found 280.2071.

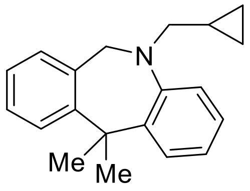

6

5-(cyclopropylmethyl)-11,11-dimethyl-6,11-dihydro-5H-dibenzo[b,e]azepine 6: The procedure **A** was followed using 9,9-dimethyl-9,10-dihydroanthracene (62.5 mg, 0.30 mmol, 1.0 equiv.) and N-(cyclopropylmethyl)-O-tosylhydroxylamine (114.9 mg, 0.45 mmol, 1.5 equiv.). Purified by silica gel chromatography (EtOAc : petroleum ether = 1 : 50) and afforded product **6** as a light yellow oil (67.6 mg, 81% yield). $\mathbf{R_f}$ = 0.6 (EtOAc : petroleum ether = 1 : 20). **^{1}H NMR (400 MHz, Chloroform-d)** δ 7.47 – 7.41 (m, 1H), 7.36 – 7.30 (m, 1H), 7.19 – 7.05 (m, 3H), 7.03 – 6.96 (m, 2H), 6.91 – 6.84 (m, 1H), 4.48 (s, 2H), 3.07 (d, *J* = 6.5 Hz, 2H), 1.97 (s, 6H), 1.17 – 1.08 (m, 1H), 0.62 – 0.55 (m, 2H), 0.30 – 0.23 (m, 2H) ppm. **^{13}C NMR (101 MHz, Chloroform-d)** δ 149.70, 145.65, 142.42, 136.82, 129.11, 127.28, 126.31, 126.09, 125.32, 124.55, 121.41, 119.61, 59.20, 58.02, 41.27, 29.36, 9.48, 4.36 ppm. **HRMS** (ESI-TOF) m/z calcd for $C_{20}H_{24}N$ (M + H)$^+$: 278.1909, found 278.1900.

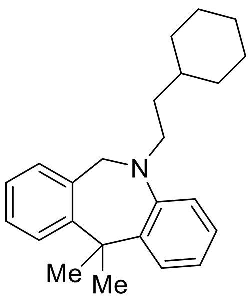

7

5-(2-cyclohexylethyl)-11,11-dimethyl-6,11-dihydro-5H-dibenzo[b,e]azepine 7: The procedure **A** was followed using 9,9-dimethyl-9,10-dihydroanthracene (62.5 mg, 0.30 mmol, 1.0 equiv.) and N-(2-cyclohexylethyl)-O-tosylhydroxylamine (133.8 mg, 0.45 mmol, 1.5 equiv.). Purified by silica gel chromatography (EtOAc : petroleum ether = 1 : 50) and afforded product **7** as a light yellow oil (98.0 mg, 98% yield). $\mathbf{R_f}$ = 0.6 (EtOAc : petroleum ether = 1 : 20). **^{1}H NMR (400 MHz, Chloroform-d)** δ 7.45 – 7.41 (m, 1H), 7.36 – 7.31 (m, 1H), 7.19 – 7.13 (m, 1H), 7.13 – 7.04 (m, 2H), 7.04 – 6.99 (m, 1H), 6.99 – 6.93 (m, 1H), 6.91 – 6.84 (m, 1H), 4.32 (s, 2H), 3.25 – 3.15 (m, 2H), 1.95 (s, 6H), 1.78 – 1.66 (m, 4H), 1.58 (q, J = 7.1 Hz, 2H), 1.40 – 1.31 (m, 1H), 1.31 – 0.97 (m, 4H), 0.97 – 0.76 (m, 2H) ppm. **^{13}C NMR (101 MHz, Chloroform-d)** δ 149.37, 145.52, 142.48, 136.86, 128.94, 127.23, 126.29, 126.05, 125.52, 124.61, 121.49, 119.41, 59.08, 51.72, 41.35, 35.99, 34.89, 33.38, 29.65, 26.61, 26.32 ppm. **HRMS** (ESI-TOF) m/z calcd for $C_{24}H_{32}N$ $(M + H)^+$: 334.2535, found 334.2528.

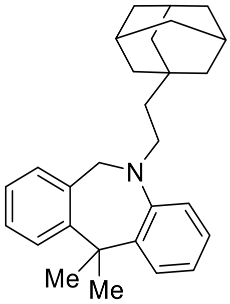

8

5-(2-(adamantan-1-yl)ethyl)-11,11-dimethyl-6,11-dihydro-5H-dibenzo[b,e]azepine 8: The procedure **A** was followed using 9,9-dimethyl-9,10-dihydroanthracene (62.5 mg, 0.30 mmol, 1.0 equiv.) and N-(2-(adamantan-1-yl)ethyl)-O-tosylhydroxylamine (157.3 mg, 0.45 mmol, 1.5 equiv.). Purified by silica gel chromatography (EtOAc : petroleum ether = 1 : 50) and afforded product **8** as a light yellow oil (100.4 mg, 87% yield). $\mathbf{R_f}$ = 0.5 (EtOAc : petroleum ether = 1 : 20). **^{1}H NMR (400 MHz, Chloroform-d)** δ 7.46 – 7.41 (m, 1H), 7.36 – 7.31 (m, 1H), 7.20 – 7.15 (m, 1H), 7.13 – 7.04 (m, 2H), 7.03 – 6.99 (m, 1H), 6.98 – 6.93 (m, 1H), 6.91 – 6.85 (m, 1H), 4.32 (s, 2H), 3.25 – 3.17 (m, 2H), 2.00 – 1.95 (m, 3H), 1.94 (s, 6H), 1.74 – 1.62 (m, 6H), 1.56 (d, J = 2.9 Hz, 6H), 1.49 – 1.43 (m, 2H) ppm. **^{13}C NMR (101 MHz, Chloroform-d)** δ 149.37, 145.50, 142.54, 136.99, 128.88, 127.23, 126.29, 126.03, 125.54, 124.66, 121.48, 119.55, 59.34, 48.15, 42.48, 41.44, 41.38, 37.19, 31.95, 29.78, 28.67 ppm.

HRMS (ESI-TOF) m/z calcd for $C_{28}H_{36}N$ (M + H)$^+$: 386.2848, found 386.2856.

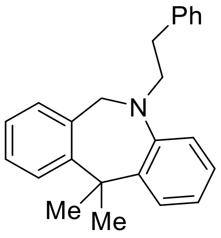

9

11,11-dimethyl-5-phenethyl-6,11-dihydro-5H-dibenzo[b,e]azepine 9: The procedure **A** was followed using 9,9-dimethyl-9,10-dihydroanthracene (62.5 mg, 0.30 mmol, 1.0 equiv.) and N-phenethyl-O-tosylhydroxylamine (131.1 mg, 0.45 mmol, 1.5 equiv.). Purified by silica gel chromatography (EtOAc : petroleum ether = 1 : 50) and afforded product **9** as a light yellow oil (74.3 mg, 71% yield). $\mathbf{R_f}$ = 0.5 (EtOAc : petroleum ether = 1 : 20). **^{1}H NMR (400 MHz, Chloroform-d)** δ 7.44 – 7.39 (m, 1H), 7.37 – 7.32 (m, 1H), 7.30 – 7.24 (m, 2H), 7.24 – 7.14 (m, 4H), 7.13 – 7.02 (m, 3H), 6.99 – 6.94 (m, 1H), 6.92 – 6.85 (m, 1H), 4.40 (s, 2H), 3.50 – 3.42 (m, 2H), 3.02 – 2.95 (m, 2H), 1.89 (s, 6H) ppm. **^{13}C NMR (101 MHz, Chloroform-d)** δ 148.78, 145.60, 142.50, 139.99, 136.50, 128.98, 128.71, 128.44, 127.32, 126.44, 126.14, 126.11, 125.63, 124.65, 121.70, 119.53, 59.02, 55.80, 41.33, 34.15, 29.56 ppm. **HRMS** (ESI-TOF) m/z calcd for $C_{24}H_{26}N$ (M + H)$^+$: 328.2065, found 328.2069.

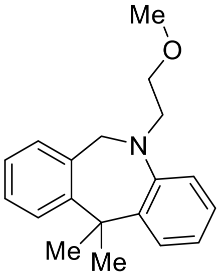

10

5-(2-methoxyethyl)-11,11-dimethyl-6,11-dihydro-5H-dibenzo[b,e]azepine 10: The procedure **A** was followed using 9,9-dimethyl-9,10-dihydroanthracene (62.5 mg, 0.30 mmol, 1.0 equiv.) and N-(2-methoxyethyl)-O-tosylhydroxylamine (110.4 mg, 0.45 mmol, 1.5 equiv.). Purified by silica gel chromatography (EtOAc : petroleum ether = 1 : 20) and afforded product **10** as a colorless oil (76.6 mg, 91% yield). $\mathbf{R_f}$ = 0.4 (EtOAc : petroleum ether = 1 : 10). **^{1}H NMR (400 MHz, Chloroform-d)** δ 7.46 – 7.41 (m, 1H), 7.37 – 7.31 (m, 1H), 7.20 – 7.14 (m, 1H), 7.14 – 7.02 (m, 3H), 7.00 – 6.95 (m, 1H), 6.92 – 6.85 (m, 1H), 4.43 (s, 2H), 3.67 (t, *J* = 6.1 Hz, 2H), 3.45 (t, *J* = 6.1 Hz, 2H), 3.36 (s, 3H), 1.94 (s, 6H) ppm. **^{13}C NMR (101 MHz, Chloroform-d)** δ 149.01, 145.60, 142.41, 136.51, 129.00, 127.36, 126.46, 126.11, 125.63, 124.65, 121.71, 119.67, 70.60, 59.06, 58.78, 53.39, 41.33, 29.57 ppm. **HRMS** (ESI-TOF) m/z calcd for $C_{19}H_{24}NO$ (M + H)$^+$: 282.1858, found 282.1866.

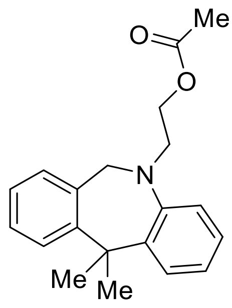

11

2-(11,11-dimethyl-6,11-dihydro-5H-dibenzo[b,e]azepin-5-yl)ethyl acetate 11: The procedure **A** was followed using 9,9-dimethyl-9,10-dihydroanthracene (62.5 mg, 0.30 mmol, 1.0 equiv.) and 2-((tosyloxy)amino)ethyl acetate (123.0 mg, 0.45 mmol, 1.5 equiv.). Purified by silica gel chromatography (EtOAc : petroleum ether = 1 : 10) and afforded product **11** as a colorless oil (66.6 mg, 72% yield). $\mathbf{R_f}$ = 0.3 (EtOAc : petroleum ether = 1 : 10). **^{1}H NMR (400 MHz, Chloroform-d)** δ 7.47 – 7.41 (m, 1H), 7.38 – 7.32 (m, 1H), 7.20 – 7.15 (m, 1H), 7.14 – 7.05 (m, 2H), 7.03 – 6.99 (m, 1H), 6.98 – 6.94 (m, 1H), 6.93 – 6.88 (m, 1H), 4.38 (s, 2H), 4.33 (t, J = 5.9 Hz, 2H), 3.49 (t, J = 5.9 Hz, 2H), 2.00 (s, 3H), 1.94 (s, 6H) ppm. **^{13}C NMR (101 MHz, Chloroform-d)** δ 170.97, 148.48, 145.42, 142.75, 136.14, 128.92, 127.40, 126.51, 126.13, 125.76, 124.70, 122.16, 119.49, 61.49, 58.81, 52.26, 41.31, 29.58, 20.87 ppm. **HRMS** (ESI-TOF) m/z calcd for $C_{20}H_{24}NO_2$ $(M + H)^+$: 310.1807, found 310.1812.

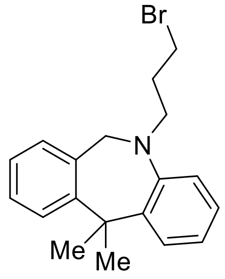

12

5-(3-bromopropyl)-11,11-dimethyl-6,11-dihydro-5H-dibenzo[b,e]azepine 12: The procedure **A** was followed using 9,9-dimethyl-9,10-dihydroanthracene (62.5 mg, 0.30 mmol, 1.0 equiv.) and N-(3-bromopropyl)-O-tosylhydroxylamine (138.7 mg, 0.45 mmol, 1.5 equiv.). Purified by silica gel chromatography (EtOAc : petroleum ether = 1 : 50) and afforded product **12** as a light yellow oil (95.3 mg, 92% yield). $\mathbf{R_f}$ = 0.6 (EtOAc : petroleum ether = 1 : 20). **^{1}H NMR (400 MHz, Chloroform-d)** δ 7.45 – 7.41 (m, 1H), 7.38 – 7.33 (m, 1H), 7.20 – 7.15 (m, 1H), 7.14 – 7.05 (m, 2H), 7.02 – 6.99 (m, 1H), 6.97 – 6.93 (m, 1H), 6.93 – 6.88 (m, 1H), 4.34 (s, 2H), 3.46 (t, J = 6.5 Hz, 2H), 3.35 (t, J = 6.8 Hz, 2H), 2.21 (p, J = 6.6 Hz, 2H), 1.94 (s, 6H) ppm. **^{13}C NMR (101 MHz, Chloroform-d)** δ 148.12, 145.30, 142.74, 136.31, 128.90, 127.29, 126.43, 126.14, 125.77, 124.64, 122.02, 119.33, 59.69, 51.82, 41.34, 31.99, 30.28, 29.69 ppm. **HRMS** (ESI-TOF) m/z calcd for $C_{19}H_{23}NBr$ $(M + H)^+$: 344.1014, found

344.1013.

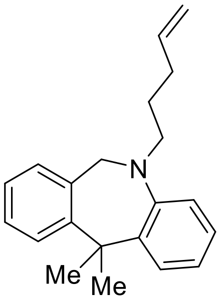

13

11,11-dimethyl-5-(pent-4-en-1-yl)-6,11-dihydro-5H-dibenzo[b,e]azepine 13: The procedure **A** was followed using 9,9-dimethyl-9,10-dihydroanthracene (62.5 mg, 0.30 mmol, 1.0 equiv.) and N-(pent-4-en-1-yl)-O-tosylhydroxylamine (114.9 mg, 0.45 mmol, 1.5 equiv.). Purified by silica gel chromatography (EtOAc : petroleum ether = 1 : 50) and afforded product **13** as a light yellow oil (52.5 mg, 60% yield). $\mathbf{R_f}$ = 0.6 (EtOAc : petroleum ether = 1 : 20). **^{1}H NMR (400 MHz, Chloroform-d)** δ 7.45 – 7.42 (m, 1H), 7.37 – 7.32 (m, 1H), 7.20 – 7.15 (m, 1H), 7.14 – 7.06 (m, 2H), 7.03 – 6.94 (m, 2H), 6.92 – 6.85 (m, 1H), 5.90 – 5.77 (m, 1H), 5.07 – 4.94 (m, 2H), 4.34 (s, 2H), 3.26 – 3.15 (m, 2H), 2.19 – 2.11 (m, 2H), 1.95 (s, 6H), 1.83 – 1.78 (m, 2H) ppm. **^{13}C NMR (101 MHz, Chloroform-d)** δ 149.10, 145.55, 142.51, 138.33, 136.71, 128.97, 127.26, 126.36, 126.09, 125.57, 124.62, 121.57, 119.44, 114.87, 59.16, 53.46, 41.36, 31.76, 29.67, 26.69 ppm. **HRMS** (ESI-TOF) m/z calcd for $C_{21}H_{26}N$ $(M + H)^+$: 292.2065, found 292.2074.

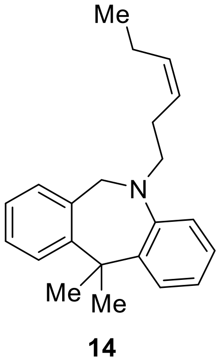

14

5-(hex-3-en-1-yl)-11,11-dimethyl-6,11-dihydro-5H-dibenzo[b,e]azepine 14: The procedure **A** was followed using 9,9-dimethyl-9,10-dihydroanthracene (62.5 mg, 0.30 mmol, 1.0 equiv.) and (Z)-N-(hex-3-en-1-yl)-O-tosylhydroxylamine (121.2 mg, 0.45 mmol, 1.5 equiv.). Purified by silica gel chromatography (EtOAc : petroleum ether = 1 : 50) and afforded product **14** as a yellow oil (87.4 mg, 96% yield). $\mathbf{R_f}$ = 0.6 (EtOAc : petroleum ether = 1 : 20). **^{1}H NMR (400 MHz, Chloroform-d)** δ 7.46 – 7.39 (m, 1H), 7.37 – 7.30 (m, 1H), 7.19 – 7.14 (m, 1H), 7.14 – 7.04 (m, 2H), 7.04 – 6.99 (m, 1H), 6.99 – 6.93 (m, 1H), 6.91 – 6.84 (m, 1H), 5.59 – 5.37 (m, 2H), 4.35 (d, *J* = 5.4 Hz, 2H), 3.23 (q, *J* = 6.8 Hz, 2H), 2.48 – 2.34 (m, 2H), 2.07 – 1.97 (m, 2H), 1.94 (d, *J* = 3.2 Hz, 6H), 0.99 – 0.90 (m, 3H) ppm. **^{13}C NMR (101 MHz,**

Chloroform-d) δ 149.08, 145.64, 142.38, 136.60, 133.66, 129.01, 127.25, 126.64, 126.36, 126.09, 125.48, 124.56, 121.44, 58.80, 54.17, 41.31, 30.78, 29.54, 25.61, 13.69 ppm. **HRMS** (ESI-TOF) m/z calcd for $C_{22}H_{28}N$ $(M + H)^+$: 306.2222, found 306.2229.

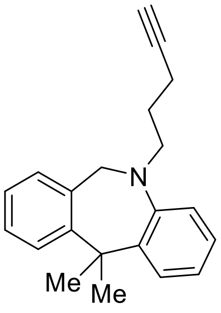

15

11,11-dimethyl-5-(pent-4-yn-1-yl)-6,11-dihydro-5H-dibenzo[b,e]azepine 15: The procedure **A** was followed using 9,9-dimethyl-9,10-dihydroanthracene (62.5 mg, 0.30 mmol, 1.0 equiv.) and N-(pent-4-yn-1-yl)-O-tosylhydroxylamine (114.0 mg, 0.45 mmol, 1.5 equiv.). Purified by silica gel chromatography (EtOAc : petroleum ether = 1 : 50) and afforded product **15** as a light yellow oil (73.0 mg, 84% yield). $\mathbf{R_f}$ = 0.6 (EtOAc : petroleum ether = 1 : 20). **^{1}H NMR (400 MHz, Chloroform-d)** δ 7.44 (dd, *J* = 7.6, 1.7 Hz, 1H), 7.35 (dd, *J* = 8.0, 1.6 Hz, 1H), 7.21 – 7.15 (m, 1H), 7.15 – 7.05 (m, 2H), 7.04 – 7.01 (m, 1H), 6.99 – 6.94 (m, 1H), 6.92 – 6.87 (m, 1H), 4.35 (s, 2H), 3.32 (dd, *J* = 8.0, 6.3, 2H), 2.32 – 2.24 (m, 2H), 1.98 – 1.88 (m, 9H) ppm. **^{13}C NMR (101 MHz, Chloroform-d)** δ 148.64, 145.46, 142.64, 136.53, 128.95, 127.28, 126.40, 126.12, 125.69, 124.65, 121.80, 119.42, 83.94, 68.74, 59.43, 52.62, 41.37, 29.69, 26.18, 16.61 ppm. **HRMS** (ESI-TOF) m/z calcd for $C_{21}H_{24}N$ $(M + H)^+$: 290.1909, found 290.1918.

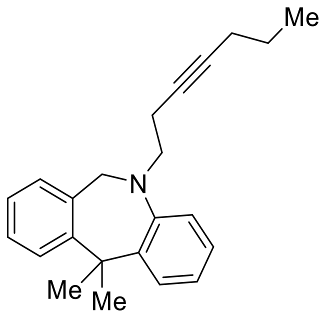

16

5-(hept-3-yn-1-yl)-11,11-dimethyl-6,11-dihydro-5H-dibenzo[b,e]azepine 16: The procedure **A** was followed using 9,9-dimethyl-9,10-dihydroanthracene (62.5 mg, 0.30 mmol, 1.0 equiv.) and N-(hept-3-yn-1-yl)-O-tosylhydroxylamine (126.6 mg, 0.45 mmol, 1.5 equiv.). Purified by silica gel chromatography (EtOAc : petroleum ether = 1 : 50) and afforded product **16** as a yellow oil (77.8 mg, 82% yield). $\mathbf{R_f}$ = 0.6 (EtOAc : petroleum ether = 1 : 20). **^{1}H NMR (400 MHz, Chloroform-d)** δ 7.45 – 7.41 (m, 1H), 7.35 – 7.32 (m, 1H), 7.18 – 7.14 (m, 1H), 7.14 – 7.05 (m, 2H), 7.03 –

6.99 (m, 1H), 6.98 – 6.94 (m, 1H), 6.91 – 6.86 (m, 1H), 4.38 (s, 2H), 3.41 (t, *J* = 7.4 Hz, 2H), 2.57 – 2.50 (m, 2H), 2.14 – 2.08 (m, 2H), 1.94 (s, 6H), 1.49 (q, *J* = 7.2 Hz, 2H), 0.96 (t, *J* = 7.4 Hz, 3H) ppm. **^{13}C NMR (101 MHz, Chloroform-d)** δ 148.36, 145.59, 142.44, 136.34, 128.99, 127.33, 126.47, 126.12, 125.62, 124.62, 121.72, 119.44, 81.48, 58.77, 53.54, 41.29, 29.60, 22.36, 20.78, 17.91, 13.51 ppm. **HRMS** (ESI-TOF) m/z calcd for $C_{23}H_{28}N$ $(M + H)^+$: 318.2222, found 318.2227.

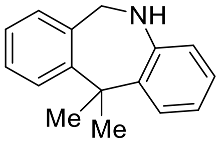

17

11,11-dimethyl-6,11-dihydro-5H-dibenzo[b,e]azepine 17: The procedure **A** was followed using 9,9-dimethyl-9,10-dihydroanthracene (62.5 mg, 0.30 mmol, 1.0 equiv.) and O-tosylhydroxylamine (84.3 mg, 0.45 mmol, 1.5 equiv.). Purified by silica gel chromatography (EtOAc : petroleum ether = 1 : 20) and afforded product **17** as a yellow oil (64.2 mg, 96% yield). **R_f** = 0.4 (EtOAc : petroleum ether = 1 : 10). **^{1}H NMR (400 MHz, Chloroform-d)** δ 7.42 – 7.38 (m, 1H), 7.28 – 7.24 (m, 1H), 7.17 – 7.09 (m, 2H), 7.03 – 6.93 (m, 2H), 6.69 – 6.63 (m, 1H), 6.52 – 6.47 (m, 1H), 4.66 (s, 2H), 3.98 (s, 1H), 1.91 (s, 6H) ppm. **^{13}C NMR (101 MHz, Chloroform-d)** δ 146.77, 145.91, 135.03, 133.29, 129.28, 127.43, 127.30, 126.74, 125.67, 124.55, 118.57, 118.24, 51.17, 40.72, 28.94 ppm. **HRMS** (ESI-TOF) m/z calcd for $C_{16}H_{18}N$ $(M + H)^+$: 224.1439, found 224.1449.

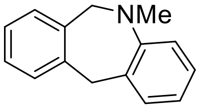

18

5-methyl-6,11-dihydro-5H-dibenzo[b,e]azepine 18: The procedure **A** was followed using 9,10-dihydroanthracene (54.1 mg, 0.30 mmol, 1.0 equiv.) and N-methyl-O-tosylhydroxylamine (90.0 mg, 0.45 mmol, 1.5 equiv.). Purified by silica gel chromatography (EtOAc : petroleum ether = 1 : 50) and afforded product **18** as a colorless oil (38.8 mg, 62% yield). **R_f** = 0.6 (EtOAc : petroleum ether = 1 : 20). **^{1}H NMR (400 MHz, Chloroform-d)** δ 7.21 – 7.17 (m, 1H), 7.17 – 7.09 (m, 4H), 7.04 – 7.00 (m, 1H), 6.93 – 6.89 (m, 1H), 6.84 – 6.79 (m, 1H), 4.33 (s, 2H), 4.14 (s, 2H), 2.93 (s, 3H) ppm. **^{13}C NMR (101 MHz, Chloroform-d)** δ 149.89, 138.55, 136.06, 132.49, 129.07, 128.45, 127.76, 127.43, 126.94, 126.26, 120.87, 118.27, 59.08, 43.09, 40.11 ppm. **HRMS** (ESI-TOF) m/z calcd for $C_{15}H_{16}N$ $(M + H)^+$: 210.1283, found 210.1292.

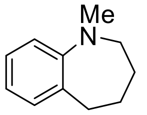

19

1-methyl-2,3,4,5-tetrahydro-1H-benzo[b]azepine 19: The procedure **A** was followed using 1,2,3,4-tetrahydronaphthalene (39.6 mg, 0.30 mmol, 1.0 equiv.) and N-methyl-O-tosylhydroxylamine (90.0 mg, 0.45 mmol, 1.5 equiv.). Purified by silica gel chromatography (EtOAc : petroleum ether = 1 : 50) and afforded product **19** as a light green oil (38.8 mg, 86% yield). Analytical data (^{1}H NMR, ^{13}C NMR) matches with the literature[14]. $\mathbf{R_f}$ = 0.6 (EtOAc : petroleum ether = 1 : 20). **^{1}H NMR (400 MHz, Chloroform-d)** δ 7.17 – 7.07 (m, 2H), 6.93 – 6.82 (m, 2H), 2.91 – 2.88 (m, 2H), 2.87 (s, 3H), 2.80 – 2.76 (m, 2H), 1.79 – 1.73 (m, 2H), 1.62 – 1.55 (m, 2H) ppm. **^{13}C NMR (101 MHz, Chloroform-d)** δ 152.70, 135.50, 129.97, 126.54, 120.83, 116.10, 57.02, 43.11, 35.24, 30.12, 25.58 ppm. **HRMS** (ESI-TOF) m/z calcd for $C_{11}H_{16}N$ (M + H)$^+$: 162.1283, found 162.1892.

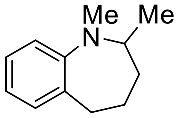

20

1,2-dimethyl-2,3,4,5-tetrahydro-1H-benzo[b]azepine 20: The procedure **A** was followed using 1-methyl-1,2,3,4-tetrahydronaphthalene (43.9 mg, 0.30 mmol, 1.0 equiv.) and N-methyl-O-tosylhydroxylamine (90.0 mg, 0.45 mmol, 1.5 equiv.). Purified by silica gel chromatography (EtOAc : petroleum ether = 1 : 50) and afforded product **20** as a colorless oil (32.2 mg, 61% yield). $\mathbf{R_f}$ = 0.6 (EtOAc : petroleum ether = 1 : 20). **^{1}H NMR (400 MHz, Chloroform-d)** δ 7.17 – 7.10 (m, 1H), 7.08 – 7.03 (m, 1H), 6.90 – 6.81 (m, 2H), 3.27 – 3.19 (m, 1H), 2.86 (s, 3H), 2.81 – 2.65 (m, 2H), 1.87 – 1.74 (m, 1H), 1.65 – 1.52 (m, 3H), 0.90 (d, J = 6.8 Hz, 3H) ppm. **^{13}C NMR (101 MHz, Chloroform-d)** δ 149.63, 135.66, 129.18, 126.41, 120.85, 118.41, 57.15, 41.06, 34.24, 34.01, 20.08, 14.82 ppm. **HRMS** (ESI-TOF) m/z calcd for $C_{12}H_{18}N$ (M + H)$^+$: 176.1439, found 176.1448.

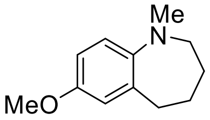

21

7-methoxy-1-methyl-2,3,4,5-tetrahydro-1H-benzo[b]azepine 21: The procedure **A** was followed using 6-methoxy-1,2,3,4-tetrahydronaphthalene (48.7 mg, 0.30 mmol, 1.0 equiv.) and N-methyl-O-tosylhydroxylamine (90.0 mg, 0.45 mmol, 1.5 equiv.). Purified by silica gel chromatography (EtOAc : petroleum ether = 1 : 10) and afforded product **21** as a colorless oil (52.7 mg, 92% yield). $\mathbf{R_f}$ = 0.4 (EtOAc : petroleum ether

= 1 : 10). **^{1}H NMR (400 MHz, Chloroform-d)** δ 6.89 – 6.82 (m, 1H), 6.72 – 6.64 (m, 2H), 3.75 (s, 3H), 2.82 (s, 5H), 2.79 – 2.71 (m, 2H), 1.79 – 1.69 (m, 2H), 1.62 – 1.51 (m, 2H) ppm. **^{13}C NMR (101 MHz, Chloroform-d)** δ 154.01, 146.40, 137.46, 116.93, 116.00, 110.59, 57.29, 55.32, 43.48, 35.27, 30.34, 25.69 ppm. **HRMS** (ESI-TOF) m/z calcd for $C_{12}H_{18}NO$ $(M + H)^+$: 192.1388, found 192.1379.

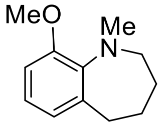

22

9-methoxy-1-methyl-2,3,4,5-tetrahydro-1H-benzo[b]azepine 22: The procedure **A** was followed using 5-methoxy-1,2,3,4-tetrahydronaphthalene (48.7 mg, 0.30 mmol, 1.0 equiv.) and N-methyl-O-tosylhydroxylamine (90.0 mg, 0.45 mmol, 1.5 equiv.). Purified by silica gel chromatography (EtOAc : petroleum ether = 1 : 10) and afforded product **22** as a colorless oil (52.9 mg, 92% yield). **R_f** = 0.4 (EtOAc : petroleum ether = 1 : 10). **^{1}H NMR (400 MHz, Chloroform-d)** δ 6.94 – 6.86 (m, 1H), 6.75 – 6.65 (m, 2H), 3.83 (s, 3H), 3.26 – 3.17 (m, 2H), 2.88 (s, 3H), 2.84 – 2.77 (m, 2H), 1.83 – 1.67 (m, 4H) ppm. **^{13}C NMR (101 MHz, Chloroform-d)** δ 155.09, 139.74, 139.66, 123.21, 122.66, 109.49, 55.67, 53.68, 40.07, 34.07, 26.30, 25.72 ppm. **HRMS** (ESI-TOF) m/z calcd for $C_{12}H_{18}NO$ $(M + H)^+$: 192.1388, found 192.1395.

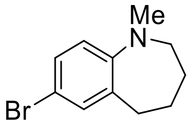

23

7-bromo-1-methyl-2,3,4,5-tetrahydro-1H-benzo[b]azepine 23: The procedure **A** was followed using 6-bromo-1,2,3,4-tetrahydronaphthalene (63.3 mg, 0.30 mmol, 1.0 equiv.) and N-methyl-O-tosylhydroxylamine (90.0 mg, 0.45 mmol, 1.5 equiv.) at 60℃. Purified by silica gel chromatography (EtOAc : petroleum ether = 1 : 50) and afforded product **23** as a yellow oil (47.3 mg, 65% yield). **R_f** = 0.6 (EtOAc : petroleum ether = 1 : 20). **^{1}H NMR (400 MHz, Chloroform-d)** δ 7.24 – 7.18 (m, 2H), 6.78 – 6.72 (m, 1H), 2.90 – 2.85 (m, 2H), 2.83 (s, 3H), 2.75 – 2.69 (m, 2H), 1.77 – 1.70 (m, 2H), 1.60 – 1.53 (m, 2H) ppm. **^{13}C NMR (101 MHz, Chloroform-d)** δ 151.73, 137.49, 132.43, 129.13, 117.81, 113.06, 56.80, 43.05, 34.84, 29.69, 25.21 ppm. **HRMS** (ESI-TOF) m/z calcd for $C_{11}H_{15}NBr$ $(M + H)^+$: 240.0388, found 240.0395.

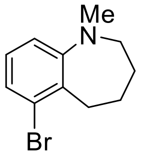

24

6-bromo-1-methyl-2,3,4,5-tetrahydro-1H-benzo[b]azepine 24: The procedure **A** was followed using 5-bromo-1,2,3,4-tetrahydronaphthalene (63.3 mg, 0.30 mmol, 1.0 equiv.) and N-methyl-O-tosylhydroxylamine (90.0 mg, 0.45 mmol, 1.5 equiv.) at 60℃. Purified by silica gel chromatography (EtOAc : petroleum ether = 1 : 50) and afforded product **24** as a yellow oil (32.2 mg, 45% yield). $\mathbf{R_f}$ = 0.6 (EtOAc : petroleum ether = 1 : 20). ^{1}**H NMR (400 MHz, Chloroform-d)** δ 7.15 – 7.10 (m, 1H), 6.99 – 6.93 (m, 1H), 6.83 – 6.79 (m, 1H), 3.07 – 3.02 (m, 2H), 2.99 – 2.95 (m, 2H), 2.84 (s, 3H), 1.71 – 1.59 (m, 4H) ppm. ^{13}**C NMR (101 MHz, Chloroform-d)** δ 153.38, 133.91, 127.24, 124.99, 124.74, 115.50, 56.26, 42.54, 32.23, 27.66, 23.10 ppm. **HRMS** (ESI-TOF) m/z calcd for $C_{11}H_{15}NBr$ $(M + H)^+$: 240.0388, found 240.0390.

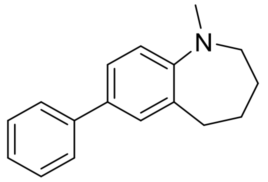

25

1-methyl-7-phenyl-2,3,4,5-tetrahydro-1H-benzo[b]azepine 25: The procedure **A** was followed using 6-phenyl-1,2,3,4-tetrahydronaphthalene (62.5 mg, 0.30 mmol, 1.0 equiv.) and N-methyl-O-tosylhydroxylamine (90.0 mg, 0.45 mmol, 1.5 equiv.). Purified by silica gel chromatography (EtOAc : petroleum ether = 1 : 50) and afforded product **25** as a yellow oil (54.4 mg, 76% yield). $\mathbf{R_f}$ = 0.5 (EtOAc : petroleum ether = 1 : 50). ^{1}**H NMR (400 MHz, Chloroform-d)** δ 7.58 – 7.54 (m, 2H), 7.42 – 7.33 (m, 4H), 7.29 – 7.23 (m, 1H), 6.99 – 6.94 (m, 1H), 2.95 – 2.92 (m, 2H), 2.90 (s, 3H), 2.87 – 2.82 (m, 2H), 1.82 – 1.75 (m, 2H), 1.67 – 1.60 (m, 2H) ppm. ^{13}**C NMR (101 MHz, Chloroform-d)** δ 152.10, 141.14, 135.53, 133.45, 128.80, 128.57, 126.64, 126.33, 125.05, 116.44, 57.02, 43.14, 35.45, 30.01, 25.61 ppm. **HRMS** (ESI-TOF) m/z calcd for $C_{17}H_{20}N$ $(M + H)^+$: 238.1590, found 238.1598.

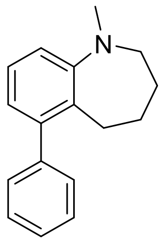

26

1-methyl-6-phenyl-2,3,4,5-tetrahydro-1H-benzo[b]azepine 26: The procedure **A** was followed using 5-phenyl-1,2,3,4-tetrahydronaphthalene (62.5 mg, 0.30 mmol, 1.0 equiv.) and N-methyl-O-tosylhydroxylamine (90.0 mg, 0.45 mmol, 1.5 equiv.). Purified by silica gel chromatography (EtOAc : petroleum ether = 1 : 50) and afforded product **26** as a yellow oil (59.8 mg, 84% yield). $\mathbf{R_f}$ = 0.5 (EtOAc : petroleum ether = 1 : 50). ^{1}**H NMR (400 MHz, Chloroform-d)** δ 7.40 – 7.28 (m, 5H), 7.19 – 7.13 (m, 1H), 6.95 – 6.90 (m, 1H), 6.89 – 6.84 (m, 1H), 3.00 – 2.96 (m, 2H), 2.89 (s, 3H), 2.70

– 2.65 (m, 2H), 1.74 (p, J = 5.8 Hz, 2H), 1.62 – 1.55 (m, 2H) ppm. **^{13}C NMR (101 MHz, Chloroform-d)** *δ* 152.67, 142.83, 142.51, 132.90, 129.32, 127.86, 126.48, 125.82, 122.86, 115.44, 56.69, 42.95, 29.55, 28.54, 25.14 ppm. **HRMS** (ESI-TOF) m/z calcd for $C_{17}H_{20}N$ $(M + H)^+$: 238.1590, found 238.1598.

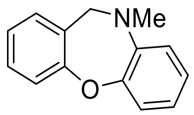

27

10-methyl-10,11-dihydrodibenzo[b,f][1,4]oxazepine 27: The procedure **A** was followed using 9H-xanthene (54.7 mg, 0.30 mmol, 1.0 equiv.) and N-methyl-O-tosylhydroxylamine (90.0 mg, 0.45 mmol, 1.5 equiv.). Purified by silica gel chromatography (EtOAc : petroleum ether = 1 : 20) and afforded product **27** as a yellow oil (64.3 mg, 86% yield). Analytical data (^{1}H NMR, ^{13}C NMR) matches with the literature[15]. **R_f** = 0.4 (EtOAc : petroleum ether = 1 : 20). **^{1}H NMR (400 MHz, Chloroform-d)** *δ* 7.24 – 7.16 (m, 2H), 7.14 – 7.09 (m, 2H), 7.05 – 6.96 (m, 2H), 6.89 – 6.86 (m, 1H), 6.83 – 6.78 (m, 1H), 4.34 (s, 2H), 2.92 (s, 3H) ppm. **^{13}C NMR (101 MHz, Chloroform-d)** *δ* 157.18, 148.83, 142.34, 129.31, 128.63, 128.51, 124.46, 123.56, 121.62, 121.03, 120.27, 119.90, 56.42, 42.89 ppm. **HRMS** (ESI-TOF) m/z calcd for $C_{14}H_{14}NO$ $(M + H)^+$: 212.1075, found 212.1085.

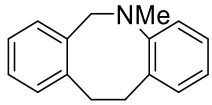

28

5-methyl-5,6,11,12-tetrahydrodibenzo[b,f]azocine 28: The procedure **A** was followed using 10,11-dihydro-5H-dibenzo[a,d][7]annulene (58.3 mg, 0.30 mmol, 1.0 equiv.) and N-methyl-O-tosylhydroxylamine (90.0 mg, 0.45 mmol, 1.5 equiv.). Purified by silica gel chromatography (EtOAc : petroleum ether = 1 : 50) and afforded product **28** as a yellow oil (64.2 mg, 96% yield). Analytical data (^{1}H NMR, ^{13}C NMR) matches with the literature[16]. **R_f** = 0.6 (EtOAc : petroleum ether = 1 : 20). **^{1}H NMR (400 MHz, Chloroform-d)** *δ* 7.18 – 7.05 (m, 6H), 6.96 – 6.92 (m, 1H), 6.83 – 6.77 (m, 1H), 4.19 (s, 2H), 3.22 – 3.16 (m, 2H), 3.15 – 3.09 (m, 2H), 2.86 (s, 3H) ppm. **^{13}C NMR (101 MHz, Chloroform-d)** *δ* 151.34, 141.96, 137.22, 133.69, 131.22, 129.82, 128.97, 127.30, 126.77, 125.90, 120.23, 116.88, 60.37, 39.66, 33.41, 33.29 ppm. **HRMS** (ESI-TOF) m/z calcd for $C_{16}H_{18}N$ $(M + H)^+$: 224.1439, found 224.1446.

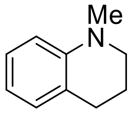

29

1-methyl-1,2,3,4-tetrahydroquinoline 29: The procedure **A** was followed using 2,3-dihydro-1H-indene (35.5 mg, 0.30 mmol, 1.0 equiv.) and N-methyl-O-tosylhydroxylamine (90.0 mg, 0.45 mmol, 1.5 equiv.). Purified by silica gel chromatography (EtOAc : petroleum ether = 1 : 50) and afforded product **29** as a yellow oil (32.5 mg, 74% yield). Analytical data (^{1}H NMR, ^{13}C NMR) matches with the literature[17]. $\mathbf{R_f}$ = 0.6 (EtOAc : petroleum ether = 1 : 20). **^{1}H NMR (400 MHz, Chloroform-d)** δ 7.09 – 7.03 (m, 1H), 6.96 – 6.92 (m, 1H), 6.62 – 6.56 (m, 2H), 3.22 – 3.18 (m, 2H), 2.87 (s, 3H), 2.76 (t, J = 6.5 Hz, 2H), 2.01 – 1.94 (m, 2H) ppm. **^{13}C NMR (101 MHz, Chloroform-d)** δ 146.71, 128.77, 127.00, 122.81, 116.15, 110.91, 51.24, 39.08, 27.75, 22.42 ppm. **HRMS** (ESI-TOF) m/z calcd for $C_{10}H_{14}N$ $(M + H)^+$: 148.1126, found 148.1133.

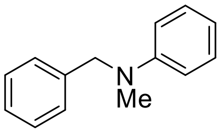

30

N-benzyl-N-methylaniline 30: The procedure **A** was followed using diphenylmethane (50.5 mg, 0.30 mmol, 1.0 equiv.) and N-methyl-O-tosylhydroxylamine (90.0 mg, 0.45 mmol, 1.5 equiv.). Purified by silica gel chromatography (EtOAc : petroleum ether = 1 : 50) and afforded product **30** as a yellow oil (57.9 mg, 98% yield). Analytical data (^{1}H NMR, ^{13}C NMR) matches with the literature.[18] $\mathbf{R_f}$ = 0.6 (EtOAc : petroleum ether = 1 : 20). **^{1}H NMR (400 MHz, Chloroform-d)** δ 7.32 – 7.26 (m, 2H), 7.25 – 7.17 (m, 5H), 6.78 – 6.67 (m, 3H), 4.51 (s, 2H), 2.99 (s, 3H) ppm. **^{13}C NMR (101 MHz, Chloroform-d)** δ 149.69, 138.98, 129.13, 128.50, 126.80, 126.67, 116.47, 112.29, 56.55, 38.44 ppm. **HRMS** (ESI-TOF) m/z calcd for $C_{14}H_{16}N$ $(M + H)^+$: 198.1283, found 198.1290.

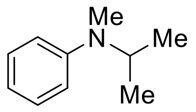

31

N-isopropyl-N-methylaniline 31: The procedure **A** was followed using cumene (36.1 mg, 0.30 mmol, 1.0 equiv.) and N-methyl-O-tosylhydroxylamine (90.0 mg, 0.45 mmol, 1.5 equiv.). Purified by silica gel chromatography (EtOAc : petroleum ether = 1 : 50) and afforded product **31** as a yellow oil (35.5 mg, 79% yield). Analytical data (^{1}H NMR, ^{13}C NMR) matches with the literature.[18] $\mathbf{R_f}$ = 0.6 (EtOAc : petroleum ether = 1 : 20). **^{1}H NMR (400 MHz, Chloroform-d)** δ 7.25 – 7.20 (m, 2H), 6.82 – 6.76 (m, 2H), 6.72 – 6.66 (m, 1H), 4.14 – 4.04 (m, 1H), 2.72 (s, 3H), 1.16 (d, J = 6.6 Hz, 6H) ppm. **^{13}C NMR (101 MHz, Chloroform-d)** δ 150.18, 129.08, 116.35, 113.27, 48.85, 29.73, 19.29 ppm. **HRMS** (ESI-TOF) m/z calcd for $C_{10}H_{16}N$ $(M + H)^+$: 150.1283, found 150.1293.

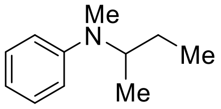

32

N-(sec-butyl)-N-methylaniline 32: The procedure **A** was followed using sec-butylbenzene (40.3 mg, 0.30 mmol, 1.0 equiv.) and N-methyl-O-tosylhydroxylamine (90.0 mg, 0.45 mmol, 1.5 equiv.). Purified by silica gel chromatography (EtOAc : petroleum ether = 1 : 50) and afforded product **32** as a yellow oil (31.4 mg, 64% yield). Analytical data (^{1}H NMR, ^{13}C NMR) matches with the literature.[19] $\mathbf{R_f}$ = 0.6 (EtOAc : petroleum ether = 1 : 20). **^{1}H NMR (400 MHz, Chloroform-d)** δ 7.25 – 7.19 (m, 2H), 6.80 – 6.75 (m, 2H), 6.69 – 6.64 (m, 1H), 3.86 – 3.75 (m, 1H), 2.70 (s, 3H), 1.64 – 1.46 (m, 2H), 1.11 (d, *J* = 6.6 Hz, 3H), 0.88 (t, *J* = 7.4 Hz, 3H) ppm. **^{13}C NMR (101 MHz, Chloroform-d)** δ 150.68, 129.06, 115.95, 112.85, 54.93, 29.67, 27.48, 16.86, 11.51 ppm. **HRMS** (ESI-TOF) m/z calcd for $C_{11}H_{18}N$ $(M + H)^+$: 164.1439, found 164.1446

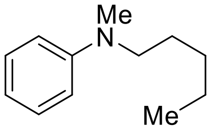

33

N-methyl-N-pentylaniline 33: The procedure **A** was followed using pentylbenzene (44.5 mg, 0.30 mmol, 1.0 equiv.) and N-methyl-O-tosylhydroxylamine (90.0 mg, 0.45 mmol, 1.5 equiv.). Purified by silica gel chromatography (EtOAc : petroleum ether = 1 : 50) and afforded product **33** as a yellow oil (22.5 mg, 42% yield). Analytical data (^{1}H NMR, ^{13}C NMR) matches with the literature.[20] $\mathbf{R_f}$ = 0.6 (EtOAc : petroleum ether = 1 : 20). **^{1}H NMR (400 MHz, Chloroform-d)** δ 7.24 – 7.19 (m, 2H), 6.72 – 6.63 (m, 3H), 3.32 – 3.26 (m, 2H), 2.92 (s, 3H), 1.61 – 1.53 (m, 2H), 1.36 – 1.28 (m, 4H), 0.90 (t, *J* = 7.0 Hz, 3H) ppm. **^{13}C NMR (101 MHz, Chloroform-d)** δ 149.35, 129.10, 115.74, 112.03, 52.78, 38.24, 29.36, 26.32, 22.61, 14.09 ppm. **HRMS** (ESI-TOF) m/z calcd for $C_{12}H_{20}N$ $(M + H)^+$: 178.1596, found 178.1594.

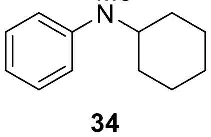

34

N-cyclohexyl-N-methylaniline 34: The procedure **A** was followed using (cyclohexylmethyl)benzene (48.1 mg, 0.30 mmol, 1.0 equiv.) and N-methyl-O-tosylhydroxylamine (90.0 mg, 0.45 mmol, 1.5 equiv.). Purified by silica gel chromatography (EtOAc : petroleum ether = 1 : 50) and afforded product **34** as a yellow oil (31.1 mg, 55% yield). Analytical data (^{1}H NMR, ^{13}C NMR) matches with the literature.[20] $\mathbf{R_f}$ = 0.6 (EtOAc : petroleum ether = 1 : 20). **^{1}H NMR (400 MHz,**

Chloroform-d) *δ* 7.24 – 7.19 (m, 2H), 6.80 – 6.74 (m, 2H), 6.71 – 6.63 (m, 1H), 3.61 – 3.51 (m, 1H), 2.76 (s, 3H), 1.87 – 1.75 (m, 4H), 1.72 – 1.64 (m, 1H), 1.50 – 1.31 (m, 4H), 1.18 – 1.07 (m, 1H) ppm. **^{13}C NMR (101 MHz, Chloroform-d)** *δ* 150.14, 129.06, 116.17, 113.10, 58.08, 31.11, 30.02, 26.19, 25.92 ppm. **HRMS** (ESI-TOF) m/z calcd for $C_{13}H_{20}N$ $(M + H)^+$: 190.1596, found 190.1605.

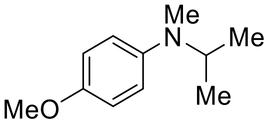

35

N-isopropyl-4-methoxy-N-methylaniline 35: The procedure **A** was followed using 1-isopropyl-4-methoxybenzene (45.1 mg, 0.30 mmol, 1.0 equiv.) and N-methyl-O-tosylhydroxylamine (90.0 mg, 0.45 mmol, 1.5 equiv.). Purified by silica gel chromatography (EtOAc : petroleum ether = 1 : 20) and afforded product **35** as a yellow oil (49.2 mg, 92% yield). Analytical data (^{1}H NMR, ^{13}C NMR) matches with the literature.[21] **R_f** = 0.4 (EtOAc : petroleum ether = 1 : 20). **^{1}H NMR (400 MHz, Chloroform-d)** *δ* 6.85 – 6.76 (m, 4H), 3.89 (p, *J* = 6.6 Hz, 1H), 3.75 (s, 3H), 2.65 (s, 3H), 1.11 (d, *J* = 6.6 Hz, 6H) ppm. **^{13}C NMR (101 MHz, Chloroform-d)** *δ* 151.99, 145.09, 116.51, 114.45, 55.61, 50.88, 30.80, 18.94 ppm. **HRMS** (ESI-TOF) m/z calcd for $C_{11}H_{18}NO$ $(M + H)^+$: 180.1388, found 180.1389.

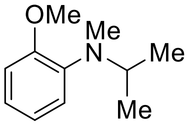

36

N-isopropyl-2-methoxy-N-methylaniline 36: The procedure **A** was followed using 1-isopropyl-2-methoxybenzene (45.1 mg, 0.30 mmol, 1.0 equiv.) and N-methyl-O-tosylhydroxylamine (90.0 mg, 0.45 mmol, 1.5 equiv.). Purified by silica gel chromatography (EtOAc : petroleum ether = 1 : 20) and afforded product **36** as a yellow oil (31.2 mg, 69% yield). Analytical data (^{1}H NMR, ^{13}C NMR) matches with the literature.[21] **R_f** = 0.4 (EtOAc : petroleum ether = 1 : 20). **^{1}H NMR (400 MHz, Chloroform-d)** *δ* 6.99 – 6.93 (m, 2H), 6.93 – 6.86 (m, 1H), 6.86 – 6.81 (m, 1H), 3.85 (s, 3H), 3.70 (p, *J* = 6.7 Hz, 1H), 2.66 (s, 3H), 1.08 (d, *J* = 6.7 Hz, 6H) ppm. **^{13}C NMR (101 MHz, Chloroform-d)** *δ* 152.86, 141.78, 122.24, 120.60, 120.49, 111.06, 55.32, 51.72, 31.87, 18.48 ppm. **HRMS** (ESI-TOF) m/z calcd for $C_{11}H_{18}NO$ $(M + H)^+$: 180.1388, found 180.1391.

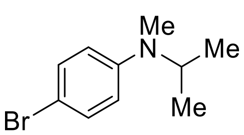

37

4-bromo-N-isopropyl-N-methylaniline 37: The procedure **A** was followed using 1-bromo-4-isopropylbenzene (59.7 mg, 0.30 mmol, 1.0 equiv.) and N-methyl-O-tosylhydroxylamine (90.0 mg, 0.45 mmol, 1.5 equiv.). Purified by silica gel chromatography (EtOAc : petroleum ether = 1 : 50) and afforded product **37** as a yellow oil (58.6 mg, 86% yield). Analytical data (^{1}H NMR, ^{13}C NMR) matches with the literature.[22] **R_f** = 0.6 (EtOAc : petroleum ether = 1 : 20). **^{1}H NMR (400 MHz, Chloroform-d)** δ 7.30 – 7.25 (m, 2H), 6.67 – 6.61 (m, 2H), 4.08 – 3.96 (m, 1H), 2.69 (s, 3H), 1.15 (d, J = 6.6 Hz, 6H) ppm. **^{13}C NMR (101 MHz, Chloroform-d)** δ 149.11, 131.69, 114.77, 108.04, 49.00, 29.77, 19.23 ppm. **HRMS** (ESI-TOF) m/z calcd for $C_{10}H_{15}N$ (M + H)$^{+}$: 228.0388, found 228.0394.

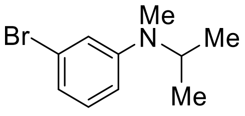

38

3-bromo-N-isopropyl-N-methylaniline 38: The procedure **A** was followed using 1-bromo-3-isopropylbenzene (59.7 mg, 0.30 mmol, 1.0 equiv.) and N-methyl-O-tosylhydroxylamine (90.0 mg, 0.45 mmol, 1.5 equiv.). Purified by silica gel chromatography (EtOAc : petroleum ether = 1 : 50) and afforded product **38** as a yellow oil (30.7 mg, 49% yield). **R_f** = 0.6 (EtOAc : petroleum ether = 1 : 20). **^{1}H NMR (400 MHz, Chloroform-d)** δ 7.08 – 7.01 (m, 1H), 6.89 – 6.85 (m, 1H), 6.80 – 6.75 (m, 1H), 6.70 – 6.63 (m, 1H), 4.09 – 3.98 (m, 1H), 2.70 (s, 3H), 1.15 (d, J = 6.6 Hz, 6H) ppm. **^{13}C NMR (101 MHz, Chloroform-d)** δ 151.27, 130.23, 123.44, 118.80, 115.60, 111.40, 48.77, 29.70, 19.35 ppm. **HRMS** (ESI-TOF) m/z calcd for $C_{10}H_{15}N$ (M + H)$^{+}$: 228.0388, found 228.0389.

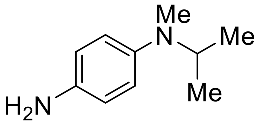

39

N^{1}-isopropyl-N^{1}-methylbenzene-1,4-diamine 39: The procedure **A** was followed using 4-isopropylaniline (40.6 mg, 0.30 mmol, 1.0 equiv.) and N-methyl-O-tosylhydroxylamine (90.0 mg, 0.45 mmol, 1.5 equiv.). Purified by silica gel chromatography (EtOAc : petroleum ether = 1 : 1) and afforded product **39** as a brown oil (31.7 mg, 64% yield). **R_f** = 0.2 (EtOAc : petroleum ether = 1 : 1). **^{1}H NMR (400 MHz, Chloroform-d)** δ 6.77 – 6.71 (m, 2H), 6.68 – 6.61 (m, 2H), 3.86 – 3.75 (m, 1H), 3.35 (brs, 2H), 2.63 (s, 3H), 1.09 (d, J = 6.7 Hz, 6H) ppm. **^{13}C NMR (101 MHz, Chloroform-d)** δ 144.07, 138.12, 117.62, 116.43, 51.43, 31.27, 18.91 ppm. **HRMS** (ESI-TOF) m/z calcd for $C_{10}H_{17}N_2$ (M + H)$^{+}$: 165.1392, found 165.1386.

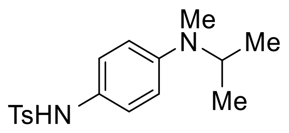

40

N-(4-(isopropyl(methyl)amino)phenyl)-4-methylbenzenesulfonamide 40: The procedure **A** was followed using 4-isopropylaniline (86.8 mg, 0.30 mmol, 1.0 equiv.) and N-methyl-O-tosylhydroxylamine (90.0 mg, 0.45 mmol, 1.5 equiv.). Purified by silica gel chromatography (EtOAc : petroleum ether = 1 : 5) and afforded product **40** as a colorless oil (81.3 mg, 85% yield). $\mathbf{R_f}$ = 0.4 (EtOAc : petroleum ether = 1 : 1). **^{1}H NMR (400 MHz, Chloroform-d)** δ 7.60 – 7.55 (m, 2H), 7.24 – 7.18 (m, 2H), 6.90 – 6.84 (m, 2H), 6.66 – 6.59 (m, 2H), 6.13 (s, 1H), 4.06 – 3.96 (m, 1H), 2.68 (s, 3H), 2.39 (s, 3H), 1.13 (d, J = 6.6 Hz, 6H) ppm. **^{13}C NMR (101 MHz, Chloroform-d)** δ 148.85, 143.32, 136.31, 129.40, 127.36, 126.16, 124.45, 113.42, 49.02, 29.78, 21.50, 19.19 ppm. **HRMS** (ESI-TOF) m/z calcd for $C_{17}H_{23}N_2O_2S$ $(M + H)^+$: 319.1480, found 319.1487.

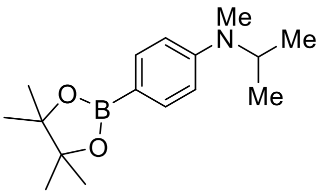

41

N-isopropyl-N-methyl-4-(4,4,5,5-tetramethyl-1,3,2-dioxaborolan-2-yl)aniline 41: The procedure **A** was followed using 2-(4-isopropylphenyl)-4,4,5,5-tetramethyl-1,3,2-dioxaborolane (73.8 mg, 0.30 mmol, 1.0 equiv.) and N-methyl-O-tosylhydroxylamine (90.0 mg, 0.45 mmol, 1.5 equiv.) for 60 h. Purified by silica gel chromatography (EtOAc : petroleum ether = 1 : 50) and afforded product **41** as a white wox (44.7 mg, 54% yield). $\mathbf{R_f}$ = 0.4 (EtOAc : petroleum ether = 1 : 20). **^{1}H NMR (400 MHz, Chloroform-d)** δ 7.70 – 7.64 (m, 2H), 6.76 – 6.71 (m, 2H), 4.23 – 4.11 (m, 1H), 2.76 (s, 3H), 1.32 (s, 12H), 1.17 (d, J = 6.6 Hz, 6H) ppm. **^{13}C NMR (101 MHz, Chloroform-d)** δ 152.16, 136.23, 111.59, 83.09, 48.11, 29.55, 24.81, 19.44 ppm. **HRMS** (ESI-TOF) m/z calcd for $C_{16}H_{25}BNO_2$ $(M - H)^-$: 273.2015, found 273.2024.

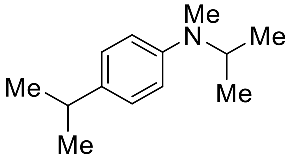

42

N,4-diisopropyl-N-methylaniline 42: The procedure **A** was followed using 1,4-diisopropylbenzene (48.7 mg, 0.30 mmol, 1.0 equiv.) and N-methyl-O-tosylhydroxylamine (90.0 mg, 0.45 mmol, 1.5 equiv.). Purified by silica gel chromatography (EtOAc : petroleum ether = 1 : 50) and afforded product **42** as a yellow oil (54.3 mg, 95% yield). $\mathbf{R_f}$ = 0.6 (EtOAc : petroleum ether = 1 : 20). **^{1}H**

NMR (400 MHz, Chloroform-d) δ 7.12 – 7.07 (m, 2H), 6.76 – 6.71 (m, 2H), 4.10 – 3.98 (m, 1H), 2.87 – 2.76 (m, 1H), 2.70 (s, 3H), 1.22 (d, J = 6.9 Hz, 6H), 1.14 (d, J = 6.6 Hz, 6H) ppm. **^{13}C NMR (101 MHz, Chloroform-d)** δ 148.36, 136.83, 126.91, 113.46, 49.08, 32.97, 29.91, 24.21, 19.26 ppm. **HRMS** (ESI-TOF) m/z calcd for $C_{13}H_{22}N$ $(M + H)^+$: 192.1752, found 192.1761.

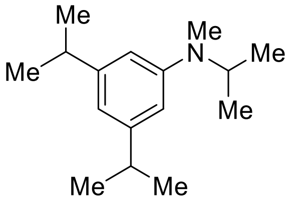

43

N,3,5-triisopropyl-N-methylaniline 43: The procedure **A** was followed using 1,3,5-triisopropylbenzene (61.3 mg, 0.30 mmol, 1.0 equiv.) and N-methyl-O-tosylhydroxylamine (90.0 mg, 0.45 mmol, 1.5 equiv.). Purified by silica gel chromatography (EtOAc : petroleum ether = 1 : 50) and afforded product **43** as a yellow oil (68.2 mg, 96% yield). **R_f** = 0.6 (EtOAc : petroleum ether = 1 : 20). **^{1}H NMR (400 MHz, Chloroform-d)** δ 6.54 – 6.42 (m, 3H), 4.15 – 4.02 (m, 1H), 2.88 – 2.77 (m, 2H), 2.73 (s, 3H), 1.25 (d, J = 7.0 Hz, 12H), 1.16 (d, J = 6.6 Hz, 6H) ppm. **^{13}C NMR (101 MHz, Chloroform-d)** δ 150.18, 149.60, 113.00, 109.17, 48.86, 34.66, 29.98, 24.12, 19.44 ppm. **HRMS** (ESI-TOF) m/z calcd for $C_{16}H_{28}N$ $(M + H)^+$: 234.2222, found 234.2231.

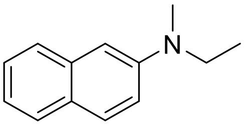

44

N-ethyl-N-methylnaphthalen-2-amine 44: The procedure **A** was followed using 2-ethylnaphthalene (46.9 mg, 0.30 mmol, 1.0 equiv.) and N-methyl-O-tosylhydroxylamine (90.0 mg, 0.45 mmol, 1.5 equiv.). Purified by silica gel chromatography (EtOAc : petroleum ether = 1 : 20) and afforded product **44** as a yellow oil (46.5 mg, 84% yield). **R_f** = 0.4 (EtOAc : petroleum ether = 1 : 20). **^{1}H NMR (400 MHz, Chloroform-d)** δ 8.25 – 8.19 (m, 1H), 7.83 – 7.76 (m, 1H), 7.56 – 7.32 (m, 4H), 7.10 – 7.04 (m, 1H), 3.12 (q, J = 7.1 Hz, 2H), 2.84 (s, 3H), 1.19 (t, J = 7.1 Hz, 3H) ppm. **^{13}C NMR (101 MHz, Chloroform-d)** δ 150.27, 134.79, 129.52, 128.24, 125.63, 125.07, 123.99, 122.87, 115.32, 51.47, 41.23, 12.72 ppm. **HRMS** (ESI-TOF) m/z calcd for $C_{13}H_{16}N$ $(M + H)^+$: 186.1277, found 186.1285.

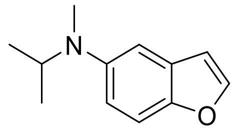

45

N-isopropyl-N-methylbenzofuran-5-amine 45: The procedure **A** was followed using 5-isopropylbenzofuran (48.1 mg, 0.30 mmol, 1.0 equiv.) and N-methyl-O-tosylhydroxylamine (90.0 mg, 0.45 mmol, 1.5 equiv.). Purified by silica gel chromatography (EtOAc : petroleum ether = 1 : 20) and afforded product **45** as a yellow oil (31.2 mg, 55% yield). $\mathbf{R_f}$ = 0.4 (EtOAc : petroleum ether = 1 : 20). ^{1}**H NMR (400 MHz, Chloroform-d)** δ 7.56 – 7.49 (m, 1H), 7.39 – 7.33 (m, 1H), 7.02 – 6.96 (m, 1H), 6.95 – 6.88 (m, 1H), 6.68 – 6.61 (m, 1H), 4.04 – 3.93 (m, 1H), 2.72 (s, 3H), 1.14 (d, J = 6.6 Hz, 6H) ppm. ^{13}**C NMR (101 MHz, Chloroform-d)** δ 149.04, 147.44, 145.15, 128.01, 114.64, 111.30, 106.65, 106.56, 51.80, 31.29, 19.10 ppm. **HRMS** (ESI-TOF) m/z calcd for $C_{12}H_{16}NO$ $(M + H)^+$: 190.1226, found 190.1233.

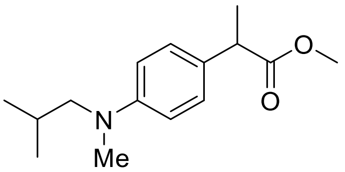

48

Methyl 2-(4-(isobutyl(methyl)amino)phenyl)propanoate 48: The procedure **A** was followed using ibuprofen methyl ester (69.1 mg, 0.30 mmol, 1.0 equiv.) and N-methyl-O-tosylhydroxylamine (90.0 mg, 0.45 mmol, 1.5 equiv.). Purified by silica gel chromatography (EtOAc : petroleum ether = 1 : 10) and afforded product **48** as a colorless oil (48.3 mg, 65% yield). $\mathbf{R_f}$ = 0.5 (EtOAc : petroleum ether = 1 : 5). ^{1}**H NMR (400 MHz, Chloroform-d)** δ 7.17 – 7.11 (m, 2H), 6.65 – 6.58 (m, 2H), 3.64 (s, 4H), 3.06 (d, J = 7.3 Hz, 2H), 2.94 (s, 3H), 2.09 – 1.97 (m, 1H), 1.46 (d, J = 7.2 Hz, 3H), 0.92 (d, J = 6.6 Hz, 6H) ppm. ^{13}**C NMR (101 MHz, Chloroform-d)** δ 175.70, 148.74, 128.05, 127.26, 111.74, 60.99, 51.86, 44.32, 39.46, 27.39, 20.41, 18.61 ppm. **HRMS** (ESI-TOF) m/z calcd for $C_{15}H_{24}NO_2$ $(M + H)^+$: 250.1807, found 250.1810.

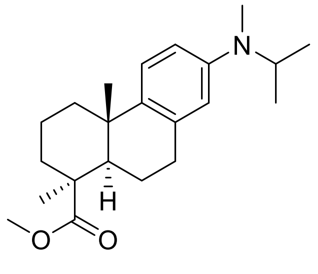

49

Methyl (1S,4aS,10aR)-7-(isopropyl(methyl)amino)-1,4a-dimethyl-1,2,3,4,4a,9,10,10a-octahydrophenanthrene-1-carboxylate 49: The procedure **A** was followed using dehydroabietic acid methyl ester (94.3 mg, 0.30 mmol, 1.0 equiv.)

and N-methyl-O-tosylhydroxylamine (90.0 mg, 0.45 mmol, 1.5 equiv.). Purified by silica gel chromatography (EtOAc : petroleum ether = 1 : 1) and afforded product **49** as a colorless oil (49.2 mg, 48% yield). **R_f** = 0.3 (EtOAc : petroleum ether = 1 : 1). **^{1}H NMR (400 MHz, Chloroform-d)** δ 7.12 – 7.05 (m, 1H), 6.66 – 6.58 (m, 1H), 6.45 – 6.39 (m, 1H), 4.10 – 3.96 (m, 1H), 3.65 (s, 3H), 2.95 – 2.78 (m, 2H), 2.68 (s, 3H), 2.30 – 2.18 (m, 2H), 1.89 – 1.61 (m, 5H), 1.52 – 1.41 (m, 1H), 1.42 – 1.33 (m, 1H), 1.26 (s, 3H), 1.19 (s, 3H), 1.13 (d, *J* = 6.6 Hz, 6H) ppm. **^{13}C NMR (101 MHz, Chloroform-d)** δ 179.17, 147.77, 137.92, 135.48, 124.82, 112.85, 111.44, 51.79, 48.61, 47.57, 45.17, 38.09, 36.64, 36.32, 30.43, 29.72, 25.08, 21.81, 19.31, 18.58, 16.40 ppm. **HRMS** (ESI-TOF) m/z calcd for $C_{22}H_{32}NO_2$ (M - H)$^-$: 342.2433, found 342.2430.

6. Further transformations

6.1 Gram-scaled experiment:

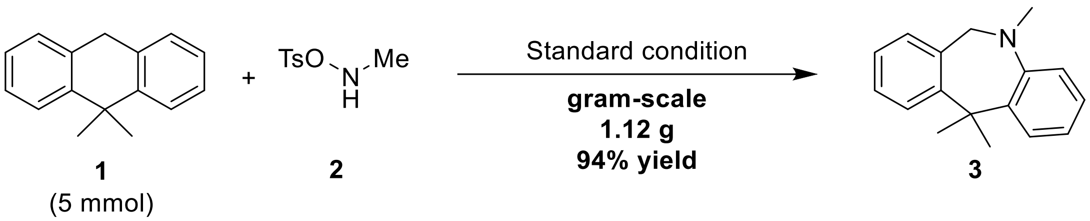

1 (5 mmol) 2 3

Supplementary Fig 15. Gram-scaled experiment

In an oven dried 100 mL round bottom flask, which containing a stirring bar, was charged with 9,9-dimethyl-9,10-dihydroanthracene **1** (5.0 mmol, 1.04 g, 1.0 equiv.), N-methyl-O-tosylhydroxylamine **2** (7.5 mmol, 1.51 g, 1.5 equiv.), DDQ (7.5 mmol, 1.70 g, 1.5 equiv.). The flask was then evacuated and back-filled under N_2 flow (this sequence was repeated three times). HFIP (25 mL) and H_2O (50 mmol, 0.9 mL, 10.0 equiv.) were added and stirred at room temperature for 12 h. Then $NaBH_3CN$ (25 mmol, 1.57 g, 5.0 equiv.) was added to the above reaction mixture and the reaction was stirred for 2 h at room temperature. The reaction was quenched with 40 mL saturated $NaHCO_3$ aq. and 80 mL H_2O. Then it was extracted with DCM (80 mL × 3). The organic layer was combined and dried over Na_2SO_4. Then filtered and concentrated by rotary evaporation. The residue was purified by silica gel chromatography (EtOAc : petroleum ether = 1 : 50) to afford the product **3** as a white wox (1.12 g, 94% yield).

6.2 Synthesis of 5-(3-bromopropyl)-5,6,11,12-tetrahydrodibenzo[b,f]azocine 51 and 4-(4-chlorophenyl)-1-(3-(11,12-dihydrodibenzo[b,f]azocin-5(6H)-yl)propyl)piperidin-4-ol 54:

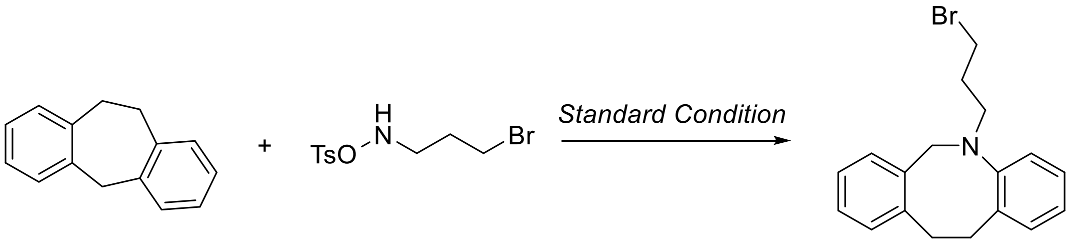

50 51

Supplementary Fig 16. Synthesis of compound **51**

In an oven dried 50 mL Schlenk tube, which containing a stirring bar, was charged with 10,11-dihydro-5H-dibenzo[a,d][7]annulene **50** (1.0 mmol, 194.3 mg, 1.0 equiv.), N-(3-bromopropyl)-O-tosylhydroxylamine (1.5 mmol, 462.3 mg, 1.5 equiv.), DDQ (1.5 mmol, 340.5 mg, 1.5 equiv.). The tube was then evacuated and bak-filled under N_2 flow (this sequence was repeated three times). HFIP (5.0 mL) and H_2O (0.18 mL, 10.0 equiv.) were added and stirred at room temperature for 12 h. Then $NaBH_3CN$ (5 mmol, 314.2 mg, 5.0 equiv.) was added to the above reaction mixture and the reaction was stirred for 2 h at room temperature. The reaction was quenched with 10 mL saturated $NaHCO_3$ aq. and 10 mL H_2O. Then it was extracted with DCM (10 mL × 3). The organic layer was combined and dried over Na_2SO_4. Then filtered and concentrated by rotary evaporation. The residue was purified by silica gel chromatography (EtOAc : petroleum ether = 1 : 50) to afford the product **51** as a colorless oil (231.5 mg, 70% yield). $\mathbf{R_f}$ = 0.6 (EtOAc : petroleum ether = 1 : 50). ^{1}**H NMR (400 MHz, Chloroform-d)** δ 7.17 – 6.96 (m, 7H), 6.89 – 6.81 (m, 1H), 4.15 (s, 2H), 3.36 (t, J = 6.5 Hz, 2H), 3.31 (t, J = 6.5 Hz, 2H), 3.22 (dd, J = 7.5, 5.0 Hz, 2H), 3.07 (dd, J = 7.4, 5.0 Hz, 2H), 2.05 – 1.94 (m, 2H) ppm. ^{13}**C NMR (101 MHz, CDCl3)** δ 149.80, 141.73, 137.38, 136.97, 131.15, 129.70, 128.86, 127.27, 126.82, 125.98, 122.62, 120.01, 61.50, 51.59, 34.61, 33.49, 31.69, 31.13 ppm. **HRMS** (ESI-TOF) m/z calcd for $C_{18}H_{21}NBr$ $(M + H)^+$: 330.0857, found 330.0858.

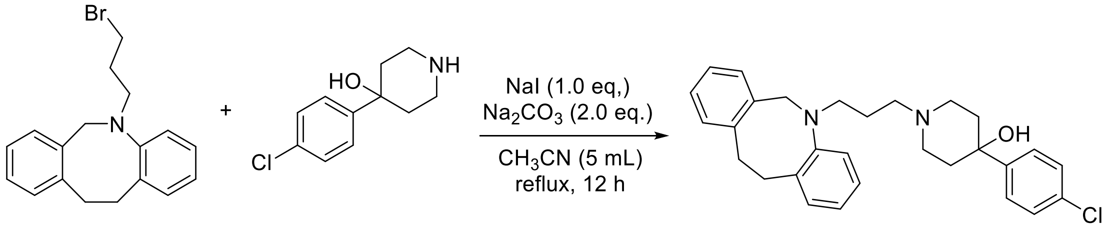

51 52 54

Supplementary Fig 17. Synthesis of compound **54**

In an oven dried 50 mL Schlenk tube, which containing a stirring bar, was charged with 4-(4-chlorophenyl)piperidin-4-ol **52** (0.6 mmol, 127.0 mg, 1.2 equiv.),

NaI (0.5 mmol, 74.9 mg, 1.0 equiv.) and Na_2CO_3 (1.0 mmol, 106.0 mg, 2.0 equiv.). The tube was then evacuated and bak-filled under N_2 flow (this sequence was repeated three times). CH_3CN (5.0 mL) and 5-(3-bromopropyl)-5,6,11,12-tetrahydrodibenzo[b,f]azocine **51** (0.5 mmol, 165.1 mg, 1.0 equiv.) were added. Heat to 85 ^oC and reflux for 12 hours. The reaction was quenched with 10 mL saturated $NaHCO_3$ aq. and 10 mL H_2O. Then it was extracted with DCM (10 mL × 3). The organic layer was combined and dried over Na_2SO_4. Then filtered and concentrated by rotary evaporation. The residue was purified by silica gel chromatography (EtOAc : petroleum ether = 1 : 5) to afford the product **54** as a white solid (151.2 mg, 66% yield). Analytical data (^{1}H NMR, ^{13}C NMR) matches with the literature.[23] **R_f** = 0.4 (EtOAc : petroleum ether = 1 : 5). **^{1}H NMR (400 MHz, Chloroform-d)** δ 7.38 7.41 – 7.35 (m, 2H), 7.21 – 7.16 (m, 2H), 7.14 – 7.09 (m, 3H), 7.08 – 6.96 (m, 4H), 6.89 – 6.84 (m, 1H), 4.11 (s, 2H), 3.27 (t, *J* = 6.2 Hz, 2H), 3.17 (d, *J* = 6.7 Hz, 2H), 3.05 (d, *J* = 7.0 Hz, 2H), 2.82 (d, *J* = 8.5 Hz, 4H), 2.65 – 2.53 (m, 2H), 2.44 – 2.30 (m, 2H), 1.95 – 1.84 (m, 2H), 1.77 (d, *J* = 13.8 Hz, 2H) ppm. **^{13}C NMR (101 MHz, CDCl3)** δ 150.08, 145.59, 141.94, 137.68, 136.51, 132.98, 131.38, 130.03, 128.96, 128.39, 127.55, 127.09, 126.34, 126.29, 122.80, 119.59, 69.12, 61.35, 55.72, 51.21, 49.01, 35.65, 34.51, 33.59, 23.28 ppm. **HRMS** (ESI-TOF) m/z calcd for $C_{29}H_{34}N_2OCl$ $(M + H)^+$: 461.2360, found 461.2366.

6.3 The attempt to convert Estrone 3-methyl ether

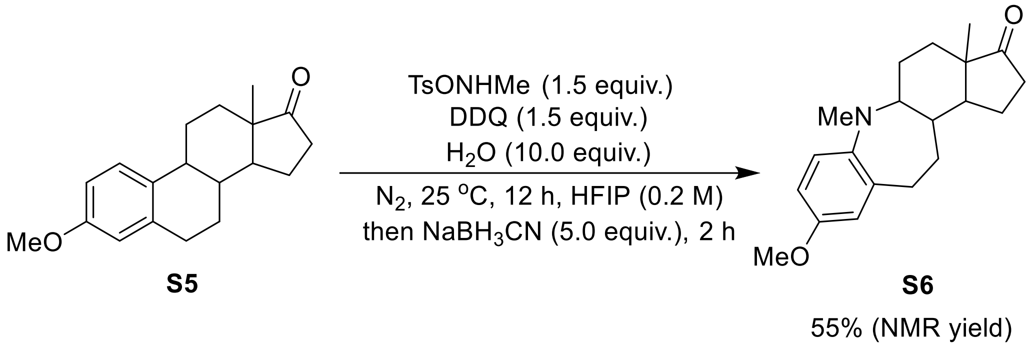

Supplementary Fig 18. The attempt to convert Estrone 3-methyl ether

In an oven dried 50 mL Schlenk tube, which containing a stirring bar, was charged with Estrone 3-methyl ether **S5** (0.3 mmol, 142.2 mg, 1.0 equiv.), TsONHMe (0.45 mmol, 90.0 mg, 1.5 equiv.) and DDQ (0.45 mmol, 103 mg, 1.5 equiv.). The tube was then evacuated and bak-filled under N_2 flow (this sequence was repeated three times). HFIP (1.5 mL) and H_2O (0.054 mL, 10.0 equiv.) were added and stirred at room temperature for 12 h. Then $NaBH_3CN$ (1.5 mmol, 96 mg, 5.0 equiv.) was added to the above reaction mixture and the reaction was stirred for 2 h at room temperature. The reaction was quenched with 2.0 mL saturated $NaHCO_3$ aq. and 3.0 mL H_2O. Then it was extracted with DCM (3.0 mL × 3). The organic layer was combined and dried over Na_2SO_4. Then filtered and concentrated by rotary evaporation. The yield was determined by ^{1}H NMR using dibromomethane (0.3 mmol) as the internal standard. The residue was purified by silica gel chromatography (DCM : EtOAc = 2 : 1) to afford the product (mixed with some inseparable by-product).

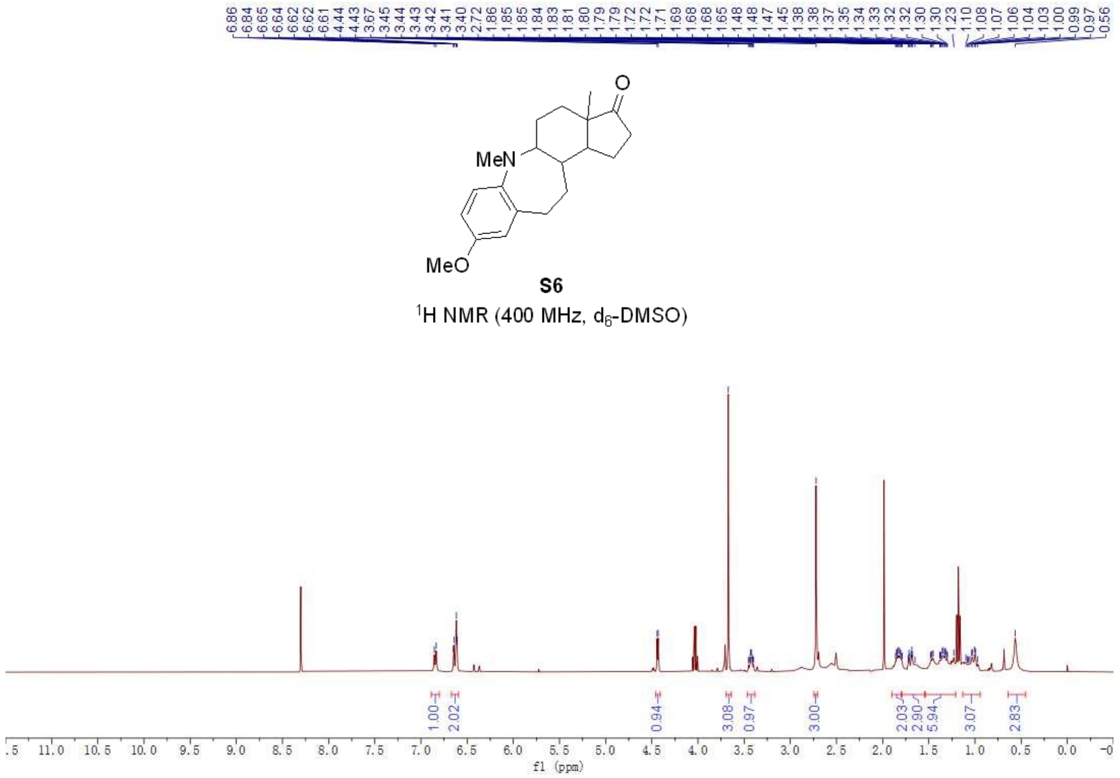

Supplementary Fig 19. The ^{1}H NMR spectrum of **S6**

6.4 The attempt of using one-pot reaction to synthesize quinoline

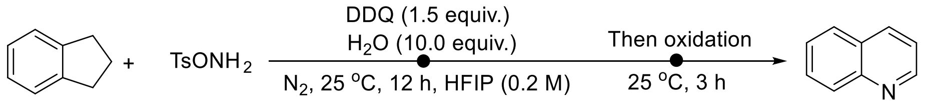

Entry	oxidation condition	Yield (%)
1	Pt/C (0.1 equiv.), air	0
2	H_2O_2 (30 wt% in H_2O, 5.0 equiv.)	0
3	$KMnO_4$ (5.0 equiv.)	26

Supplementary Table 7: The attempt of using one-pot reaction to synthesize quinoline

Entry 1: In an oven dried 50 mL Schlenk tube, which containing a stirring bar, was charged with O-tosylhydroxylamine (0.45 mmol, 84.3 mg, 1.5 equiv.) and DDQ (0.45 mmol, 103 mg, 1.5 equiv.). The tube was then evacuated and bak-filled under N_2 flow (this sequence was repeated three times). Indan (0.3 mmol, 35.4 mg, 1.0 equiv.), HFIP (1.5 mL) and H_2O (0.054 mL, 10.0 equiv.) were added and stirred at room temperature for 12 h. Then Pt/C (10 wt%, 0.03 mmol, 31.9 mg, 0.1 equiv.) was added to the above reaction mixture and the reaction was stirred for 3 h in air at room temperature. The reaction was quenched with 2.0 mL saturated $NaHCO_3$ aq. and 3.0 mL H_2O. Then it was extracted with DCM (3.0 mL × 3). The organic layer was combined and dried over Na_2SO_4. Then filtered and concentrated by rotary evaporation. No target product detected in the system.

Entry 2: In an oven dried 50 mL Schlenk tube, which containing a stirring bar, was charged with O-tosylhydroxylamine (0.45 mmol, 84.3 mg, 1.5 equiv.) and DDQ (0.45 mmol, 103 mg, 1.5 equiv.). The tube was then evacuated and bak-filled under N_2 flow (this sequence was repeated three times). Indan (0.3 mmol, 35.4 mg, 1.0 equiv.), HFIP (1.5 mL) and H_2O (0.054 mL, 10.0 equiv.) were added and stirred at room temperature for 12 h. Then H_2O_2 (30 wt%, 1.5 mmol, 170.1 mg, 5.0 equiv.) was added to the above reaction mixture and the reaction was stirred for 3 h at room temperature. The reaction was quenched with 2.0 mL saturated $NaHCO_3$ aq. and 3.0 mL H_2O. Then it was extracted with DCM (3.0 mL × 3). The organic layer was combined and dried over Na_2SO_4. Then filtered and concentrated by rotary evaporation. No target product detected in the system.

Entry 3: In an oven dried 50 mL Schlenk tube, which containing a stirring bar, was charged with O-tosylhydroxylamine (0.45 mmol, 84.3 mg, 1.5 equiv.) and DDQ (0.45 mmol, 103 mg, 1.5 equiv.). The tube was then evacuated and bak-filled under N_2 flow (this sequence was repeated three times). Indan (0.3 mmol, 35.4 mg, 1.0 equiv.), HFIP (1.5 mL) and H_2O (0.054 mL, 10.0 equiv.) were added and stirred at room temperature for 12 h. Then $KMnO_4$ (1.5 mmol, 273.1 mg, 5.0 equiv.) was added to the above reaction mixture and the reaction was stirred for 3 h at room temperature. The reaction was quenched with 2.0 mL saturated $NaHCO_3$ aq. and 3.0 mL H_2O. Then it was extracted with DCM (3.0 mL × 3). The organic layer was combined and dried

over Na_2SO_4. Then filtered and concentrated by rotary evaporation. The residue was purified by silica gel chromatography (EtOAc : petroleum ether = 1 : 1) to afford the product quinoline as a yellow oil (10.1 mg, 26% yield). **R_f** = 0.4 (EtOAc : petroleum ether = 1 : 1). **^{1}H NMR (400 MHz, Chloroform-d)** δ 8.95 – 8.88 (m, 1H), 8.20 – 8.08 (m, 2H), 7.86 – 7.80 (m, 1H), 7.76 – 7.69 (m, 1H), 7.59 – 7.52 (m, 1H), 7.44 – 7.37 (m, 1H) ppm. **^{13}C NMR (101 MHz, CDCl3)** δ 150.33, 148.19, 136.13, 129.48, 129.36, 128.29, 127.77, 126.56, 121.06 ppm.

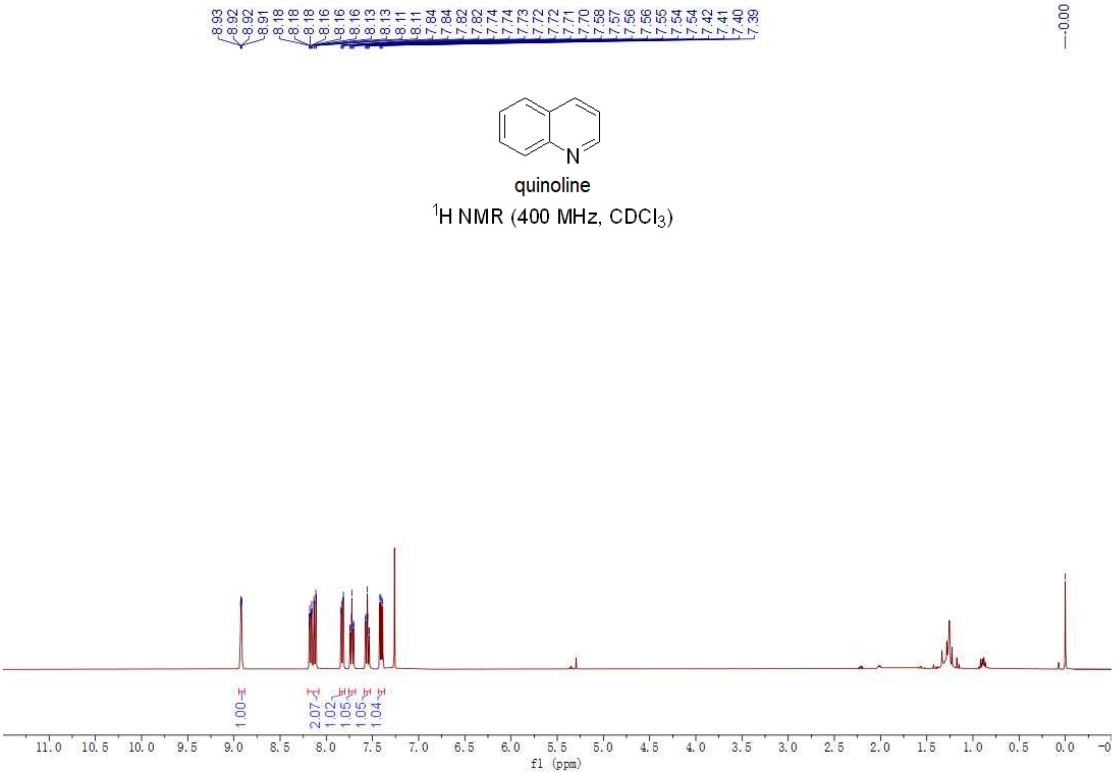

Supplementary Fig 20. The ^{1}H NMR spectrum of quinoline

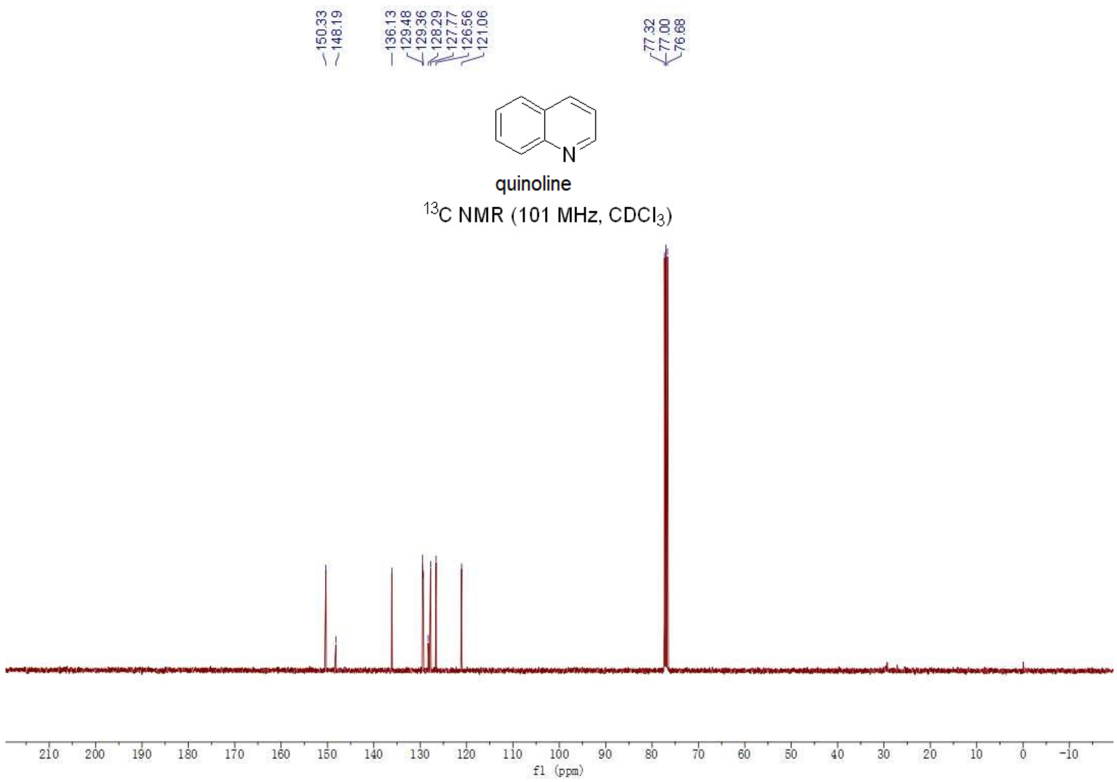

Supplementary Fig 21. The ^{13}C NMR spectrum of quinoline

6.5 The attempt of using azide as animation reagent:

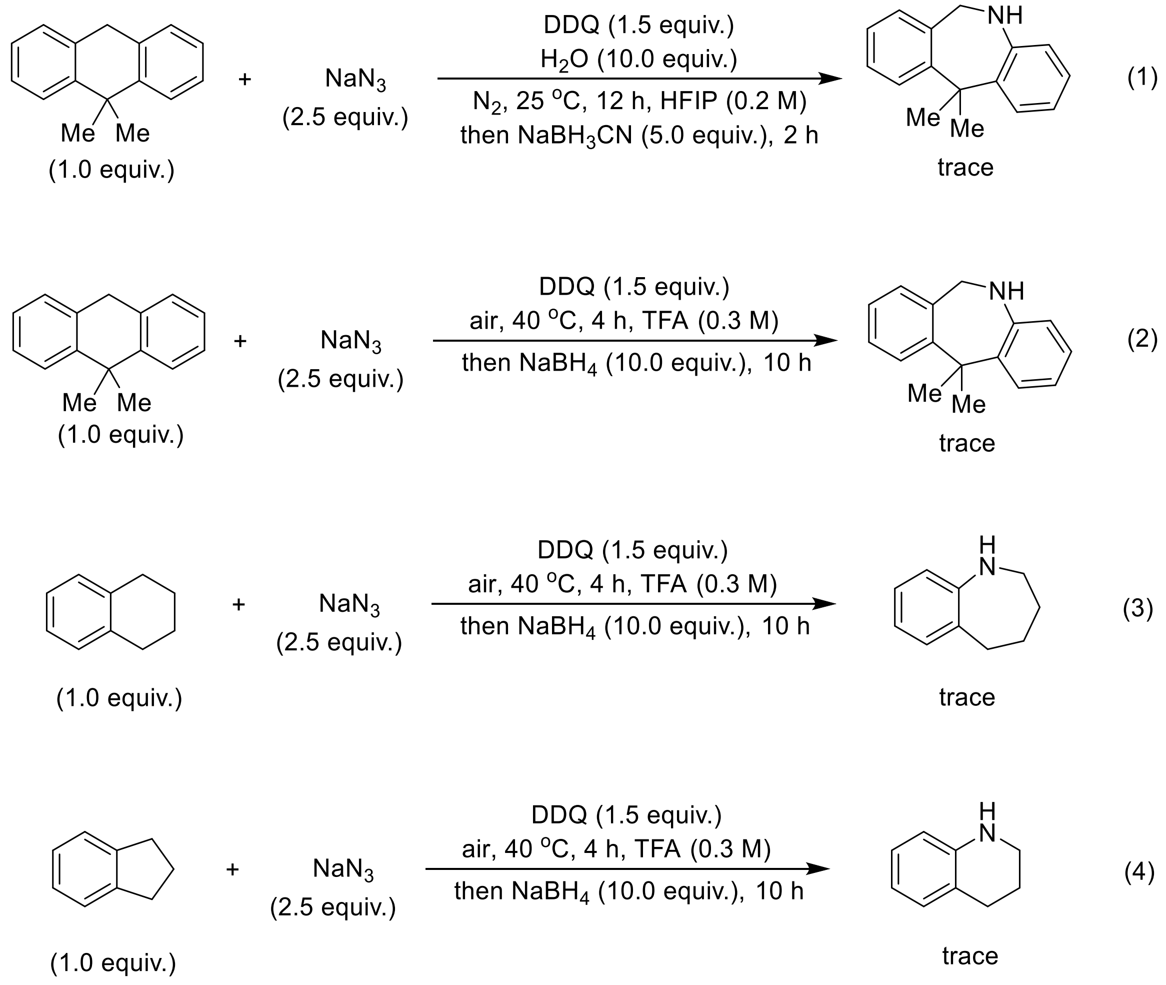

Supplementary Fig 22. The attempt of using NaN_3 as animation reagent

Reaction (1): In an oven dried 50 mL Schlenk tube, which containing a stirring bar, was charged with 9,9-dimethyl-9,10-dihydroanthracene (62.5 mg, 0.30 mmol, 1.0 equiv.), NaN_3 (0.75 mmol, 49.0 mg, 2.5 equiv.) and DDQ (0.45 mmol, 103 mg, 1.5 equiv.). The tube was then evacuated and bak-filled under N_2 flow (this sequence was repeated three times). HFIP (1.5 mL) and H_2O (0.054 mL, 10.0 equiv.) were added and stirred at room temperature for 12 h. Then $NaBH_3CN$ (1.5 mmol, 96 mg, 5.0 equiv.) was added to the above reaction mixture and the reaction was stirred for 2 h at room temperature. The reaction was quenched with 2.0 mL saturated $NaHCO_3$ aq. and 3.0 mL H_2O. Then it was extracted with DCM (3.0 mL × 3). The organic layer was combined and dried over Na_2SO_4. Then filtered and concentrated by rotary evaporation. The yield was determined by ^{1}H NMR using dibromomethane (0.3 mmol) as the internal standard, and only trace target product detected in the system.

Reaction (2): This reaction condition is from Ref. 24. The reaction of 9,9-dimethyl-9,10-dihydroanthracene (62.5 mg, 0.30 mmol, 1.0 equiv.), NaN_3 (49 mg, 0.75 mmol), DDQ (102 mg, 0.45 mmol) in TFA (1.0 mL) under air at 40 ^oC for 4 h. Then $NaBH_4$ (113 mg, 3.0 mmol) was added and stirred for 10 h. After cooling down to room temperature, quenched by 2 M NaOH (5 mL), extracted by EA, washed with

brine and dried over Na_2SO_4. The yield was determined by ^1H NMR using dibromomethane (0.3 mmol) as the internal standard, and only trace target product detected in the system.

Reaction (3): This reaction condition is from Ref. 24. The reaction of indane (35.5 mg, 0.30 mmol, 1.0 equiv.), NaN_3 (49 mg, 0.75 mmol), DDQ (102 mg, 0.45 mmol) in TFA (1.0 mL) under air at 40 °C for 4 h. Then $NaBH_4$ (113 mg, 3.0 mmol) was added and stirred for 10 h. After cooling down to room temperature, quenched by 2 M NaOH (5 mL), extracted by EA, washed with brine and dried over Na_2SO_4. The yield was determined by ^1H NMR using dibromomethane (0.3 mmol) as the internal standard, and only trace target product detected in the system.

Reaction (4): This reaction condition is from Ref. 24. The reaction of tetralin (39.7 mg, 0.30 mmol, 1.0 equiv.), NaN_3 (49 mg, 0.75 mmol), DDQ (102 mg, 0.45 mmol) in TFA (1.0 mL) under air at 40 °C for 4 h. Then $NaBH_4$ (113 mg, 3.0 mmol) was added and stirred for 10 h. After cooling down to room temperature, quenched by 2 M NaOH (5 mL), extracted by EA, washed with brine and dried over Na_2SO_4. The yield was determined by ^1H NMR using dibromomethane (0.3 mmol) as the internal standard, and only trace target product detected in the system.

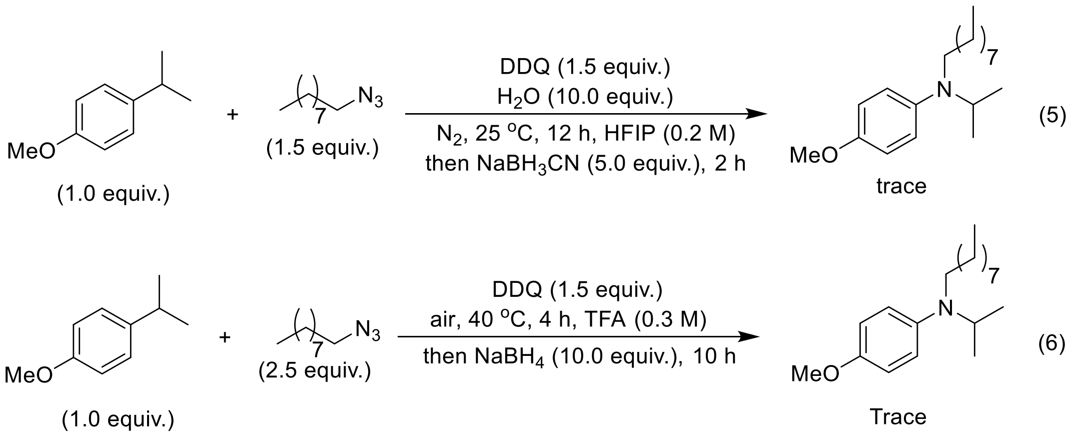

Supplementary Fig 23. The attempt of using alkyl azide as animation reagent

Reaction (5): In an oven dried 50 mL Schlenk tube, which containing a stirring bar, was charged with 1-azidononane (0.45 mmol, 76.17 mg, 1.5 equiv.) and DDQ (0.45 mmol, 103 mg, 1.5 equiv.). The tube was then evacuated and bak-filled under N_2 flow (this sequence was repeated three times). 1-isopropyl-4-methoxybenzene (0.3 mmol, 45.1 mg, 1.0 equiv.), HFIP (1.5 mL) and H_2O (0.054 mL, 10.0 equiv.) were added and stirred at room temperature for 12 h. Then $NaBH_3CN$ (1.5 mmol, 96 mg, 5.0 equiv.) was added to the above reaction mixture and the reaction was stirred for 2 h at room temperature. The reaction was quenched with 2.0 mL saturated $NaHCO_3$ aq. and 3.0 mL H_2O. Then it was extracted with DCM (3.0 mL × 3). The organic layer was combined and dried over Na_2SO_4. Then filtered and concentrated by rotary evaporation. The yield was determined by ^1H NMR using dibromomethane (0.3 mmol) as the internal standard, and only trace target product detected in the system.

Reaction (6): This reaction condition is from Ref. 24. The reaction of 1-isopropyl-4-methoxybenzene (0.3 mmol, 45.1 mg, 1.0 equiv.), 1-azidononane (126.95 mg, 0.75 mmol), DDQ (102 mg, 0.45 mmol) in TFA (1.0 mL) under air at 40 ^{o}C for 4 h. Then $NaBH_4$ (113 mg, 3.0 mmol) was added and stirred for 10 h. After cooling down to room temperature, quenched by 2 M NaOH (5 mL), extracted by EA, washed with brine and dried over Na_2SO_4. The yield was determined by ^{1}H NMR using dibromomethane (0.3 mmol) as the internal standard, and only trace target product detected in the system.

6.6 Synthesis of N-isopropyl-4-methoxyaniline:

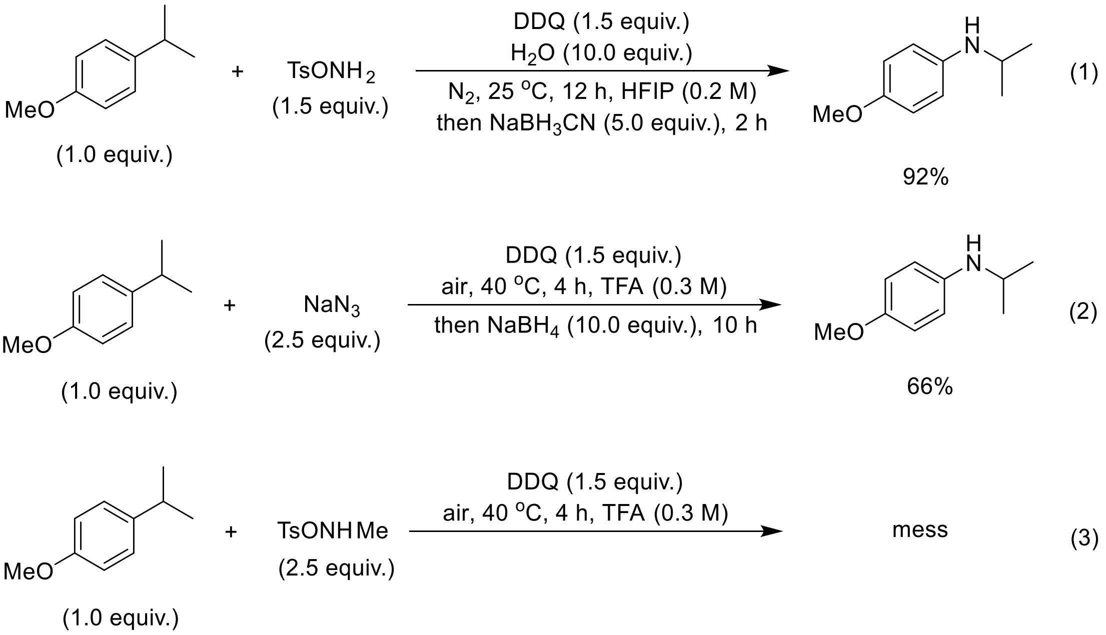

Supplementary Fig 24. Synthesis of N-isopropyl-4-methoxyaniline

Reaction (1): In an oven dried 50 mL Schlenk tube, which containing a stirring bar, was charged with $TsONH_2$ (0.45 mmol, 84.3 mg, 1.5 equiv.) and DDQ (0.45 mmol, 103 mg, 1.5 equiv.). The tube was then evacuated and bak-filled under N_2 flow (this sequence was repeated three times). 1-isopropyl-4-methoxybenzene (0.3 mmol, 45.1 mg, 1.0 equiv.), HFIP (1.5 mL) and H_2O (0.054 mL, 10.0 equiv.) were added and stirred at room temperature for 12 h. Then $NaBH_3CN$ (1.5 mmol, 96 mg, 5.0 equiv.) was added to the above reaction mixture and the reaction was stirred for 2 h at room temperature. The reaction was quenched with 2.0 mL saturated $NaHCO_3$ aq. and 3.0 mL H_2O. Then it was extracted with DCM (3.0 mL × 3). The organic layer was combined and dried over Na_2SO_4. Then filtered and concentrated by rotary evaporation. The residue was purified by silica gel chromatography (EtOAc : petroleum ether = 1 : 5) to afford the product as a reddish brown oil (45.4 mg, 92% yield).

Reaction (2): This reaction condition is from Ref. 24. The reaction of 1-isopropyl-4-methoxybenzene (0.3 mmol, 45.1 mg, 1.0 equiv.), NaN_3 (49 mg, 0.75 mmol), DDQ (102 mg, 0.45 mmol) in TFA (1.0 mL) under air at 40 ^{o}C for 4 h. Then $NaBH_4$ (113 mg, 3.0 mmol) was added and stirred for 10 h. After cooling down to room temperature, quenched by 2 M NaOH (5 mL), extracted by EA, washed with brine and dried over Na_2SO_4. Then filtered and concentrated by rotary evaporation. The residue was purified by silica gel chromatography (EtOAc : petroleum ether = 1 : 5) to afford the product as a reddish brown oil (32.8 mg, 66% yield).

Reaction (3): This reaction condition is from Ref. 24. The reaction of 1-isopropyl-4-methoxybenzene (0.3 mmol, 45.1 mg, 1.0 equiv.), TsONHMe (150.93 mg, 0.75 mmol), DDQ (102 mg, 0.45 mmol) in TFA (1.0 mL) under air at 40 ^{o}C for 4 h. After cooling down to room temperature, quenched by 2 M NaOH (5 mL), extracted by EA, washed with brine and dried over Na_2SO_4. Then filtered and

concentrated by rotary evaporation. The yield was determined by ^{1}H NMR using dibromomethane (0.3 mmol) as the internal standard, and only trace target product detected in the system.

Analytical data (^{1}H NMR, ^{13}C NMR) matches with the literature.[25] **R_f** = 0.6 (EtOAc : petroleum ether = 1 : 5). **^{1}H NMR (400 MHz, Chloroform-d)** δ 6.73 – 6.66 (m, 2H), 6.52 – 6.45 (m, 2H), 3.66 (s, 3H), 3.52 – 3.41 (m, 1H), 3.15 (brs, 1H), 1.10 (d, J = 6.3 Hz, 6H) ppm. **^{13}C NMR (101 MHz, CDCl3)** δ 151.93, 141.71, 114.93, 114.89, 55.76, 45.23, 23.04 ppm.

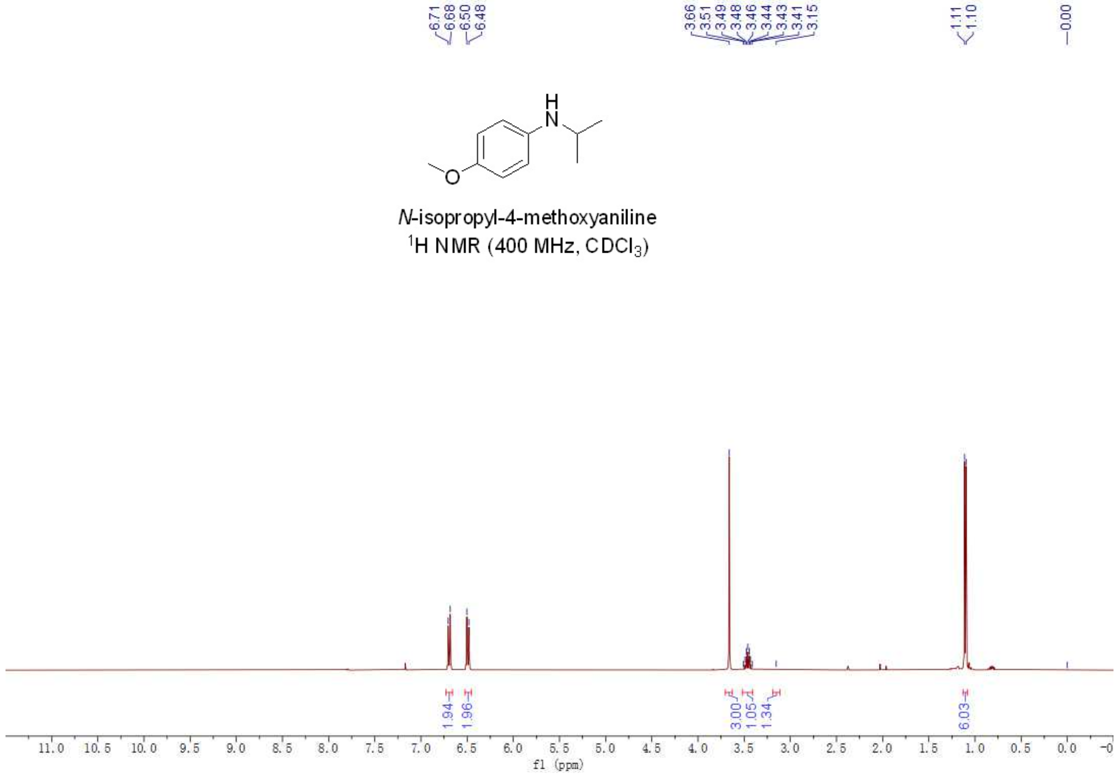

Supplementary Fig 25. The ^{1}H NMR spectrum of N-isopropyl-4-methoxyaniline

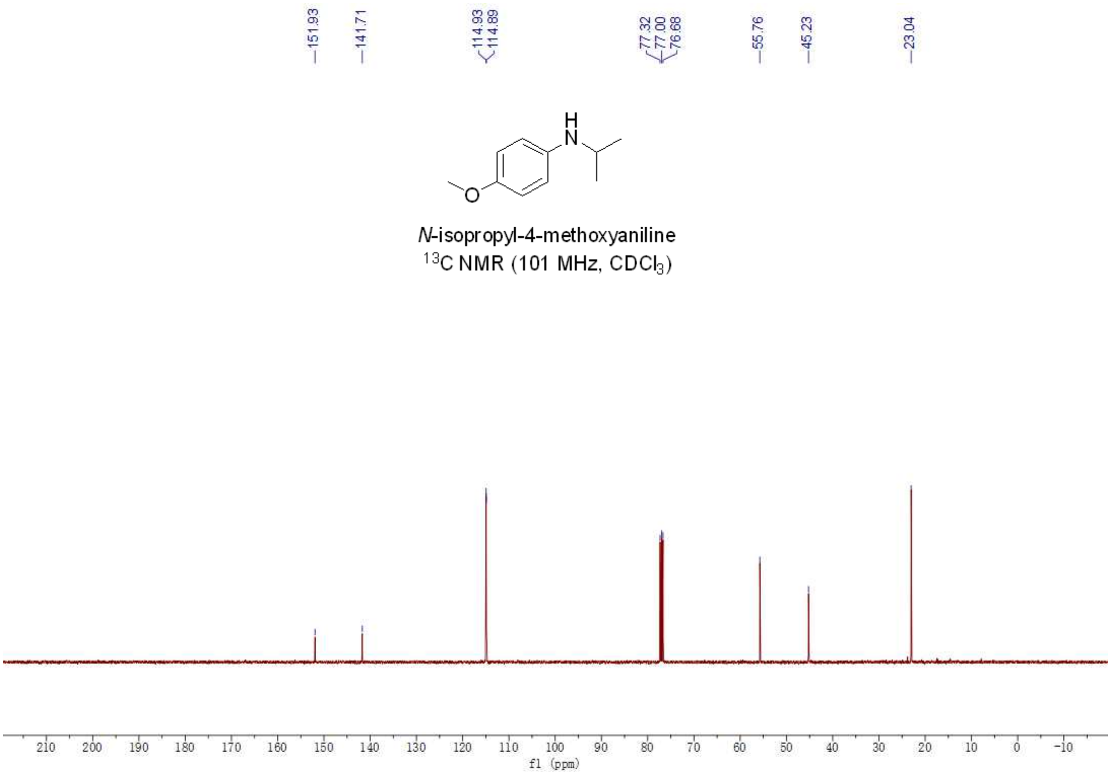

Supplementary Fig 26. The ^{13}C NMR spectrum of N-isopropyl-4-methoxyaniline

6.7 Synthesis of N-isopropyl-4-methoxy-N-methylaniline 35 using early N-alkylation or late N-alkylation:

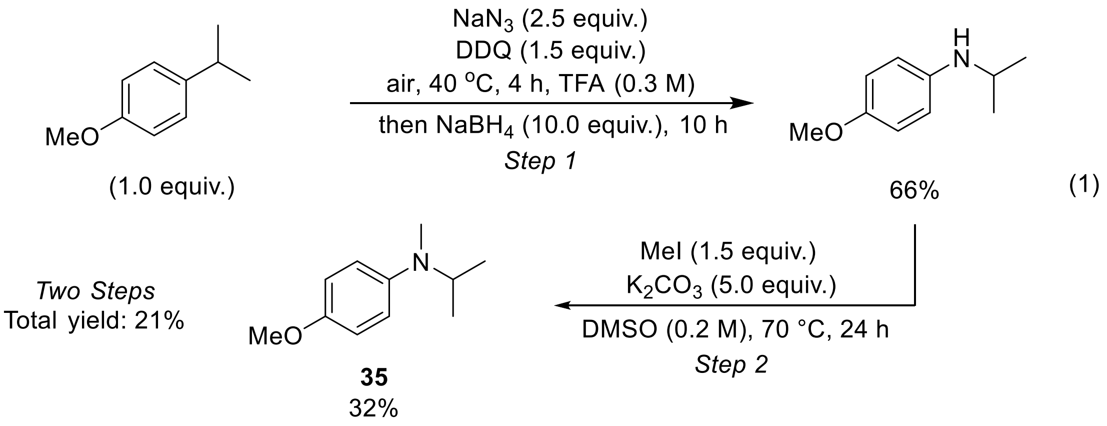

Supplementary Fig 27. Synthesis of N-isopropyl-4-methoxy-N-methylaniline 35 using early N-alkylation

Synthesis path 1: *Step 1*: This reaction condition is from Ref. 24. The reaction of 1-isopropyl-4-methoxybenzene (0.3 mmol, 45.1 mg, 1.0 equiv.), NaN_3 (49 mg, 0.75 mmol), DDQ (102 mg, 0.45 mmol) in TFA (1.0 mL) under air at 40 ^{o}C for 4 h. Then $NaBH_4$ (113 mg, 3.0 mmol) was added and stirred for 10 h. After cooling down to room temperature, quenched by 2 M NaOH (5 mL), extracted by EA, washed with brine and dried over Na_2SO_4. Then filtered and concentrated by rotary evaporation. The residue was purified by silica gel chromatography (EtOAc : petroleum ether = 1 : 5) to afford the product as a reddish brown oil (32.8 mg, 66% yield).

Step 2: In an oven dried 50 mL Schlenk tube, which containing a stirring bar, was charged with K_2CO_3 (1.5 mmol, 207.32 mg, 5.0 equiv.). N-isopropyl-4-methoxyaniline (0.3 mmol, 49.3 mg, 1.0 equiv.), MeI (0.45 mmol, 63.9 mg, 1.5 equiv.) and DMSO (1.5 mL) were added and stirred at 70 ^{o}C for 24 h. The reaction was quenched with 2.0 mL saturated NH_4Cl aq. and 3.0 mL H_2O. Then it was extracted with DCM (3.0 mL × 3). The organic layer was combined and dried over Na_2SO_4. Then filtered and concentrated by rotary evaporation. The residue was purified by silica gel chromatography (EtOAc : petroleum ether = 1 : 10) to afford the product **35** as a yellow oil (17.1 mg, 32% yield). The total yield is 21%.

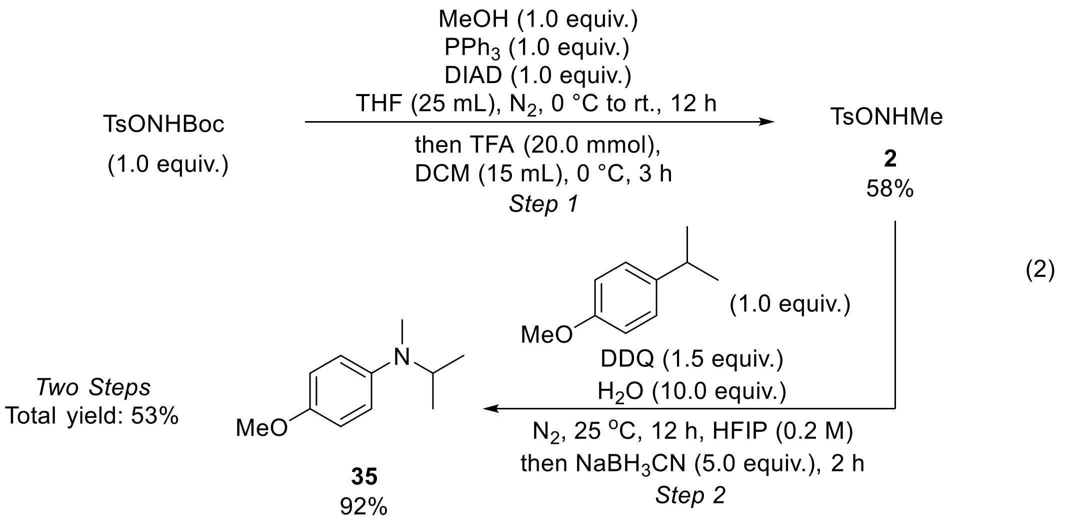

Supplementary Fig 28. Synthesis of N-isopropyl-4-methoxy-N-methylaniline 35 using late N-alkylation

Synthesis path 1: *Step 1*: In an oven dried 50 mL round bottom flask, which equipped with a stir bar was added PPh_3 (3.14g, 12.0 mmol, 1.2 equiv.). The flask was then evacuated and back-filled under N_2 flow (this sequence was repeated three times). Dry THF (12.0 mL) and DIAD (2.4 mL, 12.0 mmol, 1.2 equiv.) was added sequentially at 0 ^{o}C under nitrogen atmosphere. A white solid was formed quickly, and then it's stirred for 30 min at 0 ^{o}C. Then the solution of MeOH (320.4 mg, 10.0 mmol, 1.0 equiv.) in THF (4.0 mL) and TsONHBoc (2.87g, 10.0 mmol, 1.0 equiv.) in THF (4.0 mL) were added to the above mixture successively. Then the reaction was stirred at 0 ^{o}C for another 2 h, the resulting mixture was then warmed to 25 ^{o}C (oil bath) and stirred overnight (monitored by TLC). The reaction mixture diluted with 10.0 mL H_2O. Then it was extracted with DCM (10.0 mL × 3). The organic layer was combined and dried over anhydrous Na_2SO_4. Then filtered and concentrated by rotary evaporation. The residue was purified by silica gel chromatography (EtOAc/petroleum ether= 1:10) to afford the TsONBocR (containing a small amount of DIAD, directly feeding the next step). To a solution of N-Boc-N-alkyl-O-tosyl hydroxylamine (1.0 equiv.) in DCM (1.0 M) was added TFA (20.0 equiv.) at 0 ^{o}C, then it was stirred at 0 ^{o}C overnight (monitored by TLC). The reaction was quenched with cold water (10.0 mL) at 0 ^{o}C and then extracted with DCM (10.0 mL × 3). The organic layer was combined and dried over anhydrous Na_2SO_4. Then filtered and concentrated by rotary evaporation. The residue was purified by silica gel chromatography (EtOAc/petroleum ether= 1:5) to afford the product **2** as a white solid (1.1689 g, 58% yield).

Step 2: In an oven dried 25 mL Schlenk tube, which containing a stirring bar, was charged with N-methyl-O-tosylhydroxylamine (**2**, 90.0 mg, 0.45 mmol, 1.5 equiv.), DDQ (0.45 mmol, 102 mg, 1.5 equiv.). The tube was then evacuated and back-filled under N_2 flow (this sequence was repeated three times). H_2O (3.0 mmol, 0.054 mL, 10.0 equiv.), HFIP (1.5 mL) and 1-isopropyl-4-methoxybenzene **55** (0.3 mmol, 45.1 mg, 1.0 equiv.) were added and stirred at room temperature for 12 h. Then $NaBH_3CN$ (1.5 mmol, 96 mg, 5.0 equiv.) was added to the above reaction mixture and

the reaction was stirred for 2 h at room temperature. The reaction was quenched with 2.0 mL saturated $NaHCO_3$ aq. and 3.0 mL H_2O. Then it was extracted with DCM (3.0 mL × 3). The organic layer was combined and dried over Na_2SO_4. Then filtered and concentrated by rotary evaporation. The residue was purified by silica gel chromatography (EtOAc/petroleum ether = 1: 10) to afford the product **35** (49.2 mg, 92% yield). The total yield is 53%.

Supplementary Table 8: Some examples of low yield and non-reactive substrates.

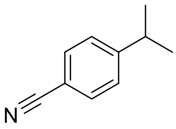	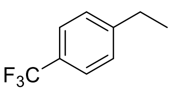	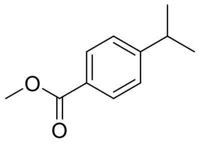
NR.	NR.	NR.
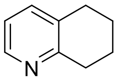	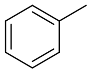	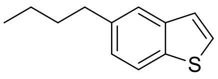
NR.	NR.	Trace

7. Mechanistic studies

7.1 Deuterium experiments:

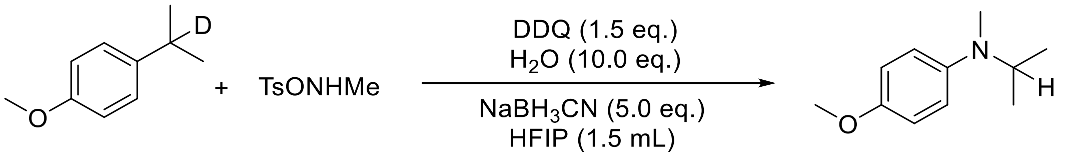

55-D **2** **35**

Supplementary Fig 29. Deuterium experiment (1)

In an oven dried 25 mL Schlenk tube, which containing a stirring bar, was charged with N-methyl-O-tosylhydroxylamine (**2**, 90.0 mg, 0.45 mmol, 1.5 equiv.), DDQ (0.45 mmol, 102 mg, 1.5 equiv.). The tube was then evacuated and back-filled under N_2 flow (this sequence was repeated three times). H_2O (3.0 mmol, 0.054 mL, 10.0 equiv.), HFIP (1.5 mL) and 1-methoxy-4-(propan-2-yl-2-d)benzene **55-D** (0.3 mmol, 45.2 mg, 1.0 equiv.) were added and stirred at room temperature for 12 h. Then $NaBH_3CN$ (1.5 mmol, 96 mg, 5.0 equiv.) was added to the above reaction mixture and the reaction was stirred for 2 h at room temperature. The reaction was quenched with 2.0 mL saturated $NaHCO_3$ aq. and 3.0 mL H_2O. Then it was extracted with DCM (3.0 mL × 3). The organic layer was combined and dried over Na_2SO_4. Then filtered and concentrated by rotary evaporation. The residue was purified by silica gel chromatography (EtOAc/petroleum ether = 1: 10) to afford the product **35** (48.5 mg, 90% yield, D% = 0).

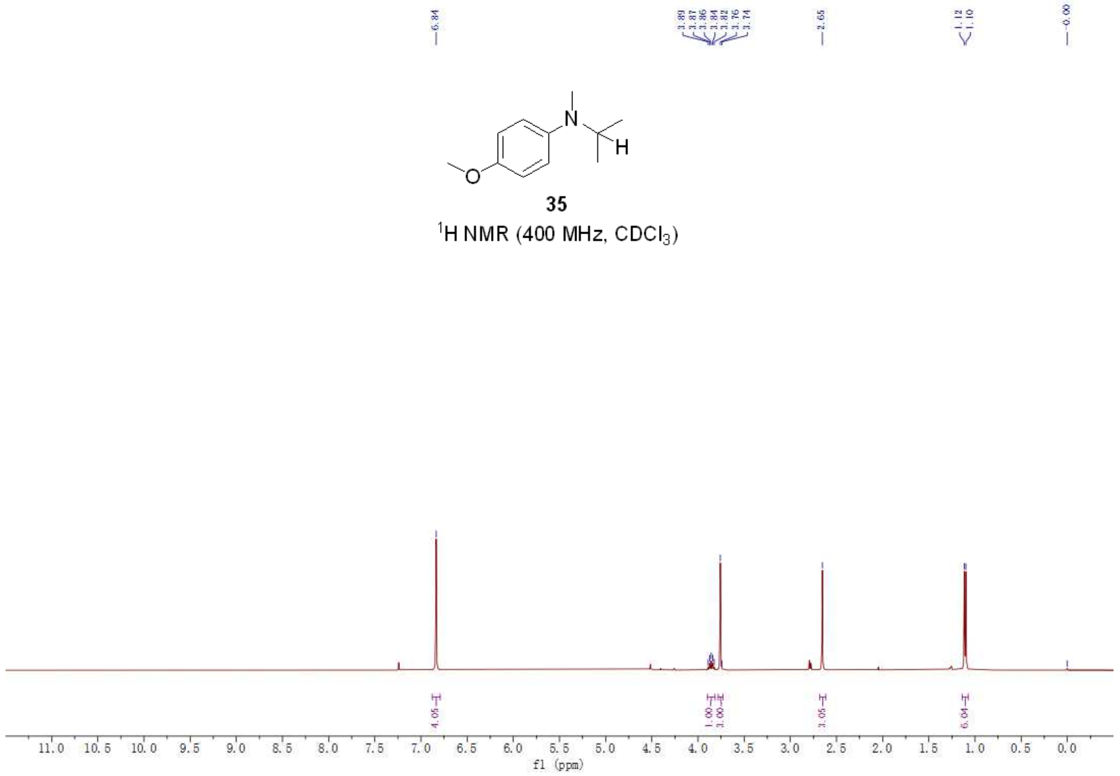

Supplementary Fig 30. The ^{1}H NMR spectrum of **35** from deuterium experiment (1)

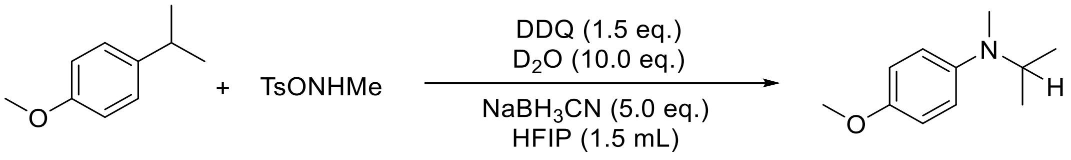

55 **2** **35**

Supplementary Fig 31. Deuterium experiment (2)

In an oven dried 25 mL Schlenk tube, which containing a stirring bar, was charged with N-methyl-O-tosylhydroxylamine (**2**, 90.0 mg, 0.45 mmol, 1.5 equiv.), DDQ (0.45 mmol, 102 mg, 1.5 equiv.). The tube was then evacuated and back-filled under N_2 flow (this sequence was repeated three times). D_2O (3.0 mmol, 0.054 mL, 10.0 equiv.), HFIP (1.5 mL) and 1-isopropyl-4-methoxybenzene **55** (0.3 mmol, 45.1 mg, 1.0 equiv.) were added and stirred at room temperature for 12 h. Then $NaBH_3CN$ (1.5 mmol, 96 mg, 5.0 equiv.) was added to the above reaction mixture and the reaction was stirred for 2 h at room temperature. The reaction was quenched with 2.0 mL saturated $NaHCO_3$ aq. and 3.0 mL H_2O. Then it was extracted with DCM (3.0 mL × 3). The organic layer was combined and dried over Na_2SO_4. Then filtered and concentrated by rotary evaporation. The residue was purified by silica gel chromatography (EtOAc/petroleum ether = 1: 10) to afford the product **35** (48.4 mg, 90% yield, D% = 0).

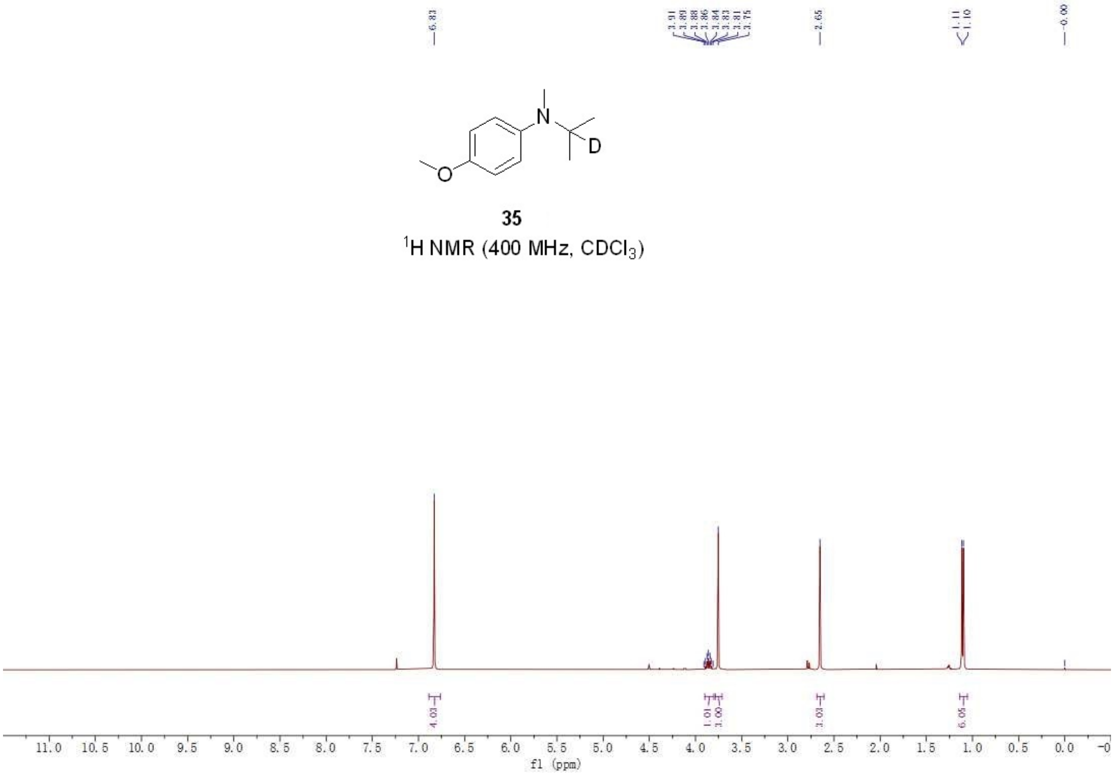

Supplementary Fig 32. The ^{1}H NMR spectrum of **35** from deuterium experiment (2)

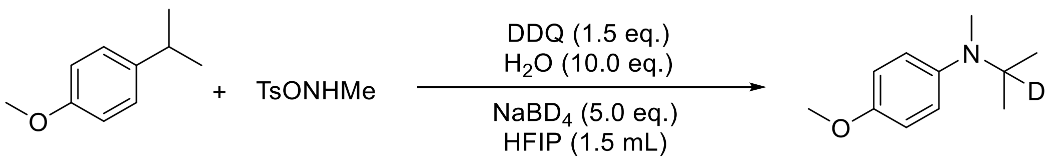

55 **2** **35-D**

Supplementary Fig 33. Deuterium experiment (3)

In an oven dried 25 mL Schlenk tube, which containing a stirring bar, was charged with N-methyl-O-tosylhydroxylamine (**2**, 90.0 mg, 0.45 mmol, 1.5 equiv.), DDQ (0.45 mmol, 102 mg, 1.5 equiv.). The tube was then evacuated and back-filled under N_2 flow (this sequence was repeated three times). H_2O (3.0 mmol, 0.054 mL, 10.0 equiv.), HFIP (1.5 mL) and 1-isopropyl-4-methoxybenzene **55** (0.3 mmol, 45.1 mg, 1.0 equiv.) were added and stirred at room temperature for 12 h. Then $NaBD_4$ (1.5 mmol, 62.5 mg, 5.0 equiv.) was added to the above reaction mixture and the reaction was stirred for 2 h at room temperature. The reaction was quenched with 2.0 mL saturated $NaHCO_3$ aq. and 3.0 mL H_2O. Then it was extracted with DCM (3.0 mL × 3). The organic layer was combined and dried over Na_2SO_4. Then filtered and concentrated by rotary evaporation. The residue was purified by silica gel chromatography (EtOAc/petroleum ether = 1: 10) to afford the product **35-D** (44.5 mg, 83%, D% = 95%).

^{1}H NMR (400 MHz, Chloroform-d) δ 6.82 (d, 4H), 3.75 (s, 3H), 2.65 (s, 3H), 1.10 (s, 6H) ppm. **HRMS** (ESI-TOF) m/z calcd for $DC_{11}H_{17}NO$: 181.1451, found 181.1460.

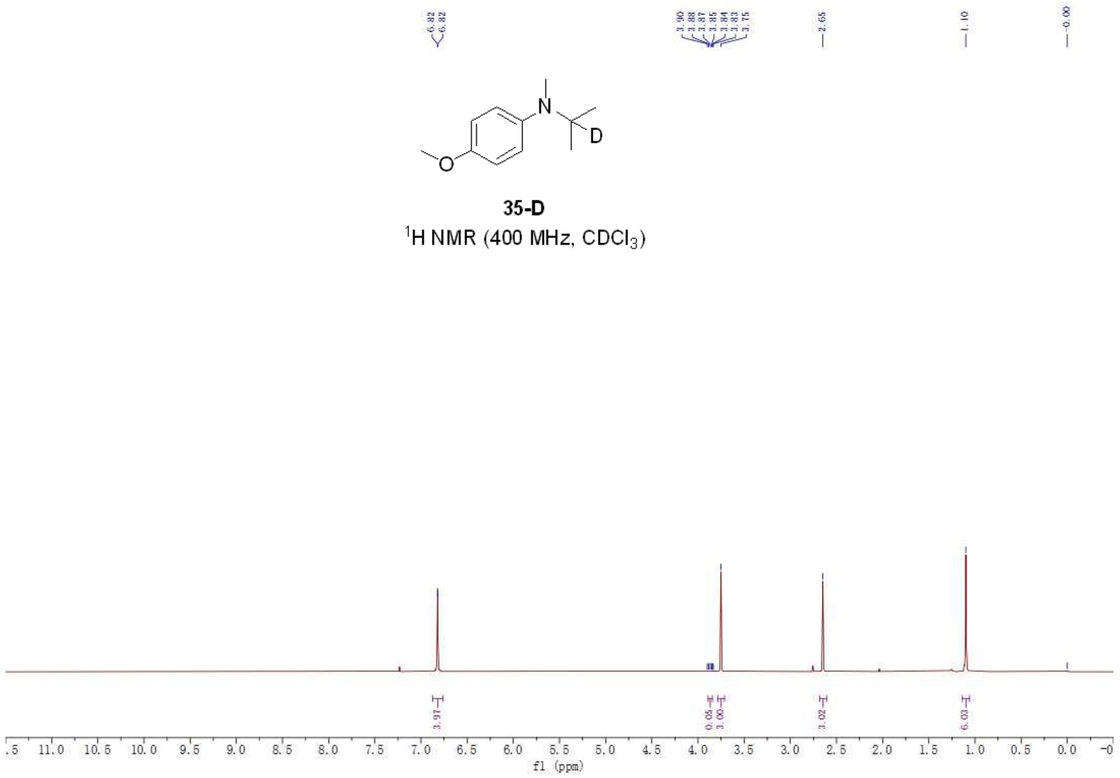

Supplementary Fig 34. The ^{1}H NMR spectrum of **35-D** from deuterium experiment (3)

7.2 Kinetic isotope experiments:

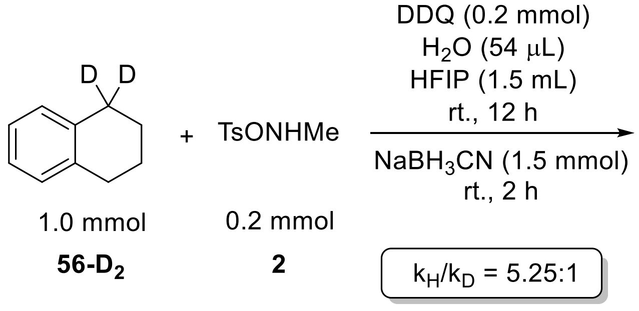

Supplementary Fig 35. Kinetic isotope experiments

In an oven dried 25 mL Schlenk tube, which containing a stirring bar, was charged with N-methyl-O-tosylhydroxylamine (**2**, 40.0 mg, 0.2 mmol, 1.5 equiv.), DDQ (0.45 mmol, 45.2 mg, 1.5 equiv.). The tube was then evacuated and back-filled under N_2 flow (this sequence was repeated three times). H_2O (3.0 mmol, 0.054 mL, 10.0 equiv.), HFIP (1.5 mL) and 1,2,3,4-tetrahydronaphthalene-1,1-d_2 **56-D$_2$** (0.3 mmol, 45.1 mg, 1.0 equiv.) were added and stirred at room temperature for 12 h. Then $NaBH_3CN$ (1.5 mmol, 62.5 mg, 5.0 equiv.) was added to the above reaction mixture and the reaction was stirred for 2 h at room temperature. The reaction was quenched with 2.0 mL saturated $NaHCO_3$ aq. and 3.0 mL H_2O. Then it was extracted with DCM (3.0 mL × 3). The organic layer was combined and dried over Na_2SO_4. Then filtered and concentrated by rotary evaporation. The residue was purified by silica gel chromatography (EtOAc/petroleum ether = 1: 50) to afford the product **19-D$_1$** and **19-D$_2$** (26.9 mg, 83% yield). The KIE of sp^3 C-H breakage: $K_H : K_D$ = **19-D$_1$** : **19-D$_2$** = 5.25 : 1 .

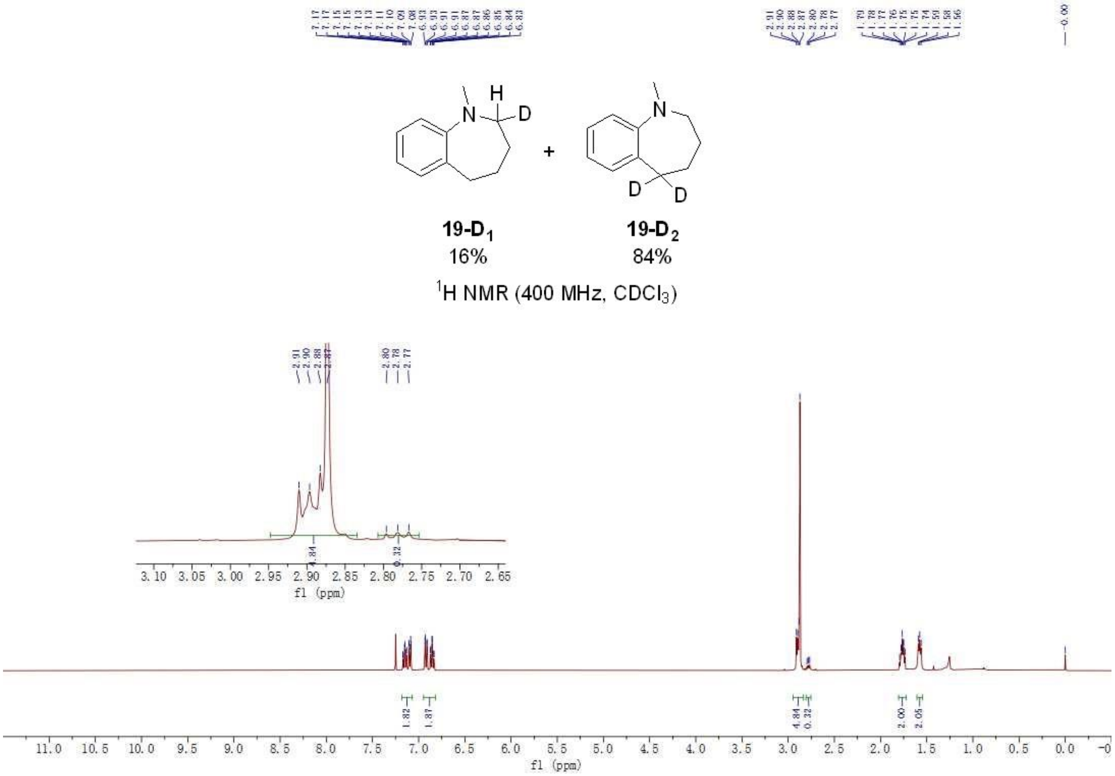

Supplementary Fig 36. The ^{1}H NMR spectrum of **19-D$_1$** and **19-D$_2$**

7.3 Functional groups cross competition experiments:

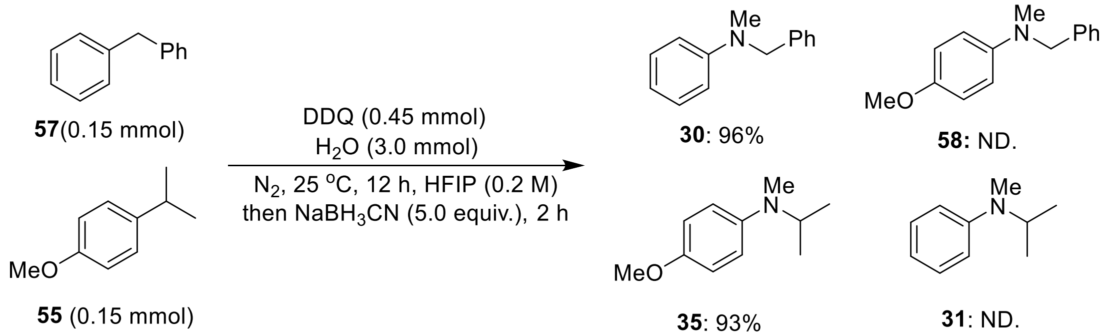

Supplementary Fig 37. Functional groups cross competition experiments

In an oven dried 25 mL Schlenk tube, which containing a stirring bar, was charged with N-methyl-O-tosylhydroxylamine (**2**, 90.0 mg, 0.45 mmol, 1.5 equiv.), DDQ (0.45 mmol, 102 mg, 1.5 equiv.). The tube was then evacuated and back-filled under N_2 flow (this sequence was repeated three times). H_2O (3.0 mmol, 0.054 mL, 10.0 equiv.), HFIP (1.5 mL), diphenylmethane **57** (0.15 mmol, 25.2 mg, 0.5 equiv.) 1-isopropyl-4-methoxybenzene **55** (0.15 mmol, 22.5 mg, 0.5 equiv.) were added and stirred at room temperature for 12 h. Then $NaBH_3CN$ (1.5 mmol, 62.5 mg, 5.0 equiv.) was added to the above reaction mixture and the reaction was stirred for 2 h at room temperature. The reaction was quenched with 2.0 mL saturated $NaHCO_3$ aq. and 3.0 mL H_2O. Then it was extracted with DCM (3.0 mL × 3). The organic layer was combined and dried over Na_2SO_4. Then filtered and concentrated by rotary evaporation. The residue was purified by silica gel chromatography (EtOAc : petroleum ether = 1 : 20) to afford the product **30** (28.4 mg, 96% yield) and **35** (25.0 mg, 93% yield).

7.4 Competition experiments of different kind phenyl alkanes:

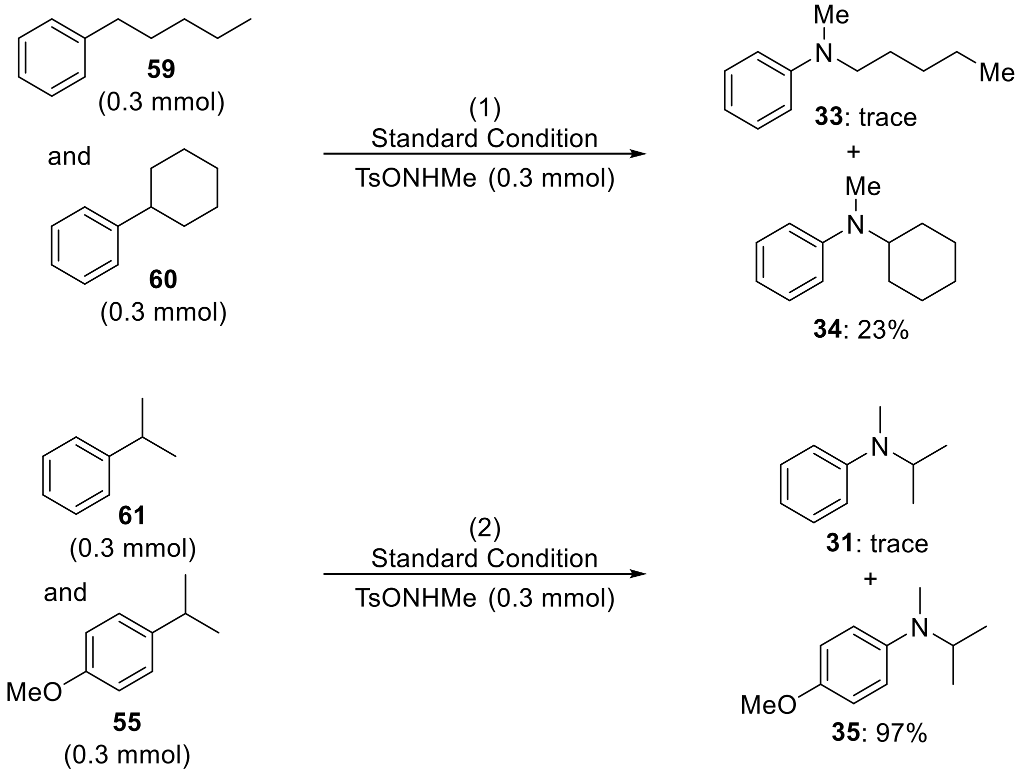

Supplementary Fig 38. Competition experiments of different kind phenyl alkanes

Reaction (1): In an oven dried 25 mL Schlenk tube, which containing a stirring bar, was charged with N-methyl-O-tosylhydroxylamine (**2**, 90.0 mg, 0.45 mmol, 1.5 equiv.), DDQ (0.45 mmol, 102 mg, 1.5 equiv.). The tube was then evacuated and back-filled under N_2 flow (this sequence was repeated three times). H_2O (3.0 mmol, 0.054 mL, 10.0 equiv.), HFIP (1.5 mL), phenylpentane **59** (0.3 mmol, 44.5 mg, 1.0 equiv.), cyclohexylbenzene **60** (0.3 mmol, 48.1 mg, 0.5 equiv.) were added and stirred at room temperature for 12 h. Then $NaBH_3CN$ (1.5 mmol, 62.5 mg, 5.0 equiv.) was added to the above reaction mixture and the reaction was stirred for 2 h at room temperature. The reaction was quenched with 2.0 mL saturated $NaHCO_3$ aq. and 3.0 mL H_2O. Then it was extracted with DCM (3.0 mL × 3). The organic layer was combined and dried over Na_2SO_4. Then filtered and concentrated by rotary evaporation. The yield was determined by ^{1}H NMR using dibromomethane (0.3 mmol) as the internal standard.

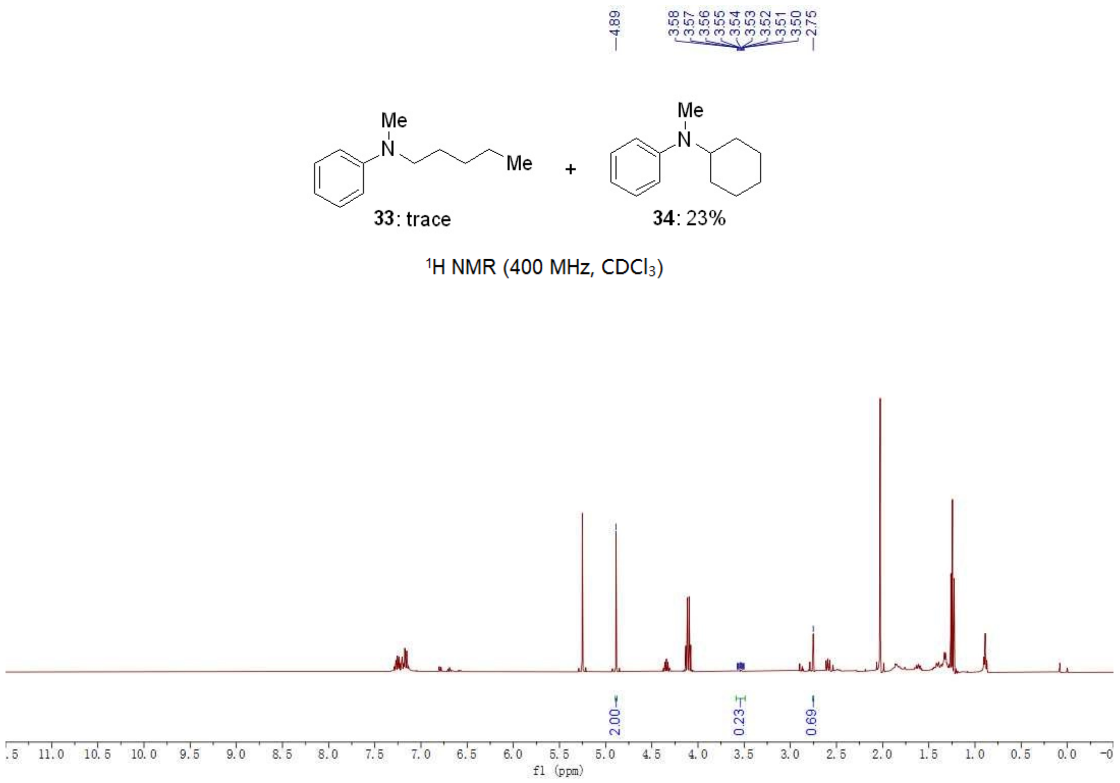

Supplementary Fig 39. The ^{1}H NMR spectrum of Competition experiments of different kind phenyl alkanes (1)

Reaction (2): In an oven dried 25 mL Schlenk tube, which containing a stirring bar, was charged with N-methyl-O-tosylhydroxylamine (**2**, 90.0 mg, 0.45 mmol, 1.5 equiv.), DDQ (0.45 mmol, 102 mg, 1.5 equiv.). The tube was then evacuated and back-filled under N_2 flow (this sequence was repeated three times). H_2O (3.0 mmol, 0.054 mL, 10.0 equiv.), HFIP (1.5 mL), cumene **61** (0.3 mmol, 36.1 mg, 0.5 equiv.), 1-isopropyl-4-methoxybenzene **55** (0.3 mmol, 45.1 mg, 1.0 equiv.) were added and stirred at room temperature for 12 h. Then $NaBH_3CN$ (1.5 mmol, 62.5 mg, 5.0 equiv.) was added to the above reaction mixture and the reaction was stirred for 2 h at room temperature. The reaction was quenched with 2.0 mL saturated $NaHCO_3$ aq. and 3.0 mL H_2O. Then it was extracted with DCM (3.0 mL × 3). The organic layer was combined and dried over Na_2SO_4. Then filtered and concentrated by rotary evaporation. The yield was determined by ^{1}H NMR using dibromomethane (0.3 mol) as the internal standard.

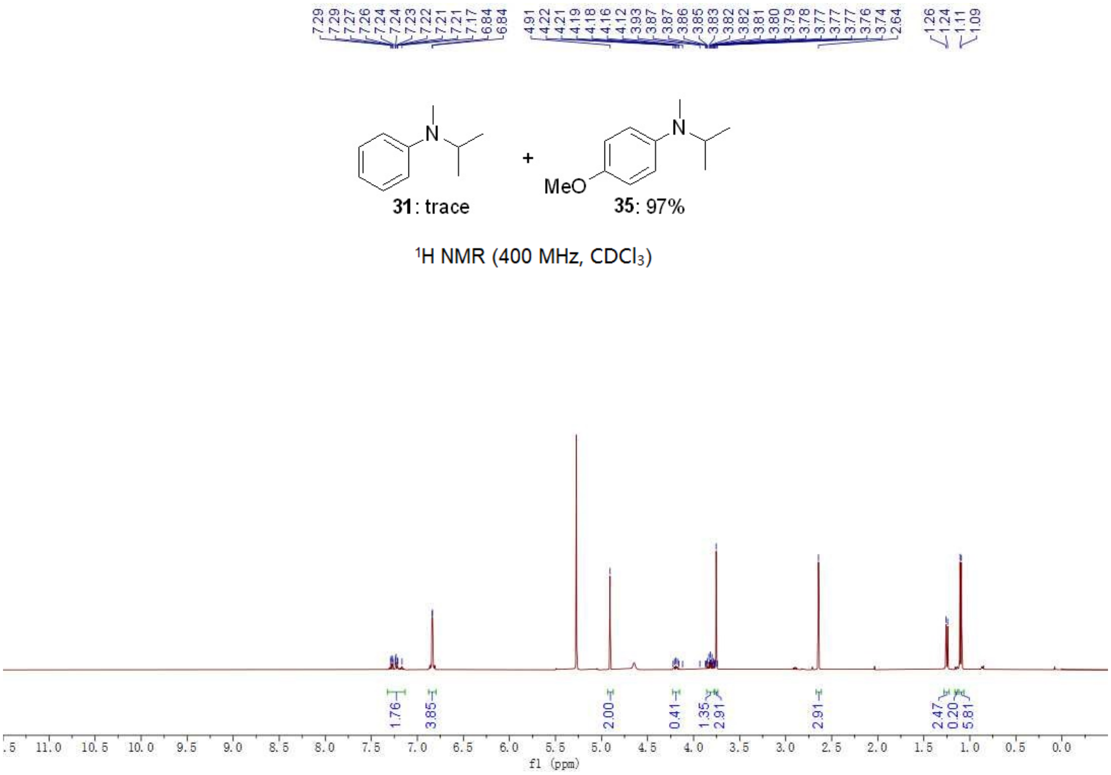

Supplementary Fig 40. The ^{1}H NMR spectrum of Competition experiments of different kind phenyl alkanes (2)

7.5 Radical trapping experiments:

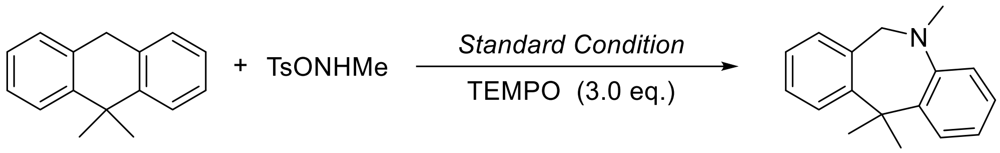

1 2 3

Supplementary Fig 41. Radical trapping experiment (1)

In an oven dried 50 mL Schlenk tube, which containing a stirring bar, was charged with **1** (0.3 mmol, 62.5 mg, 1.0 equiv.), aminating reagent **2** (0.45 mmol, 90.0 mg, 1.5 equiv.), DDQ (0.45 mmol, 103 mg, 1.5 equiv.) and TEMPO (0.9 mmol, 140.6 mg, 3.0 equiv.). The tube was then evacuated and bak-filled under N_2 flow (this sequence was repeated three times). HFIP (1.5 mL) and H_2O (0.054 mL, 10.0 equiv.) were added and stirred at room temperature for 12 h. Then $NaBH_3CN$ (1.5 mmol, 96 mg, 5.0 equiv.) was added to the above reaction mixture and the reaction was stirred for 2 h at room temperature. The reaction was quenched with 2.0 mL saturated $NaHCO_3$ aq. and 3.0 mL H_2O. Then it was extracted with DCM (3.0 mL × 3). The organic layer was combined and dried over Na_2SO_4. Then filtered and concentrated by rotary evaporation. The residue was purified by silica gel chromatography (EtOAc/petroleum ether = 1: 50) to afford the product **3** (59.4 mg, 83% yield), and the recovery rate of TEMPO is 87% (120.4 mg).

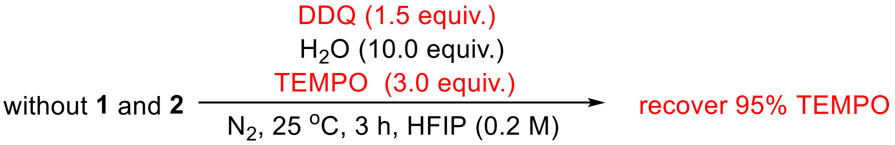

Supplementary Fig 42. Radical trapping experiment (2)

In an oven dried 50 mL Schlenk tube, which containing a stirring bar, was charged DDQ (0.45 mmol, 103 mg, 1.5 equiv.) and TEMPO (0.9 mmol, 140.6 mg, 3.0 equiv.). The tube was then evacuated and bak-filled under N_2 flow (this sequence was repeated three times). HFIP (1.5 mL) and H_2O (0.054 mL, 10.0 equiv.) were added and stirred at room temperature for 3 h. The reaction was quenched with 2.0 mL saturated $NaHCO_3$ aq. and 3.0 mL H_2O. Then it was extracted with DCM (3.0 mL × 3). The organic layer was combined and dried over Na_2SO_4. Then filtered and concentrated by rotary evaporation. The residue was purified by silica gel chromatography (EtOAc/petroleum ether = 1: 10) to afford TEMPO, and the recovery rate of TEMPO is 95% (133.6 mg).

7.6 Competition experiments with Isochroman:

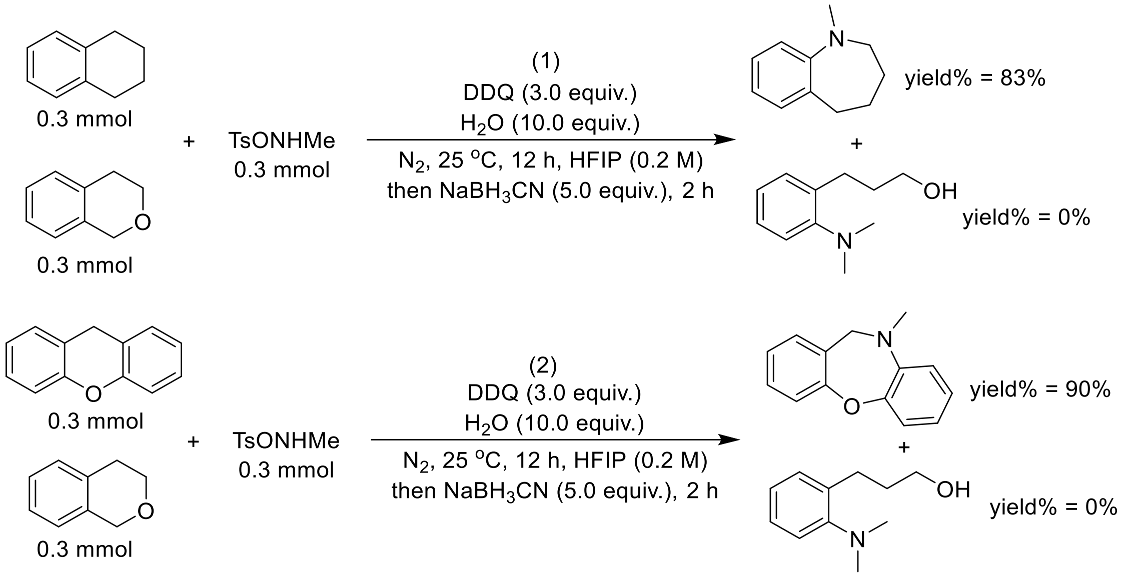

Supplementary Fig 43. Competition experiments with Isochroman

Reaction (1): In an oven dried 25 mL Schlenk tube, which containing a stirring bar, was charged with N-methyl-O-tosylhydroxylamine (**2**, 90.0 mg, 0.45 mmol, 1.5 equiv.), DDQ (0.45 mmol, 102 mg, 1.5 equiv.). The tube was then evacuated and back-filled under N_2 flow (this sequence was repeated three times). H_2O (3.0 mmol, 0.054 mL, 10.0 equiv.), HFIP (1.5 mL), 1,2,3,4-tetrahydronaphthalene (0.3 mmol, 39.6 mg, 1.0 equiv.), isochroman (0.3 mmol, 40.3 mg, 0.5 equiv.) were added and stirred at room temperature for 12 h. Then $NaBH_3CN$ (1.5 mmol, 62.5 mg, 5.0 equiv.) was added to the above reaction mixture and the reaction was stirred for 2 h at room temperature. The reaction was quenched with 2.0 mL saturated $NaHCO_3$ aq. and 3.0 mL H_2O. Then it was extracted with DCM (3.0 mL × 3). The organic layer was combined and dried over Na_2SO_4. Then filtered and concentrated by rotary evaporation. The yield was determined by ^1H NMR using dibromomethane (0.3 mmol) as the internal standard.

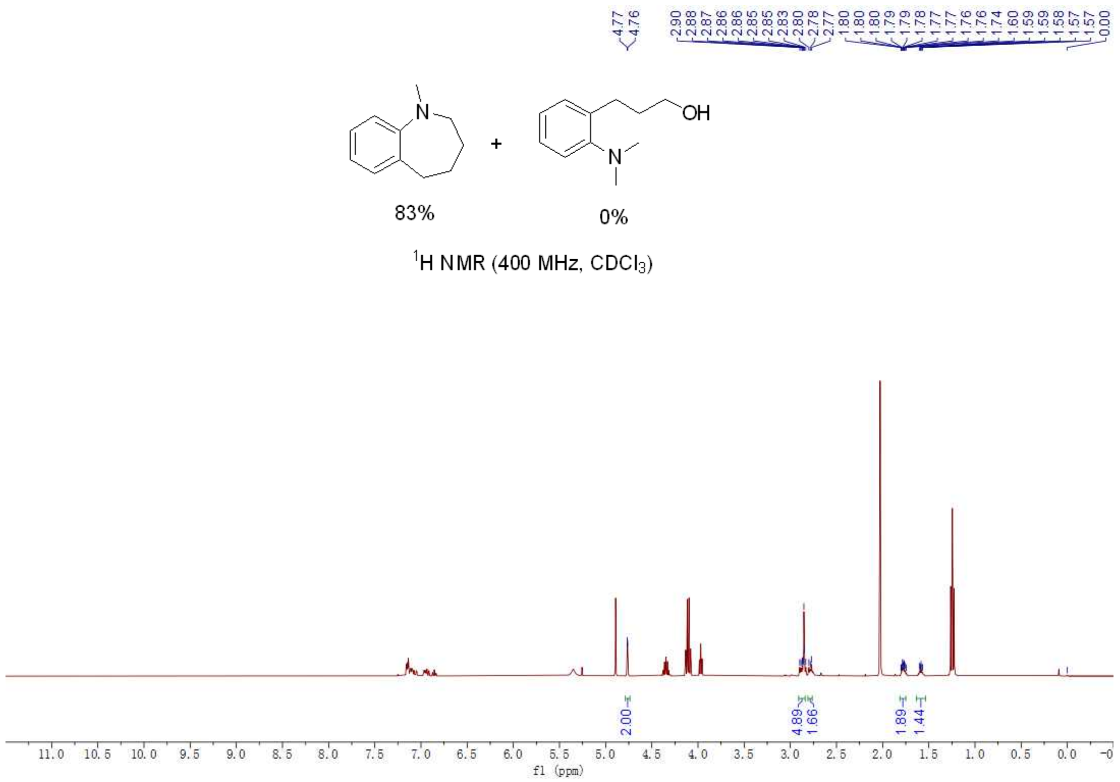

Supplementary Fig 44. The ^{1}H NMR spectrum of Competition experiment with Isochroman (1)

Reaction (2): In an oven dried 25 mL Schlenk tube, which containing a stirring bar, was charged with N-methyl-O-tosylhydroxylamine (**2**, 90.0 mg, 0.45 mmol, 1.5 equiv.), DDQ (0.45 mmol, 102 mg, 1.5 equiv.). The tube was then evacuated and back-filled under N_2 flow (this sequence was repeated three times). H_2O (3.0 mmol, 0.054 mL, 10.0 equiv.), HFIP (1.5 mL), xanthene (0.3 mmol, 54.7 mg, 1.0 equiv.), isochroman (0.3 mmol, 40.3 mg, 0.5 equiv.) were added and stirred at room temperature for 12 h. Then $NaBH_3CN$ (1.5 mmol, 62.5 mg, 5.0 equiv.) was added to the above reaction mixture and the reaction was stirred for 2 h at room temperature. The reaction was quenched with 2.0 mL saturated $NaHCO_3$ aq. and 3.0 mL H_2O. Then it was extracted with DCM (3.0 mL × 3). The organic layer was combined and dried over Na_2SO_4. Then filtered and concentrated by rotary evaporation. The yield was determined by ^{1}H NMR using dibromomethane (0.3 mmol) as the internal standard.

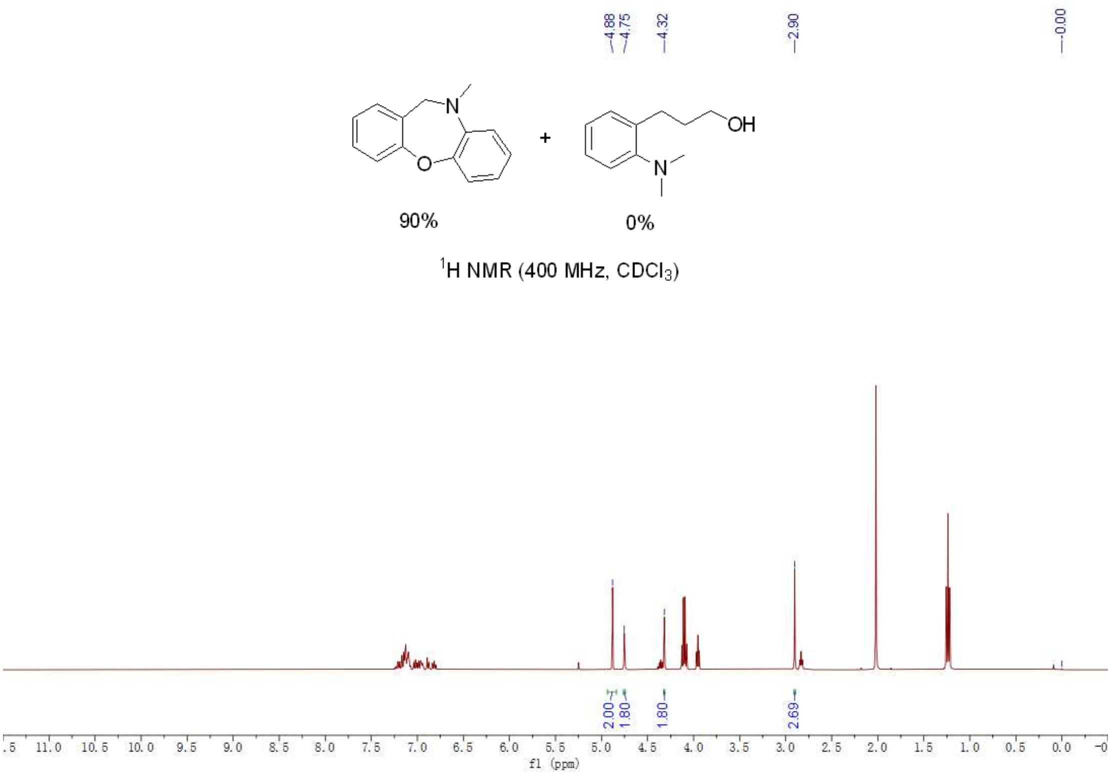

Supplementary Fig 45. The ^{1}H NMR spectrum of Competition experiment with Isochroman (2)

7.7 Iminium ion capture experiments:

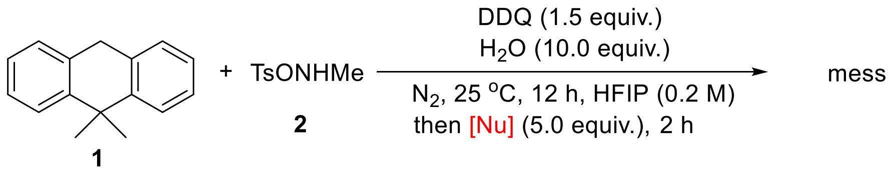

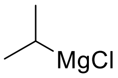	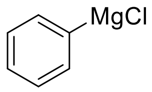
(2.0 M in THF, 1.5 mmol, 0.75 mL) 0%	(1.0 M in THF, 1.5 mmol, 1.5 mL) 0%
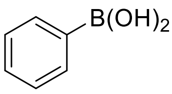	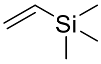
(1.5 mmol, 182.9 mg) 0%	(1.5 mmol, 171.39 mg) 0%
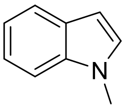	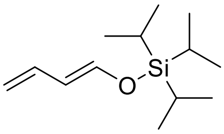
(1.5 mmol, 196.76 mg) 0%	(1.5 mmol, 339.66 mg) 0%
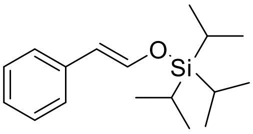	
(1.5 mmol, 414.74 mg) 0%	

Supplementary Table 9: Iminium ion capture experiments

In an oven dried 25 mL Schlenk tube, which containing a stirring bar, was charged with 9,9-dimethyl-9,10-dihydroanthracene **1** (62.5 mg, 0.30 mmol, 1.0 equiv.), N-methyl-O-tosylhydroxylamine (**2**, 90.0 mg, 0.45 mmol, 1.5 equiv.) and DDQ (0.45 mmol, 102 mg, 1.5 equiv.). The tube was then evacuated and back-filled under N_2 flow (this sequence was repeated three times). H_2O (3.0 mmol, 0.054 mL, 10.0 equiv.), HFIP (1.5 mL) were added and stirred at room temperature for 12 h. Then nucleophile (1.5 mmol, 5.0 equiv.) was added to the above reaction mixture and the reaction was stirred for 2 h at room temperature. The reaction was quenched with 2.0 mL saturated $NaHCO_3$ aq. and 3.0 mL H_2O. Then it was extracted with DCM (3.0 mL × 3). The organic layer was combined and dried over Na_2SO_4. Then filtered and concentrated by rotary evaporation. The reaction was detected by GC-MS.

7.8 Control experiments about oxidation reaction step with DDQ:

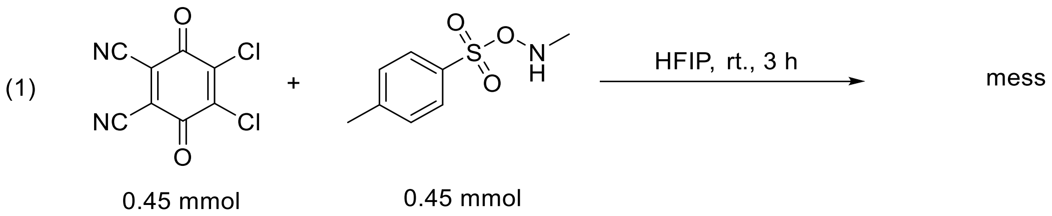

Supplementary Fig 46. Control experiments about oxidation reaction step with DDQ (1)

Reaction (1): In an oven dried 50 mL Schlenk tube, which containing a stirring bar, was charged with N-methyl-O-tosylhydroxylamine (**2**, 90.0 mg, 0.45 mmol, 1.5 equiv.) and DDQ (0.45 mmol, 103 mg, 1.5 equiv.). The tube was then evacuated and bak-filled under N_2 flow (this sequence was repeated three times). HFIP (1.5 mL) were added and stirred at room temperature for 3 h. The reaction was concentrated by rotary evaporation. The yield was determined by ^{1}H NMR using dibromomethane (0.3 mmol) as the internal standard.

(2) 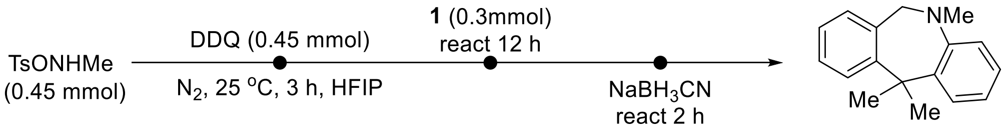

3: 92% NMR yield

Supplementary Fig 47. Control experiments about oxidation reaction step with DDQ (2)

Reaction (2): In an oven dried 50 mL Schlenk tube, which containing a stirring bar, was charged with N-methyl-O-tosylhydroxylamine (**2**, 90.0 mg, 0.45 mmol, 1.5 equiv.) and DDQ (0.45 mmol, 103 mg, 1.5 equiv.). The tube was then evacuated and bak-filled under N_2 flow (this sequence was repeated three times). HFIP (1.5 mL) were added and stirred at room temperature for 3 h. Then 9,9-dimethyl-9,10-dihydroanthracene (62.5 mg, 0.30 mmol, 1.0 equiv.) was added to the above reaction mixture and the reaction was stirred for 12 h at room temperature. Then $NaBH_3CN$ (1.5 mmol, 62.5 mg, 5.0 equiv.) was added to the above reaction mixture and the reaction was stirred for 2 h at room temperature. The reaction was quenched with 2.0 mL saturated $NaHCO_3$ aq. and 3.0 mL H_2O. Then it was extracted with DCM (3.0 mL × 3). The organic layer was combined and dried over Na_2SO_4. Then filtered and concentrated by rotary evaporation. The yield was determined by ^1H NMR using dibromomethane (0.3 mmol) as the internal standard.

(3) 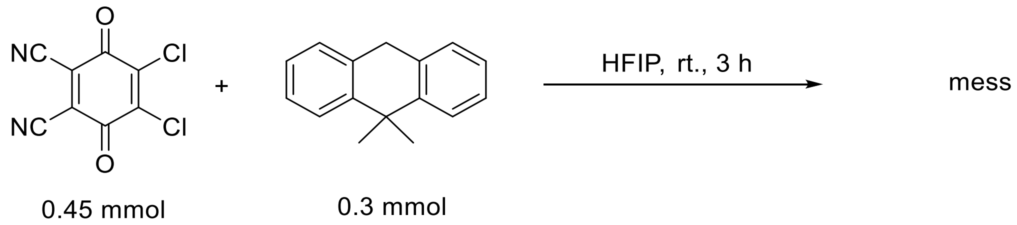

Supplementary Fig 48. Control experiments about oxidation reaction step with DDQ (3)

Reaction (3): In an oven dried 50 mL Schlenk tube, which containing a stirring bar, was charged with 9,9-dimethyl-9,10-dihydroanthracene (62.5 mg, 0.30 mmol, 1.0 equiv.) and DDQ (0.45 mmol, 103 mg, 1.5 equiv.). The tube was then evacuated and bak-filled under N_2 flow (this sequence was repeated three times). HFIP (1.5 mL) were added and stirred at room temperature for 3 h. The reaction was concentrated by rotary evaporation. The yield was determined by ^{1}H NMR using dibromomethane (0.3 mmol) as the internal standard.

8. NMR Spectra

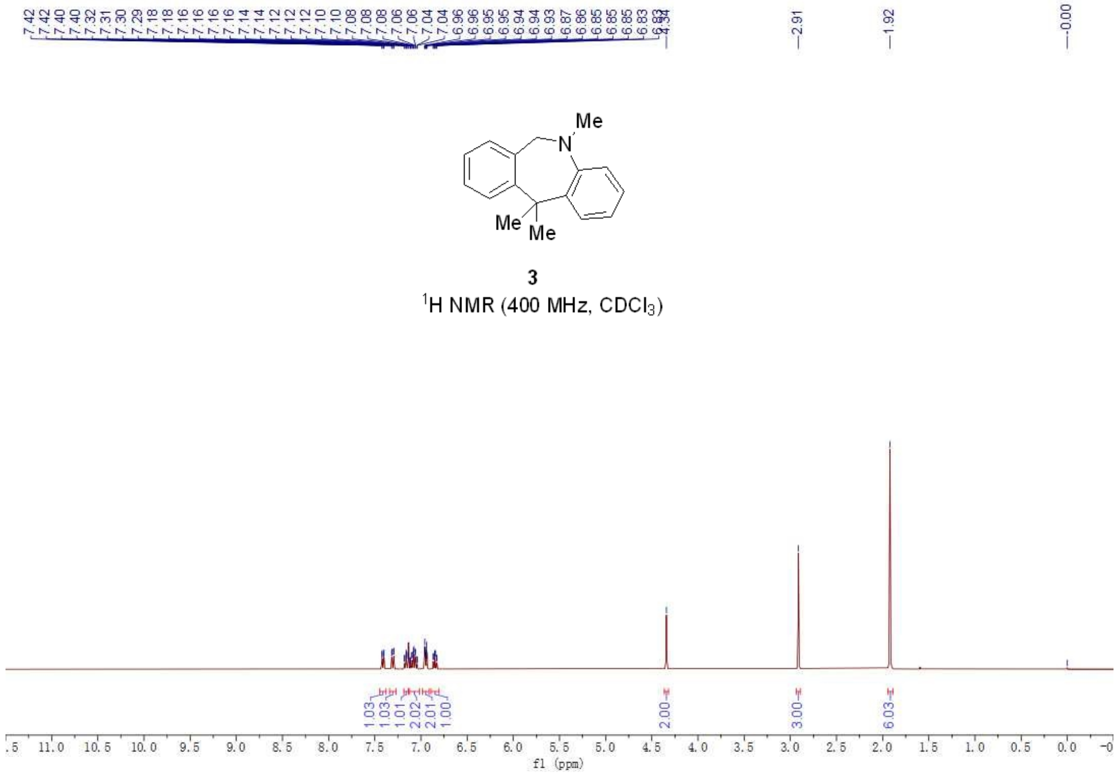

3

^{1}H NMR (400 MHz, $CDCl_3$)

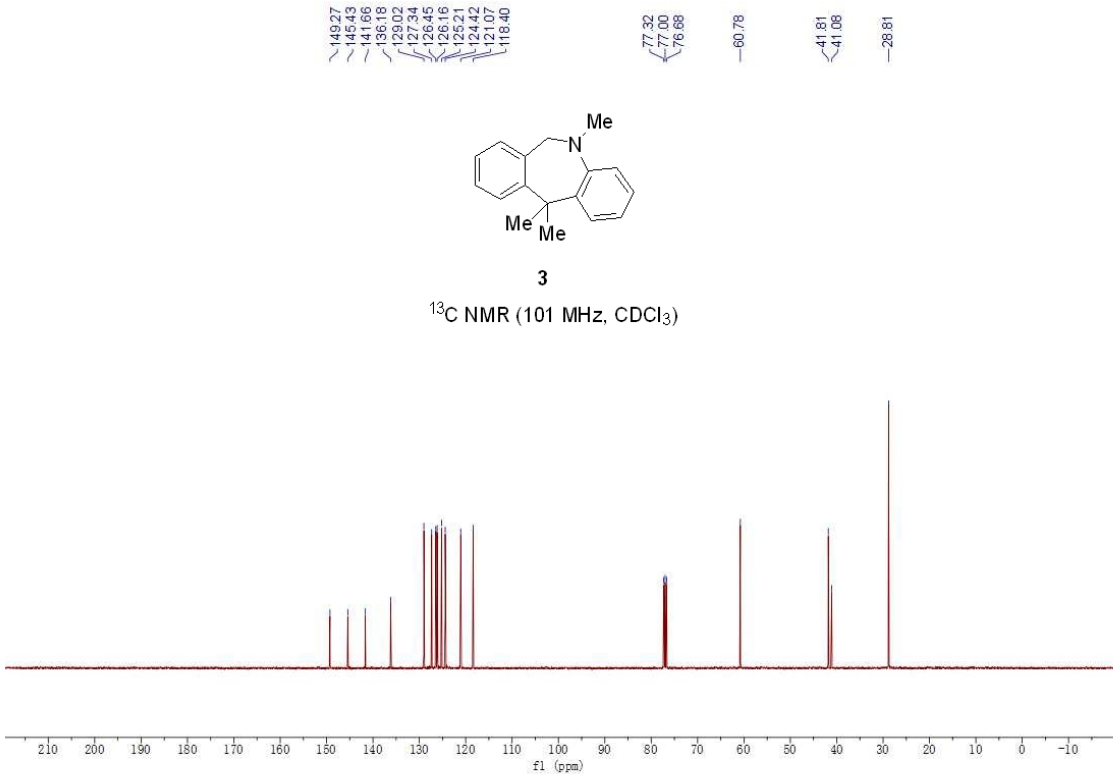

3

^{13}C NMR (101 MHz, $CDCl_3$)

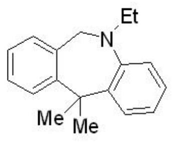

4

^{1}H NMR (400 MHz, $CDCl_3$)

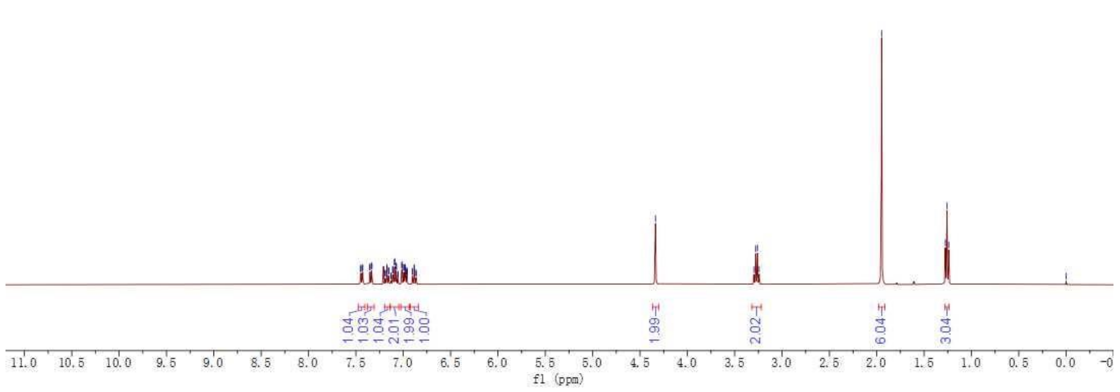

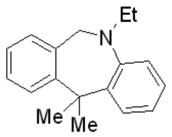

4

^{13}C NMR (101 MHz, $CDCl_3$)

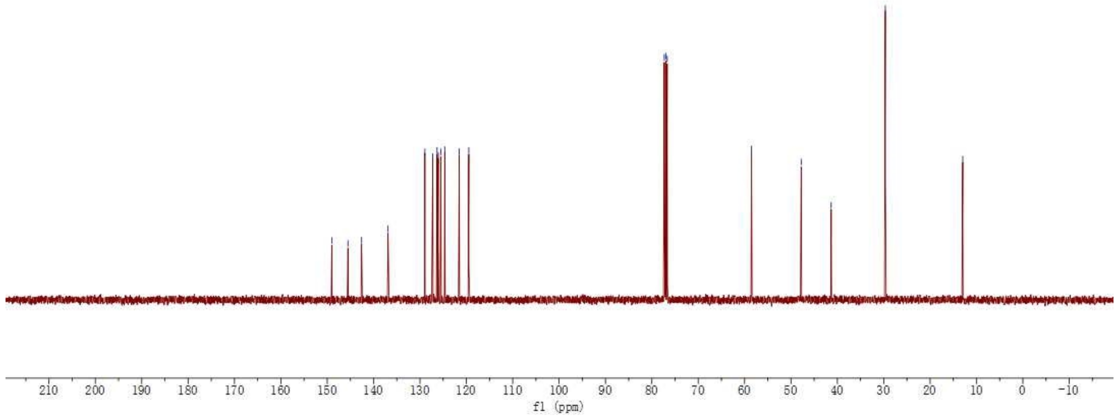

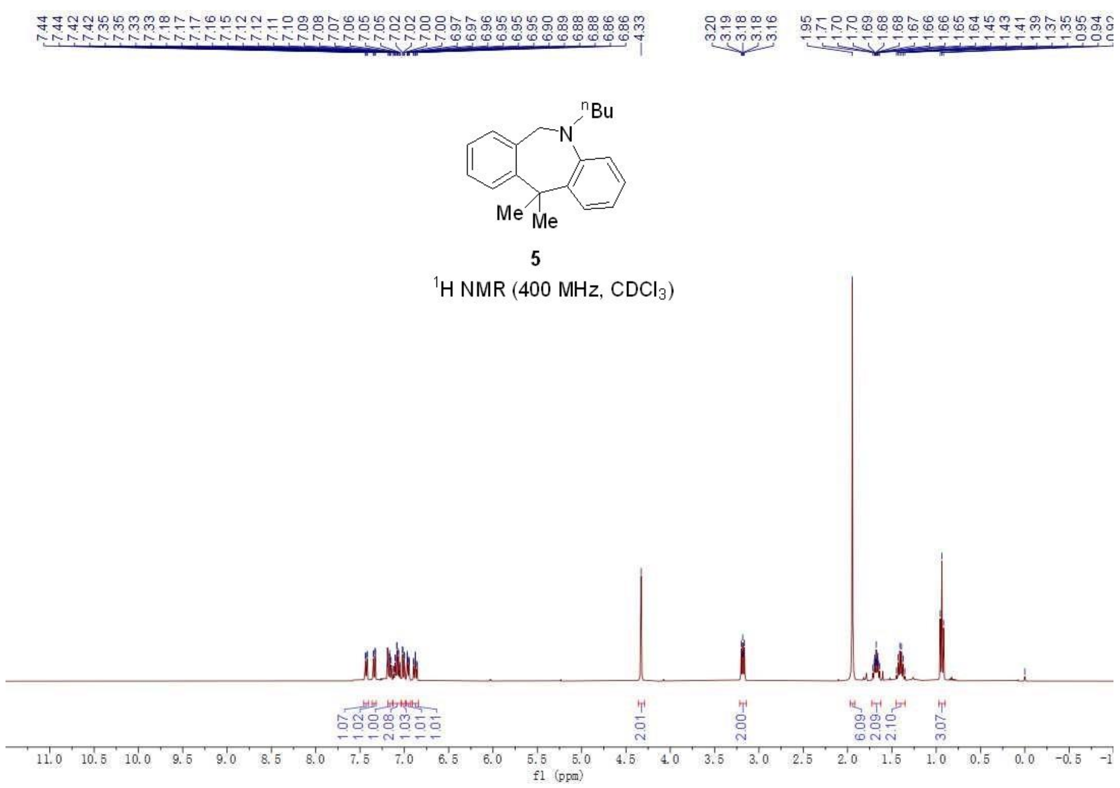

5

^{1}H NMR (400 MHz, $CDCl_3$)

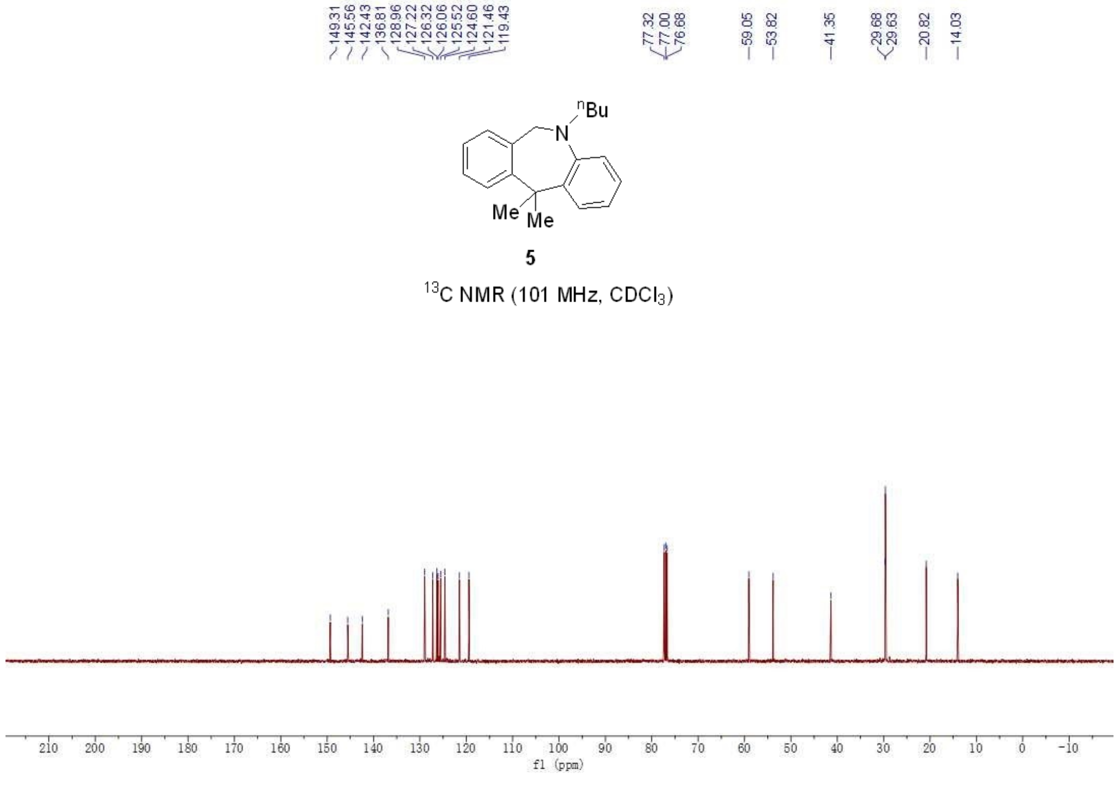

5

^{13}C NMR (101 MHz, $CDCl_3$)

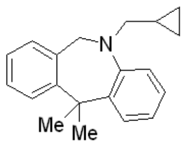

6

^{1}H NMR (400 MHz, $CDCl_3$)

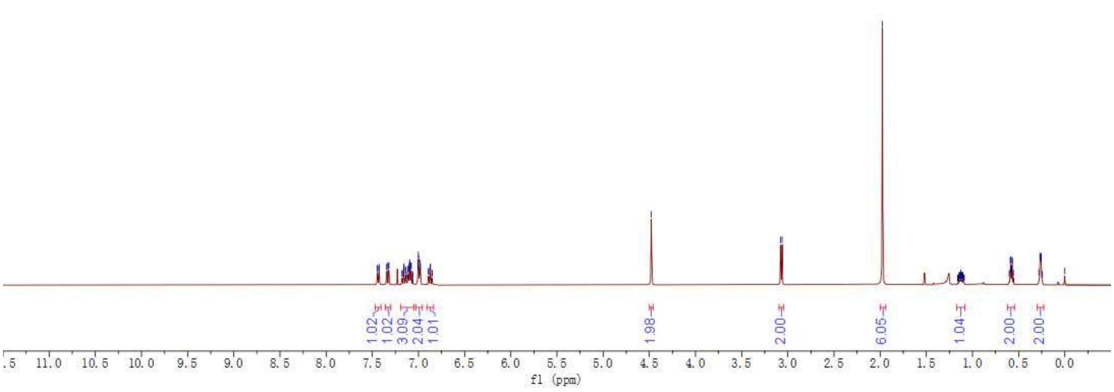

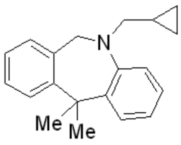

6

^{13}C NMR (101 MHz, $CDCl_3$)

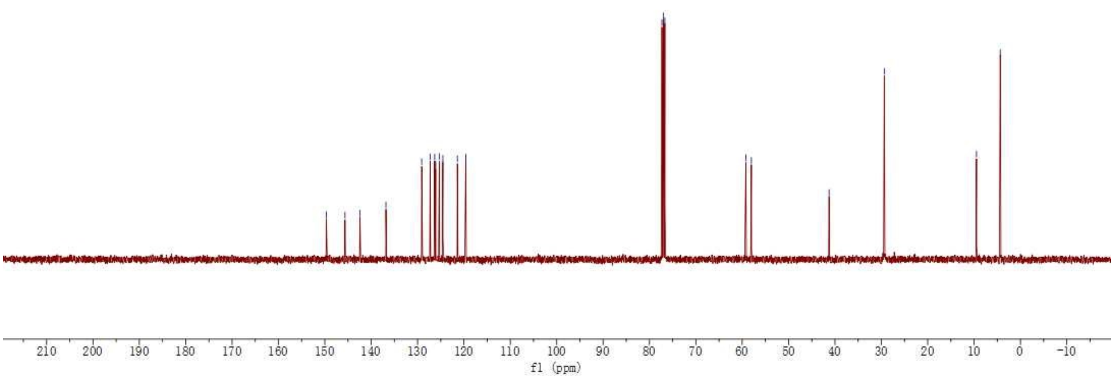

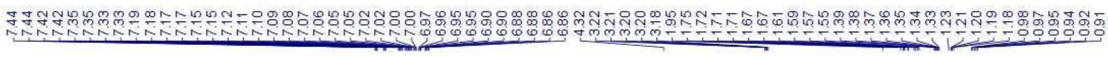

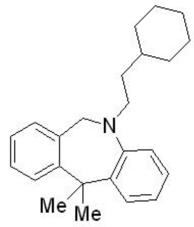

7

^{1}H NMR (400 MHz, $CDCl_3$)

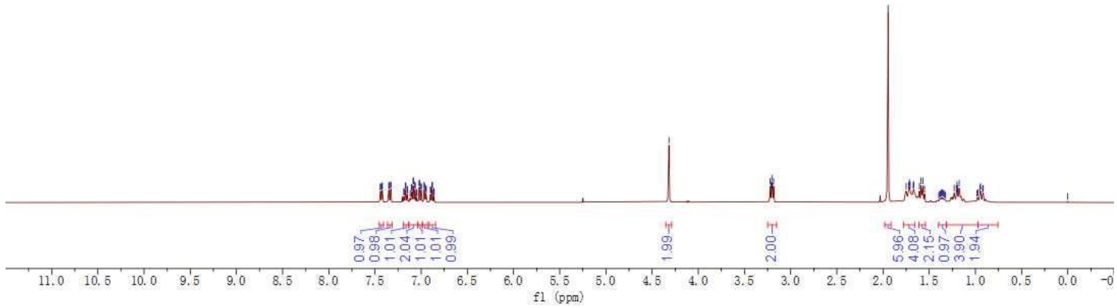

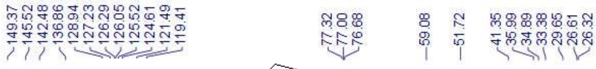

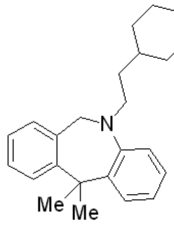

7

^{13}C NMR (101 MHz, $CDCl_3$)

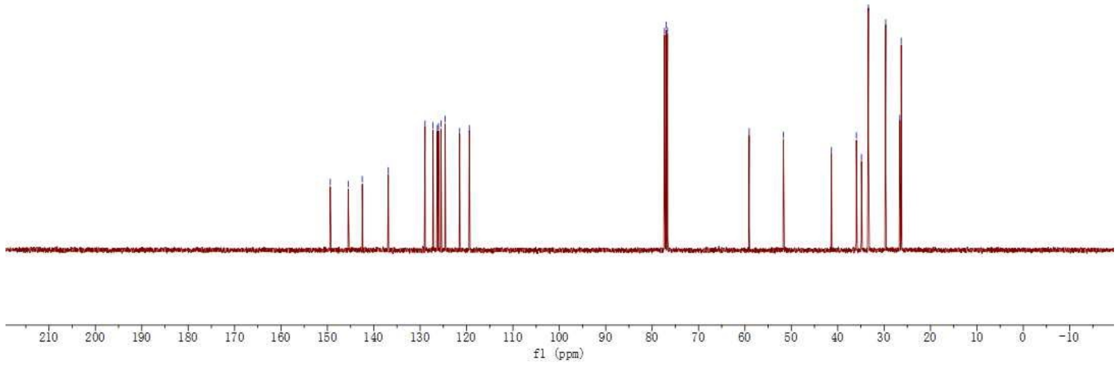

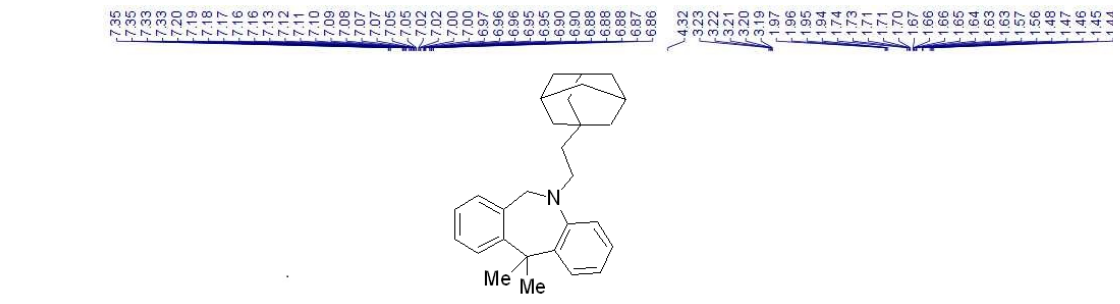

8

^{1}H NMR (400 MHz, $CDCl_3$)

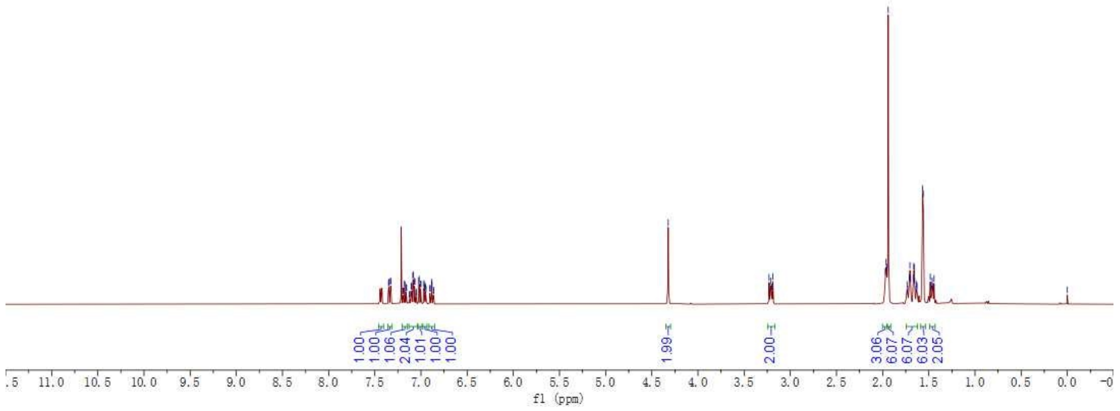

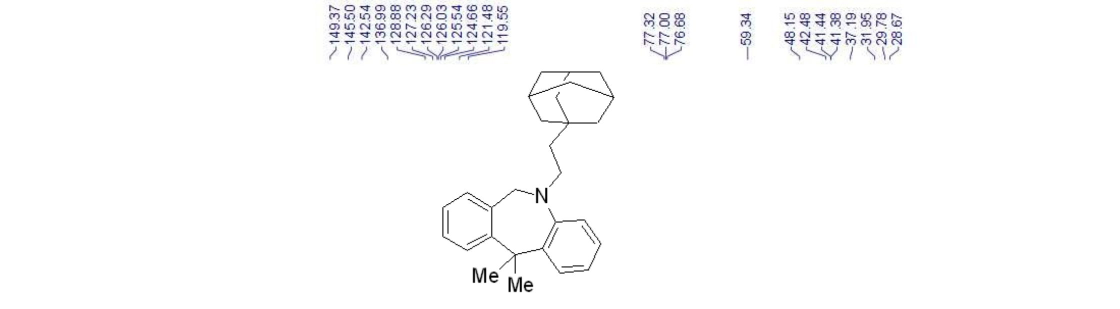

8

^{13}C NMR (101 MHz, $CDCl_3$)

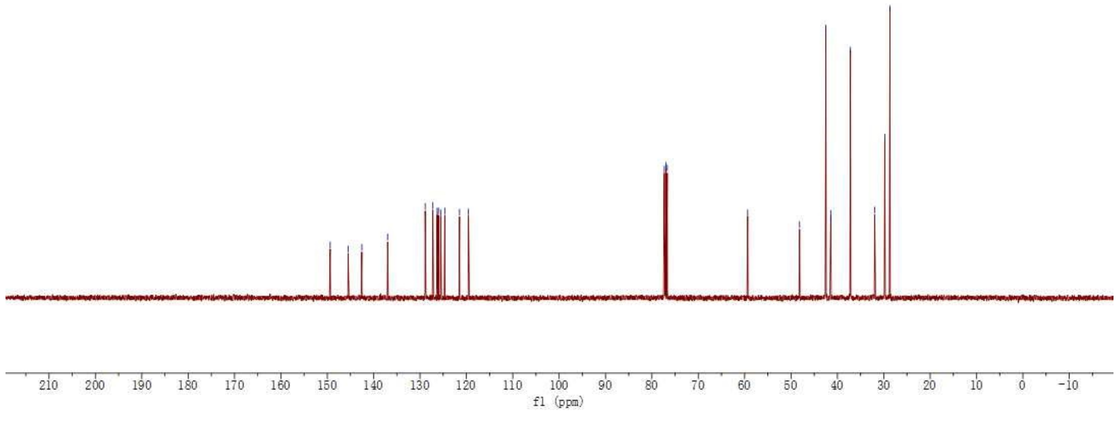

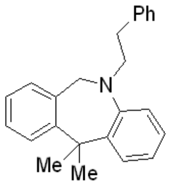

9

^{1}H NMR (400 MHz, $CDCl_3$)

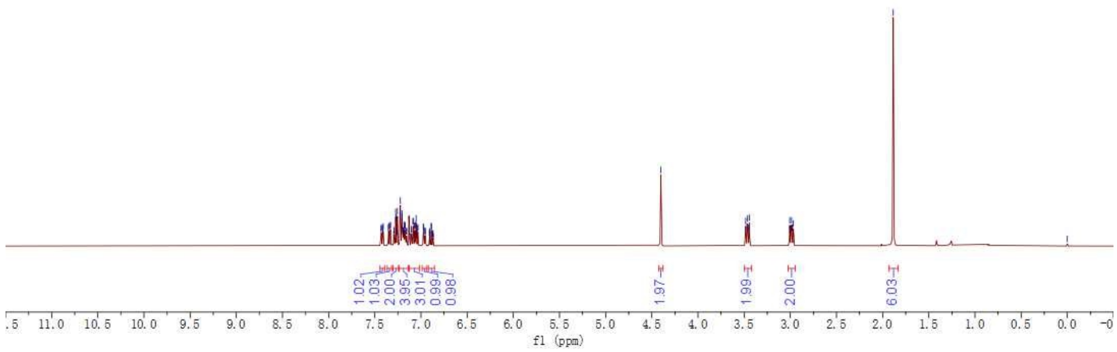

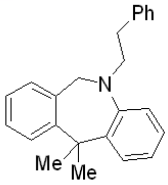

9

^{13}C NMR (101 MHz, $CDCl_3$)

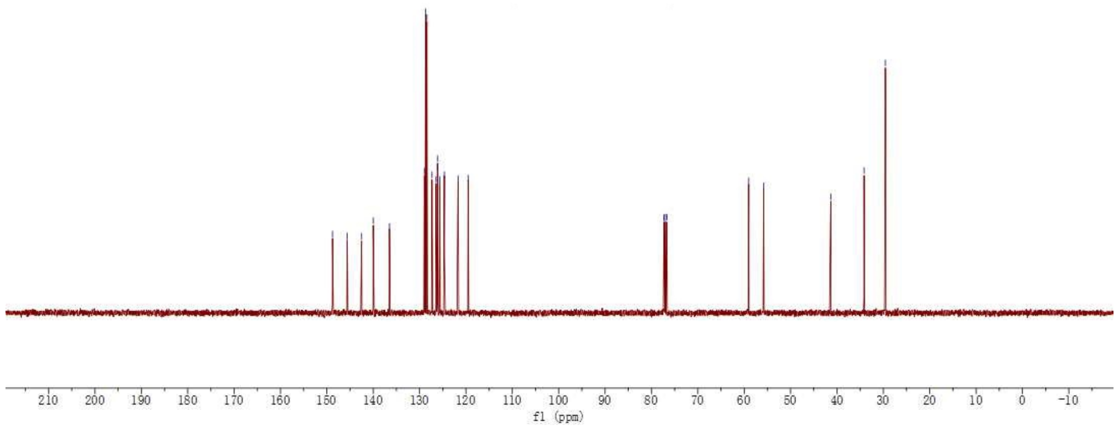

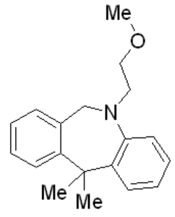

10

^{1}H NMR (400 MHz, $CDCl_3$)

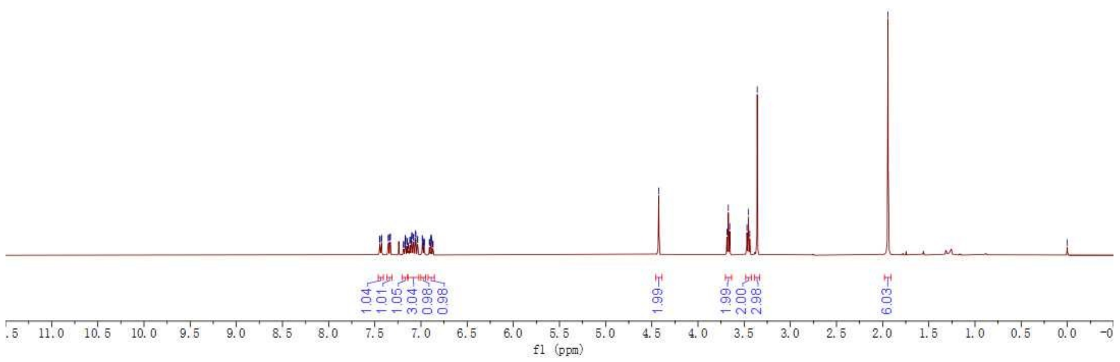

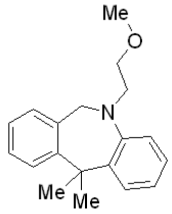

10

^{13}C NMR (101 MHz, $CDCl_3$)

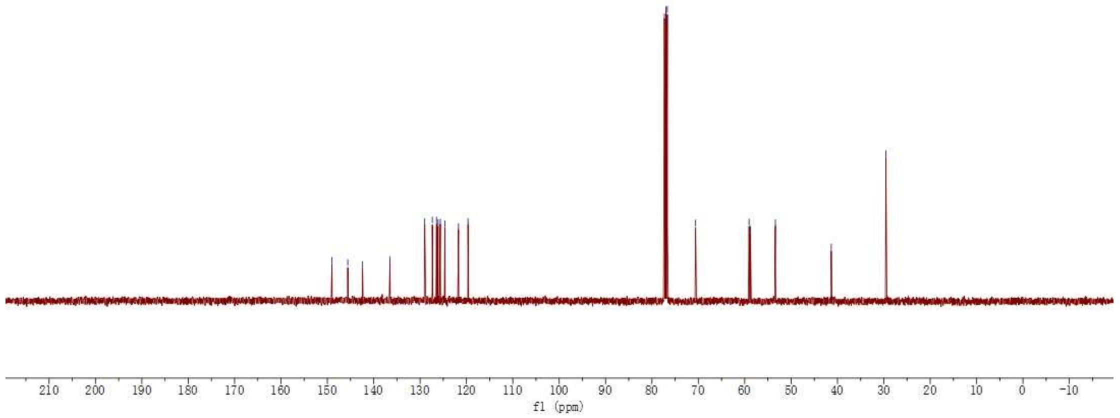

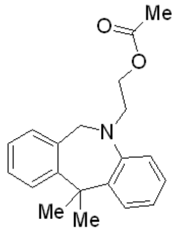

11

^{1}H NMR (400 MHz, $CDCl_3$)

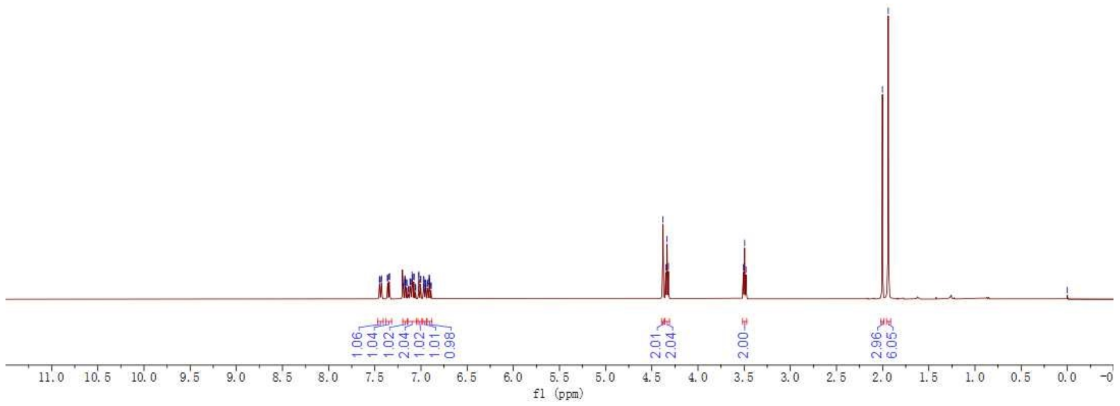

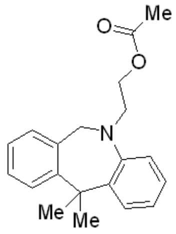

11

^{13}C NMR (101 MHz, $CDCl_3$)

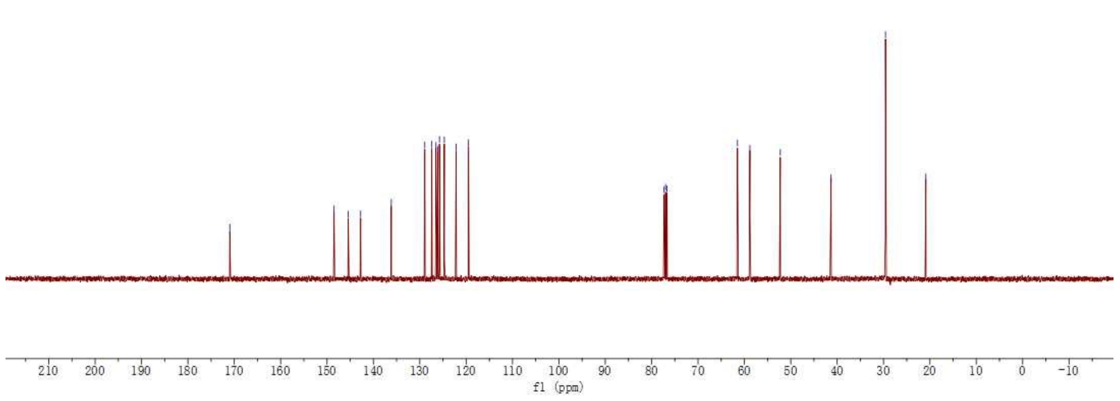

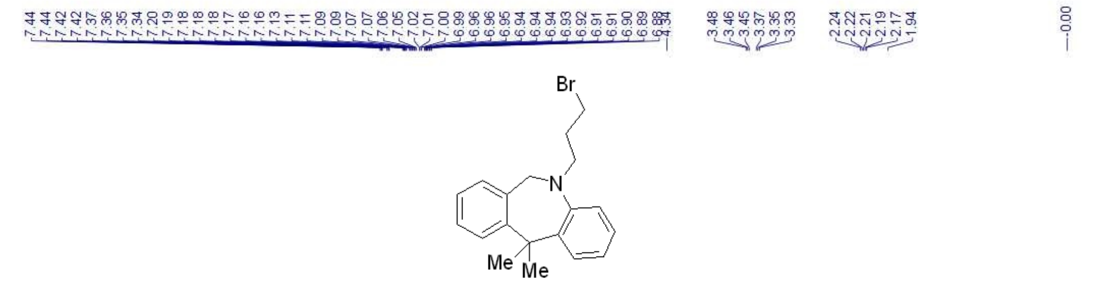

12

^{1}H NMR (400 MHz, $CDCl_3$)

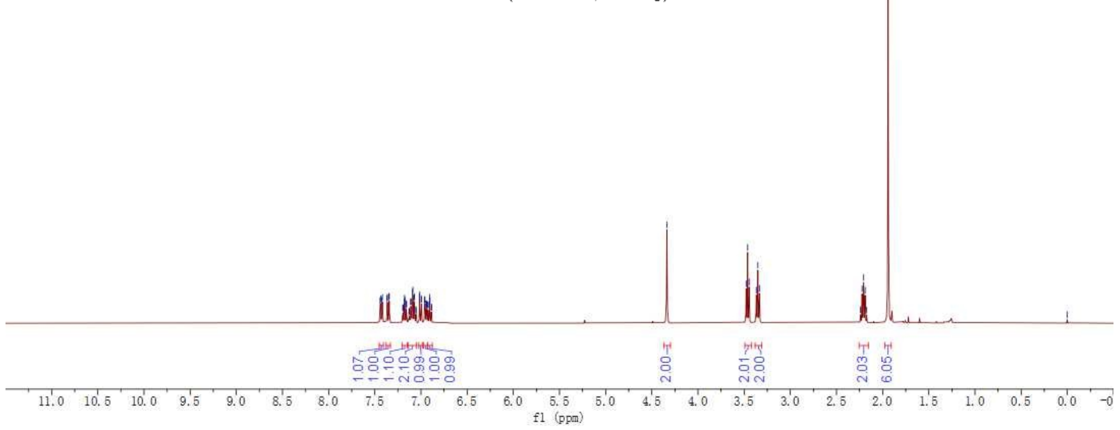

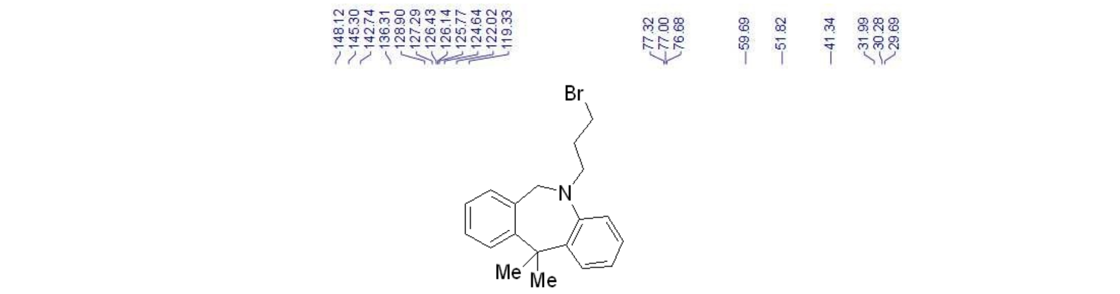

12

^{13}C NMR (101 MHz, $CDCl_3$)

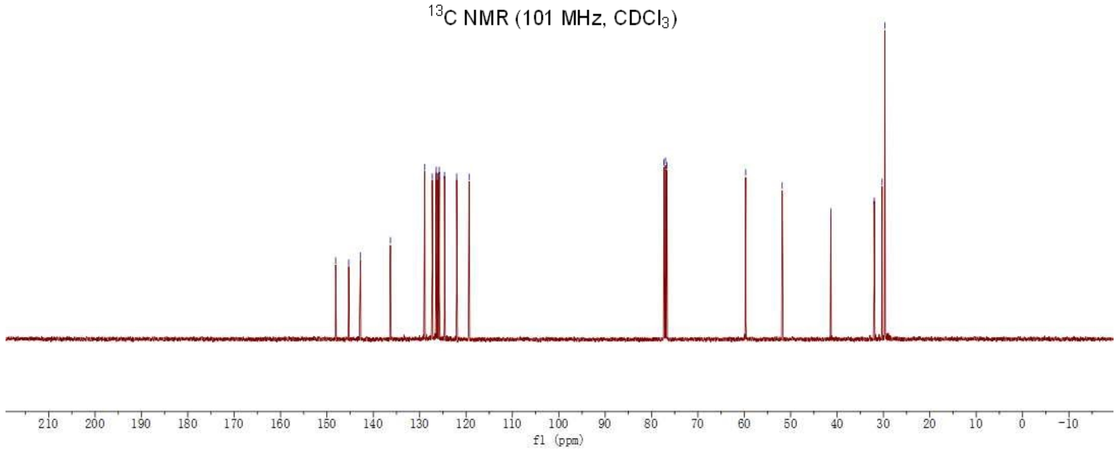

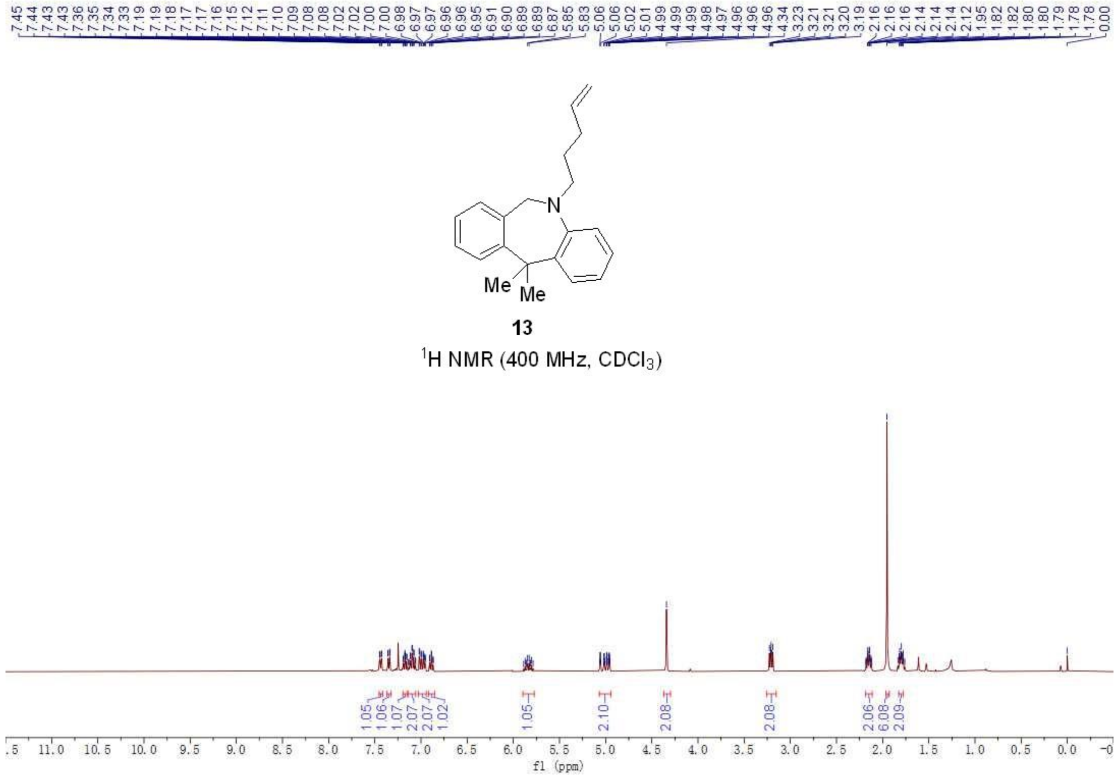
7.45 7.44 7.43 7.36 7.35 7.34 7.33 7.19 7.18 7.17 7.16 7.15 7.12 7.11 7.10 7.09 7.08 7.08 7.02 7.02 7.00 7.00 6.98 6.97 6.97 6.96 6.96 6.95 6.91 6.90 6.89 6.89 6.87 5.85 5.83 5.06 5.06 5.02 5.01 4.99 4.99 4.98 4.97 4.96 4.96 4.34 3.23 3.21 3.21 3.20 3.19 2.16 2.16 2.14 2.14 2.12 1.95 1.82 1.80 1.80 1.79 1.78 0.00
N
Me
Me
13
^{1}H NMR (400 MHz, CDCl$_3$)
1.05 1.06 1.07 2.07 2.07 1.02 1.05 2.10 2.08 2.08 2.06 6.08 2.09
f1 (ppm)

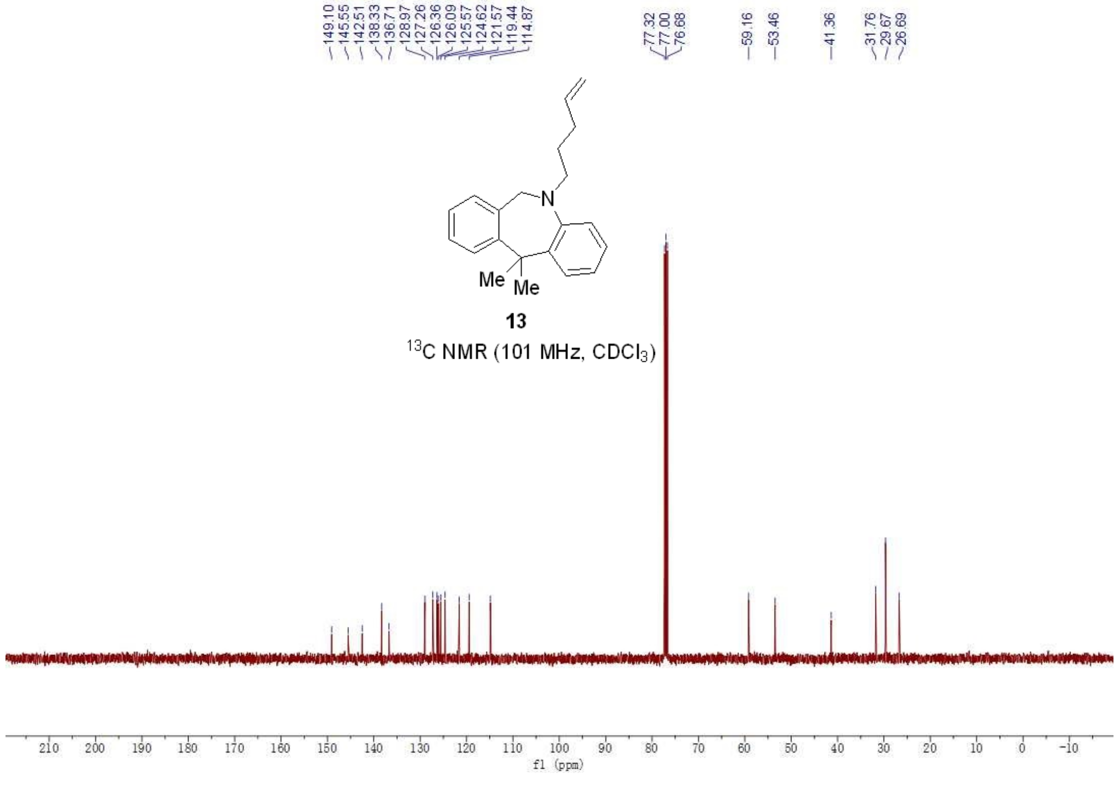
149.10 145.55 142.51 138.33 136.71 128.97 127.26 126.36 126.09 125.57 124.62 121.57 119.44 114.87 77.32 77.00 76.68 59.16 53.46 41.36 31.76 29.67 26.69
N
Me
Me
13
^{13}C NMR (101 MHz, CDCl$_3$)
f1 (ppm)

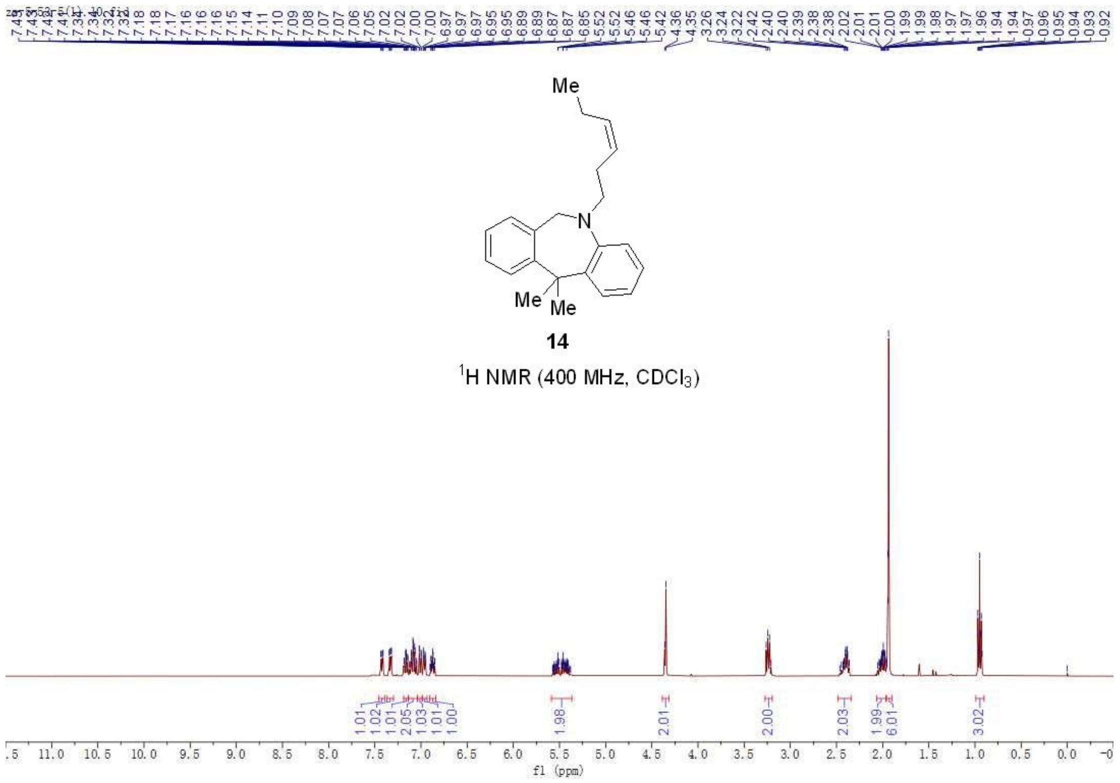
Me
N
Me
Me
14
1H NMR (400 MHz, CDCl3)
f1 (ppm)

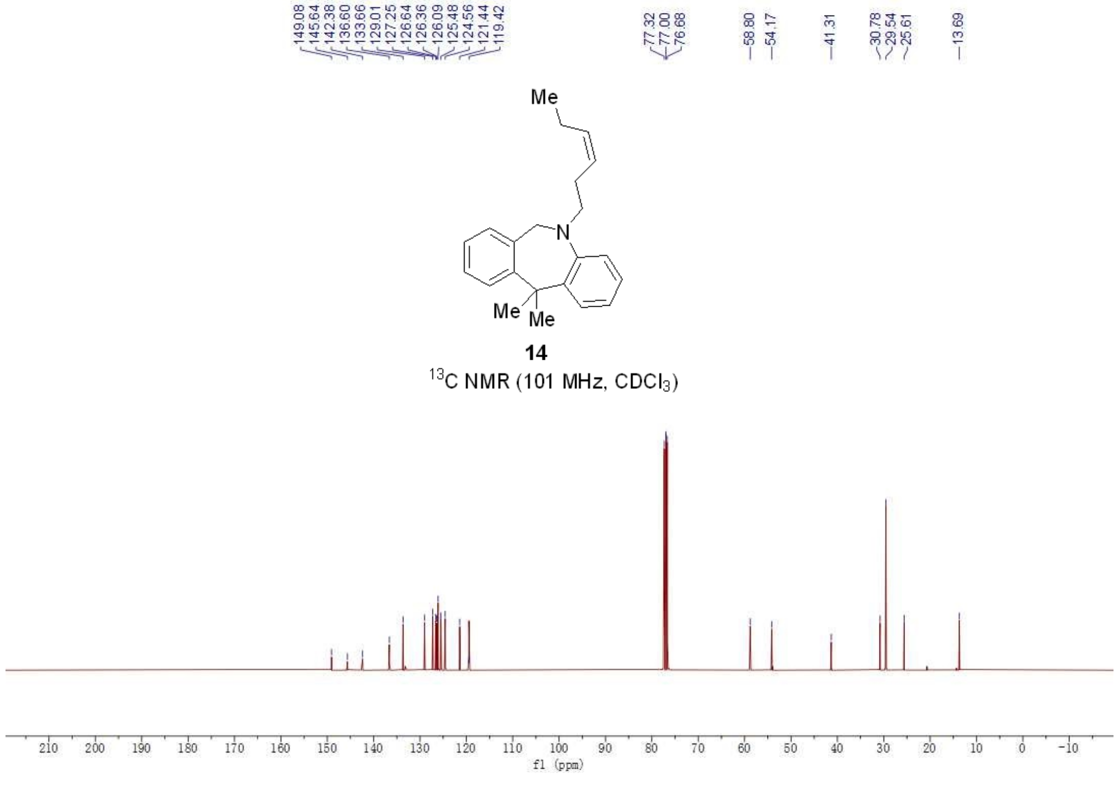
149.08
145.64
142.38
136.60
133.66
129.01
127.25
126.64
126.36
126.09
125.48
124.56
121.44
119.42
77.32
77.00
76.68
58.80
54.17
41.31
30.78
29.54
25.61
13.69
Me
N
Me
Me
14
13C NMR (101 MHz, CDCl3)
f1 (ppm)

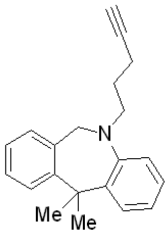

15

^{1}H NMR (400 MHz, $CDCl_3$)

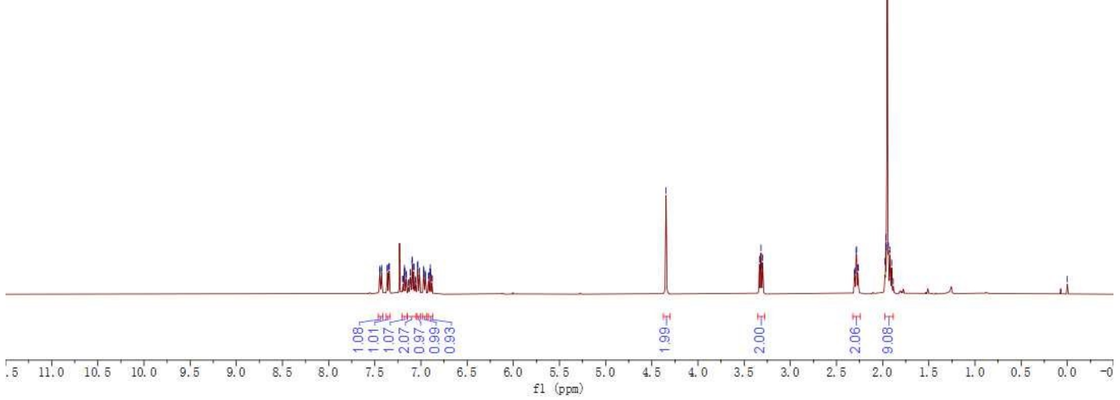

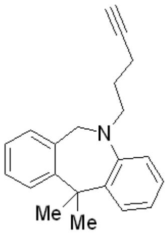

15

^{13}C NMR (101 MHz, $CDCl_3$)

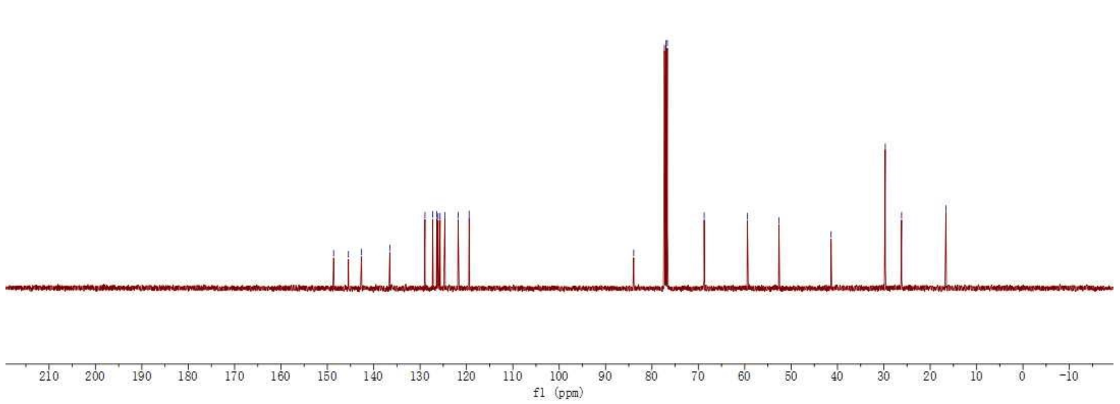

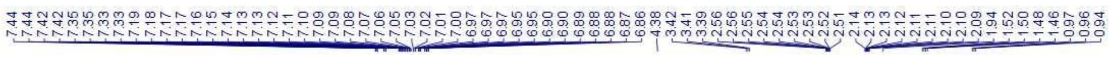

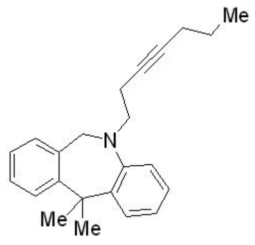

16

^{1}H NMR (400 MHz, $CDCl_3$)

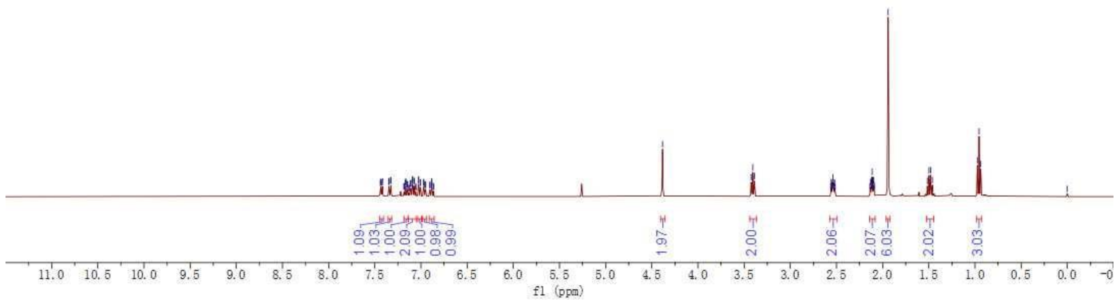

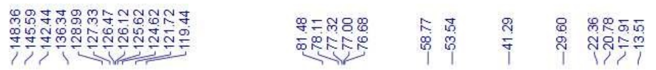

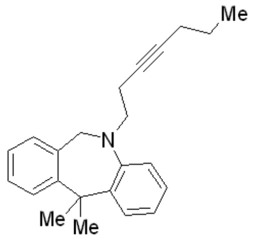

16

^{13}C NMR (101 MHz, $CDCl_3$)

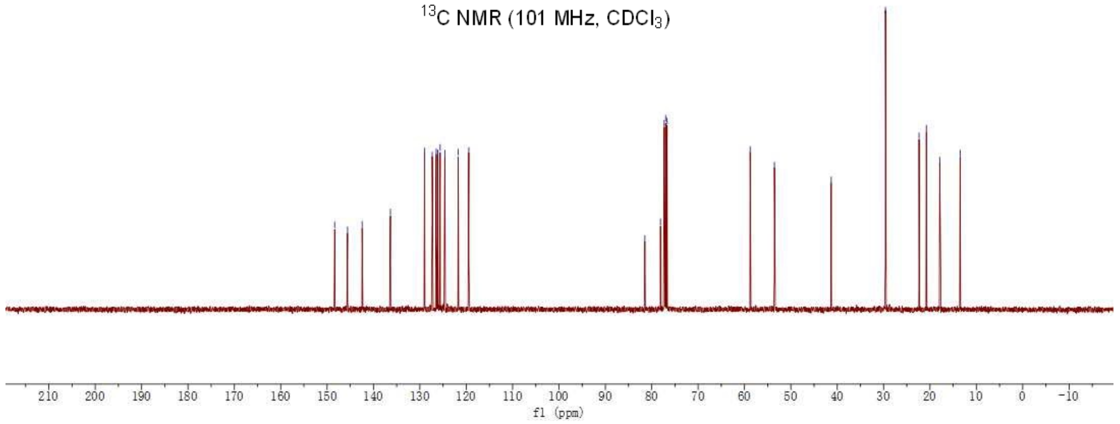

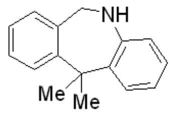

17

^{1}H NMR (400 MHz, $CDCl_3$)

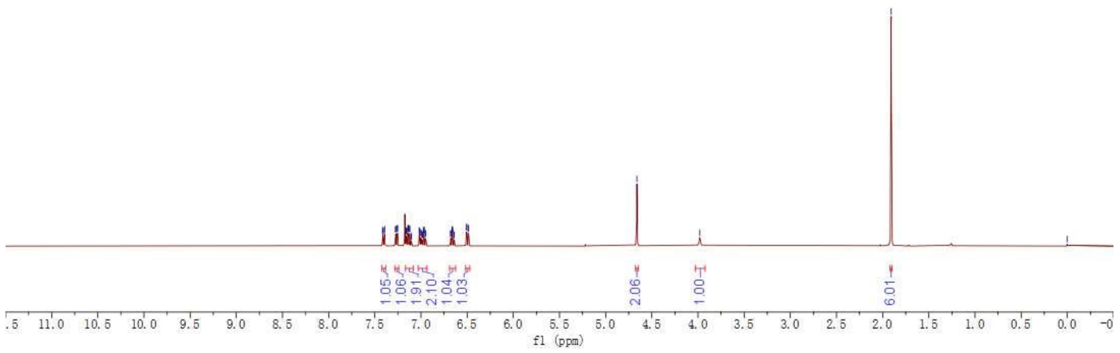

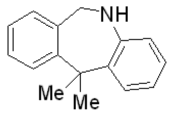

17

^{13}C NMR (101 MHz, $CDCl_3$)

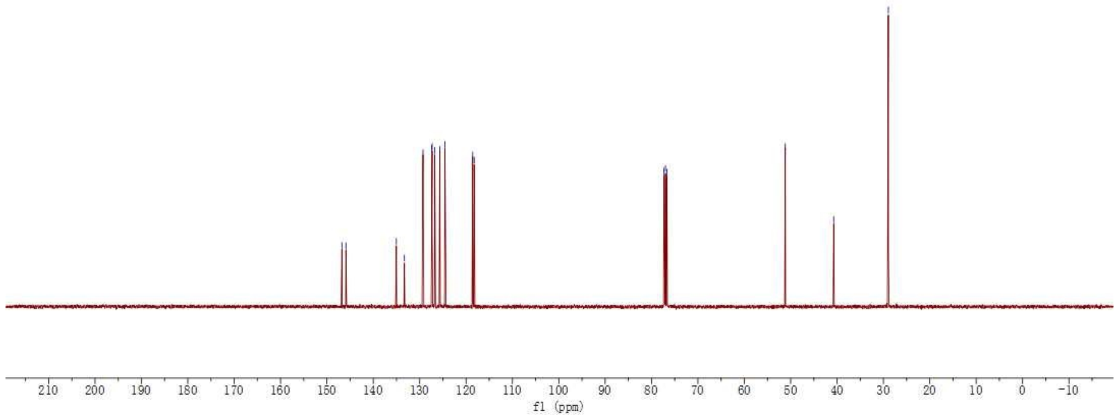

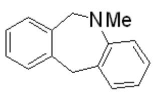

18

^{1}H NMR (400 MHz, $CDCl_3$)

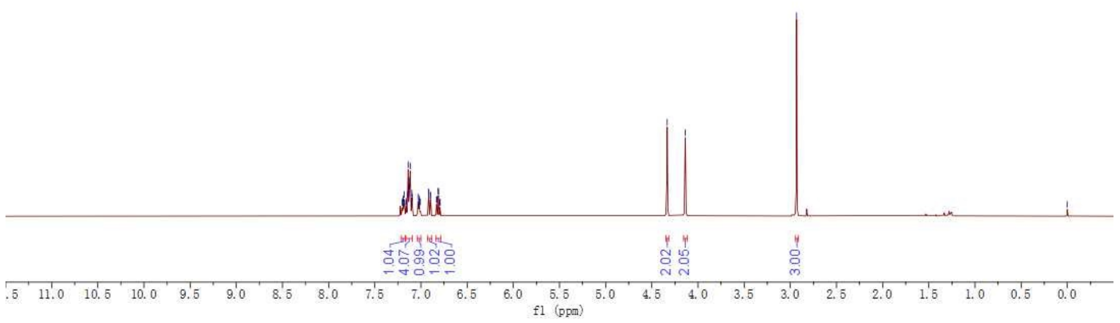

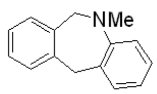

18

^{13}C NMR (101 MHz, $CDCl_3$)

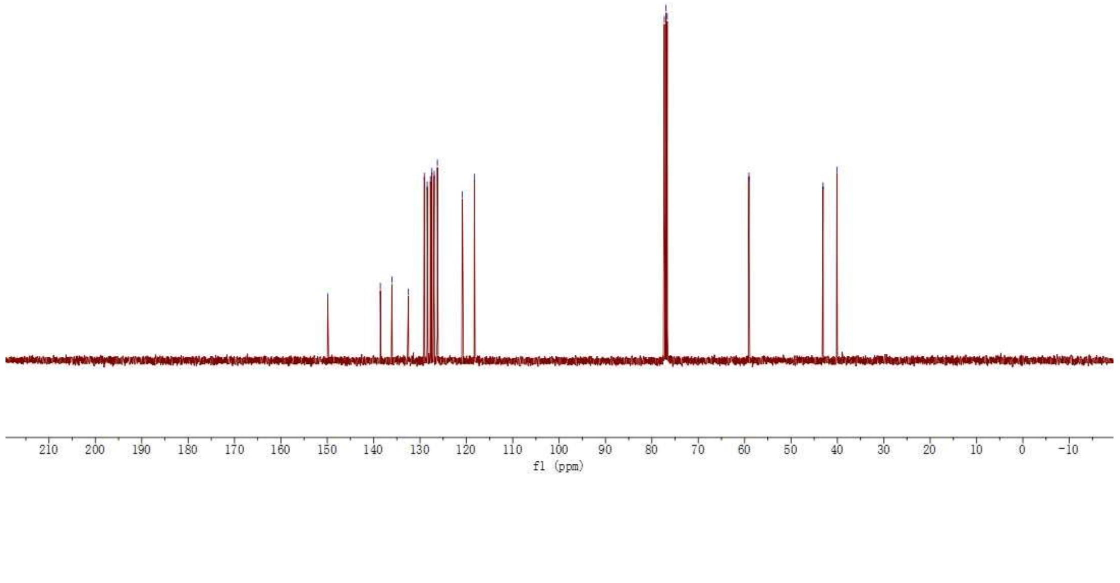

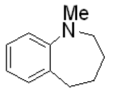

19

^{1}H NMR (400 MHz, $CDCl_3$)

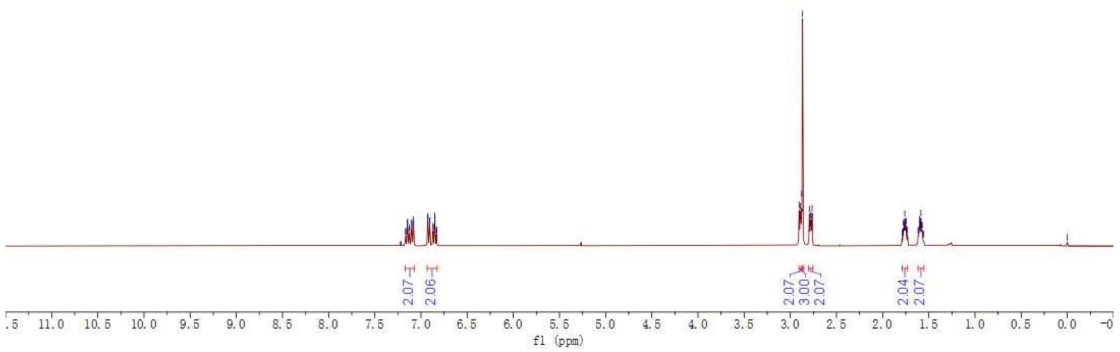

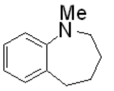

19

^{13}C NMR (101 MHz, $CDCl_3$)

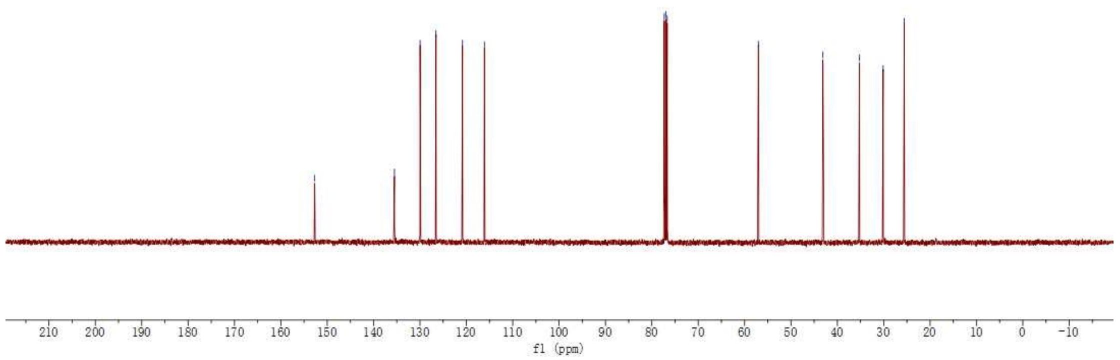

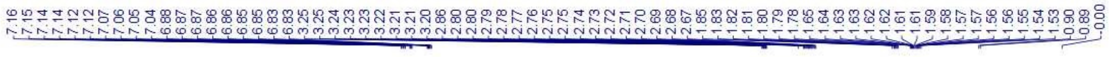

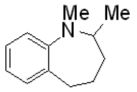

20

^{1}H NMR (400 MHz, $CDCl_3$)

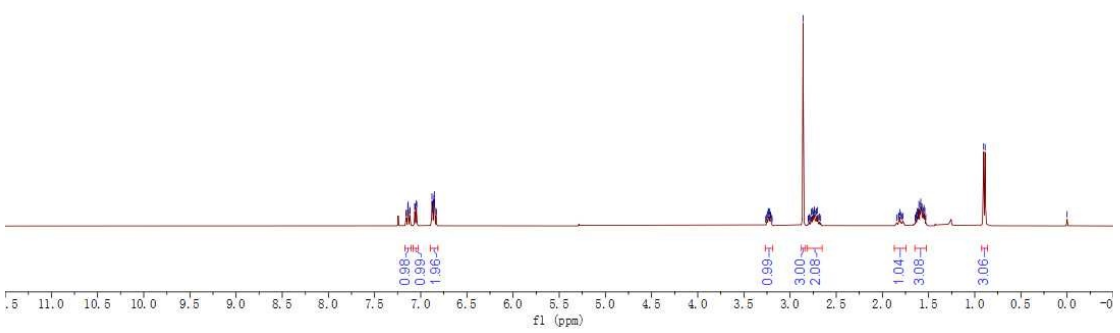

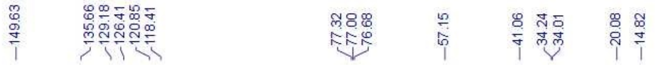

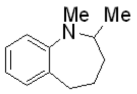

20

^{13}C NMR (101 MHz, $CDCl_3$)

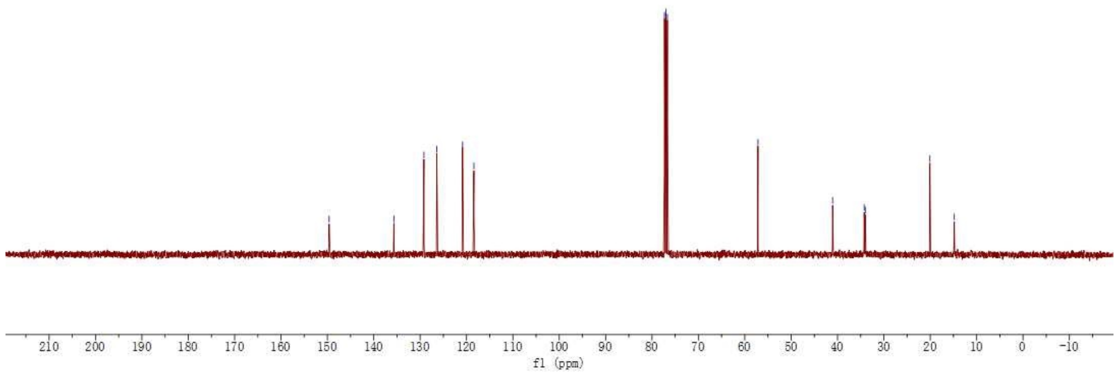

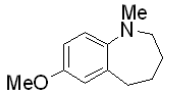

21

^{1}H NMR (400 MHz, $CDCl_3$)

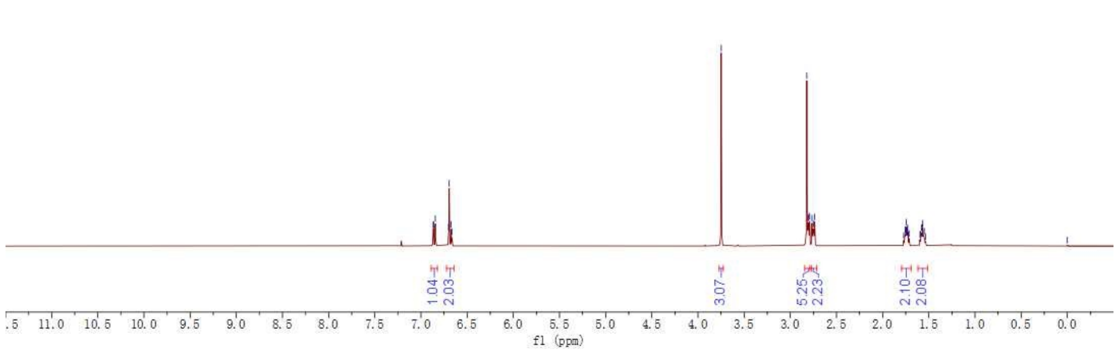

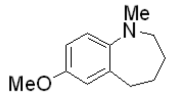

21

^{13}C NMR (101 MHz, $CDCl_3$)

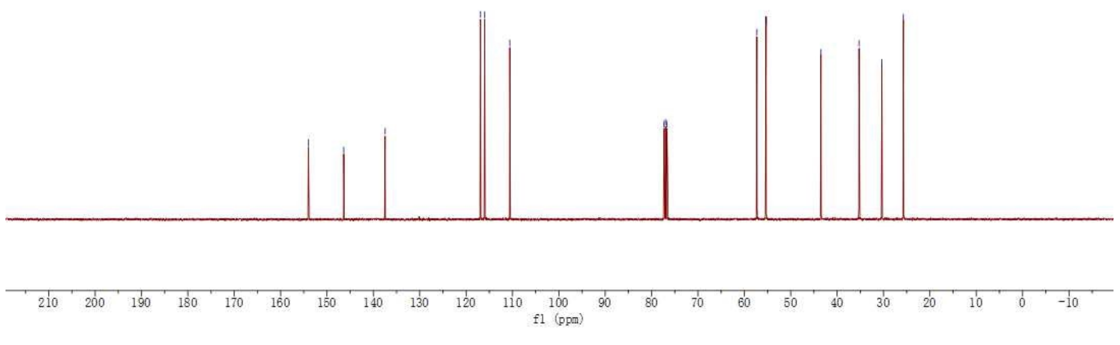

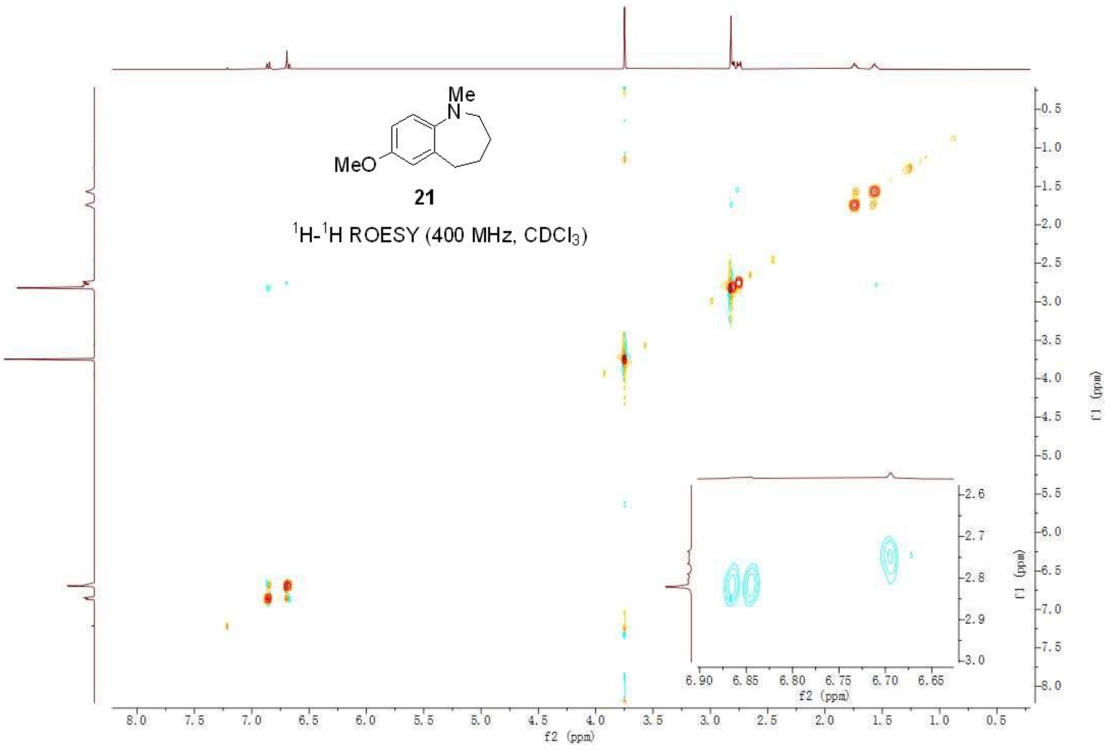

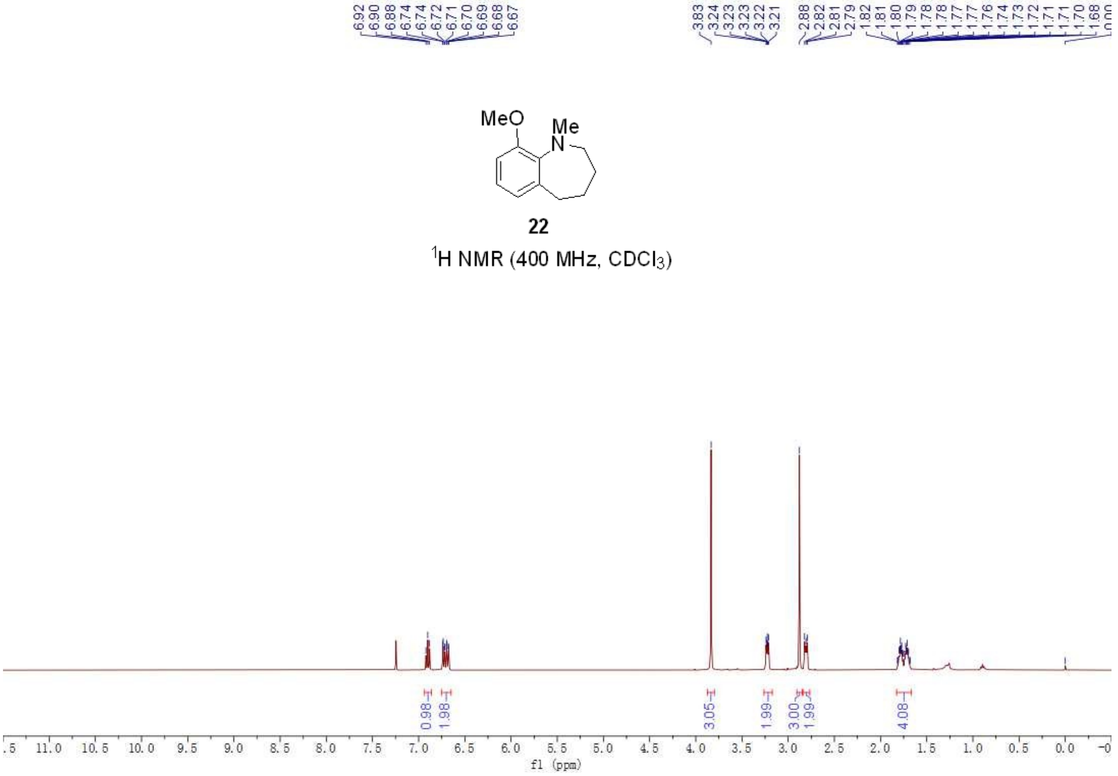

22

^{1}H NMR (400 MHz, $CDCl_3$)

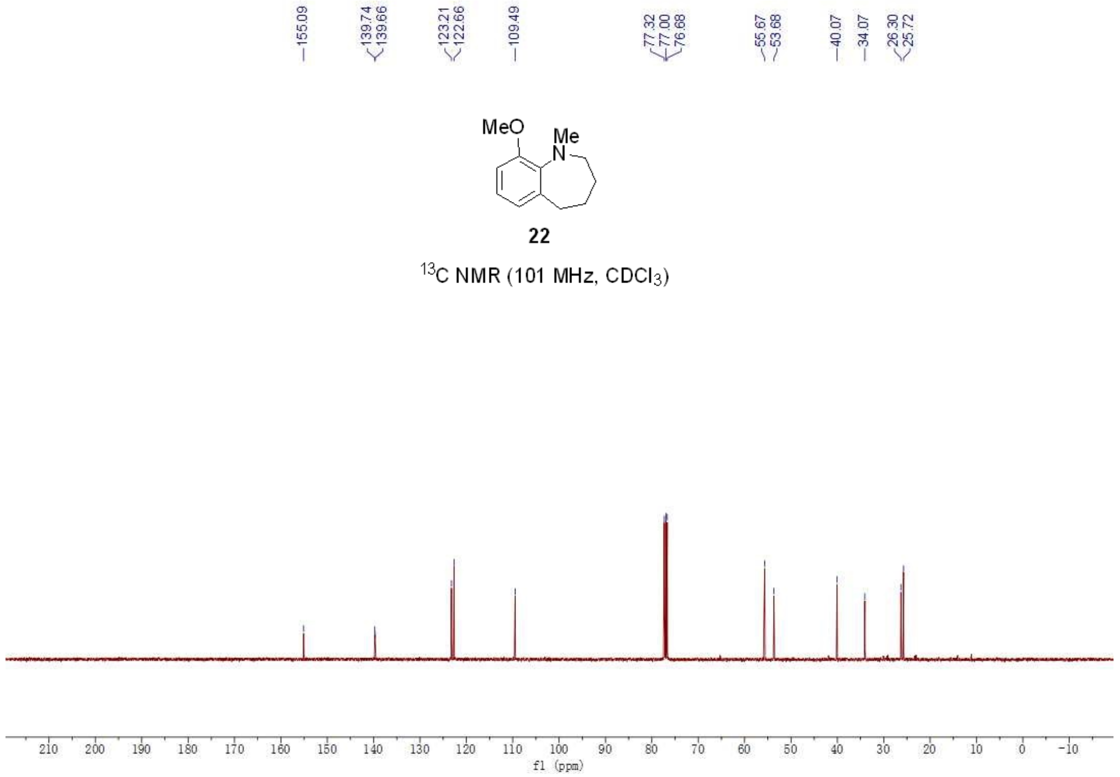

155.09
139.74
139.66
123.21
122.66
109.49
77.32
77.00
76.68
55.67
53.68
40.07
34.07
26.30
25.72
MeO
Me
N
22
^{13}C NMR (101 MHz, CDCl$_3$)
210 200 190 180 170 160 150 140 130 120 110 100 90 80 70 60 50 40 30 20 10 0 -10
f1 (ppm)

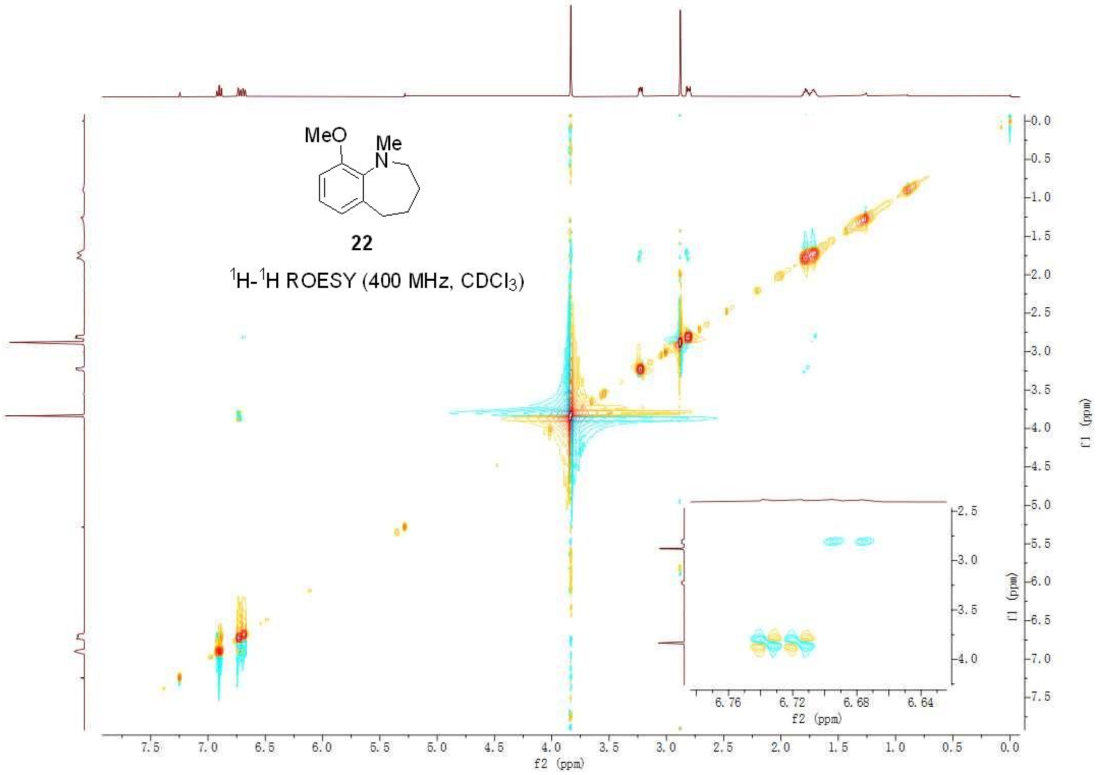

MeO
Me
N
22
^{1}H-^{1}H ROESY (400 MHz, CDCl$_3$)
7.5 7.0 6.5 6.0 5.5 5.0 4.5 4.0 3.5 3.0 2.5 2.0 1.5 1.0 0.5 0.0
f2 (ppm)
f1 (ppm)
6.76 6.72 6.68 6.64

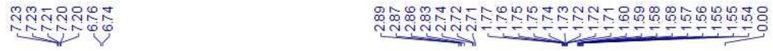

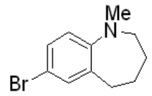

23

^{1}H NMR (400 MHz, $CDCl_3$)

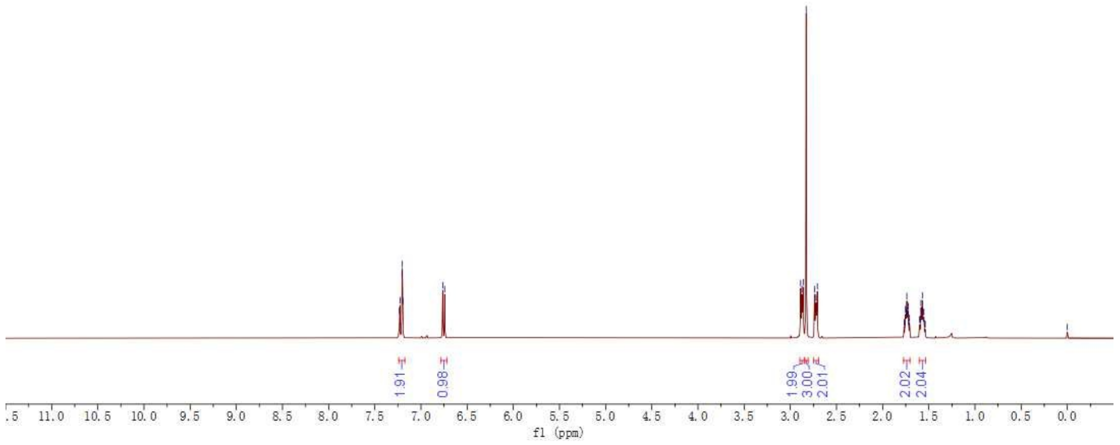

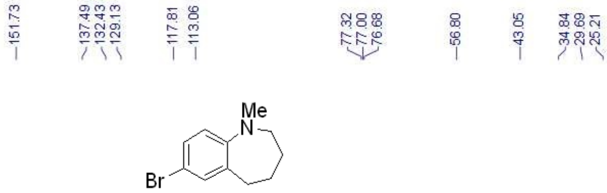

23

^{13}C NMR (101 MHz, $CDCl_3$)

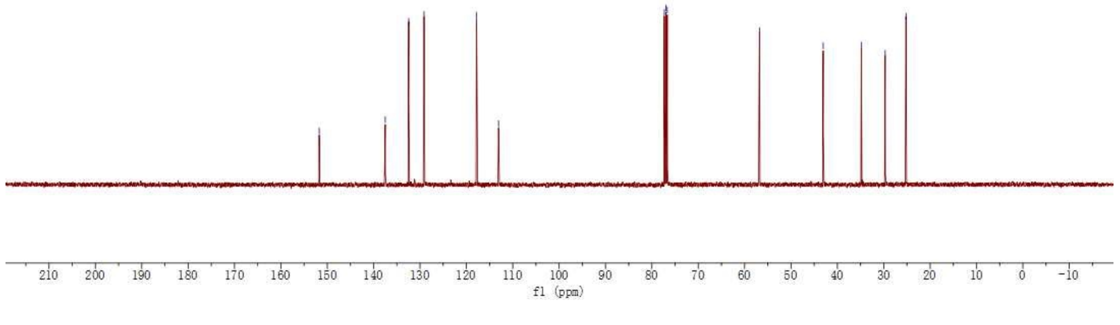

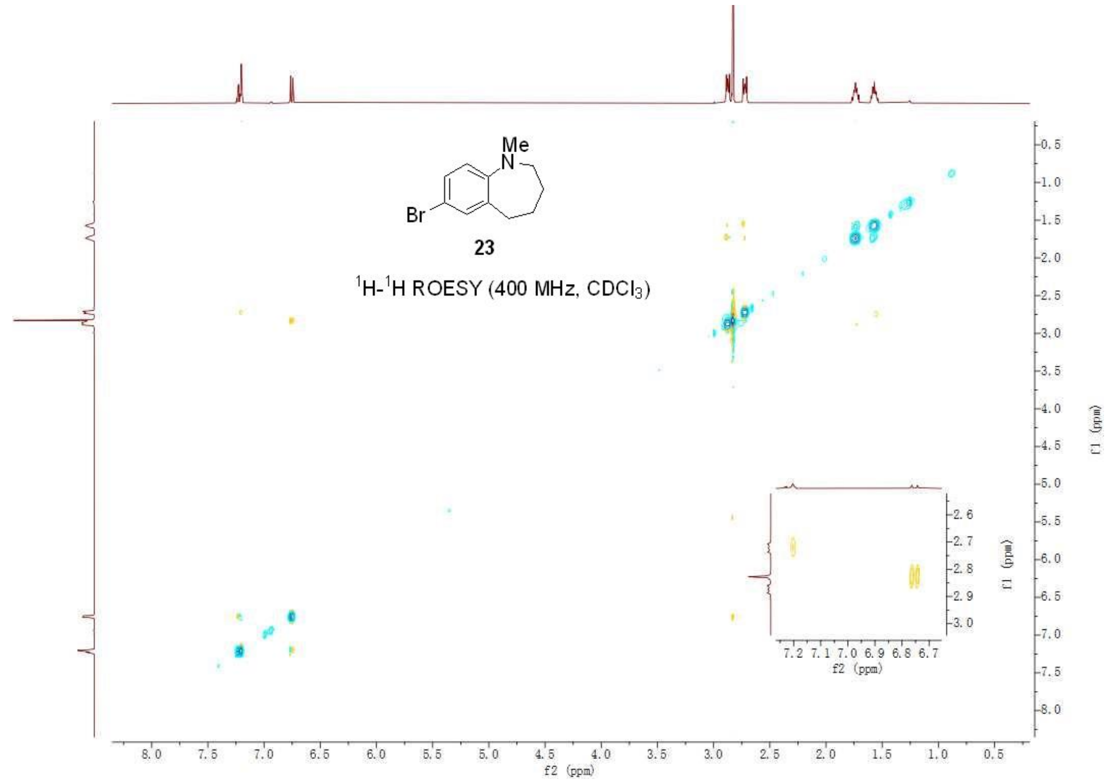

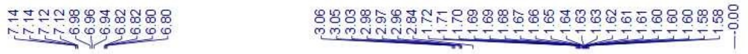

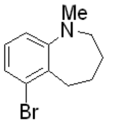

24

^{1}H NMR (400 MHz, CDCl$_3$)

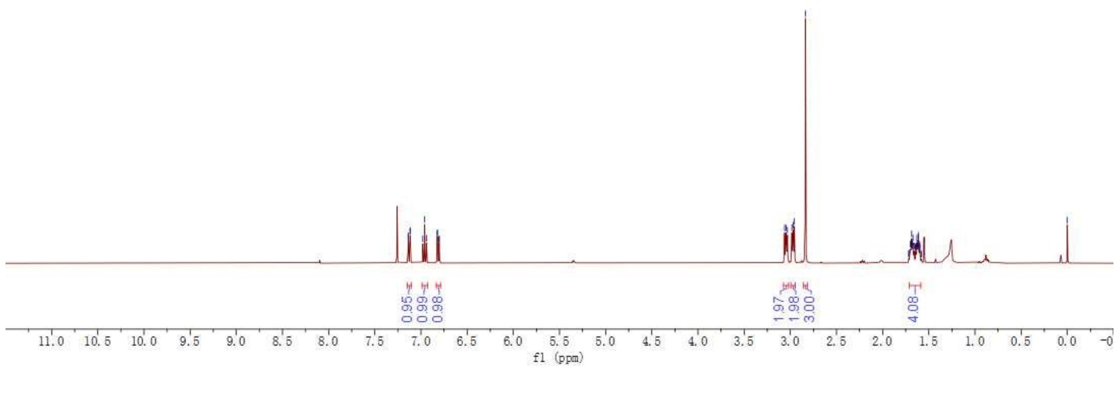

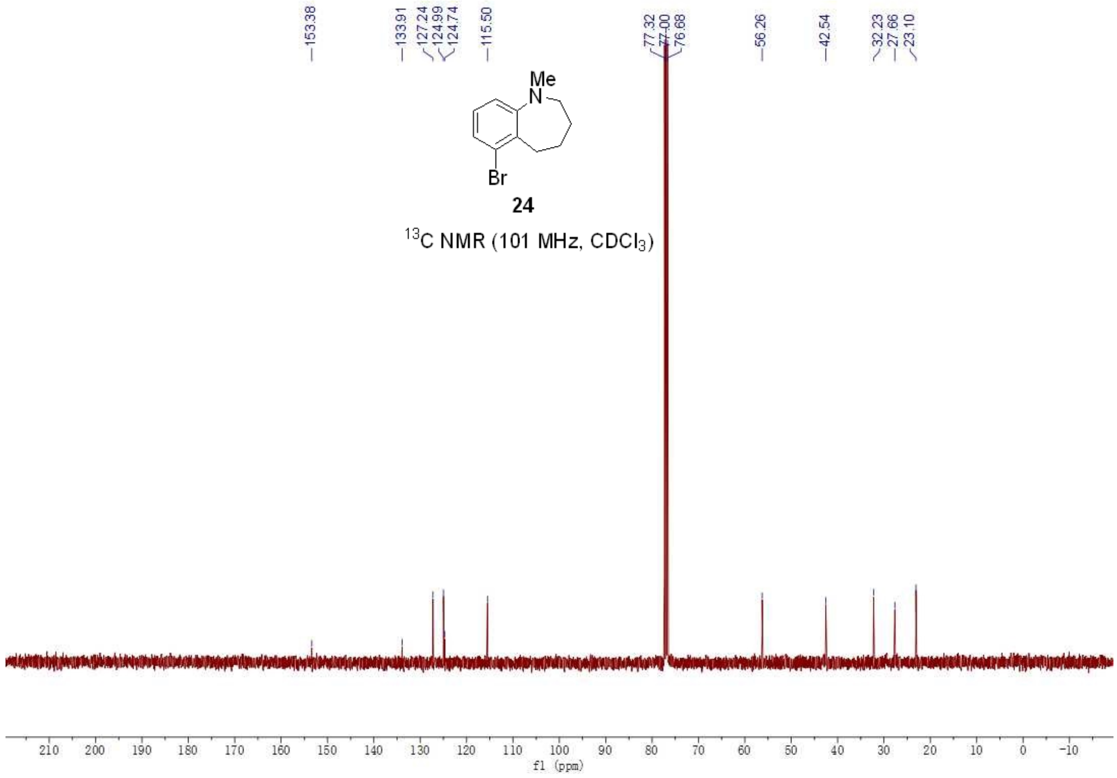

153.38
133.91
127.24
124.99
124.74
115.50
77.32
77.00
76.68
56.26
42.54
32.23
27.66
23.10
Me
N
Br
24
^{13}C NMR (101 MHz, CDCl$_3$)
f1 (ppm)

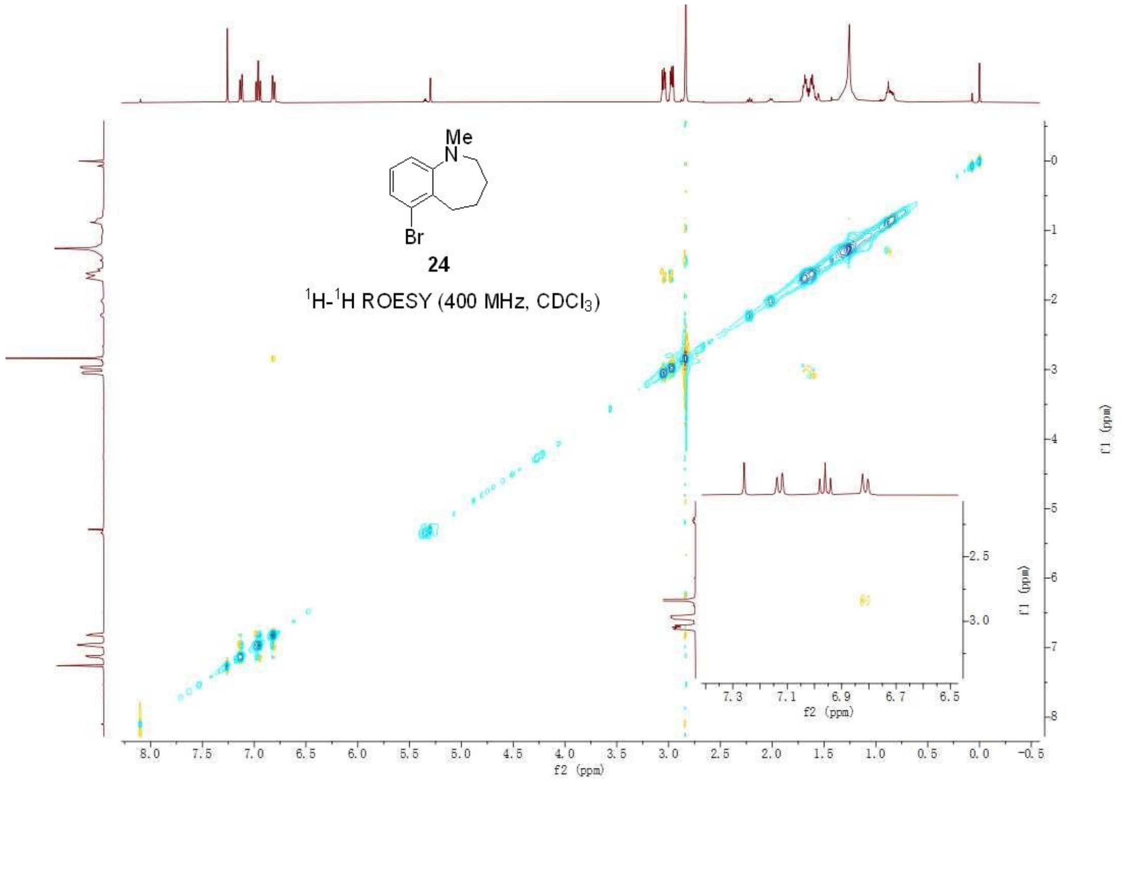

Me
N
Br
24
^{1}H-^{1}H ROESY (400 MHz, CDCl$_3$)
f2 (ppm)
f1 (ppm)

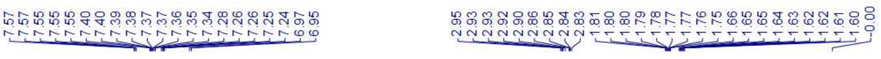

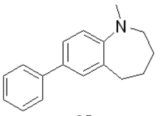

25

^{1}H NMR (400 MHz, $CDCl_3$)

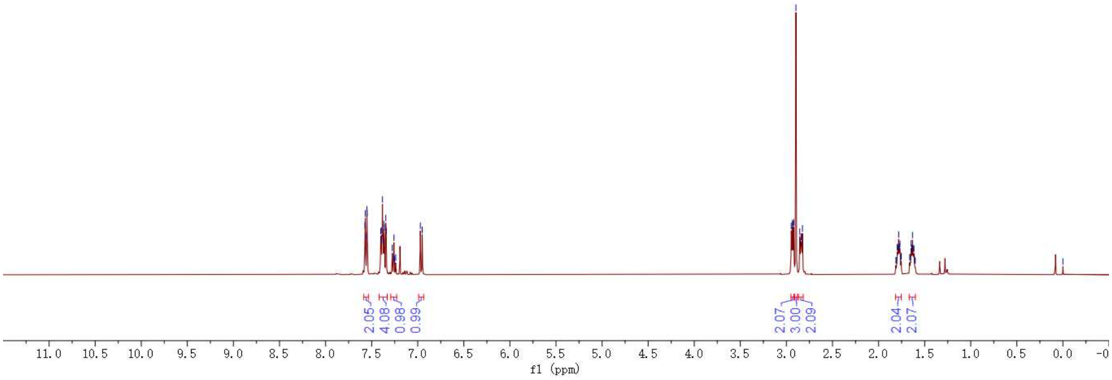

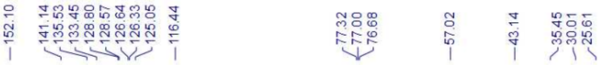

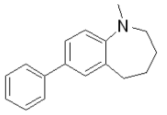

25

^{13}C NMR (101 MHz, $CDCl_3$)

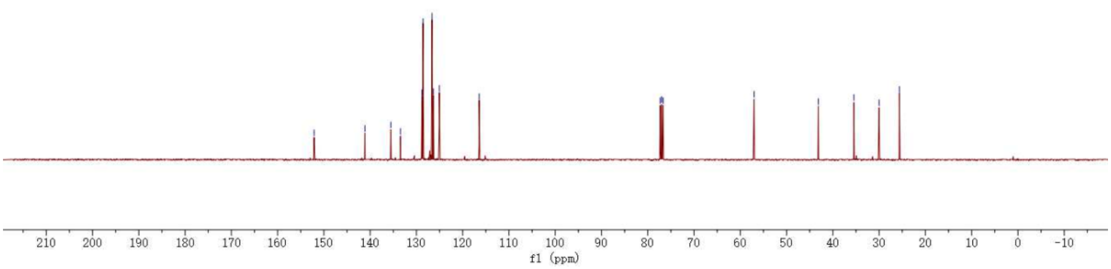

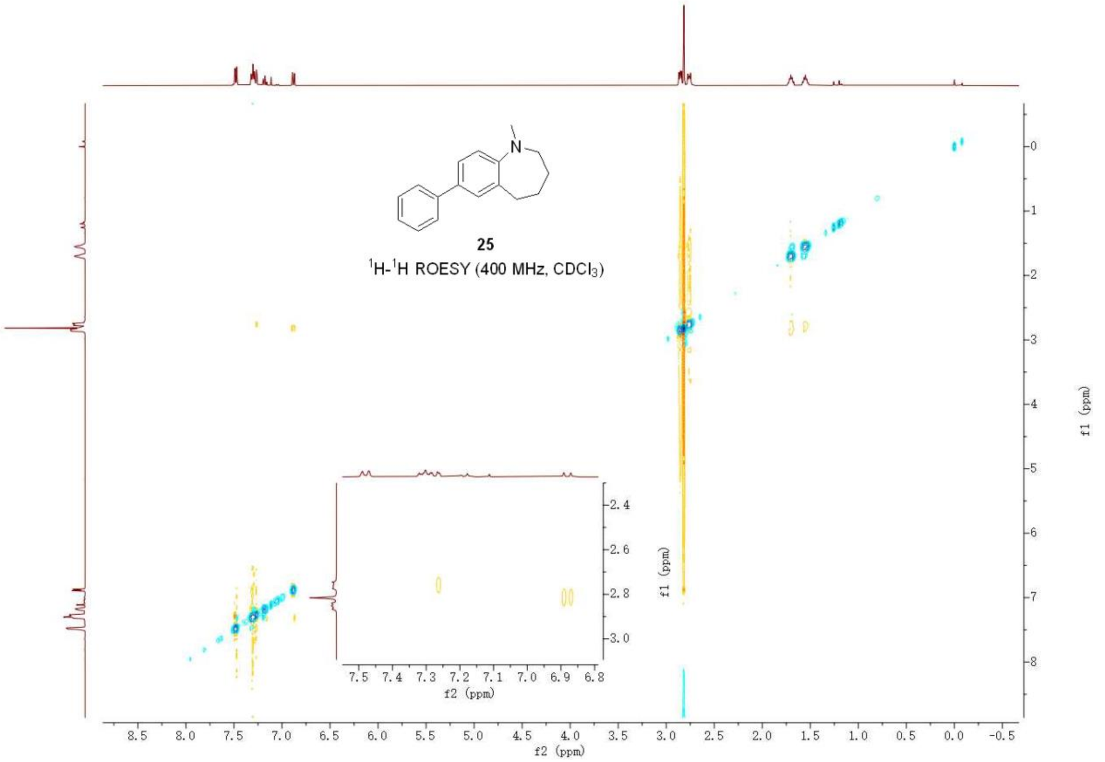
25
1H-1H ROESY (400 MHz, CDCl3)
f1 (ppm)
f2 (ppm)

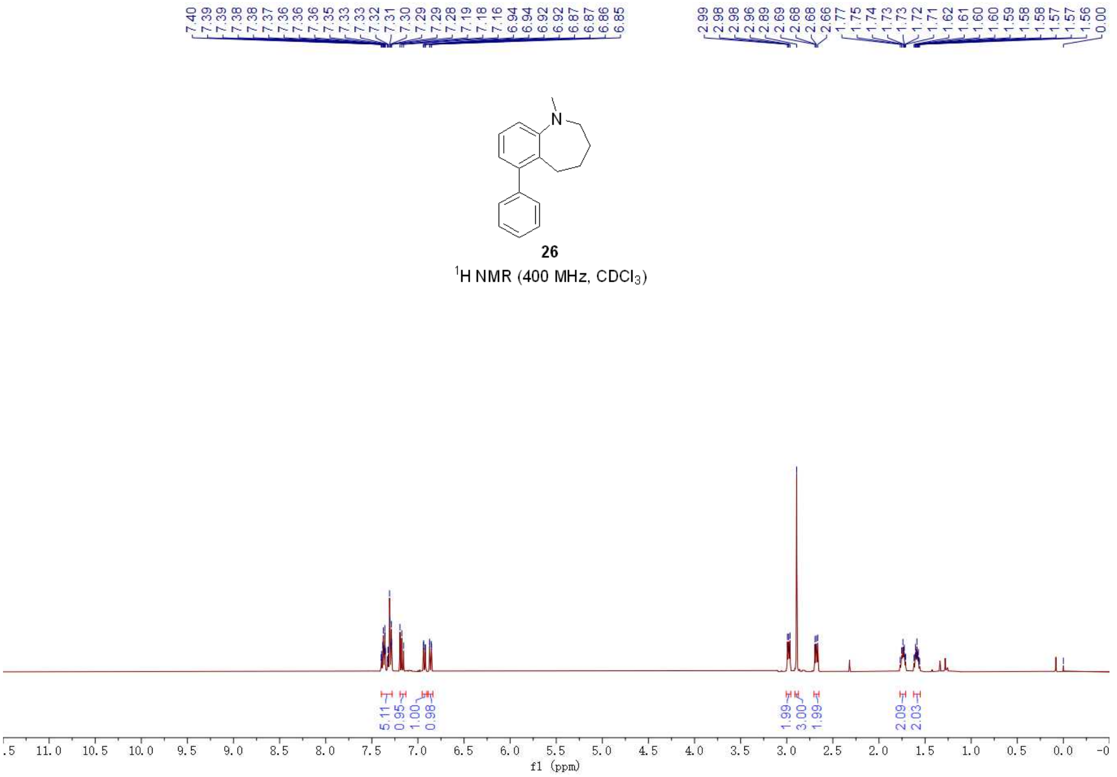
7.40 7.39 7.38 7.37 7.36 7.36 7.35 7.33 7.32 7.31 7.30 7.29 7.28 7.19 7.18 7.16 6.94 6.92 6.92 6.87 6.86 6.85
2.99 2.98 2.98 2.96 2.89 2.69 2.68 2.68 2.66 1.77 1.75 1.74 1.73 1.73 1.72 1.71 1.62 1.61 1.60 1.59 1.58 1.58 1.57 1.56 0.00
26
1H NMR (400 MHz, CDCl3)
5.11 0.95 1.00 0.98
1.99 3.00 1.99
2.09 2.03
f1 (ppm)

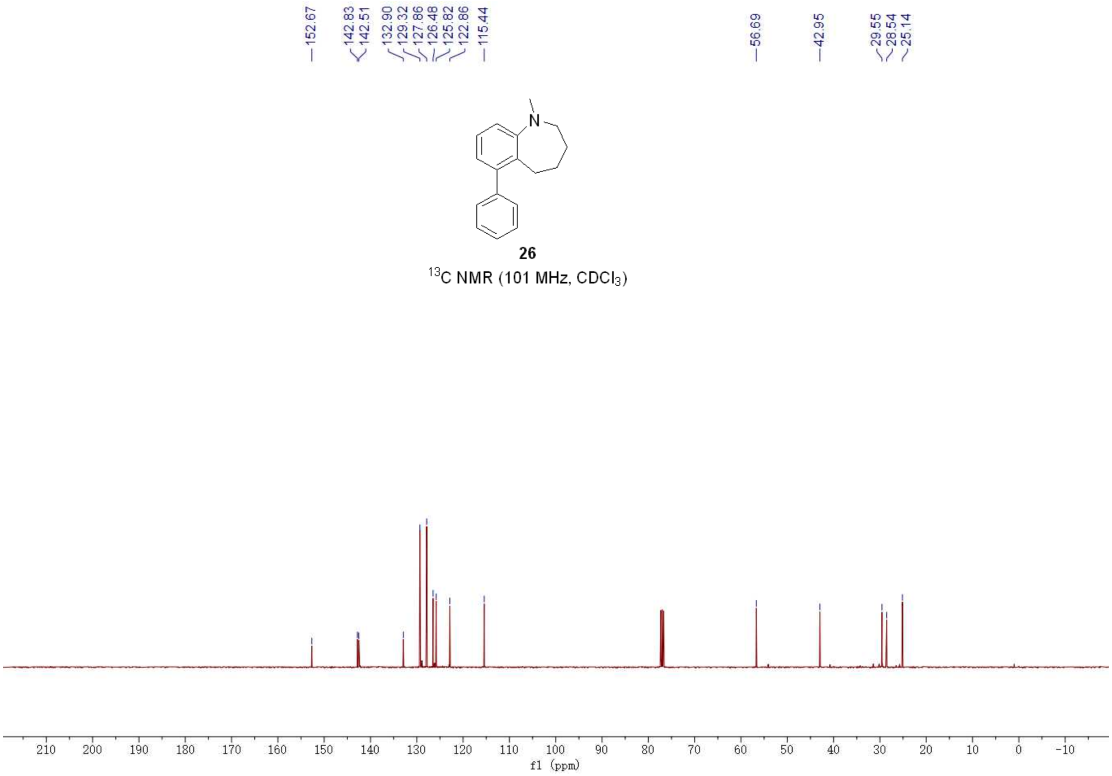

26

^{13}C NMR (101 MHz, $CDCl_3$)

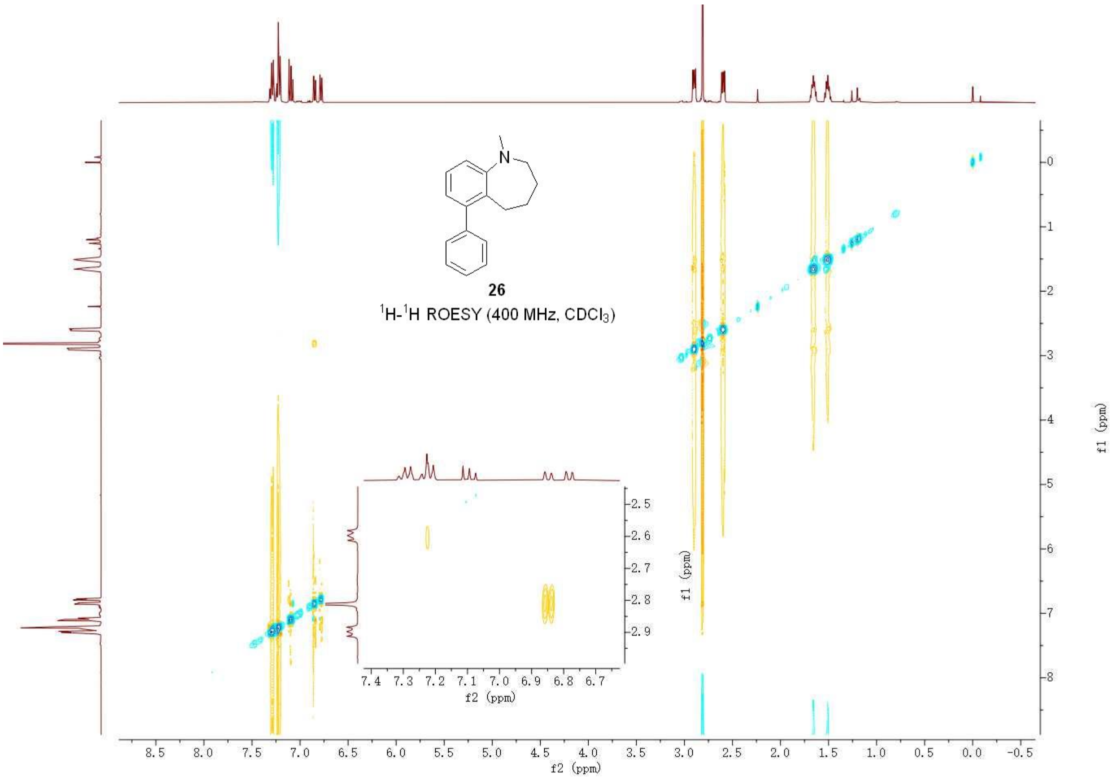

26

^{1}H-^{1}H ROESY (400 MHz, $CDCl_3$)

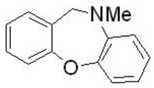

27

^{1}H NMR (400 MHz, $CDCl_3$)

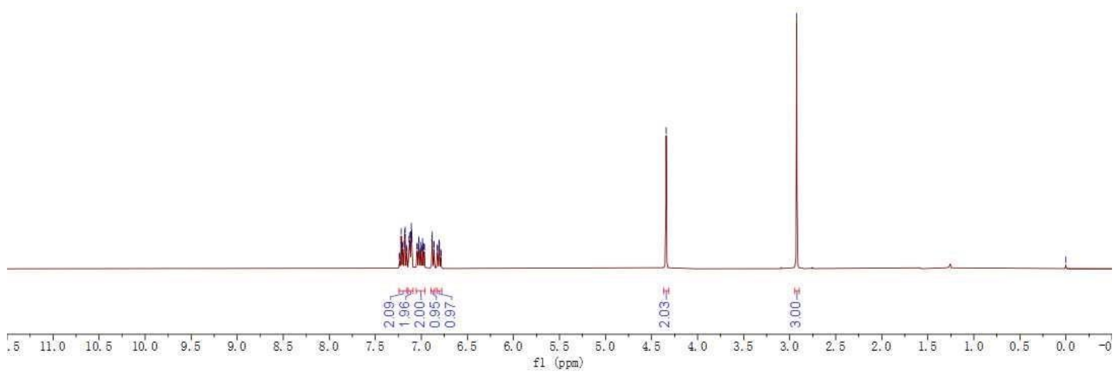

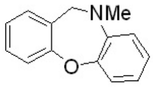

27

^{13}C NMR (101 MHz, $CDCl_3$)

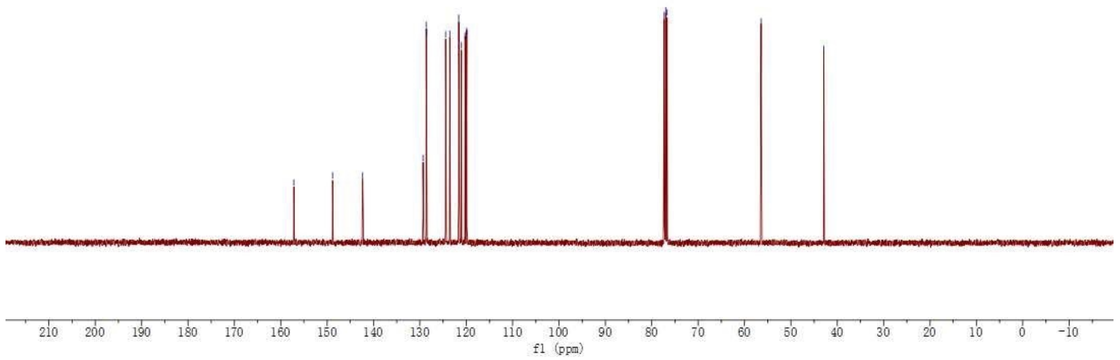

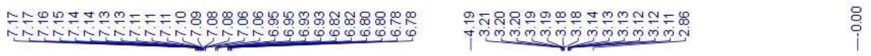

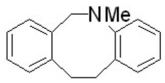

28

^{1}H NMR (400 MHz, $CDCl_3$)

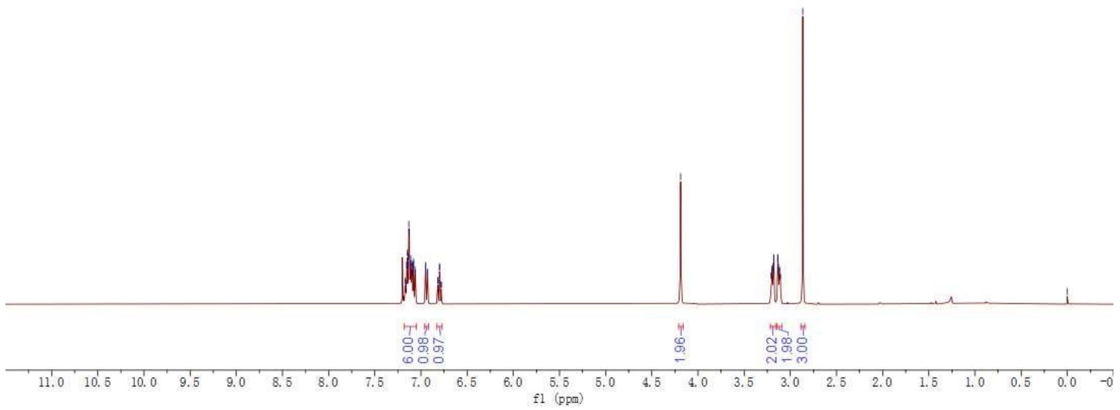

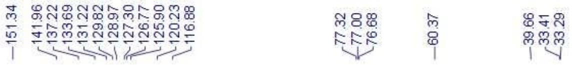

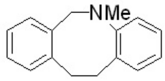

28

^{13}C NMR (101 MHz, $CDCl_3$)

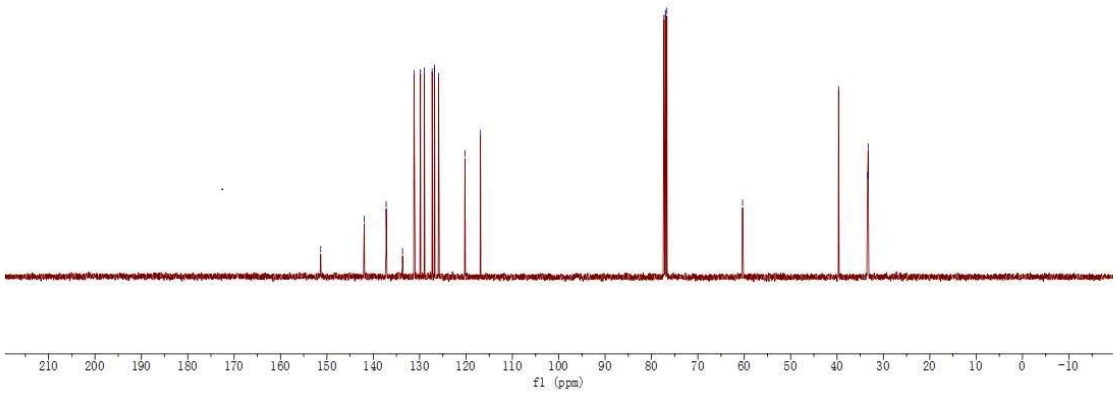

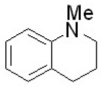

29

^{1}H NMR (400 MHz, $CDCl_3$)

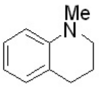

29

^{13}C NMR (101 MHz, $CDCl_3$)

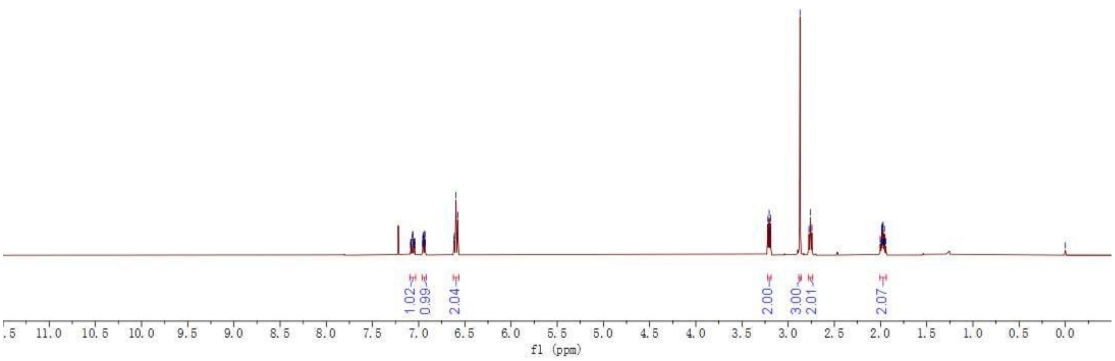

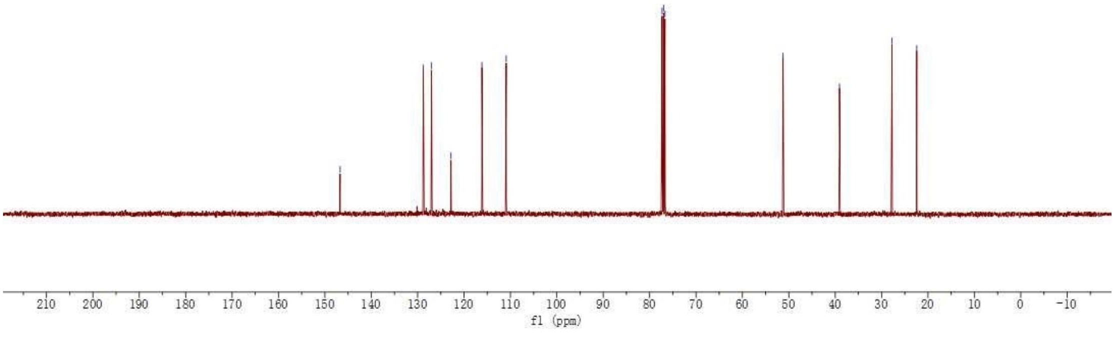

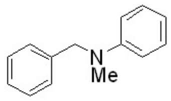

30

^{1}H NMR (400 MHz, $CDCl_3$)

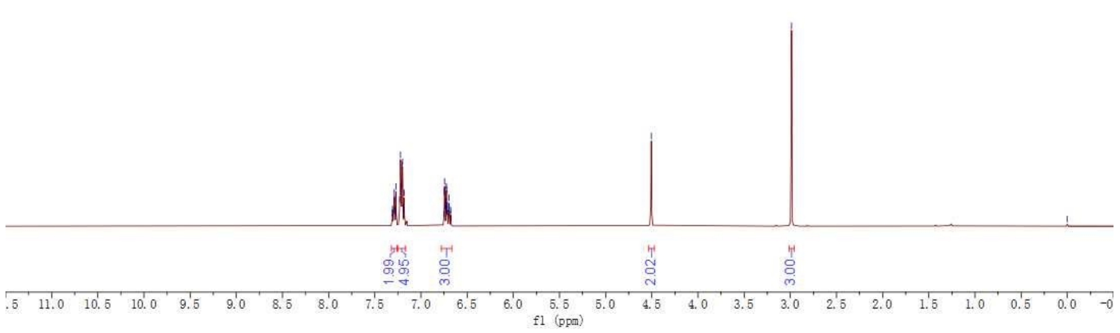

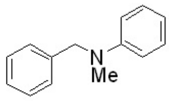

30

^{13}C NMR (101 MHz, $CDCl_3$)

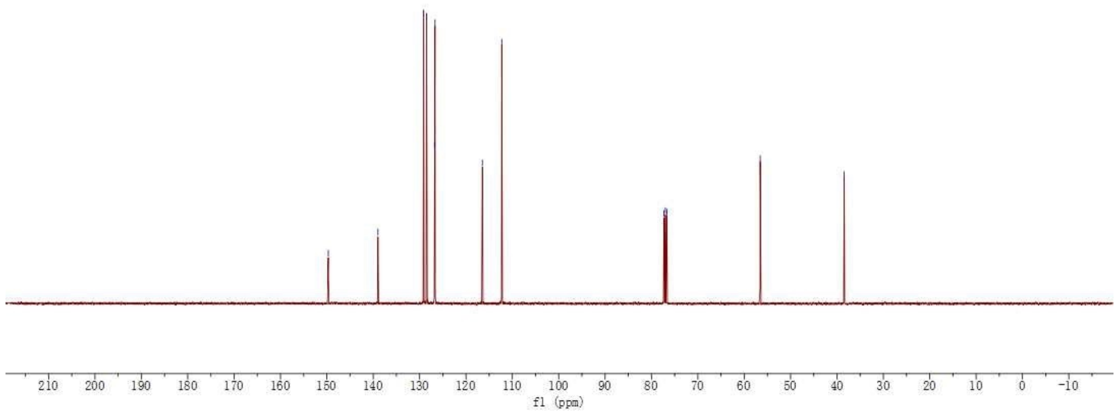

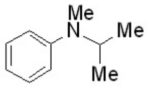

31

^{1}H NMR (400 MHz, $CDCl_3$)

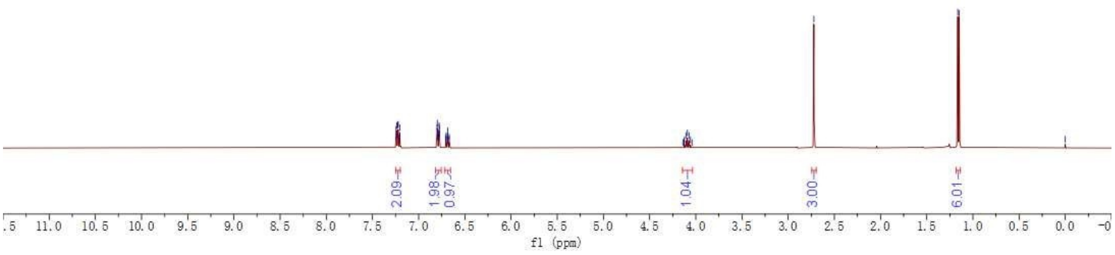

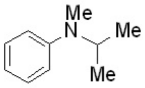

31

^{13}C NMR (101 MHz, $CDCl_3$)

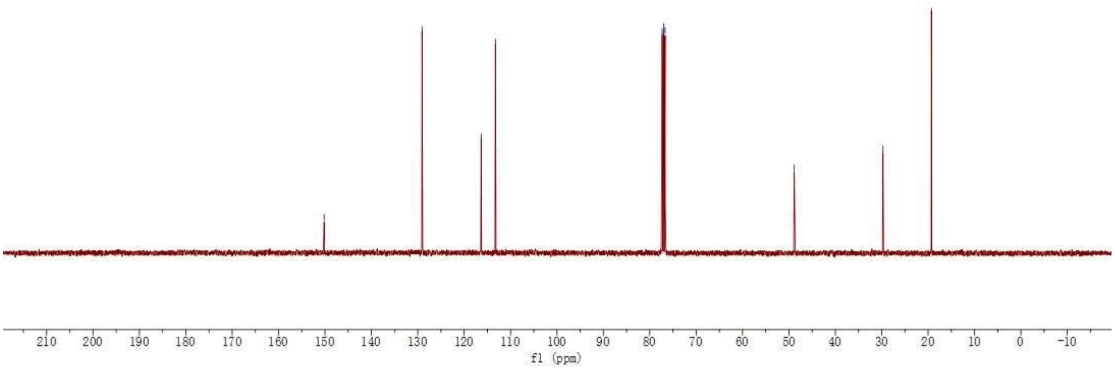

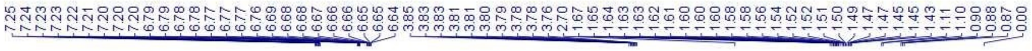

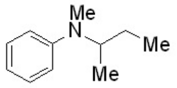

32

^{1}H NMR (400 MHz, $CDCl_3$)

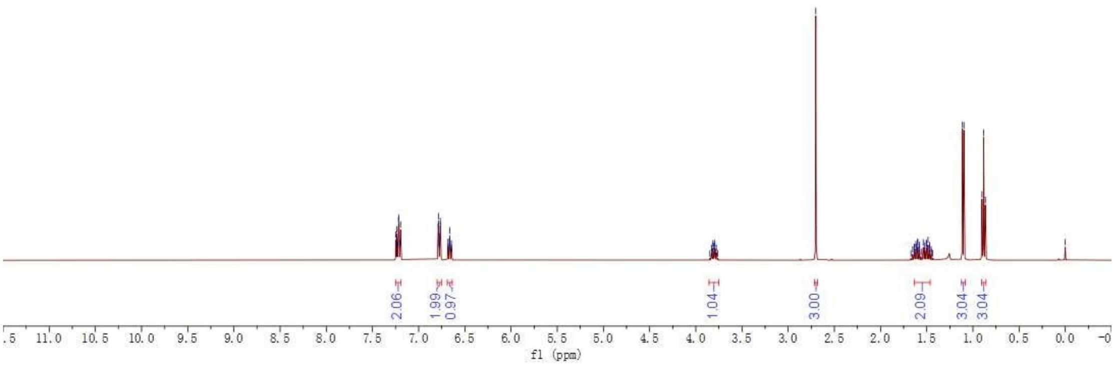

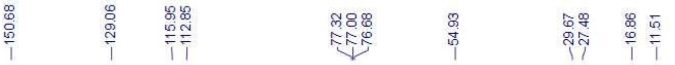

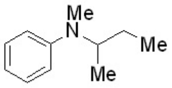

32

^{13}C NMR (101 MHz, $CDCl_3$)

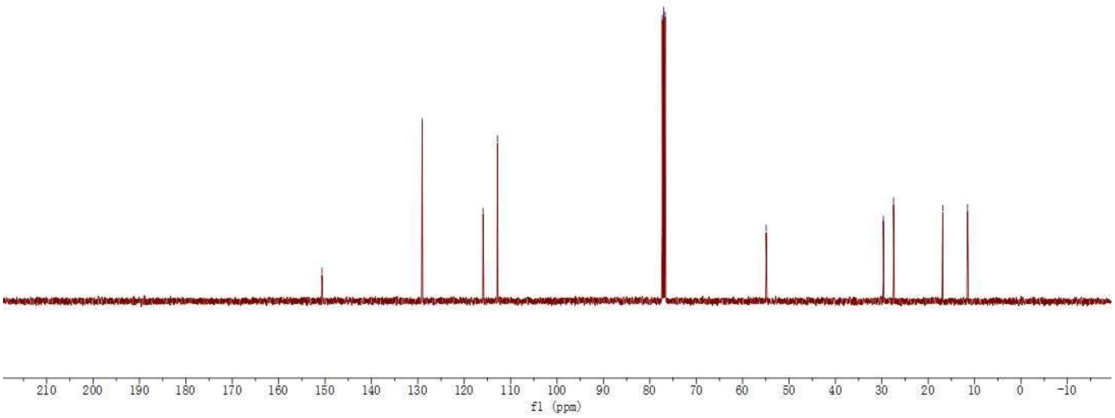

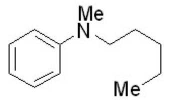

33

^{1}H NMR (400 MHz, $CDCl_3$)

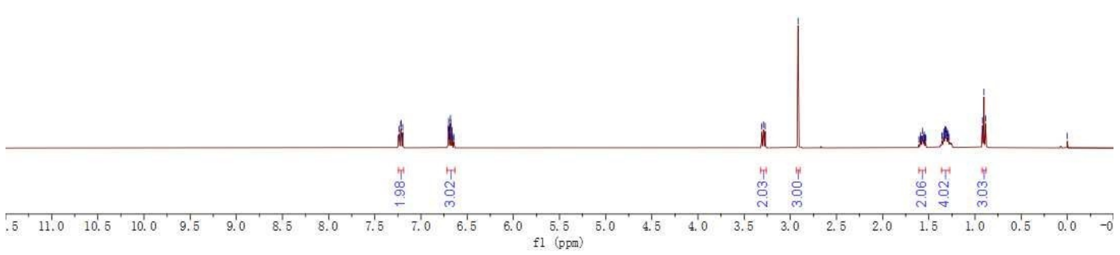

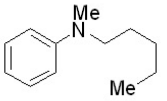

33

^{13}C NMR (101 MHz, $CDCl_3$)

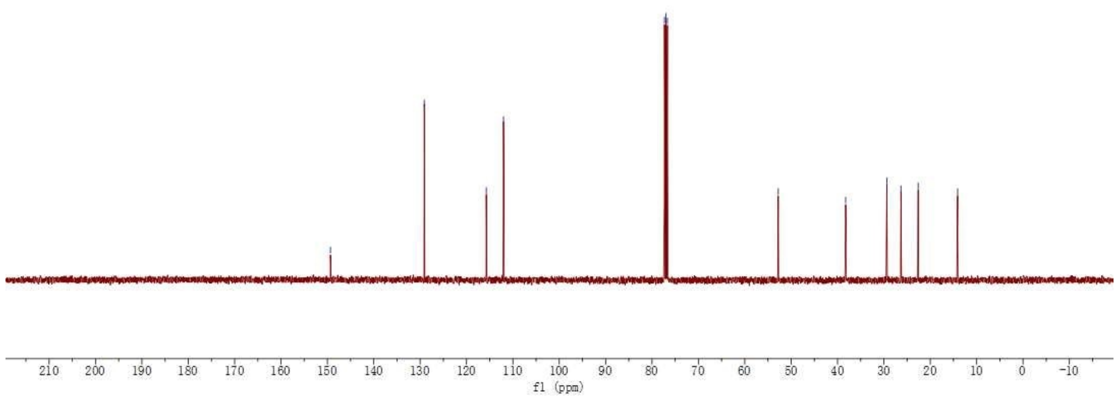

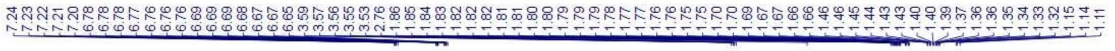

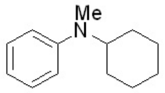

34

^{1}H NMR (400 MHz, $CDCl_3$)

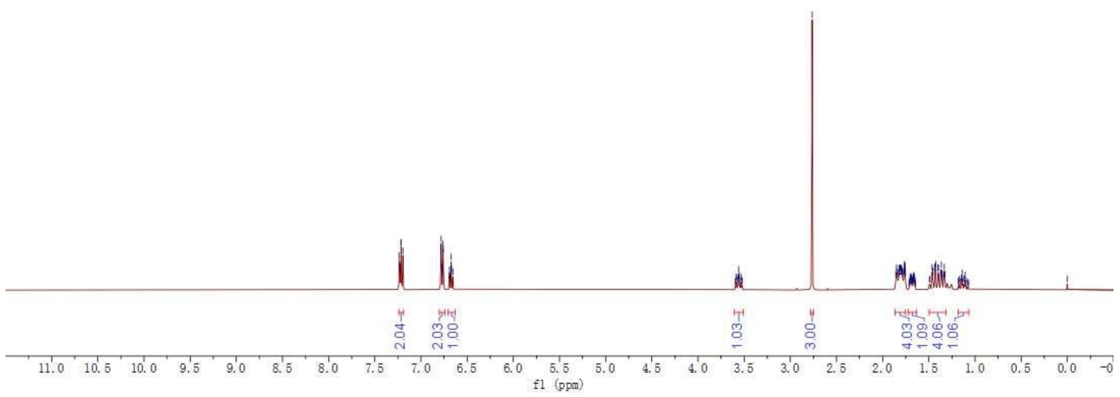

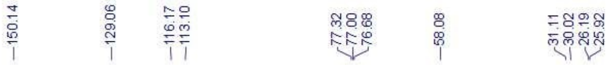

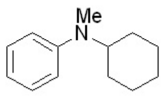

34

^{13}C NMR (101 MHz, $CDCl_3$)

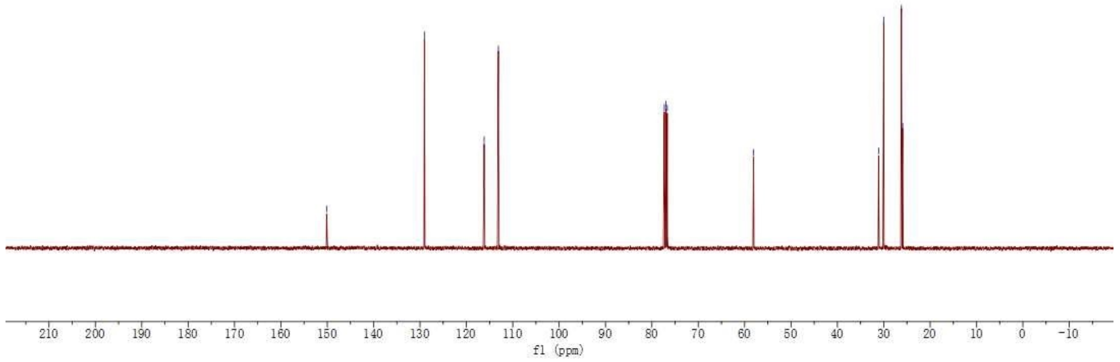

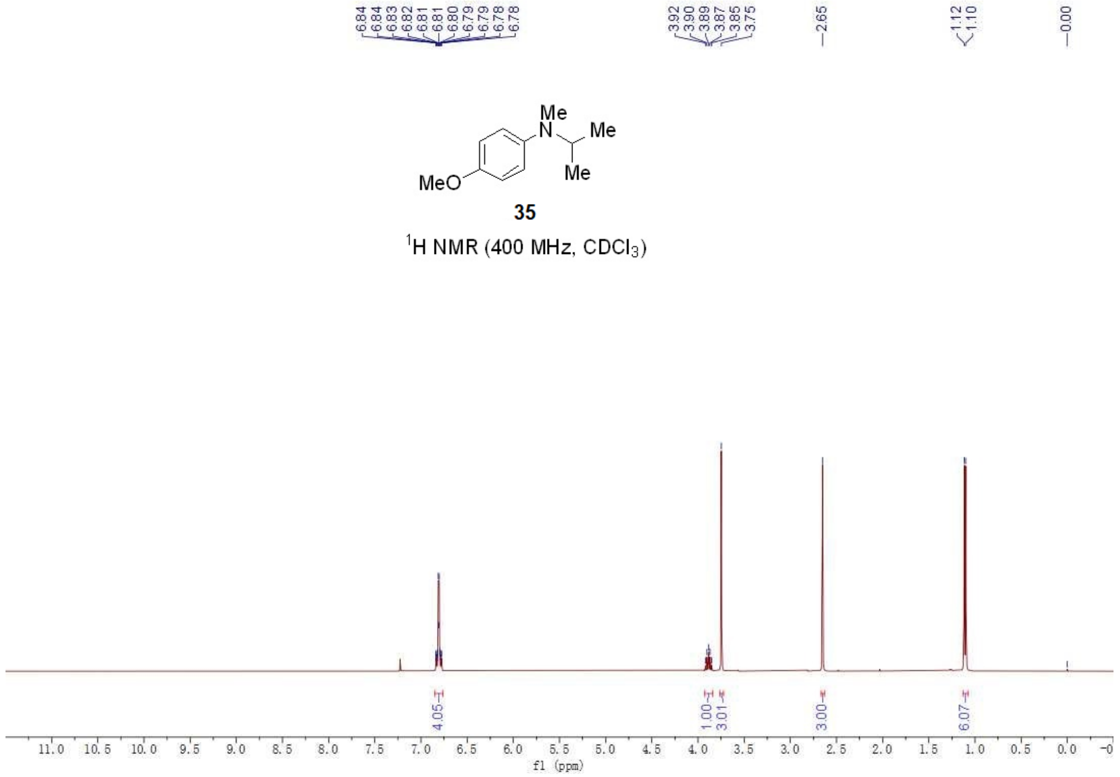

35

^{1}H NMR (400 MHz, $CDCl_3$)

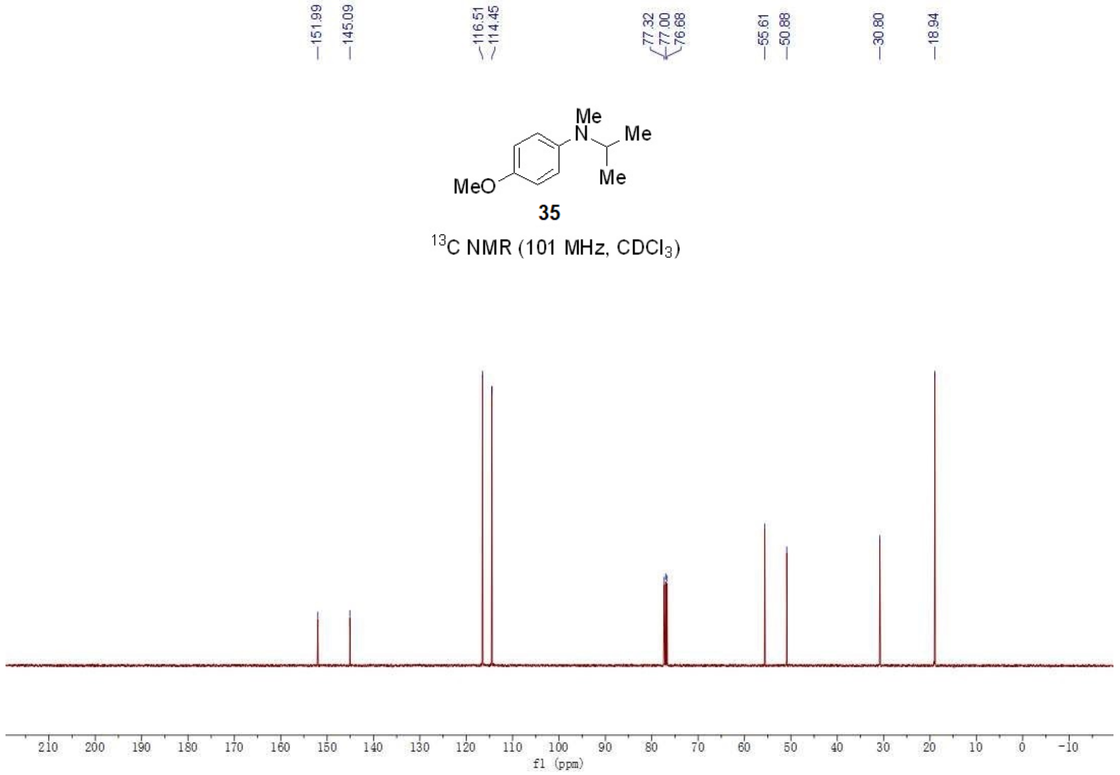

35

^{13}C NMR (101 MHz, $CDCl_3$)

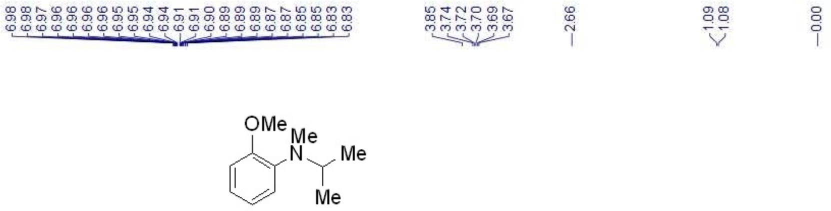

36

^{1}H NMR (400 MHz, $CDCl_3$)

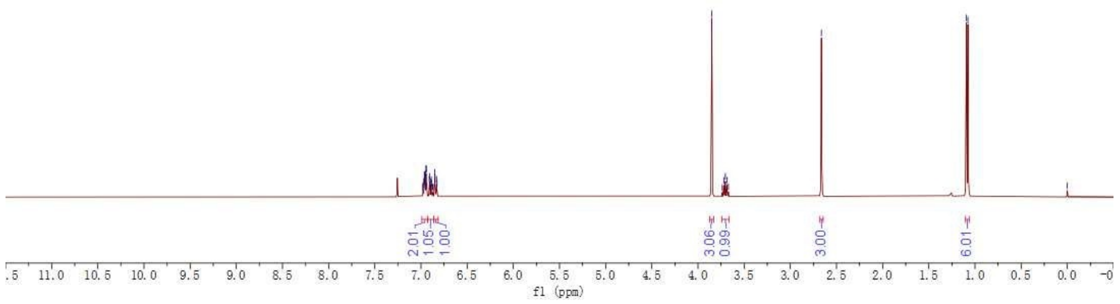

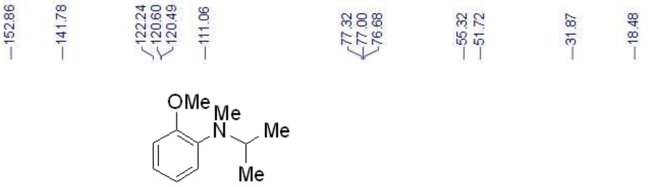

36

^{13}C NMR (101 MHz, $CDCl_3$)

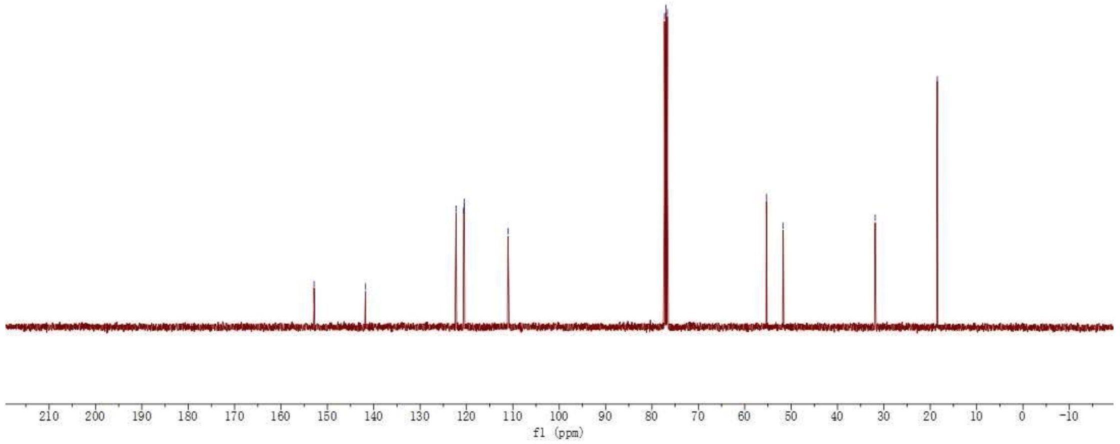

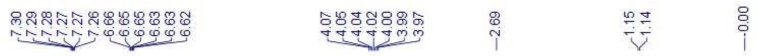

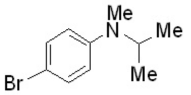

37

^{1}H NMR (400 MHz, $CDCl_3$)

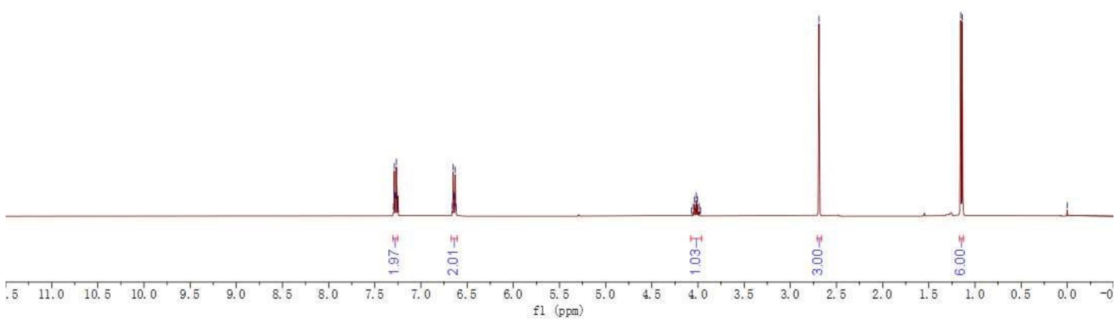

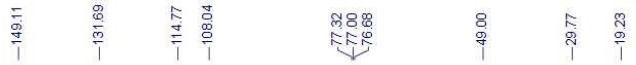

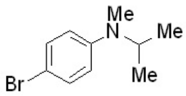

37

^{13}C NMR (101 MHz, $CDCl_3$)

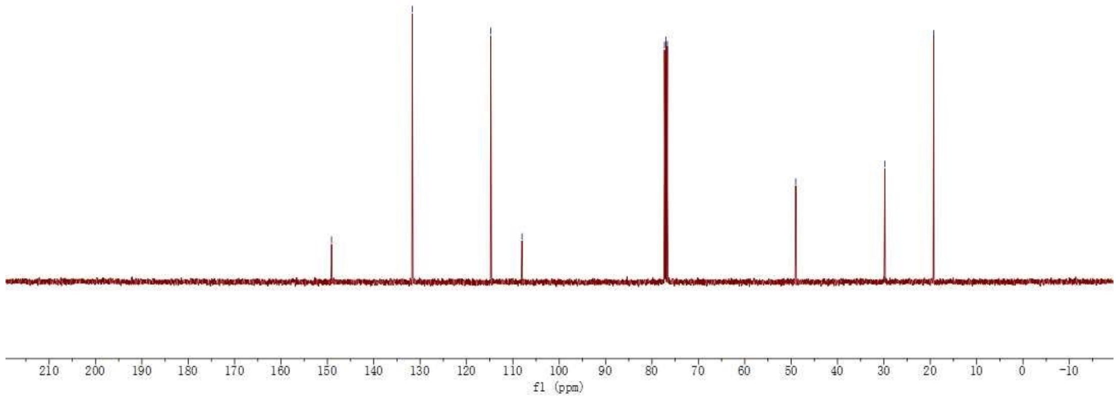

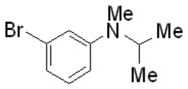

38

^{1}H NMR (400 MHz, $CDCl_3$)

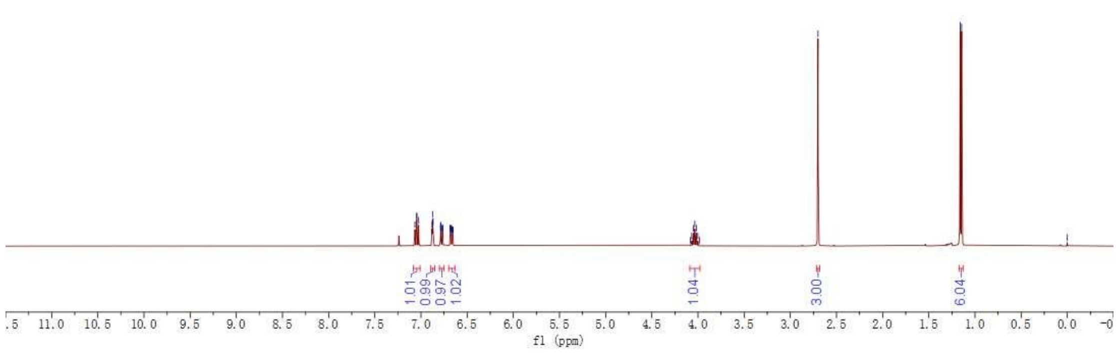

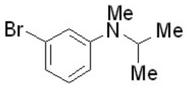

38

^{13}C NMR (101 MHz, $CDCl_3$)

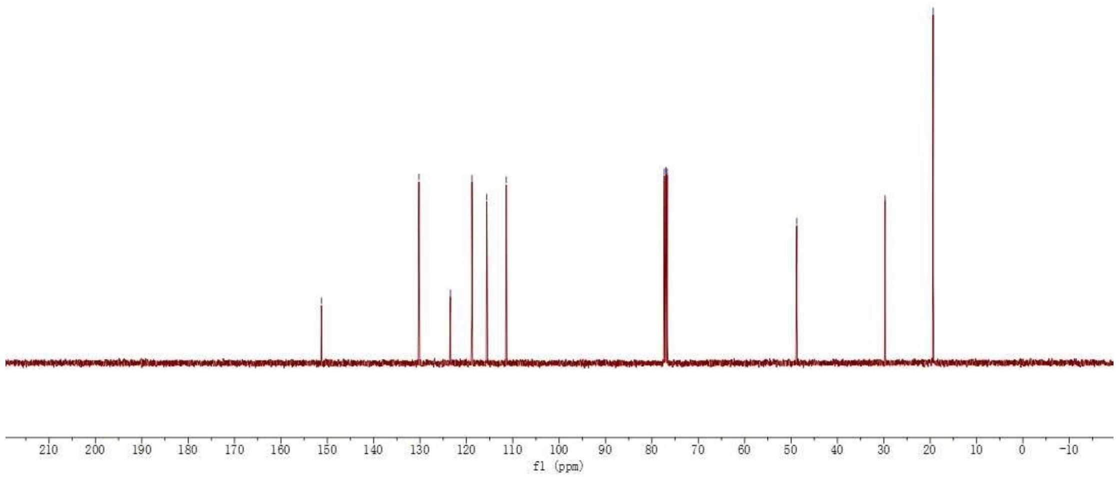

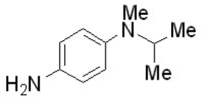

39

^{1}H NMR (400 MHz, $CDCl_3$)

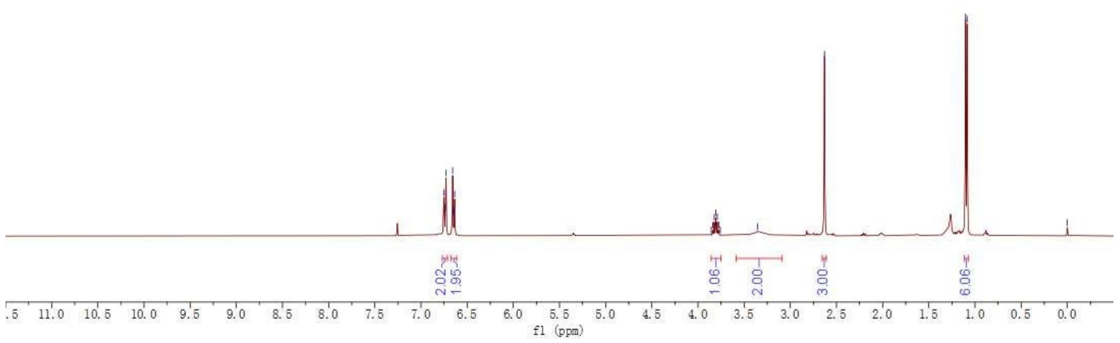

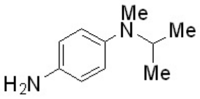

39

^{13}C NMR (101 MHz, $CDCl_3$)

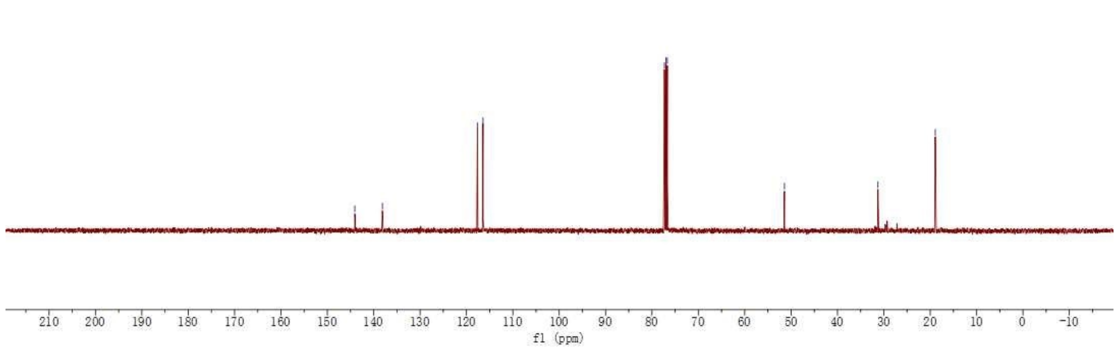

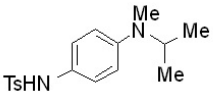

40

^{1}H NMR (400 MHz, $CDCl_3$)

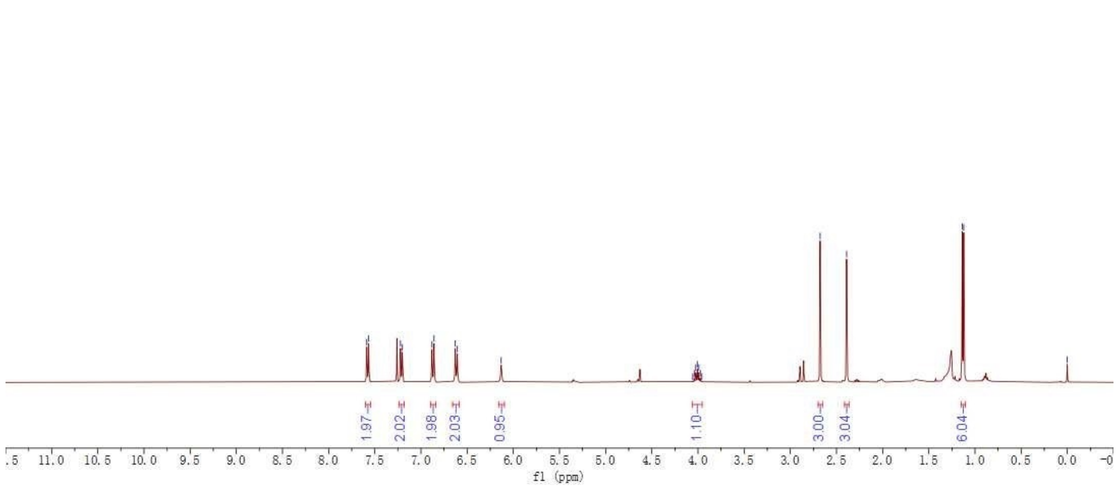

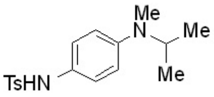

40

^{13}C NMR (101 MHz, $CDCl_3$)

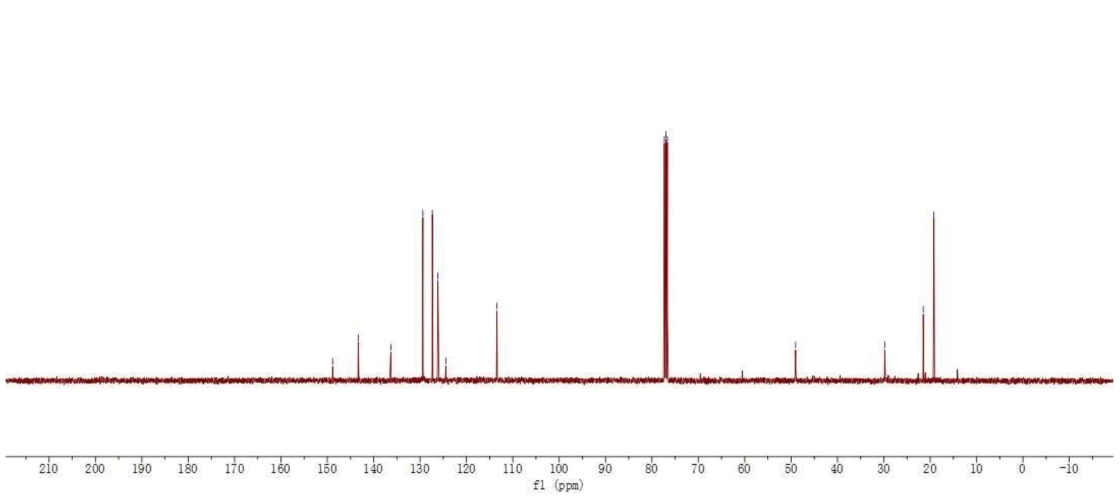

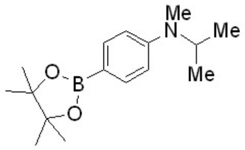

41

^{1}H NMR (400 MHz, $CDCl_3$)

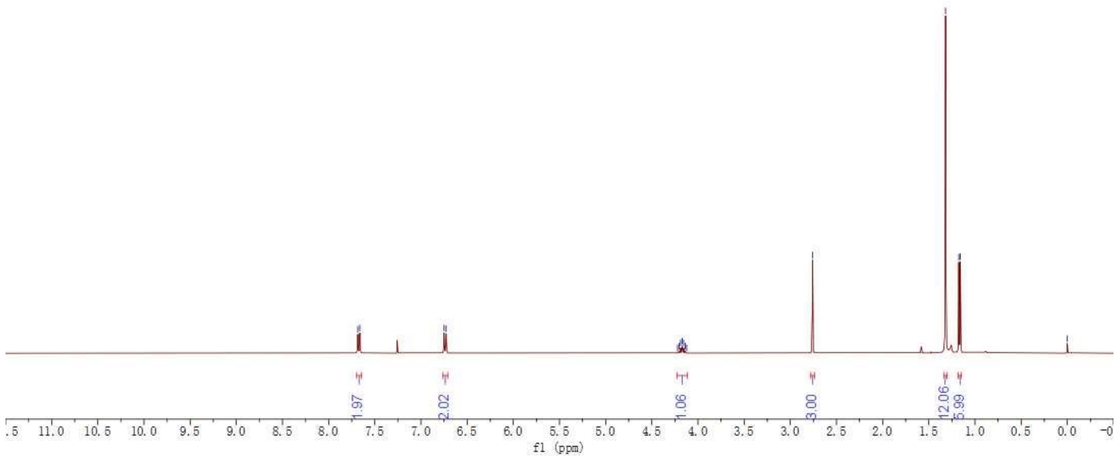

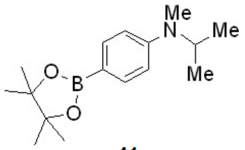

41

^{13}C NMR (101 MHz, $CDCl_3$)

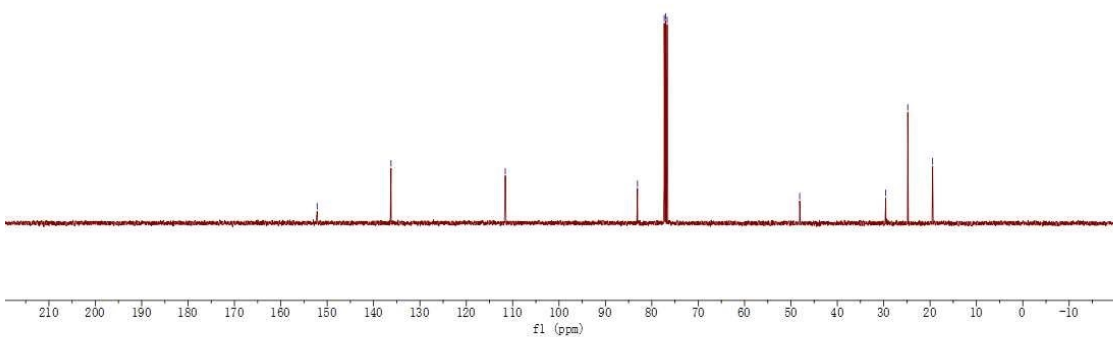

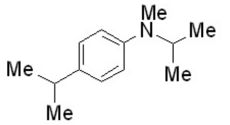

42

^{1}H NMR (400 MHz, $CDCl_3$)

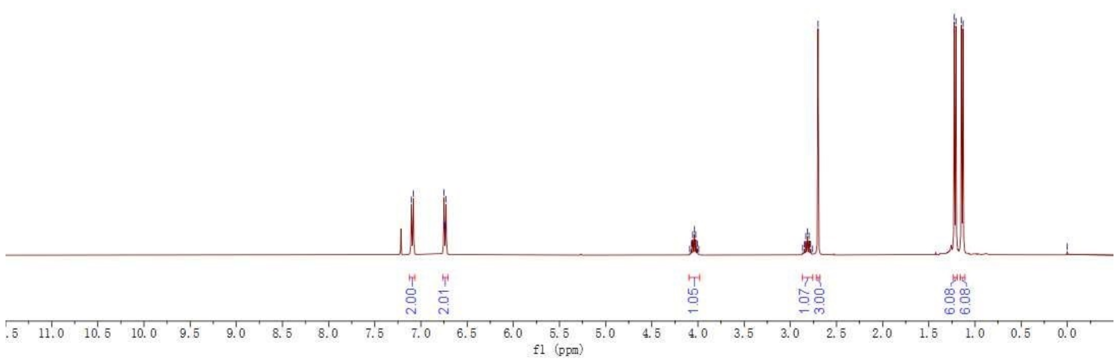

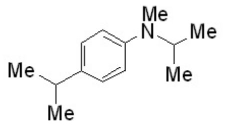

42

^{13}C NMR (101 MHz, $CDCl_3$)

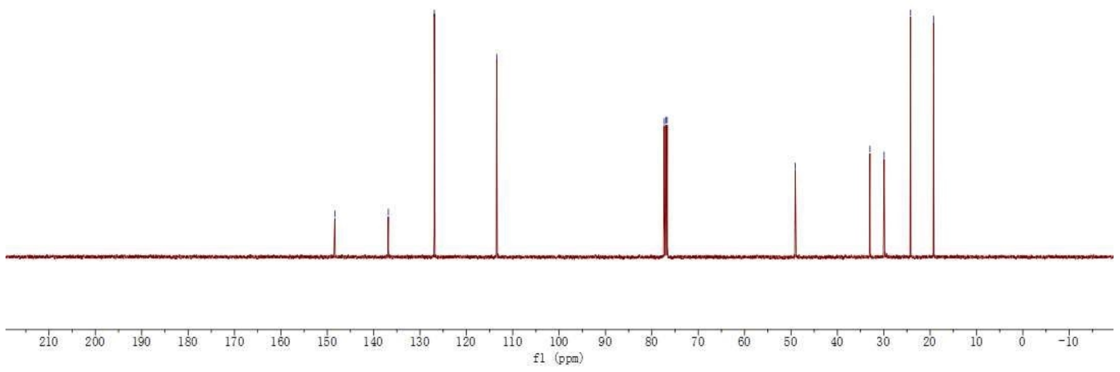

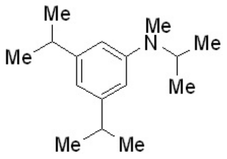

43

^{1}H NMR (400 MHz, $CDCl_3$)

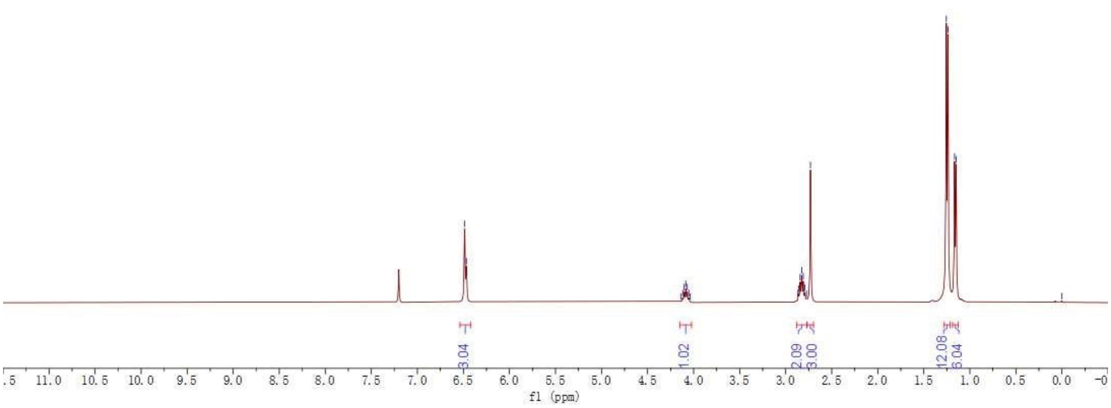

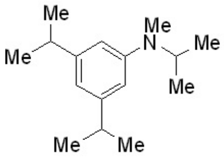

43

^{13}C NMR (101 MHz, $CDCl_3$)

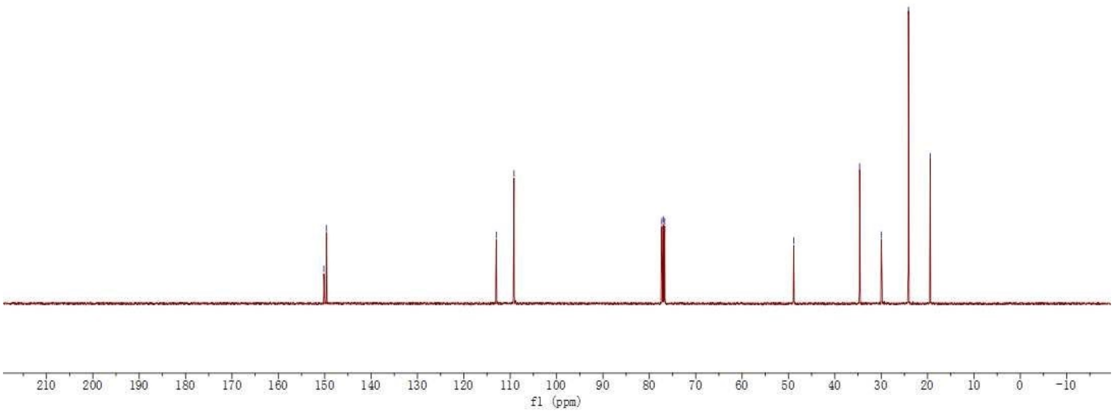

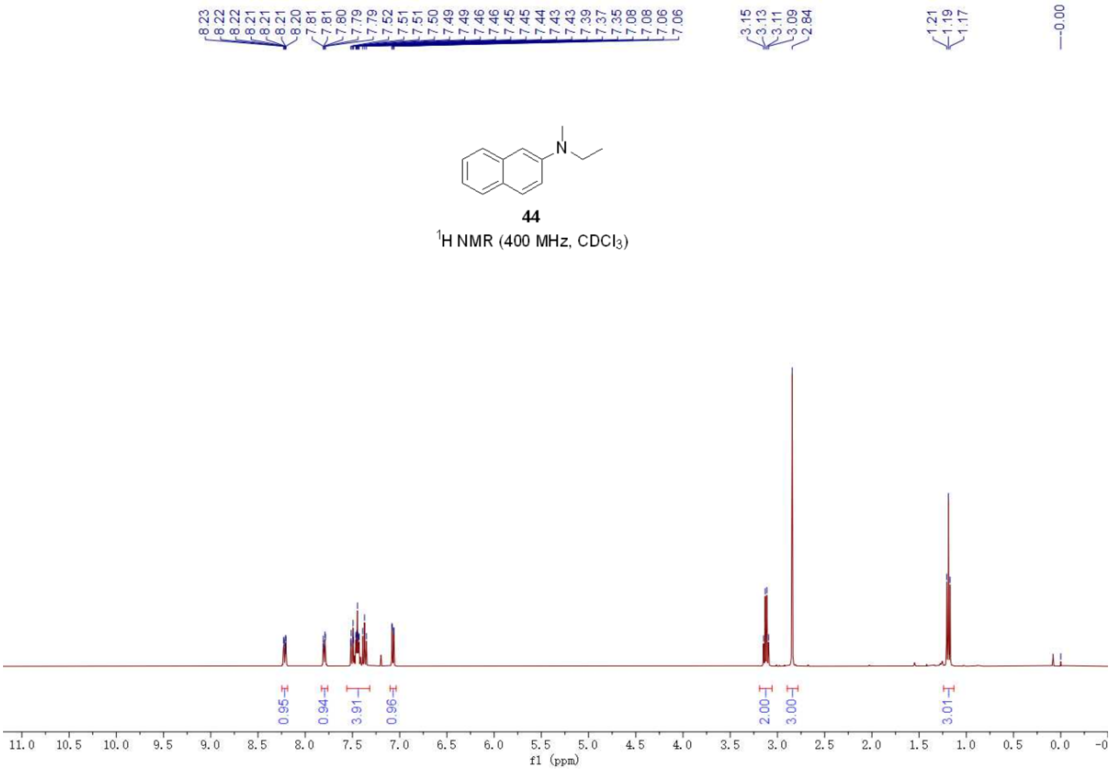

44

^{1}H NMR (400 MHz, CDCl$_3$)

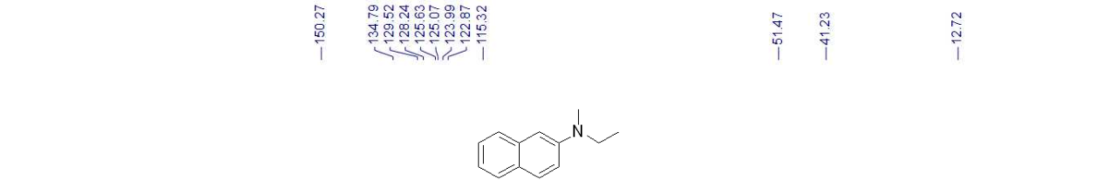

44

^{13}C NMR (101 MHz, CDCl$_3$)

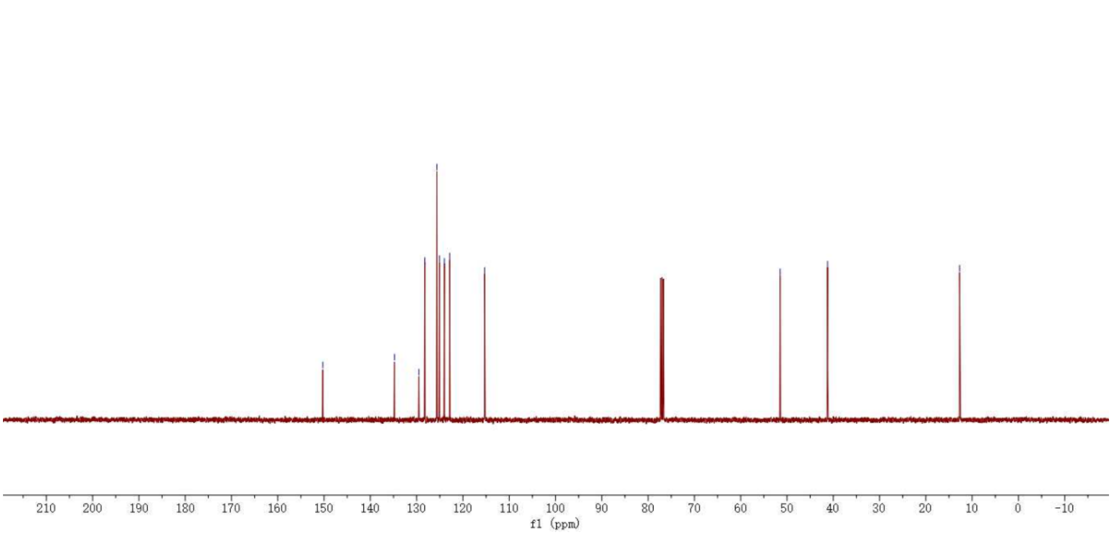

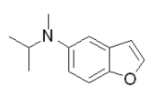

45

^{1}H NMR (400 MHz, $CDCl_3$)

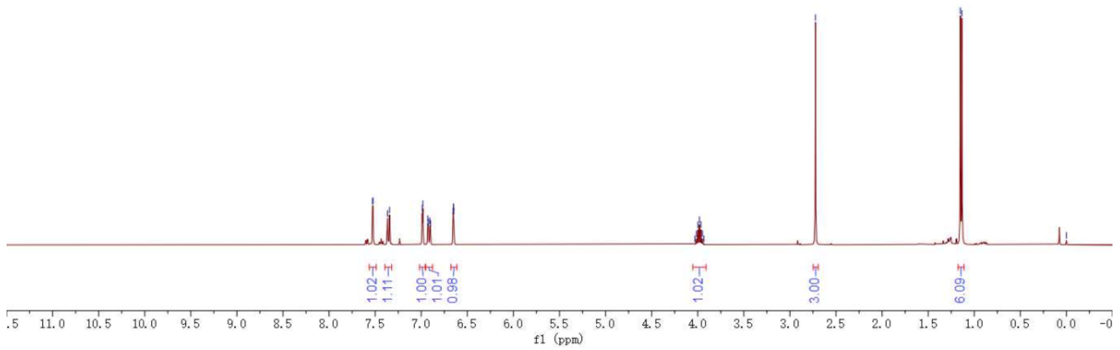

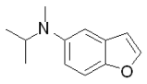

45

^{13}C NMR (101 MHz, $CDCl_3$)

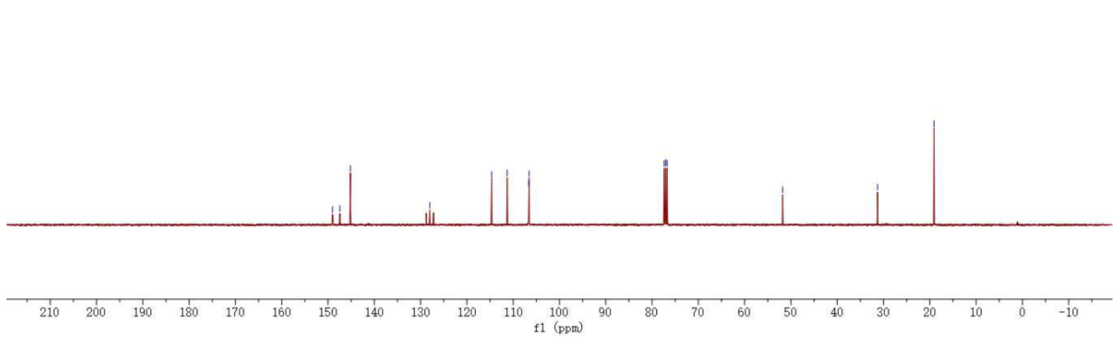

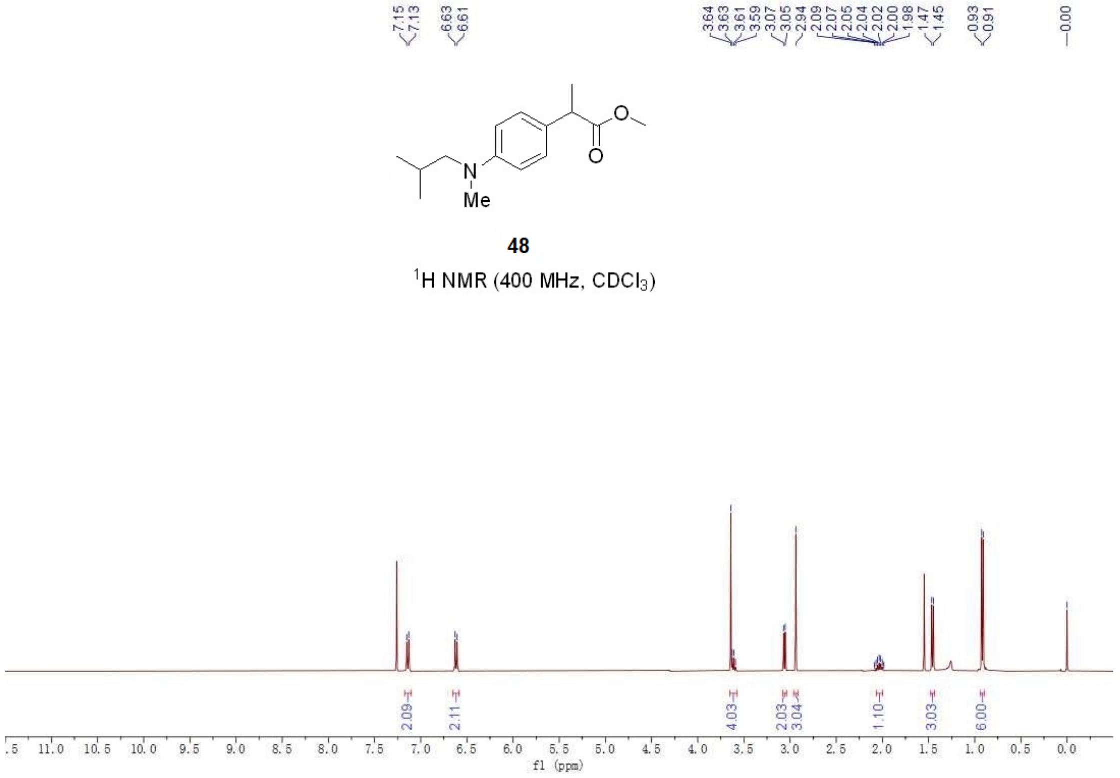

48

^{1}H NMR (400 MHz, $CDCl_3$)

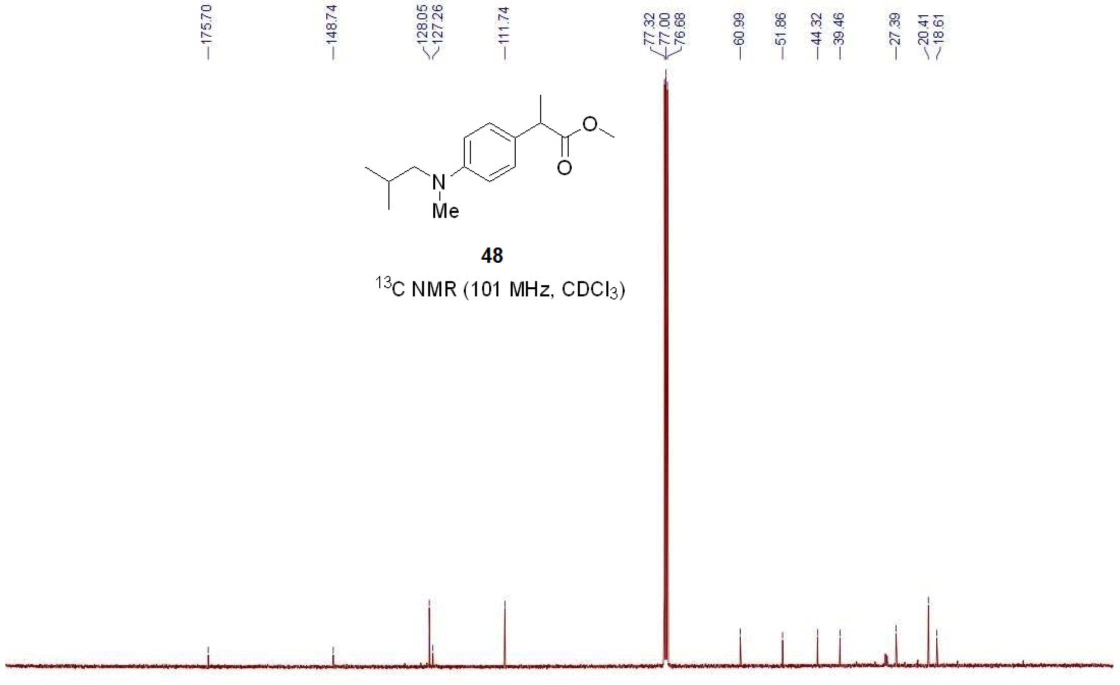

48

^{13}C NMR (101 MHz, $CDCl_3$)

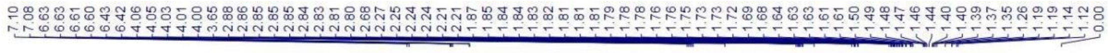

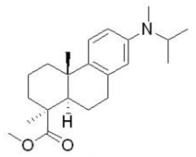

49

^{1}H NMR (400 MHz, $CDCl_3$)

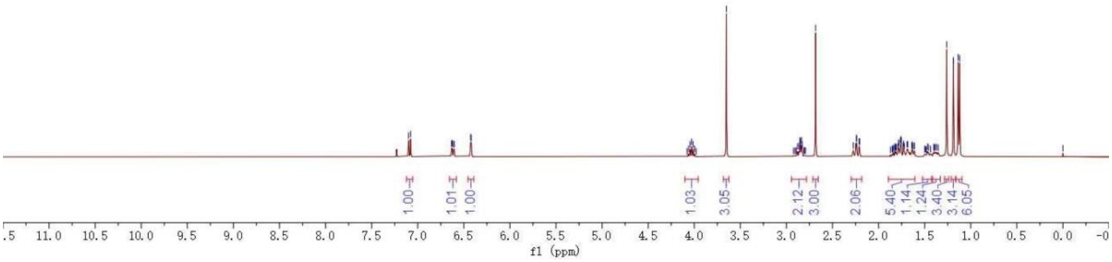

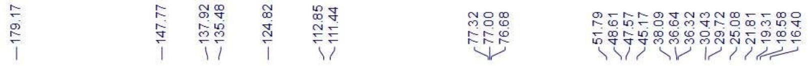

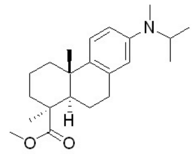

49

^{13}C NMR (101 MHz, $CDCl_3$)

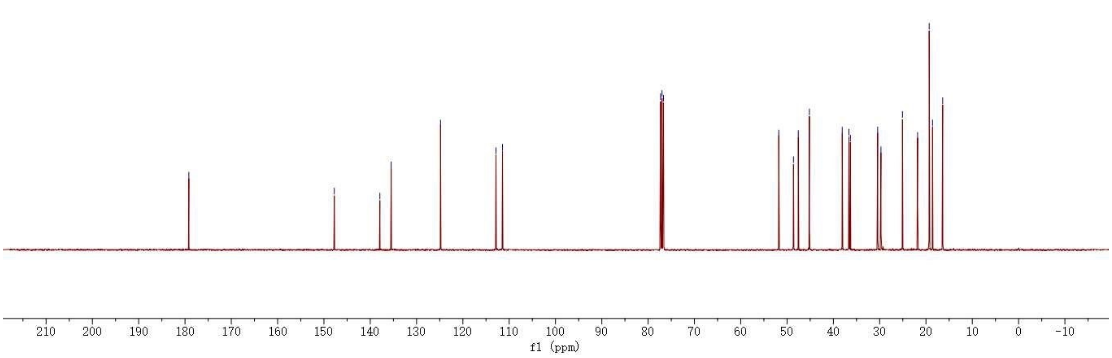

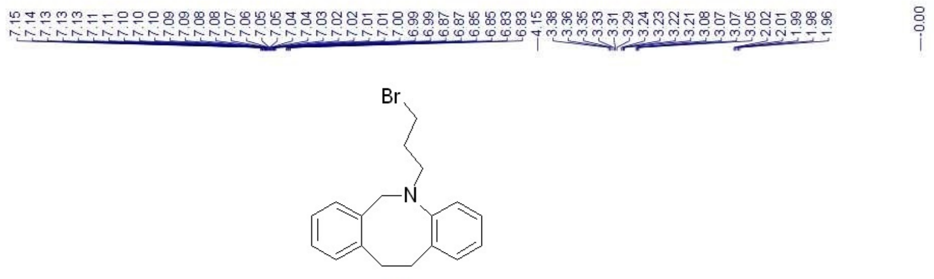

51

^{1}H NMR (400 MHz, $CDCl_3$)

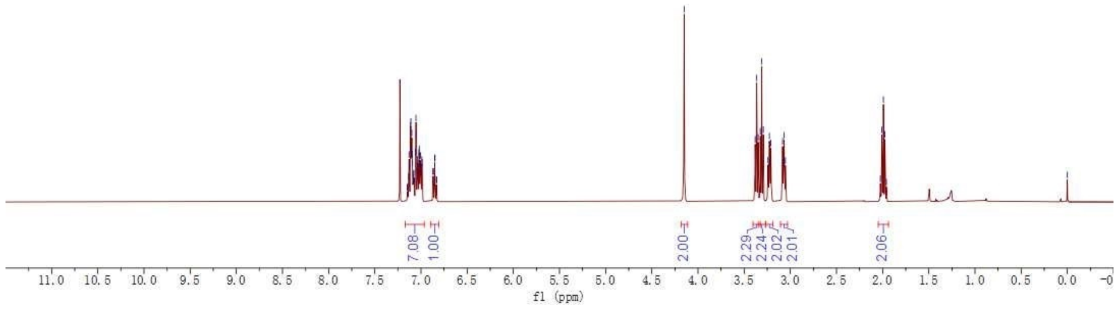

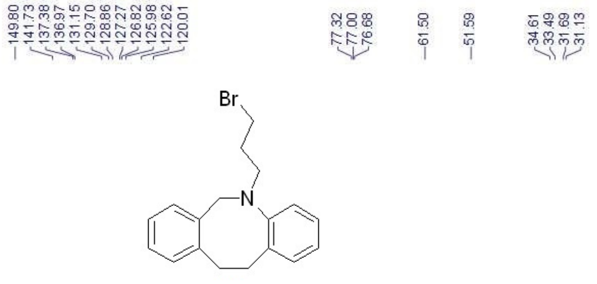

51

^{13}C NMR (101 MHz, $CDCl_3$)

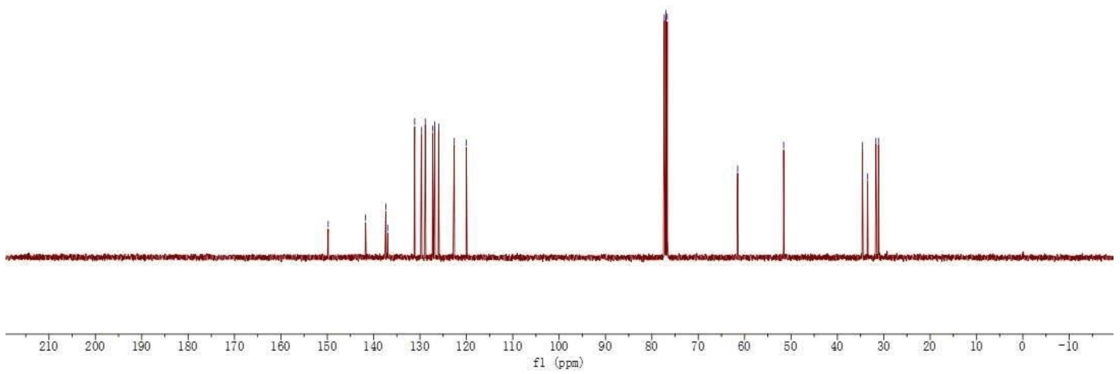

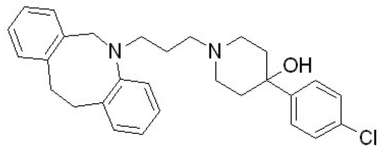

54

^{1}H NMR (400 MHz, $CDCl_3$)

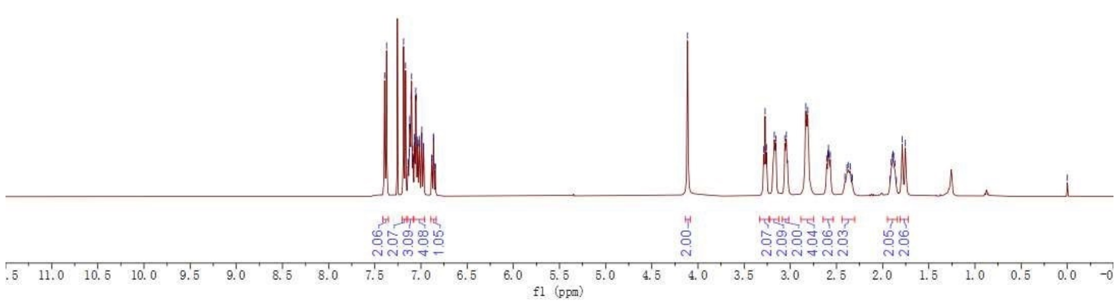

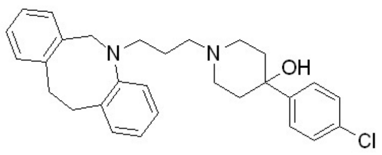

54

^{13}C NMR (101 MHz, $CDCl_3$)

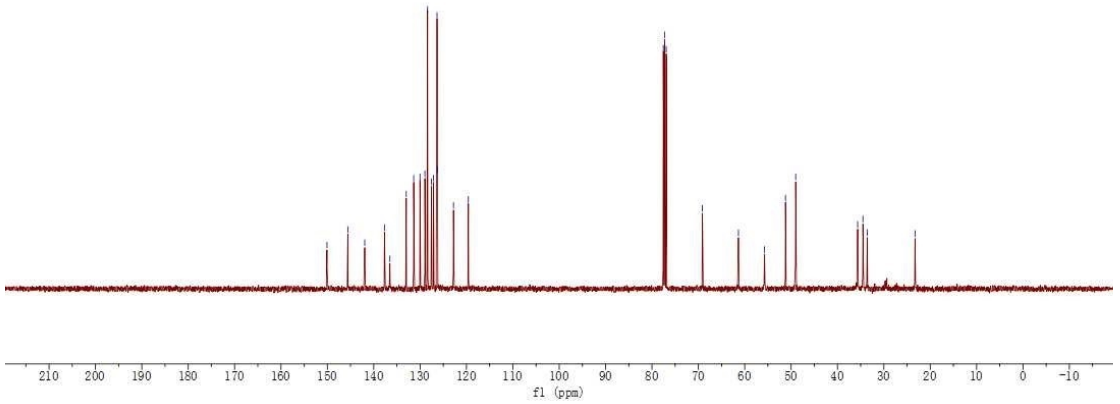

Supplementary references

[1] Miyai, T., Ueba, M. & Baba, A. Novel Synthetic Usage of Indium Compounds as Catalyst: Reductive Deoxygenation of Aryl Ketones and sec-Benzylic Alcohols. *Synlett* , **2**, 182-184 (1999).

[2] Bhanuchandra, M., Yorimitsu, H. & Osuka, A. Synthesis of Spirocyclic Diarylfluorenes by One-Pot Twofold S_NAr Reactions of Diaryl Sulfones with Diarylmethanes. *Org. Lett.* **18**, 384-387 (2016).

[3] Kato, S. et al. Hybrid Catalysis Enabling Room-Temperature Hydrogen Gas Release from N-Heterocycles and Tetrahydronaphthalenes. *J. Am. Chem. Soc.* **139**, 2204-2207 (2017).

[4] Sun, G.-J., Wang, Y. & Kang Q. Palladium-Catalyzed Allylic Amination of Homoallylic Alcohols with Amines via Carbon–Carbon Bond Cleavage. *Synthesis*, **47**, 2931-2936 (2015).

[5] Kawai, H., et al. Direct C–H bond arylation of arenes with aryltin reagents catalysed by palladium complexes. *Chem. Commun.* **12**, 1464-1466 (2008).

[6] Wang, T., et al. Hydroxylamine-mediated C–C amination via an aza-hock rearrangement. *Nat. Commun.* **12**, 7029-7039 (2021).

[7] Wang, C., et al. Site-selective desaturation of C(sp^3)–C(sp^3) bonds via photoinduced ruthenium catalysis. *Org. Chem. Front.* **9**, 4316-4327 (2022).

[8] Bhati, A. & Kale, N. Cyclization of Acid Chlorides by Polyphosphoric Acid. *Angew. Chem. Int. Ed*. **6**, 1086-1087 (1967).

[9] Ofosu-Asante, K. & Stock, L.M. Selective deuteriation of arylalkanes with mixed metal reagents. *J. Org. Chem.* **55**, 3409-3410 (1990).

[10] Kang, Q.-K., et al. Rhodium-Catalyzed Stereoselective Deuteration of Benzylic C–H Bonds via Reversible η^6-Coordination. *Angew. Chem. Int. Ed*. **61**, e202117381 (2022).

[11] Tu, J.-L., Hu, A.-M., Guo, L. & Xia, W. Iron-Catalyzed C(Sp^3)-H Borylation, Thiolation, and Sulfinylation Enabled by Photoinduced Ligand-to-Metal Charge Transfer. *J. Am. Chem. Soc.* **145**, 7600-7611 (2023).

[12] Munda, M., et al. Total syntheses of naturally occurring antiviral indolosesquiterpene alkaloids, xiamycins C-F via Csp^3-H functionalization. *Chem. Sci.* **13**, 11666-11671 (2022).

[13] Zhou, Z.-z., et al. Silver(I)-Catalyzed Widely Applicable Aerobic 1,2-Diol Oxidative Cleavage. *Angew. Chem. Int. Ed.* **57**, 2616-2620 (2018).

[14] Huang, Z., et al. Mn-Catalyzed Selective Double and Mono-N-Formylation and N-Methylation of Amines by using CO_2. *ChemSusChem*, **12**, 3054-3059 (2019).

[15] Jamsheena, V., Mahesha, C. K., Joy, M.N. & Lankalapalli, R. S. Metal-Free Diaryl Etherification of Tertiary Amines by Ortho-C(sp^2)−H Functionalization for Synthesis of Dibenzoxazepines and -ones. *Org. Lett.* **19**, 6614-6617 (2017).

[16] Coates, R. M. & Johnson, E. F. Synthesis and Base-Catalyzed Exchange of Dihydrobenzazocines. *J. Am. Chem. Soc.* **93**, 4016-4027 (1971).

[17] Reed, H., Paul, T.R. & Chain, W. J. Synthesis of Halogenated Anilines by

Treatment of N,N-Dialkylaniline N-Oxides with Thionyl Halides. *J. Org. Chem.* **83**, 11359-11368 (2018).
[18] Qiao, C., Liu, X. -F., Liu, X. & He, L.-N. Copper(II)-Catalyzed Selective Reductive Methylation of Amines with Formic Acid: An Option for Indirect Utilization of CO_2. *Org. Lett.* **19**, 1490-1493 (2017).
[19] Karnik, K. S., Sarkate, A. P., Bahekar, S. S. & Wakte, P. S. Ligand Free Microwave Assisted Copper-Catalyzed Convenient Synthesis of Substituted Tertiary Amines from Nitroarenes. *Curr. Micro. Chem.* **4**, 256-261 (2017).
[20] Mao, F., et al. Heterogeneous cobalt catalysts for reductive amination with H_2: general synthesis of secondary and tertiary amines. *RSC Adv.* **6**, 94068-94073 (2016).
[21] Lundgren, R. J., Sappong-Kumankumah, A. & Stradiotto, M. A Highly Versatile Catalyst System for the Cross-Coupling of Aryl Chlorides and Amines. *Chem. Eur. J.* **16**, 1983-1991 (2010).
[22] Matsuya, T., et al. Synthesis and evaluation of [^{11}C]RU40555, a selective glucocorticoid receptor antagonist. *J Label Compd Radiopharm*, **48**, 657-668 (2005).
[23] Vischer, H. F., et al. Identification of novel allosteric nonpeptidergic inhibitors of the human cytomegalovirus-encoded chemokine receptor US28. *Bioorg. Med. Chem.* **18**, 675-688 (2010).
[24] Liu, J., et al. From Alkylarenes to Anilines via Site-directed Carbon-carbon Amination. *Nat. Chem.* **11**, 71-77 (2019).
[25] Saidi, O., et al. Selective Amine Cross-Coupling Using Iridium-Catalyzed "Borrowing Hydrogen" Methodology. *Angew. Chem. Int. Ed.* **48**, 7375-7378 (2009).
